# Synthesis of the C1–C27 Fragment of Stambomycin D Validates Modular Polyketide Synthase-Based Stereochemical Assignments

**DOI:** 10.1021/acs.orglett.1c02650

**Published:** 2021-09-08

**Authors:** Jieyan Lim, Venkaiah Chintalapudi, Haraldur G. Gudmundsson, Minh Tran, Alice Bernasconi, Araceli Blanco, Lijiang Song, Gregory L. Challis, Edward A. Anderson

**Affiliations:** † Chemistry Research Laboratory, University of Oxford, 12 Mansfield Road, Oxford, OX1 3TA, U.K.; § Sezione Chimica Generale e Organica “A. Marchesini”, Università degli Studi di Milano, Via G. Venezian 21, 20133 Milano, Italy; ¶ Departamento de Química Orgánica, Facultad de Ciencias Químicas, Universidad de Salamanca, 37008 Salamanca, Spain; # Department of Chemistry and Warwick Integrative Synthetic Biology Centre, University of Warwick, Coventry, CV4 7AL, U.K.; ¥ Department of Biochemistry and Molecular Biology and ARC Centre of Excellence for Innovations in Peptide and Protein Science, Biomedicine Discovery Institute, Monash University, Clayton, Victoria 3800, Australia

## Abstract

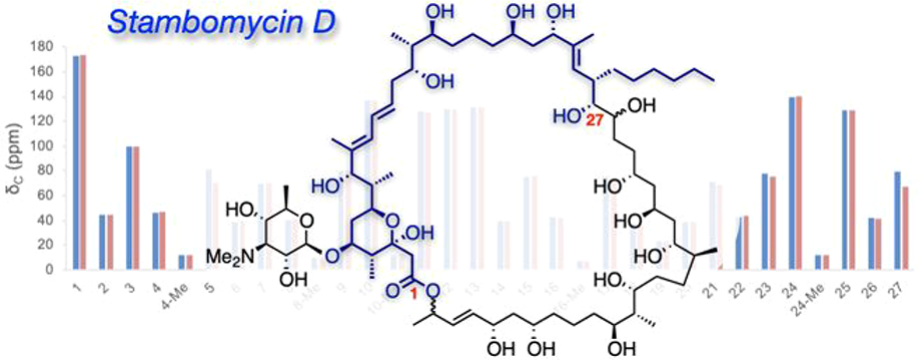

The stambomycins are a family of bioactive macrolides isolated from *Streptomyces ambofaciens*. Aside from two stereocenters installed through cytochrome P450 oxidations, their stereochemistry has been predicted by sequence analysis of the polyketide synthase. We report a synthesis of the C1–C27 fragment of stambomycin D, the spectroscopic data of which correlates well with that of the natural product, further validating predictive sequence analysis as a powerful tool for stereochemical assignment of complex polyketide natural products.

Stereochemical determination is a key element in natural product discovery, as bioactivity is often intrinsically linked to stereochemistry. It can also be one of the most challenging aspects, especially for polyketides where conformational flexibility and noncrystallinity render conclusive assignment challenging using NMR-based methods or X-ray crystallography.^1−3^ Computational approaches using NMR parameters are emerging as reliable tools^4,5^ but are unsuitable when individual stereoclusters are “insulated” by regions of flexible nonfunctionalized carbon chains, or by rigid (poly)alkene regions. NMR spectroscopy can equally be ambiguous for certain stereoclusters/conformations or complicated by overlapping signals in more complex natural products, rendering the extraction of coupling constants or nOes highly challenging and ultimately not definitive. This uncertainty provides a significant obstacle for synthesis and applications.^6^

Advances in bioinformatics have enabled the application of predictive sequence analysis of biosynthetic enzymes not only in the discovery of natural products but also in their structural and stereochemical determination.^7−11^ One example is the stambomycins, a family of 51-membered glycosylated macrolides discovered by Challis, Aigle, and co-workers in 2011 (Figure 1a).^12^ These were identified as the metabolic products of a modular polyketide synthase (PKS) in *Streptomyces ambofaciens* through a genomics-driven approach involving rational genetic manipulation to induce transcription of the biosynthetic genes, which are poorly expressed in laboratory cultures. Four members of the family (A–D) were identified, differing at the C26 side chain, all of which showed potent antibacterial and antitumor activity. The planar structures and stereochemistry of the stambomycins were predicted via sequence analysis of the modular PKS responsible for their biosynthesis,^12−14^ with the exception of the C28 and C50 stereocenters, which are of non-PKS origin.^15^ Notably, the stambomycins are one of the earliest structurally complex polyketides for which predictive sequence analysis was employed for stereochemical assignment, and remain one of the most elaborate examples to date.^16^

**Figure 1:**
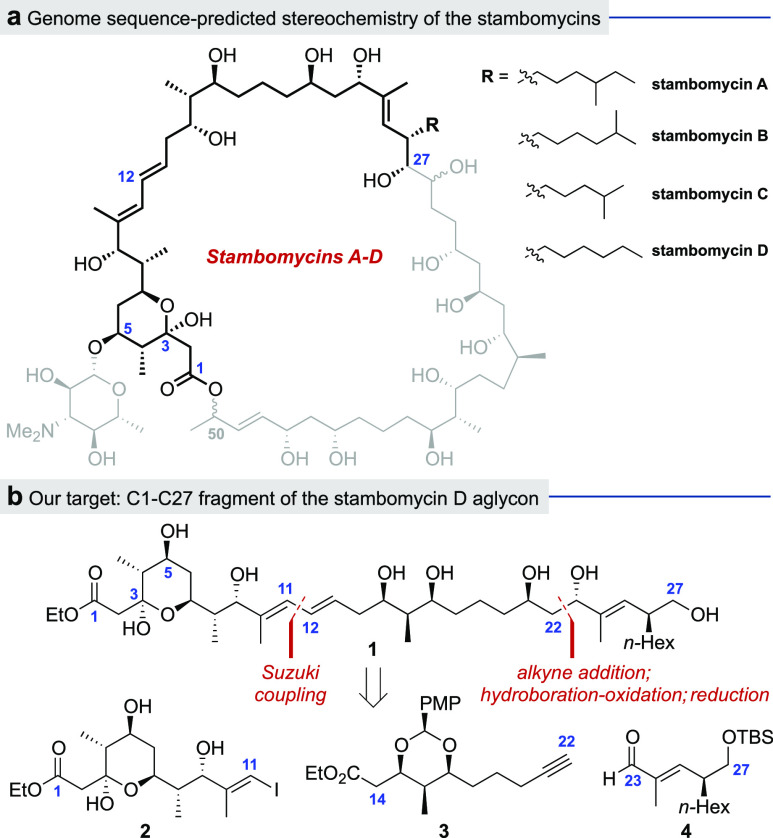
(a) Stambomycins A–D and (b) the C1–C27 fragment and planned retrosynthesis.

While the predicted planar structures of the stambomycins have been confirmed by NMR spectroscopy, their stereochemistry remains to be unequivocally confirmed. This inspired our interest in stambomycin D: a synthesis of this molecule would represent a powerful validation of sequence-based polyketide stereochemical assignment, and one that could offer a rapid and complementary approach to traditional NMR-based methods. Here, we report the synthesis of the C1–C27 aglycon fragment of stambomycin D and its comparison with the natural macrocycle. The excellent agreement between the synthetic and natural material supports the sequence-based stereochemical assignment in this region.

We envisioned that the northern and southern hemispheres of the stambomycin D aglycon would make ideal targets to establish a synthetic strategy for the entire molecule and allow a preliminary comparison of NMR data to support the predicted stereochemistry. To avoid the uncertainty of the C28 stereocenter, our initial target for the northern hemisphere consisted of the C1–C27 fragment **1** (Figure 1b). Retrosynthetically, **1** could be disconnected at the C11–C12 bond to reveal C1–C11 alkenyl iodide **2**, which could be coupled to a vinyl organometallic at C12, for example by Suzuki coupling. Disconnection at the C22–C23 bond reveals C13–C22 fragment **3** (in which the required boronic ester could be derived from manipulation of the ester group) and C23–C27 fragment **4**. Union of the latter two fragments could be achieved by asymmetric alkyne addition of **3** to **4**, followed by Hoveyda hydroboration/oxidation^17^ and reduction of the resulting propargylic alcohol to install the desired 1,3-*anti*-diol at C21/C23.

Synthesis of the C1–C11 fragment **2** (Scheme 1) commenced with an enantio- and diastereoselective Leighton crotylation^18^ of aldehyde **5** with *cis*-crotyltrichlorosilane **7** to give homoallylic alcohol **8** in 87% yield (89% *ee*, 15:1 *dr*). Cross metathesis of **8** with methyl acrylate afforded α,β-unsaturated ester **9** (90%), which was subjected to Evans–Prunet acetalization^19^ to obtain acetal **10** in 33% yield. Formation of this acetal appeared to be in an unfavorable equilibrium with the retro-Michael reaction, as the cyclization failed to reach completion even with extended reaction times; interestingly, the recovered starting material bore mainly a *Z*-alkene. This problem is attributed to the presence of the C8 (*R*)*-*methyl group, which must adopt an axial position in the six-membered cyclic acetal. Following a DIBALH reduction of the ester in **10**, a second Leighton crotylation was carried out on the resulting aldehyde, giving homoallylic alcohol **11** in 70% yield (10:1 *dr*). Protection of the alcohol as the PMB ether and subsequent oxidative cleavage of the terminal alkene afforded aldehyde **12**. A Mukaiyama aldol reaction of **12** with silyl ketene acetal **13** then gave the corresponding β-hydroxy ester (85%), which after oxidation of the alcohol, furnished β-keto ester **14** in 87% yield.

**Scheme 1:**
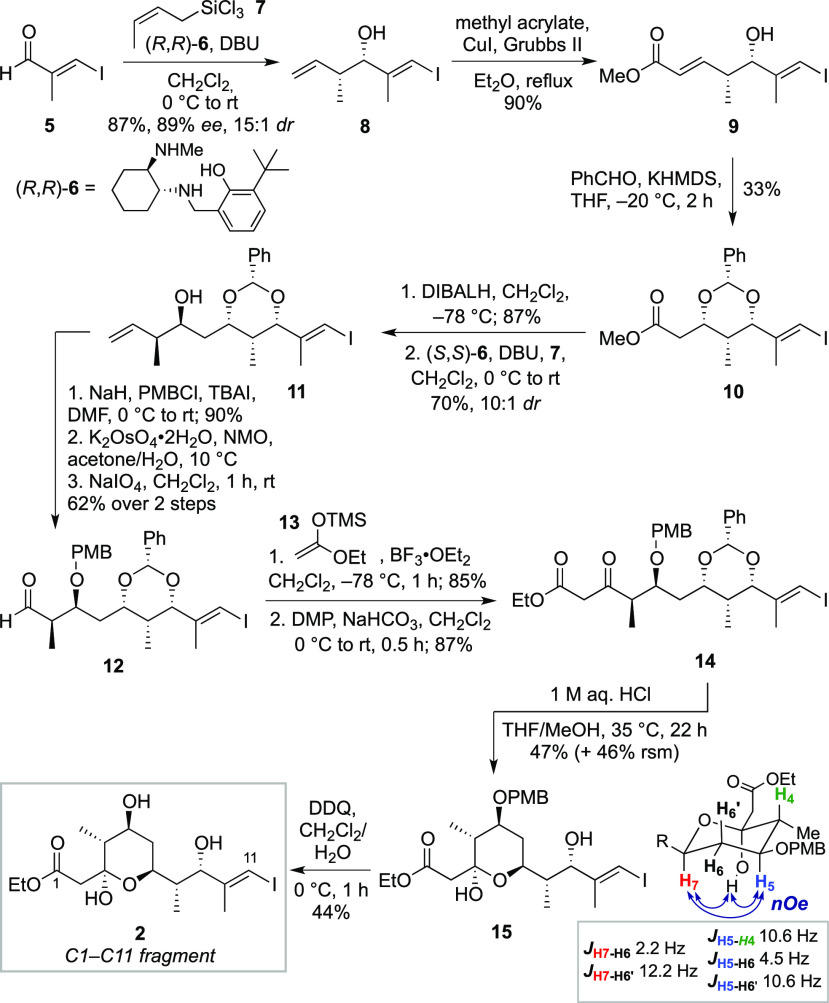
Synthesis of C1–C11 Fragment **2**

We expected that deprotection of the acetal under acidic conditions would also promote spontaneous cyclization of the resulting C7 hydroxyl group onto the C3 ketone to form the desired tetrahydropyran. This step proved unexpectedly challenging as the acetal was surprisingly robust; conditions that allowed for full conversion of the starting material also resulted in significant degradation and the formation of an unidentified side product which was difficult to separate from the product **15**. Various deprotection conditions were tested to achieve an optimal balance between conversion of the starting material and product formation, most of which involved different concentrations of aqueous HCl in MeOH/THF, as this acid was observed to give a relatively clean reaction. After fine-tuning the solvent ratio, temperature, and reaction time, it was found that use of 1.0 M aqueous HCl in MeOH/THF (1:1) at 35 °C for 22 h gave the desired tetrahydropyran **15** in 47% yield, with 46% recovered starting material. NOESY correlations and coupling constant analysis confirmed the relative stereochemistry of the various substituents on the 6-membered ring. Finally, PMB deprotection afforded the C1–C11 fragment **2** in 44% yield (12 steps from **5**).

Synthesis of the C13–C22 fragment **3** (Scheme 2a) began with 5-hexynal **16**. A Leighton crotylation^18^ was again employed to set the two adjacent stereocenters in homoallylic alcohol **17** (82%, 92% *ee*, > 20:1 *dr*). Adopting a similar strategy to that used for fragment **2**, alcohol **17** was protected as the PMB ether, with subsequent oxidative cleavage of the terminal alkene affording aldehyde **18**. A Mukaiyama aldol reaction of **18** with silyl ketene acetal **13** gave β-hydroxy ester **19** in 86% yield (5:1 *dr*); the stereochemistry of the alcohol was confirmed by Mosher ester analysis.^20^ Finally, treatment of **19** with DDQ under anhydrous conditions resulted in the formation of the 1,3-PMP acetal, giving C13–C22 fragment **3** in 55% yield after removal of the minor diastereomer (6 steps from **16**).

**Scheme 2:**
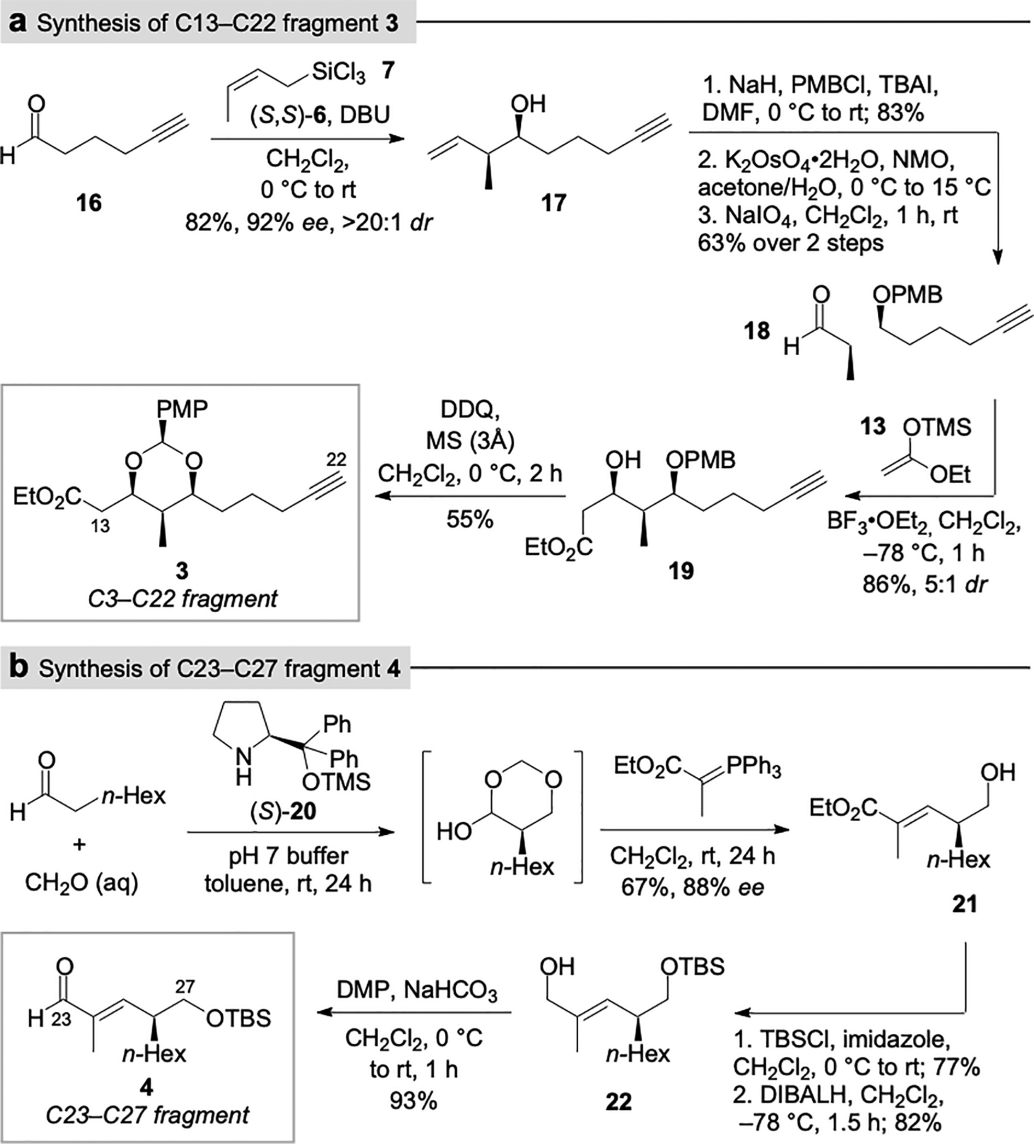
(a) Synthesis of C13–C22 Fragment **3** and (b) Synthesis of C23–C27 Fragment **4**

Attention now turned to the construction of the C23–C27 aldehyde **4** (Scheme 2b). To install the hexyl-bearing stereocenter in this fragment, an enantioselective organocatalytic aldol reaction^21^ of octanal and formaldehyde was employed at the outset. This gave a lactol intermediate, which was subjected to a Wittig olefination to obtain enoate **21** in 67% yield (88% *ee*). Protection of the alcohol as the TBS ether (77%) and DIBALH reduction of the ester (82%) gave alcohol **22**. Oxidation of alcohol **22** then afforded the C23–C27 aldehyde **4** in 93% yield.

With fragments **2**–**4** in hand, we proceeded to combine them toward the full C1–C27 fragment **1** (Scheme 3). First, a diastereoselective alkynylzinc addition^22^ of **3** to **4** afforded propargylic alcohol **23** in 62% yield (11:1 *dr*), with Mosher ester analysis confirming the stereochemistry of the alcohol. Hydroboration/oxidation of **23** employing a modification^23^ of Hoveyda’s conditions^17^ gave β-hydroxy ketone **24** in 80% yield. Following an Evans–Saksena reduction^24^ of the β-hydroxy ketone (>20:1 *dr*), the resulting 1,3-*anti*-diol was protected as the acetonide (**25**), which moreover served to confirm its stereochemistry through the Rychnovsky method.^25^ A DIBALH reduction of the ester in **25** afforded the aldehyde, which was then alkynylated using the Ohira–Bestmann reagent. During alkynylation, it was observed that the PMP acetal was prone to ring-opening, presumably via enolization of the adjacent aldehyde under the mildly basic reaction conditions (K_2_CO_3_). This resulted in the formation of a side product which not only lowered the yield of the alkyne (**26**) but also led to problems with purification. It was eventually found that use of an excess of the Ohira–Bestmann reagent overcame this problem, enabling alkyne **26** to be obtained in 81% yield.

**Scheme 3:**
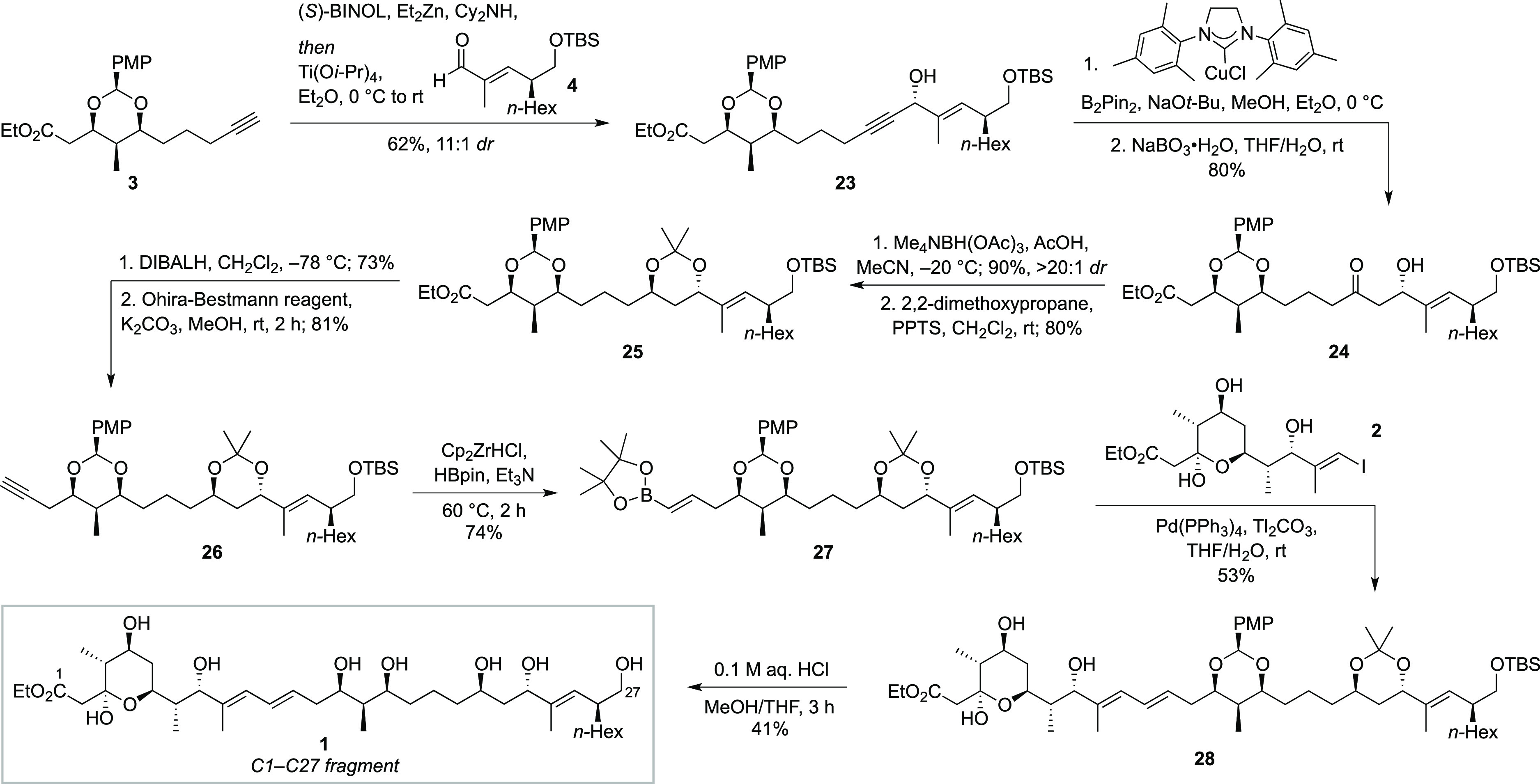
Completion of C1–C27 Fragment **1**

Following Zr-mediated hydroboration^26^ of alkyne **26**, the resulting vinylboronic ester **27** was coupled with C1–C11 fragment **2** via a Suzuki coupling. A variety of reaction conditions were screened, but use of Tl_2_CO_3_^27^ was found to be essential for reaction success, giving the complete C1–C27 framework **28** in 53% yield. Deprotection of **28** proved nontrivial, as the C10–C13 1,3-diene was observed to be highly acid-sensitive and prone to degradation, potentially via acid-promoted cyclization of the C17 hydroxyl group. After much experimentation, we found that deprotection could be achieved using 0.1 M aqueous HCl in MeOH/THF without degradation of the diene. Lower acid concentrations of 0.05 and 0.02 M could also be used, although longer reaction times were required. Treatment of **28** with 0.1 M aqueous HCl in MeOH/THF for three hours at room temperature thus afforded C1–C27 fragment **1** in 41% yield.

Having obtained C1–C27 fragment **1**, we were inspired to compare its NMR spectra with the corresponding NMR data of stambomycin D. To our delight, the data for **1** showed an excellent match with the reported^12^ data for stambomycin D (Figure 2 and Supporting Information). Although slight discrepancies existed, this is not unexpected due to potential conformational differences between the acyclic fragment and the cyclic macrolide. For example, the acyclic fragment contains a free hydroxyl at C5, whereas in the macrolide this oxygen atom is attached to the amino sugar mycaminose; in addition, the acyclic fragment is truncated at C27, as compared to the macrolide. These differences were therefore reflected in discrepancies in the ^13^C NMR data of C5 and C27. An additional discrepancy was noted at the C19 protons; re-examination of the spectroscopic data for the natural product confirmed these signals should be reassigned. Overall, there is good agreement in the ^1^H and ^13^C NMR data between the C1–C27 fragment and stambomycin D, supporting the stereochemical assignment of this region of the natural product.

**Figure 2:**
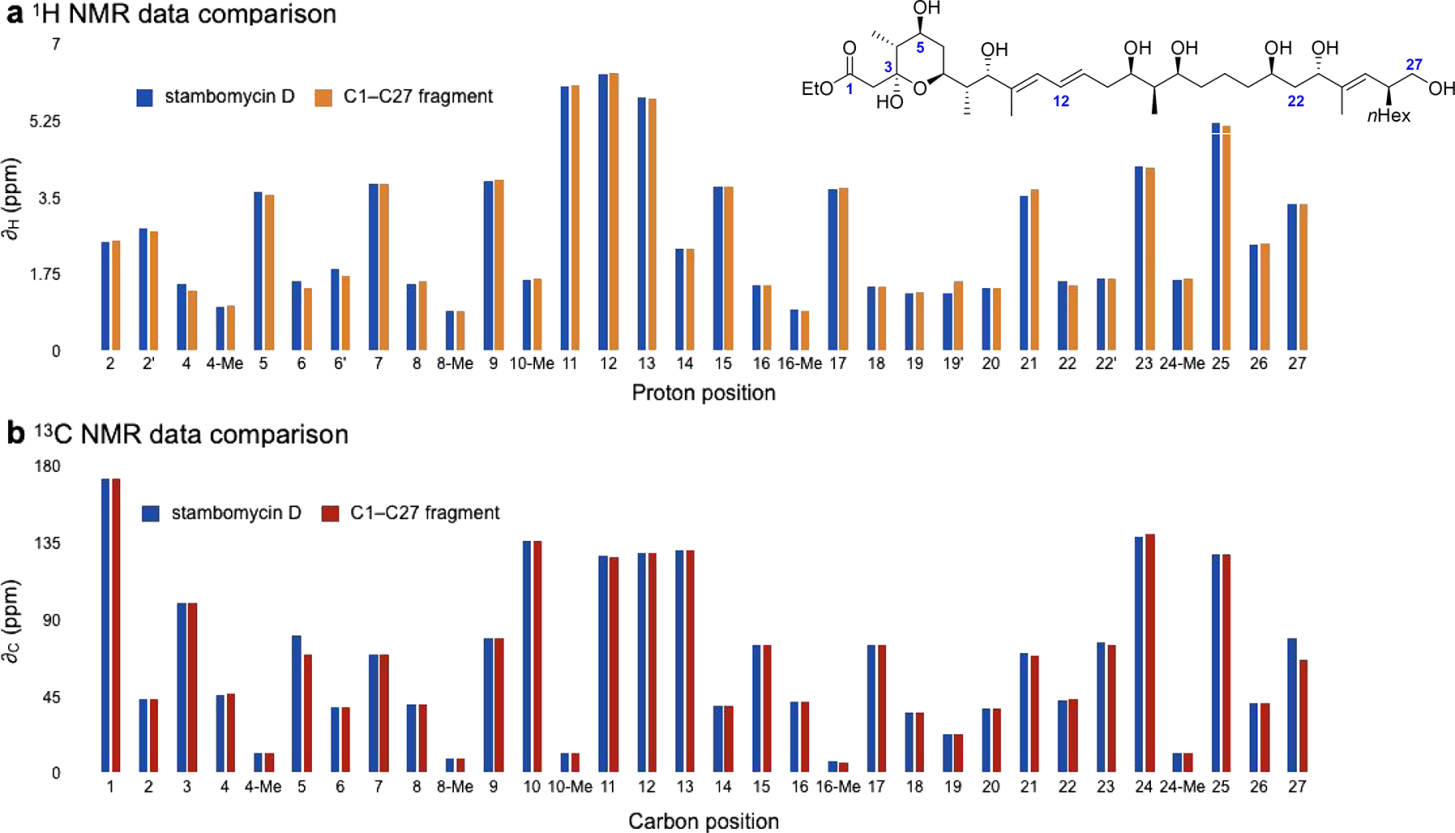
Comparison of (a) ^1^H NMR and (b) ^13^C NMR data of stambomycin D and C1–C27 fragment **1**.

In summary, we have synthesized the C1–C27 “northern” fragment of the stambomycin D aglycon. Comparison of NMR data of this fragment with the reported data of stambomycin D showed good agreement between the two, providing preliminary proof of the accuracy of the sequence-based stereochemical assignment of the macrolide.

## Supporting Information Available

Experimental procedures and characterization data for novel compounds (PDF)
