# Supplementary material for: Synthesis of the C1–C27 Fragment of Stambomycin D Validates Modular Polyketide Synthase-Based Stereochemical Assignments
Source: Org Lett. 2021 Sep 8;23(19):7439–44. doi: 10.1021/acs.orglett.1c02650 (PMC8491158; doi:10.1021/acs.orglett.1c02650)
Supplement: Supplementary file 1 — ol1c02650_si_001.pdf [file ol1c02650_si_001.pdf]

—— *Supporting Information* ——

Synthesis of the C1–C27 fragment of stambomycin D validates modular polyketide synthase-based stereochemical assignments

Jieyan Lim,[†#] Venkaiah Chintalapudi,[†#] Haraldur G. Gudmundsson,[†] Minh Tran,[†] Alice Bernasconi,[§] Araceli Blanco,[¶] Lijiang Song,[‡] Gregory C. Challis[‡¥] and Edward A. Anderson[†]*

[†]*Chemistry Research Laboratory, University of Oxford, 12 Mansfield Road, Oxford, OX1 3TA, U.K.*
[§]*Sezione Chimica Generale e Organica "A. Marchesini", Università degli Studi di Milano, Via G. Venezian 21, 20133 Milano, Italy*
[¶]*Departamento de Química Orgánica, Facultad de Ciencias Químicas, Universidad de Salamanca, Spain*
[‡]*Department of Chemistry and Warwick Integrative Synthetic Biology Centre, University of Warwick, Coventry, CV4 7AL, U.K.*
[¥]*Department of Biochemistry and Molecular Biology and ARC Centre of Excellence for Innovations in Peptide and Protein Science, Biomedicine Discovery Institute, Monash University, Clayton, Victoria 3800, Australia*

1. General information ……………………………………………………………...S2
2. Experimental procedures …………………………………………………………S3
   NMR data comparison of stambomycin D and C1–C27 fragment ……………………..S57
3. ^{1}H and ^{13}C NMR spectra ……………………………………………………..S58
4. References ……………………………………………………………………..S104

1. General information

Reagents, Solvents and Reactions

Commercial reagents and solvents were used without further purification. Anhydrous solvents were obtained from solvent dispenser units having passed through an activated alumina column under nitrogen. Reactions performed under an argon atmosphere were carried out using heat gun-dried glassware. Heated reactions were performed using an oil bath.

Chromatography

Analytical thin layer chromatography (TLC) was performed using Merck TLC Silica gel 60 F_{254} plates. TLC plates were visualised under a UV lamp (254 nm) or with vanillin or phosphomolybdic acid (PMA) stain. Flash column chromatography was carried out using Merck silica gel (technical grade, pore size 60 Å, 230-400 mesh particle size) under positive pressure. Chiral high performance liquid chromatography (HPLC) was performed on an Agilent 1200 Series running in normal phase under UV detection using a DAICEL CHIRALPAK-IC column (250 mm × 4.6 mm, particle size 3 μm).

Physical and Spectroscopic Data

Optical rotations were measured at 589 nm (Na-D line) on a Perkin-Elmer 341 polarimeter with a 1-dm pathlength cell using solutions of chloroform. Specific rotations ($[\boldsymbol{\alpha}]_{\boldsymbol{D}}^{\boldsymbol{25}}$) are given in deg dm^2g^{-1} and concentration (c) is reported in g/100 mL. Melting points were obtained using a Griffin melting point apparatus and are uncorrected. Infrared (IR) spectra were measured on a Bruker Tensor 27 Fourier FT-IR spectrometer using material applied as a thin film on a diamond/ZnSe PIKE Miracle ATR module. Absorption maxima (ν_{max}) are quoted in wavenumbers (cm^{-1}). Proton (^1H), carbon (^{13}C) and fluorine (^{19}F) NMR spectra were measured on a Bruker AVIII HD 400, AVIII HD 500 or AVIII HD 600 nuclear magnetic resonance spectrometer. Chemical shifts are reported in parts per million (ppm) with reference to the residual solvent peak ($CDCl_3$: δ 7.26/77.16; MeOD: δ 3.31/49.00). Multiplicities are reported as follows: s = singlet, brs = broad singlet, d = doublet, t = triplet, q = quartet, quint = quintet, and combinations thereof, and m = multiplet. Coupling constants are reported in hertz (Hz). High-resolution mass spectra (HRMS) were recorded on a Bruker Daltonics microTOF spectrometer (resolution = 5000 FWHM) by the University of Oxford departmental mass spectrometry service.

2. Experimental procedures

Aldehyde **5** was synthesized according to Scheme 1.

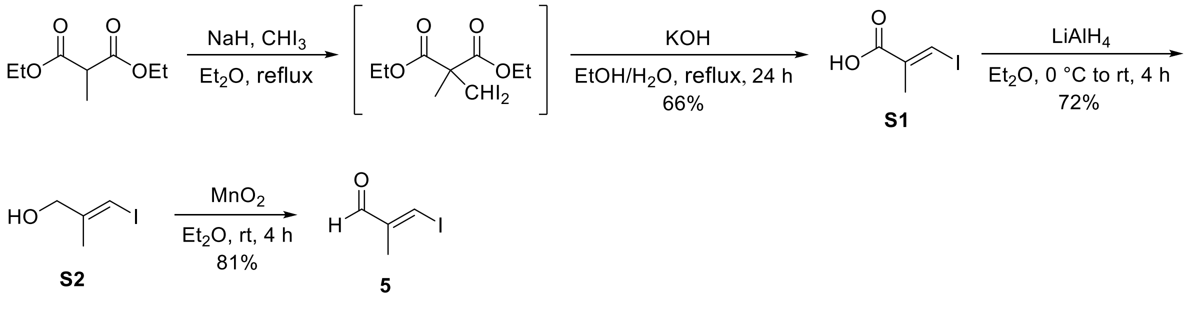

Scheme 1. *Synthesis of aldehyde* ***5***

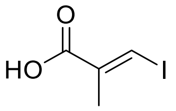

***(E)*-3-iodo-2-methylacrylic acid (S1).** Synthesized according to a modified literature procedure.[1] To a suspension of NaH (60% in mineral oil, 8.6 g, 216.0 mmol, 1.2 eq.) in anhydrous diethyl ether (112.5 mL) under argon was added diethyl methylmalonate (30.7 mL, 180.0 mmol, 1.0 eq.) dropwise over 30 minutes. The resulting thick white suspension was heated at reflux for 4 hours. Upon cooling to room temperature, iodoform (70.9 g, 180.0 mmol, 1.0 eq.) was added and the mixture was heated at reflux for 36 hours. It was cooled to 0 °C and 1.0 M aqueous HCl (120 mL) was added. The resulting dark reddish-brown solution was stirred at 0 °C for 20 minutes. The organic layer was separated and the aqueous layer was extracted with diethyl ether (3 × 60 mL). The combined organic layers were dried over Na_2SO_4 and concentrated under reduced pressure. The residue was dissolved in EtOH/H_2O (3:1, 546 mL) and KOH (27.3 g, 486.0 mmol) was added. The reaction mixture was heated at reflux for 24 hours. It was cooled to room temperature and concentrated under reduced pressure. The residue was dissolved in 10% aqueous K_2CO_3 (300 mL), and the resulting precipitated iodoform was removed by filtration, washing with 10% aqueous K_2CO_3. The filtrate was washed with CH_2Cl_2 (2 × 100 mL) and the organic layers were discarded. The aqueous layer was acidified with concentrated HCl (140 mL) and extracted with CH_2Cl_2 (7 × 40 mL). The

organic extracts were dried over Na_2SO_4 and concentrated under reduced pressure to afford the crude product (25.18 g, 118.8 mmol, 66%) as an orange oil, which solidified over time. The crude product was used in the next step without further purification. **^{1}H NMR** (400 MHz, $CDCl_3$): δ 11.77 (brs, 1H), 8.03 (q, *J* = 1.3 Hz, 1H), 2.06 (d, *J* = 1.2 Hz, 3H); **^{13}C NMR** (101 MHz, $CDCl_3$): δ 169.1, 139.1, 102.0, 19.9. The physical and spectroscopic data were consistent with literature reported data.[2]

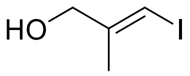

***(E)*-3-iodo-2-methylprop-2-en-1-ol (S2).** Synthesized according to a modified literature procedure.[2] To a solution of acid **S1** (10.28 g, 48.5 mmol, 1.0 eq.) in anhydrous diethyl ether (32 mL) at 0 °C under argon was added $LiAlH_4$ (4.0 M in diethyl ether, 12.6 mL, 50.4 mmol, 1.04 eq.) dropwise. The reaction mixture was stirred at room temperature for 4 hours. It was cooled to 0 °C and quenched with successive dropwise addition of water (2.1 mL), 15% aqueous NaOH (2.1 mL), and water (6.4 mL). The mixture was stirred at room temperature for 30 minutes, dried over $MgSO_4$, filtered and concentrated under reduced pressure. The residue was purified by flash chromatography on silica gel (pentane/diethyl ether = 4/1) to afford the title compound (6.93 g, 35.0 mmol, 72%) as a colourless oil. **^{1}H NMR** (400 MHz, $CDCl_3$): δ 6.29 (q, *J* = 1.2 Hz, 1H), 4.14 (dq, *J* = 6.2, 0.5 Hz, 2H), 1.85 (dt, *J* = 1.2, 0.5 Hz, 3H), 1.54 (t, *J* = 6.2 Hz, 1H); **^{13}C NMR** (101 MHz, $CDCl_3$): δ 147.4, 77.4, 67.4, 21.5. The physical and spectroscopic data were consistent with literature reported data.[2]

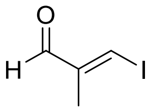

***(E)*-3-iodo-2-methylacrylaldehyde (5).** Synthesized according to a modified literature procedure.[3] To a solution of alcohol **S2** (5.0 g, 25.3 mmol, 1 eq.) in anhydrous diethyl ether (101.0 mL) under argon was added MnO_2 (33.0 g, 379.5 mmol, 15.0 eq.). The reaction mixture was stirred at room temperature for 4 hours. Celite (~16 g) was added and the mixture was stirred for 20 minutes. It was filtered through celite and concentrated under reduced pressure. The residue was purified by flash chromatography on silica gel (pentane/diethyl ether = 9/1) to afford the title compound (4.0 g, 20.4 mmol, 81%) as a light yellow oil. **^{1}H NMR** (400 MHz, $CDCl_3$): δ 9.53 (s, 1H), 7.81 (q, J = 1.2 Hz, 1H), 1.92 (d, J = 1.2 Hz, 3H); **^{13}C NMR** (101 MHz, $CDCl_3$): δ 189.5, 151.0, 109.5, 16.6. The physical and spectroscopic data were consistent with literature reported data.[3]

The diaminophenol ligand (*R*,*R*)-**6** was synthesized according to Scheme 2, following a literature reported route.[4] Ligand (*S*,*S*)-**6'** was likewise synthesized following the same route.

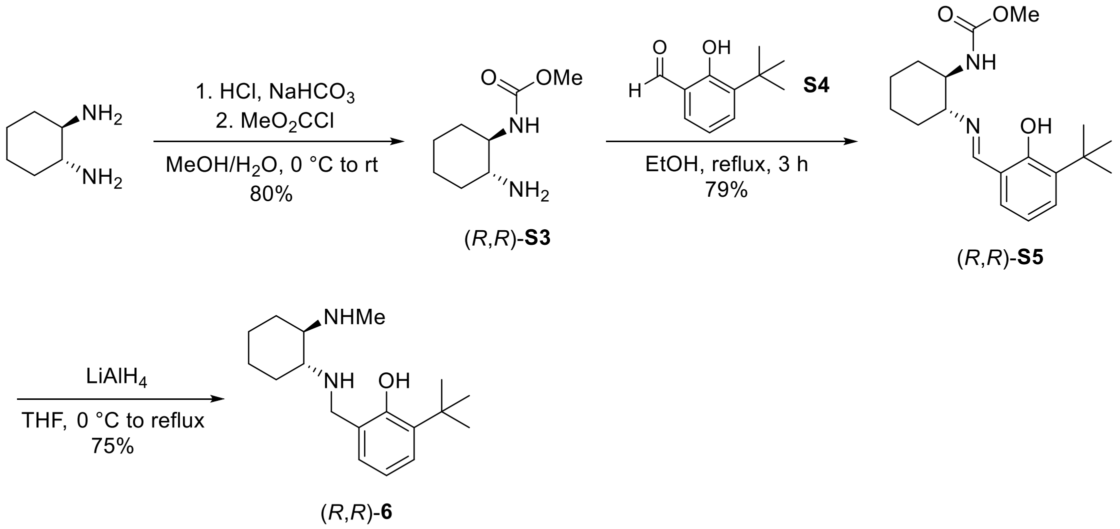

Scheme 2**. Synthesis of diaminophenol ligand (R,R)-**6

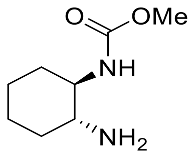

Methyl ((1*R*,2*R*)-2-aminocyclohexyl)carbamate ((*R*,*R*)-S3). Synthesized according to a literature procedure.[4] To a solution of concentrated HCl (9.0 mL, 110.0 mmol, 1.0 eq.) in MeOH (47.8 mL) at 0 °C was added (*R*,*R*)-(–)-1,2-diaminocyclohexane (12.6 g, 110.0 mmol, 1.0 eq.). The mixture was stirred at room temperature for 15 minutes, after which water (14.5 mL) and $NaHCO_3$ (11.1 g, 132.0 mmol, 1.2 eq.) were added. After stirring for an additional 30 minutes, the resulting thick mixture was cooled to 0 °C. A solution of methyl chloroformate (8.5 mL, 110.0 mmol, 1.0 eq.) in MeOH (11.3 mL) was added dropwise and the reaction mixture was stirred at 0 °C overnight, allowing the ice bath to warm to room temperature. The mixture was diluted with water (60 mL) and extracted with diethyl ether (3 × 60 mL). The organic layers were discarded and the aqueous layer was treated with 15% aqueous NaOH until the pH was >10. It was then extracted with CH_2Cl_2 (5 × 60 mL); the organic extracts were dried over Na_2SO_4 and concentrated under reduced pressure to afford the title compound (15.2 g, 88.3 mmol, 80%) as a white solid. **^{1}H NMR** (400 MHz, $CDCl_3$): δ 4.62 (brs, 1H), 3.67 (s,

3H), 3.21 – 3.13 (m, 1H), 2.37 – 2.31 (m, 1H), 2.03 – 1.92 (m, 2H), 1.73 – 1.68 (m, 2H), 1.31 – 1.09 (m, 4H); **^{13}C NMR** (101 MHz, $CDCl_3$): δ 157.4, 58.3, 55.7, 52.2, 35.5, 33.0, 25.3, 25.2. The physical and spectroscopic data were consistent with literature reported data.[5]

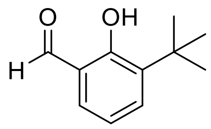

3-(*tert*-Butyl)-2-hydroxybenzaldehyde (S4). Synthesized according to a literature procedure.[6] To a solution of 2-*tert*-butylphenol (30.7 mL, 0.20 mol, 1.0 eq.) in anhydrous acetonitrile (400 mL) was added paraformaldehyde (40.5 g, 1.35 mol, 6.75 eq.), $MgCl_2$ (28.6 g, 0.30 mol, 1.5 eq.) and anhydrous Et_3N (104.3 mL, 0.75 mol, 3.74 eq.). The reaction mixture was heated at reflux for 5 hours. Upon cooling to room temperature, 5% aqueous HCl (200 mL) was added and the mixture was extracted with CH_2Cl_2 (3 × 125 mL). The organic layers were concentrated under reduced pressure and the residue was partitioned between diethyl ether (250 mL) and water (250 mL). The organic layer was washed with brine (100 mL), dried over Na_2SO_4 and concentrated under reduced pressure. The resulting oil was purified by vacuum distillation (b.p.: 94 °C @ ~ 2 mbar) to afford the title compound (21.0 g, 0.12 mol, 59%) as a pale yellow oil. **^{1}H NMR** (400 MHz, $CDCl_3$): δ 11.79 (s, 1H), 9.88 (s, 1H), 7.53 (dd, J = 7.7, 1.5 Hz, 1H), 7.40 (dd, J = 7.7, 1.7 Hz, 1H), 6.95 (t, J = 7.7 Hz, 1H), 1.42 (s, 9H); **^{13}C NMR** (101 MHz, $CDCl_3$): δ 197.3, 161.4, 138.4, 134.2, 132.1, 120.8, 119.3, 35.0, 29.3. The physical and spectroscopic data were consistent with literature reported data.[6]

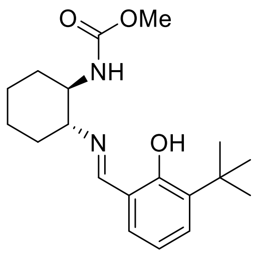

Methyl ((1*R*,2*R*)-2-(((*E*)-3-(*tert*-butyl)-2-hydroxybenzylidene)amino)cyclohexyl)carbamate ((*R*,*R*)-S5). Synthesized according to a literature procedure.[4] To a solution of amine (*R*,*R*)-**S3** (15.2 g, 88.1 mmol, 1.0 eq.) in EtOH (294 mL) was added aldehyde **S4** (17.3 g, 96.9 mmol, 1.1 eq.). The reaction mixture was heated at reflux for 3 hours. It was cooled to room

temperature and concentrated under reduced pressure. The resulting solid was recrystallized from minimal boiling hexanes to afford the title compound (23.0 g, 69.2 mmol, 79%) as light yellow crystals.

m.p. 123–125 °C; $[\alpha]_D^{25}$ –94.9 (c = 1.2, $CHCl_3$); **^{1}H NMR** (400 MHz, $CDCl_3$): δ 13.7 (s, 1H), 8.32 (s, 1H), 7.31 (dd, *J* = 7.7, 1.7 Hz, 1H), 7.09 (dd, *J* = 7.6, 1.7 Hz, 1H), 6.80 (t, *J* = 7.6 Hz, 1H), 4.53 (brs, 1H), 3.69 – 3.61 (m, 1H), 3.56 (s, 3H), 3.10 (brs, 1H), 2.12 (d, *J* = 10.4 Hz, 1H), 1.90 – 1.75 (m, 3H), 1.74 – 1.64 (m, 1H), 1.43 (s, 9H), 1.43 – 1.29 (m, 4H); **^{13}C NMR** (101 MHz, $CDCl_3$): δ 165.1, 160.5, 137.5, 129.9, 129.5, 118.7, 117.9, 55.1, 34.9, 33.6, 31.8, 29.5, 24.8, 24.2. The physical and spectroscopic data were consistent with literature reported data.[4]

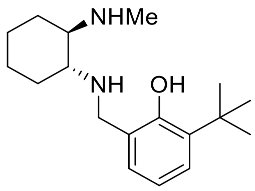

2-(*tert*-Butyl)-6-((((1*R*,2*R*)-2-(methylamino)cyclohexyl)amino)methyl)phenol ((*R*,*R*)-6). Synthesized according to a literature procedure.[4] To a suspension of $LiAlH_4$ (7.9 g, 207.9 mmol, 3.0 eq.) in anhydrous THF (540 mL) at 0 °C under argon was added a solution of imine (*R*,*R*)-**S5** (23.0 g, 69.3 mmol, 1.0 eq.) in anhydrous THF (150 mL) dropwise via addition funnel. The reaction mixture was stirred at room temperature for 1 hour and at reflux overnight. It was cooled to 0 °C and quenched with successive dropwise addition of water (7.9 mL), 15% aqueous NaOH (7.9 mL), and water (23.7 mL). The mixture was stirred at room temperature for 10 minutes, $MgSO_4$ was added, and it was stirred for another 20 minutes. It was filtered and concentrated under reduced pressure. The resulting solid was recrystallized from minimal boiling hexanes to afford the title compound (15.1 g, 52.0 mmol, 75%) as white crystals.

m.p. 116–118 °C; $[\alpha]_D^{25}$ –112.5 (c = 1.1, $CHCl_3$); **IR** (thin film): ν_{max}/cm^{-1} 3223, 2930, 2856, 1434, 1242, 1087, 749; **^{1}H NMR** (400 MHz, $CDCl_3$): δ 7.19 (dd, *J* = 7.9, 1.7 Hz, 1H), 6.88 (dd, *J* = 7.3, 1.7 Hz, 1H), 6.72 (t, *J* = 7.6 Hz, 1H), 4.03 (d, *J* = 13.5 Hz, 1H), 3.85 (d, *J* = 13.5 Hz, 1H), 2.41 (s, 3H), 2.21 – 2.14 (m, 4H), 1.79 – 1.71 (m, 2H), 1.45 (s, 9H), 1.29 – 1.18 (m, 3H), 1.02 – 0.92 (m, 1H); **^{13}C NMR** (101 MHz, $CDCl_3$): δ 157.4, 136.8, 126.2, 125.7, 124.4, 118.1, 62.4, 62.2, 51.0, 34.8, 33.5, 31.2, 31.1, 29.7, 25.3, 24.8; **HRMS** (ESI) m/z calcd for

$C_{18}H_{30}N_2O$ $[M+H]^+$: 291.2431, found 291.2429. The physical and spectroscopic data were consistent with literature reported data.[4]

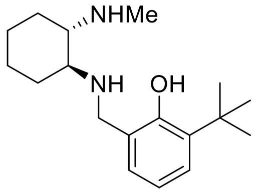

2-(*tert*-Butyl)-6-((((1*S*,2*S*)-2-(methylamino)cyclohexyl)amino)methyl)phenol ((*S*,*S*)-6'). Synthesized from (*S*,*S*)-(+)-1,2-diaminocyclohexane following the same route as (*R*,*R*)-**S6**. White crystals, 73%. **m.p.** 116–118 °C; $[\alpha]_D^{25}$ +112.4 (c = 0.8, $CHCl_3$); **IR** (thin film): ν_{max}/ cm^{-1} 3223, 2930, 2856, 1434, 1242, 1087, 749; **^{1}H NMR** (400 MHz, $CDCl_3$): δ 7.17 (dd, *J* = 7.9, 1.7 Hz, 1H, ArH), 6.88 (dd, J = 7.3, 1.7 Hz, 1H), 6.70 (t, *J* = 7.6 Hz, 1H), 4.02 (d, *J* = 13.5 Hz, 1H), 3.83 (d, *J* = 13.5 Hz, 1H), 2.39 (s, 3H), 2.24 – 2.09 (m, 4H), 1.78 – 1.70 (m, 2H), 1.42 (s, 9H), 1.29 – 1.17 (m, 3H), 1.02 – 0.91 (m, 1H); **^{13}C NMR** (101 MHz, $CDCl_3$): δ 157.5, 136.9, 126.2, 125.7, 124.4, 118.1, 62.4, 62.2, 51.0, 34.8, 33.6, 31.2, 31.1, 29.7, 25.3, 24.8; **HRMS** (ESI) m/z calcd for $C_{18}H_{30}N_2O$ $[M+H]^+$: 291.2431, found 291.2430. The physical and spectroscopic data were consistent with literature reported data.[4]

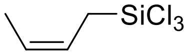

***cis*-Crotyltrichlorosilane (7).** Synthesized according to a modified literature procedure.[6] To a solution of $Pd(PPh_3)_4$ (289 mg, 0.25 mmol, 0.25 mol%) in anhydrous THF (25 mL) under argon at –78 °C was added 1,3-butadiene (2.0 M in THF, 50.0 mL, 100.0 mmol, 1.0 eq.) and trichlorosilane (12.1 mL, 120.0 mmol, 1.2 eq.). The reaction mixture was stirred at –78 °C for 15 minutes and at room temperature for 24 hours. The solvent was removed by distillation and the residue was purified by vacuum distillation (b.p. 54 °C @ ~ 40 mbar) to afford the title compound (15.5 g, 81.8 mmol, 82%) as a colourless oil. **^{1}H NMR** (400 MHz, $CDCl_3$): δ 5.77 – 5.68 (m, 1H), 5.46 – 5.38 (m, 1H), 2.36 (ddq, *J* = 8.2, 1.3, 0.8 Hz, 2H), 1.66 (ddt, *J* = 6.8, 1.7, 0.8 Hz, 3H); **^{13}C NMR** (101 MHz, $CDCl_3$): δ 128.5, 118.7, 24.9, 13.2. The physical and spectroscopic data were consistent with literature reported data.[6]

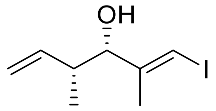

(3*S*,4*R*,*E*)-1-iodo-2,4-dimethylhexa-1,5-dien-3-ol (8). Synthesized according to a modified literature procedure.[6] To a solution of (*R*,*R*)-**6** (8.36 g, 28.8 mmol, 1.1 eq.) in anhydrous CH_2Cl_2 (105 mL) at 0 °C under argon was added DBU (12.9 mL, 86.5 mmol, 3.3 eq.), followed by slow addition of *cis*-crotyltrichlorosilane **7** (5.95 g, 31.4 mmol, 1.2 eq.). The mixture was stirred at room temperature for 1 hour, after which it was recooled to 0 °C. Aldehyde **5** (5.13 g, 26.2 mmol, 1.0 eq.) was added dropwise and the reaction mixture was stirred at 0 °C for 1 hour. The mixture was treated with TBAF (1.0 M in THF, 34.0 mL, 34.0 mmol, 1.3 eq.) at 0 °C, stirred at room temperature for 1 hour and concentrated under reduced pressure. The residue was suspended in diethyl ether (125 mL) and the mixture was stirred vigorously for 20 minutes. The resulting precipitated DBU.HCl salts were removed by filtration. The filtrate was treated with 1 M HCl (143.9 mL, 143.9 mmol, 5.5 eq.) and the mixture was extracted with diethyl ether (3 × 140 mL). The organic layers were washed with water (2 × 140 mL) and saturated aqueous $NaHCO_3$ (140 mL), dried over Na_2SO_4 and concentrated under reduced pressure. The residue was purified by flash chromatography on silica gel (pentane/diethyl ether = 9/1) to afford the title compound (5.77 g, 22.9 mmol, 87%, 89% *ee*,[a] 15:1 *dr*) as a colourless oil.

$[\boldsymbol{\alpha}]_{\boldsymbol{D}}^{\mathbf{25}}$ –8.2 (c = 1.0, $CHCl_3$); $\mathbf{R_f}$ 0.30 (pentane/diethyl ether = 4/1); **IR** (thin film): ν_{max}/cm^{-1} 3380, 3079, 2975, 2928, 1640, 1615, 1455, 1377, 1266, 1011, 917, 783, 665; **^{1}H NMR** (400 MHz, $CDCl_3$): δ 6.26 (app t, *J* = 1.0 Hz, 1H, *H6*), 5.72 (ddd, *J* = 17.1, 10.5, 7.1 Hz, 1H, *H2*), 5.10 (app dt, *J* = 8.1, 1.2 Hz, 1H, *H1*), 5.06 (app d, *J* = 1.2 Hz, 1H, *H1'*), 4.04 (dd, *J* = 5.9, 3.5 Hz, 1H, H*4*), 2.49 – 2.40 (m, 1H, *H3*), 1.80 (d, *J* = 1.0 Hz, 3H, CCH_3), 1.71 (d, *J* = 3.5 Hz, 1H, O*H*), 1.01 (d, *J* = 6.8 Hz, 3H, $CHCH_3$); **^{13}C NMR** (101 MHz, $CDCl_3$): δ 148.4, 140.1, 115.6, 79.8, 79.1, 41.1, 20.7, 14.1; **HRMS** not found.

[a]Determined by ^{19}F NMR analysis of the Mosher esters of **8**.

<u>Recovery of (*R*,*R*)-6 ligand</u>

The combined aqueous acid and water layers were treated with 1 M NaOH (287.9 mL, 287.9 mmol, 10 eq. with respect to the ligand) and extracted with CH_2Cl_2 (5 × 140 mL). The organic layers were washed with water (2 × 142 mL), dried over Na_2SO_4 and concentrated under reduced pressure. The resulting solid was recrystallized from minimal boiling hexanes to afford the recovered (*R*,*R*)-**6** ligand (6.96 g, 24.0 mmol, 83%) as white crystals.

Determination of *ee* by ^{19}F NMR of the Mosher ester: 89% *ee*

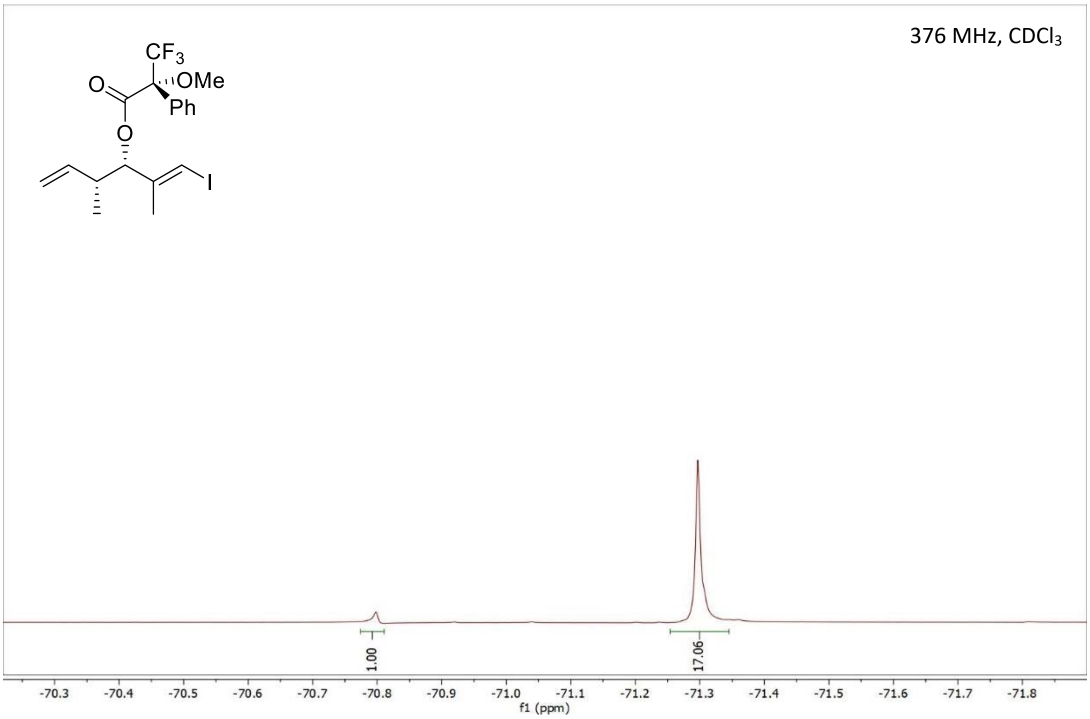

Mosher ester analysis of alcohol 8. Alcohol **8** was derivatized to the (*R*)-MTPA and (*S*)-MTPA esters using (*S*)-(+)-MTPA-Cl and (*R*)-(–)-MTPA-Cl respectively according to a modified literature procedure.[7]

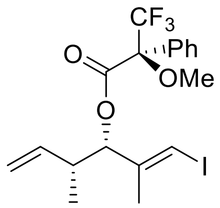

(*S*)-MTPA ester of 8. To a solution of alcohol **8** (10.0 mg, 0.040 mmol, 1.0 eq.) in CH_2Cl_2 (800 µL) was added pyridine (19.6 mg, 0.248 mmol, 6.2 eq.) and (*R*)-(–)-α-methoxy-α-(trifluoromethyl)phenylacetyl chloride (38.4 mg, 0.152 mmol, 3.8 eq.). The reaction mixture was stirred at room temperature overnight. Water was added and the mixture was extracted with CH_2Cl_2. The organic layers were dried over Na_2SO_4 and concentrated under reduced pressure. The residue was purified by flash chromatography on silica gel (pentane/diethyl ether = 49/1) to afford the title compound (18.3 mg, 0.039 mmol, 98%) as a colourless oil.

$[\boldsymbol{\alpha}]_{\boldsymbol{D}}^{\mathbf{25}}$ –35.1 (c = 1.8, $CHCl_3$); **R**$_f$ 0.74 (pentane/diethyl ether = 4/1); **IR** (thin film): ν_{max}/cm^{-1} 2980, 2360, 1749, 1271, 1242, 1170, 1121, 1016, 994; **^{1}H NMR** (400 MHz, $CDCl_3$): δ 7.47 – 7.40 (m, 5H, Ar*H*), 6.39 – 6.38 (m, 1H, *H6*), 5.55 (ddd, *J* = 17.0, 10.5, 7.7 Hz, 1H, *H2*), 5.34 (d, *J* = 8.4 Hz, 1H, *H4*), 5.05 (app dt, *J* = 7.4, 1.1 Hz, 1H, *H1*), 5.02 (app d, *J* = 1.1 Hz, 1H, *H1'*), 3.49 (s, 3H, OCH_3), 2.60 – 2.50 (m, 1H, *H3*), 1.78 (d, *J* = 1.1 Hz, 3H, CCH_3), 0.93 (d, *J* = 6.7 Hz, 3H, $CHCH_3$); **^{13}C NMR** (101 MHz, $CDCl_3$): δ 166.0, 143.9, 137.8, 132.3, 129.8, 128.6, 127.5, 123.5 (q, *J* = 290 Hz), 116.4, 84.7 (q, *J* = 28 Hz), 83.5, 83.1, 55.5, 40.0, 20.6, 15.7; **^{19}F NMR** (376 MHz, $CDCl_3$): δ –70.8; **HRMS** (ESI) m/z calcd for $C_{18}H_{20}F_3IO_3$ $[M+Na]^+$: 491.0301, found 491.0300.

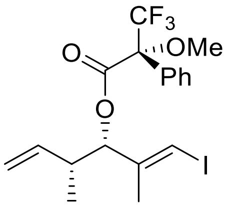

(*R*)-MTPA ester of 8. Synthesized in the same manner as the (*S*)-MTPA ester of **8** using 0.042 mmol of alcohol **8** and substituting (*R*)-(–)-α-methoxy-α-(trifluoromethyl)phenylacetyl chloride with (*S*)-(+)-α-methoxy-α-(trifluoromethyl)phenylacetyl chloride. The title compound (19.5 mg, 0.042 mmol, 99%) was obtained as a colourless oil.

$[\alpha]_D^{25}$ +23.9 (c = 1.9, $CHCl_3$); **R_f** 0.74 (pentane/diethyl ether = 4/1); **IR** (thin film): ν_{max}/cm^{-1} 2980, 2360, 1748, 1272, 1247, 1169, 1123, 1016, 994; **^{1}H NMR** (400 MHz, $CDCl_3$): δ 7.47 – 7.40 (m, 5H, Ar*H*), 6.27 – 6.21 (m, 1H, *H6*), 5.59 (ddd, *J* = 17.2, 10.4, 7.5 Hz, 1H, *H2*), 5.27 (d, *J* = 8.0 Hz, 1H, *H4*), 5.08 (app dt, *J* = 7.9, 1.1 Hz, 1H, *H1*), 5.04 (app d, *J* = 1.1 Hz, 1H, *H1'*), 3.57 (s, 3H, OCH_3), 2.62 – 2.52 (m, 1H, *H3*), 1.62 (d, *J* = 1.1 Hz, 3H, CCH_3), 1.05 (d, *J* = 6.7 Hz, 3H, CHCH_3); **^{13}C NMR** (101 MHz, $CDCl_3$): δ 165.9, 143.7, 138.1, 132.2, 129.8, 128.6, 127.3, 123.4 (q, *J* = 290 Hz), 116.4, 84.8 (q, *J* = 28 Hz), 83.2, 83.0, 56.0, 39.7, 20.6, 15.7; **^{19}F NMR** (376 MHz, $CDCl_3$): δ –71.3; **HRMS** (ESI) m/z calcd for $C_{18}H_{20}F_3IO_3$ $[M+Na]^+$: 491.0301, found 491.0299.

Comparison of the ^{1}H NMR data of the two Mosher ester derivatives is shown below.

Assignment	δ, (*S*)-ester (ppm)	δ, (*R*)-ester (ppm)	$\Delta\delta^{SR}$
**H1**	5.05	5.08	–0.03
**H1'**	5.02	5.04	–0.02
**H2**	5.55	5.59	–0.04
**H3**	2.55	2.57	–0.02
**CHCH_3**	0.93	1.05	–0.12
**CCH_3**	1.78	1.62	+0.16
**H6**	6.38	6.24	+0.14

Analysis indicates that the C4 centre is of *S* configuration.

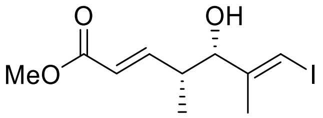

Methyl (2*E*,4*R*,5*S*,6*E*)-5-hydroxy-7-iodo-4,6-dimethylhepta-2,6-dienoate (9). To an oven-dried flask under argon was added alcohol **8** (5.77 g, 22.9 mmol, 1.0 eq.), methyl acrylate (6.2 mL, 68.7 mmol, 3.0 eq.), CuI (87 mg, 0.46 mmol, 2 mol%) and Grubbs Catalyst® 2nd generation (194 mg, 0.23 mmol, 1 mol%). Anhydrous degassed diethyl ether (92 mL) was added and the reaction mixture was heated at reflux overnight. It was cooled to room temperature and concentrated under reduced pressure. The residue was purified by flash chromatography on silica gel (pentane/ethyl acetate = 9/1) to afford the title compound (6.37 g, 20.5 mmol, 90%) as an orange oil.

$[\boldsymbol{\alpha}]_{\boldsymbol{D}}^{\mathbf{25}}$ –2.4 (c = 1.1, $CHCl_3$); **R_f** 0.26 (pentane/ethyl acetate = 4/1); **IR** (thin film): ν_{max}/cm^{-1} 3460, 2950, 2361, 1704, 1654, 1436, 1284, 1179, 1009; **^{1}H NMR** (400 MHz, $CDCl_3$): δ 6.85 (dd, *J* = 15.8, 7.6 Hz, 1H, *H3*), 6.29 (app t, *J* = 1.0 Hz, 1H, *H7*), 5.83 (dd, *J* = 15.8, 1.3 Hz, 1H, *H2*), 4.10 (dd, *J* = 5.6, 3.9 Hz, 1H, *H5*), 3.71 (s, 3H, OCH_3), 2.61 – 2.52 (m, 1H, *H4*), 2.22 (d, *J* = 3.9 Hz, 1H, O*H*), 1.77 (d, *J* = 1.0 Hz, 3H, CCH_3), 1.05 (d, *J* = 6.8 Hz, 3H, $CHCH_3$); **^{13}C NMR** (101 MHz, $CDCl_3$): δ 167.0, 150.4, 148.0, 121.2, 80.0, 79.1, 51.7, 40.1, 20.7, 13.9; **HRMS** not found.

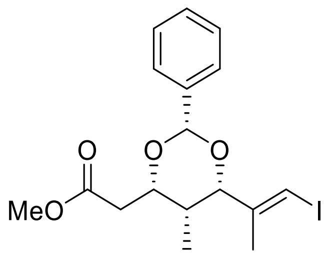

Methyl 2-((4*R*,6*S*)-6-((*E*)-1-iodoprop-1-en-2-yl)-2-phenyl-1,3-dioxan-4-yl)acetate (10). To a solution of ester **9** (3.07 g, 9.9 mmol, 1.0 eq.) in anhydrous THF (49.5 mL) at –20 °C under argon was added dropwise distilled benzaldehyde (4.4 mL, 43.6 mmol, 4.4 eq.) and KHMDS (1.0 M in THF, 5.9 mL, 5.9 mmol, 0.6 eq.) over 1.5 hours (addition was carried out in 4 portions, each consisting of 1.1 eq. of benzaldehyde, followed by 0.15 eq. of KHMDS; each portion was added over approximately 15 minutes, with a 10-minute interval between addition of each portion). The reaction mixture was stirred at –20 °C for 2 hours. It was quenched with pH 7 phosphate buffer and extracted with diethyl ether. The organic layers were washed with brine, dried over Na_2SO_4 and concentrated under reduced pressure. The residue was purified by flash chromatography on silica gel (pentane/ethyl acetate = 19/1 → pentane/ethyl acetate = 9/1) to afford the title compound (1.37 g, 3.3 mmol, 33%) as a light yellow oil. Ester **9** (190.1 mg, 0.61 mmol, 6%) was also recovered.[b]

$[\boldsymbol{\alpha}]_D^{25}$ –25.7 (c = 2.0, $CHCl_3$); **R$_f$** 0.38 (pentane/ethyl acetate = 17/3); **IR** (thin film): ν_{max}/cm^{-1} 2950, 2360, 2341, 1739, 1437, 1349, 1176, 1132, 1031, 699; **^{1}H NMR** (400 MHz, $CDCl_3$): δ 7.49 (dd, *J* = 7.5, 2.0 Hz, 2H, Ar*H*), 7.38 – 7.35 (m, 3H, Ar*H*), 6.35 – 6.34 (m, 1H, *H7*), 5.63 (s, 1H, C*H*Ph), 4.47 (ddd, *J* = 7.8, 5.9, 2.3 Hz, 1H, *H3*), 4.42 (brs, 1H, *H5*), 3.71 (s, 3H, OCH_3), 2.73 (dd, *J* = 16.0, 7.8 Hz, 1H, *H1*), 2.52 (dd, *J* = 16.0, 5.9 Hz, 1H, *H1'*), 1.89 (qdd, *J* = 7.0, 2.3, 2.3 Hz, 1H, *H4*), 1.80 (s, 3H, CCH_3), 0.87 (d, *J* = 7.0 Hz, 3H, CHCH_3); **^{13}C NMR** (101 MHz, $CDCl_3$): δ 171.4, 143.9, 138.2, 129.1, 128.4, 126.4, 101.5, 83.6, 78.4, 76.8, 52.0, 37.9, 33.2, 21.5, 6.3; **HRMS** (ESI) m/z calcd for $C_{17}H_{21}IO_4$ $[M+Na]^+$: 439.0377, found 439.0374.

[b]The reaction does not go to completion even with extended reaction times, likely due to a retro Michael reaction of the product. Only a small amount of the original starting material was recovered, as most became converted to the *Z* alkene.

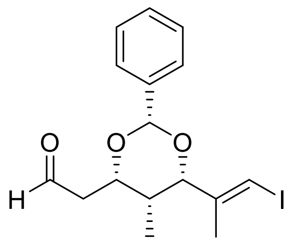

2-((4*S*,5*R*,6*S*)-6-((*E*)-1-iodoprop-1-en-2-yl)-5-methyl-2-phenyl-1,3-dioxan-4-yl)acetaldehyde (10.1). To a solution of ester **10** (1.77 g, 4.25 mmol, 1.0 eq.) in anhydrous CH_2Cl_2 (42.5 mL) under argon at –78 °C was added DIBALH (1.0 M in hexanes, 4.46 mL, 4.46 mmol, 1.05 eq.) dropwise. The reaction mixture was stirred at –78 °C for 15 minutes. It was quenched with ethyl acetate at –78 °C, followed by water (560 µL), 15% aqueous NaOH (560 µL) and water (1.2 mL). The mixture was stirred at room temperature for 15 minutes, dried over $MgSO_4$, filtered through celite and concentrated under reduced pressure. The residue was purified by flash chromatography on silica gel (pentane/ethyl acetate = 19/1 → pentane/ethyl acetate = 9/1) to afford the title compound (1.42 g, 3.68 mmol, 87%) as a colourless oil.

$[\boldsymbol{\alpha}]_{\boldsymbol{D}}^{\mathbf{25}}$ –43.4 (c = 1.0, $CHCl_3$); **R$_f$** 0.31 (pentane/ethyl acetate = 4/1); **IR** (thin film): ν_{max}/cm^{-1} 2975, 2729, 1726, 1385, 1347, 1133, 1107, 1028, 1008, 762, 700; **^{1}H NMR** (400 MHz, $CDCl_3$): δ 9.83 (t, *J* = 1.6 Hz, 1H, C*H*O), 7.49 (dd, *J* = 7.6, 2.1 Hz, 2H, Ar*H*), 7.40 – 7.36 (m, 3H, Ar*H*), 6.37 – 6.36 (m, 1H, *H7*), 5.65 (s, 1H, C*H*Ph), 4.56 (ddd, *J* = 8.4, 4.7, 2.3 Hz, 1H, *H3*), 4.44 (brs, 1H, *H5*), 2.87 (ddd, *J* = 17.2, 8.4, 1.6 Hz, 1H, *H2*), 2.54 (ddq, *J* = 17.2, 4.7, 1.6 Hz, 1H, *H2'*), 1.87 (qdd, *J* = 6.9, 2.3, 2.3 Hz, 1H, *H4*), 1.81 (s, 3H, CC*H*$_3$), 0.88 (d, *J* = 6.9 Hz, 3H, CHC*H*$_3$); **^{13}C NMR** (101 MHz, $CDCl_3$): δ 200.1, 143.7, 138.1, 129.2, 128.4, 126.3, 101.6, 83.6, 78.5, 75.4, 46.9, 33.5, 21.5, 6.5; **HRMS** (ESI) m/z calcd for $C_{16}H_{19}IO_3$ $[M+Na]^+$: 409.0271, found 409.0272.

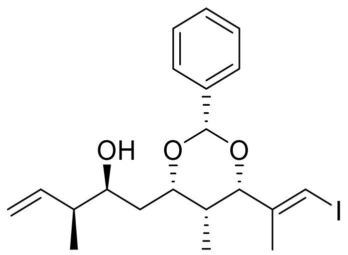

(2S,3S)-1-((4S,5R,6S)-6-((E)-1-iodoprop-1-en-2-yl)-5-methyl-2-phenyl-1,3-dioxan-4-yl)-3-methylpent-4-en-2-ol (11). Synthesized according to a modified literature procedure.[6] To a solution of (*S*,*S*)-**6'** (1.18 g, 4.05 mmol, 1.1 eq.) in anhydrous CH_2Cl_2 (14.7 mL) at 0 °C under argon was added DBU (1.8 mL, 12.14 mmol, 3.3 eq.), followed by slow addition of *cis*-crotyltrichlorosilane **7** (837.0 mg, 4.42 mmol, 1.2 eq.). The mixture was stirred at room temperature for 1 hour, after which it was recooled to 0 °C. Aldehyde **10.1** (1.42 g, 3.68 mmol, 1.0 eq.) was added dropwise and the reaction mixture was stirred at 0 °C for 1 hour. The mixture was treated with TBAF (1.0 M in THF, 4.8 mL, 4.8 mmol, 1.3 eq.) at 0 °C, stirred at room temperature for 1 hour and concentrated under reduced pressure. The residue was suspended in diethyl ether (22 mL) and the mixture was stirred vigorously for 20 minutes. The resulting precipitated DBU.HCl salts were removed by filtration. The filtrate was treated with 1 M HCl (20.2 mL, 20.2 mmol, 5.5 eq.) and the mixture was extracted with diethyl ether (3 × 20 mL). The organic layers were washed with water (2 × 20 mL) and saturated aqueous $NaHCO_3$ (20 mL), dried over Na_2SO_4 and concentrated under reduced pressure. The residue was purified by flash chromatography on silica gel (pentane/ethyl acetate = 19/1) to afford the title compound (1.14 g, 2.58 mmol, 70%, 10:1 *dr*) as a colourless oil.

$[\alpha]_D^{25}$ –60.0 (c = 0.6, $CHCl_3$); **R$_f$** 0.39 (pentane/ethyl acetate = 7/3); **IR** (thin film): ν_{max}/cm^{-1} 3415, 2971, 1408, 1384, 1347, 1107, 1029, 915, 756, 699; **^{1}H NMR** (400 MHz, $CDCl_3$): δ 7.50 (dd, *J* = 7.8, 1.9 Hz, 2H, Ar*H*), 7.41 – 7.35 (m, 3H, Ar*H*), 6.33 – 6.32 (m, 1H, *H10*), 5.80 (ddd, *J* = 17.5, 10.0, 7.3 Hz, 1H, *H2*), 5.61 (s, 1H, C*H*Ph), 5.14 – 5.08 (m, 2H, *H1* and *H1'*), 4.40 (brs, 1H, *H8*), 4.26 (td, *J* = 10.1, 2.3 Hz, 1H, *H6*), 3.82 (ddd, *J* = 10.0, 5.1, 2.2 Hz, 1H, *H4*), 2.35 – 2.27 (m, 1H, *H3*), 1.85 (ddd, *J* = 14.4, 10.1, 2.2 Hz, 1H, *H5*), 1.78 (s, 3H, CCH_3), 1.77 – 1.72 (m, 1H, *H7*), 1.62 (brs, 1H, O*H*), 1.42 (ddd, *J* = 14.4, 10.0, 2.3 Hz, 1H, *H5'*), 1.05 (d, *J* = 6.9 Hz, 3H, $CHCH_3$), 0.85 (d, *J* = 6.9 Hz, 3H, $CHCH_3$); **^{13}C NMR** (101 MHz, $CDCl_3$): δ 144.1, 140.8, 138.6, 128.9, 128.3, 126.3, 115.8, 101.4, 83.9, 78.1, 77.3, 70.9, 43.8, 37.5, 34.6, 21.5, 14.2, 6.5; **HRMS** (ESI) m/z calcd for $C_{20}H_{27}IO_3$ $[M+Na]^+$: 465.0897, found 465.0895.

Recovery of (*S*,*S*)-6' ligand

The combined aqueous acid and water layers were treated with 1 M NaOH (40.5 mL, 40.5 mmol, 10 eq. with respect to the ligand) and extracted with CH_2Cl_2 (5 × 20 mL). The organic layers were washed with water (2 × 20 mL), dried over Na_2SO_4 and concentrated under reduced pressure. The resulting solid was recrystallized from minimal boiling hexanes to afford the recovered (*S*,*S*)-**6'** ligand (558.3 mg, 1.92 mmol, 47%) as white crystals.

Mosher ester analysis of alcohol 11. Alcohol **11** was derivatized to the (*R*)-MTPA and (*S*)-MTPA esters using (*S*)-(+)-MTPA-Cl and (*R*)-(–)-MTPA-Cl respectively according to a modified literature procedure.[7]

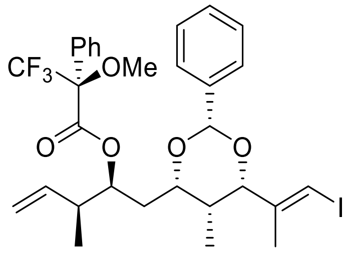

(*S*)-MTPA ester of 11. To a solution of alcohol **11** (15.0 mg, 0.034 mmol, 1.0 eq.) in CH_2Cl_2 (680 µL) was added pyridine (16.7 mg, 0.211 mmol, 6.2 eq.) and (*R*)-(–)-α-methoxy-α-(trifluoromethyl)phenylacetyl chloride (32.6 mg, 0.129 mmol, 3.8 eq.). The reaction mixture was stirred at room temperature overnight. Water was added and the mixture was extracted with CH_2Cl_2. The organic layers were dried over Na_2SO_4 and concentrated under reduced pressure. The residue was purified by flash chromatography on silica gel (pentane/ethyl acetate = 19/1) to afford the title compound (21.6 mg, 0.033 mmol, 96%) as a colourless oil.

$[\boldsymbol{\alpha}]_{\boldsymbol{D}}^{\mathbf{25}}$ –82.1 (c = 2.2, $CHCl_3$); **R_f** 0.55 (pentane/ethyl acetate = 9/1); **IR** (thin film): ν_{max}/cm^{-1} 2976, 1745, 1259, 1169, 1028, 996, 698; **^{1}H NMR** (400 MHz, $CDCl_3$): δ 7.63 – 7.61 (m, 2H, Ar*H*), 7.51 (dd, *J* = 7.9, 1.8 Hz, 2H, Ar*H*), 7.42 – 7.36 (m, 6H, Ar*H*), 6.33 (s, 1H, *H10*), 5.81 (ddd, *J* = 17.1, 10.5, 6.6 Hz, 1H, *H2*), 5.40 (ddd, *J* = 10.5, 4.3, 2.3 Hz, 1H, *H4*), 5.34 (s, 1H, C*H*Ph), 5.11 – 5.04 (m, 2H, *H1* and *H1'*), 4.08 (s, 1H, *H8*), 3.60 – 3.56 (m, 1H, *H6*), 3.59 (s, 3H, OCH_3), 2.64 – 2.59 (m, 1H, *H3*), 1.87 (ddd, *J* = 14.8, 10.6, 2.3 Hz, 1H, *H5*), 1.73 (s, 3H, CCH_3), 1.57 (ddd, *J* = 14.8, 10.5, 2.0 Hz, 1H, *H5'*), 1.51 (qdd, *J* = 6.9, 2.3, 2.3 Hz, 1H, *H7*), 1.06 (d, *J* = 6.9 Hz, 3H, $CHCH_3$), 0.76 (d, *J* = 6.8 Hz, 3H, $CHCH_3$); **^{13}C NMR** (126 MHz, $CDCl_3$): δ 166.4, 143.9, 138.9, 138.3, 132.5, 129.8, 128.9, 128.6, 128.4, 127.5, 126.1, 123.6 (q, *J* = 290 Hz), 116.2, 100.9, 84.5 (q, *J* = 27 Hz), 83.7, 78.1, 76.8, 76.1, 55.8, 41.1, 34.5, 34.2, 21.5, 14.6, 6.3; **^{19}F NMR** (376 MHz, $CDCl_3$) δ –70.6; **HRMS** (ESI) m/z calcd for $C_{30}H_{34}F_3IO_5$ $[M+Na]^+$: 681.1295, found 681.1291.

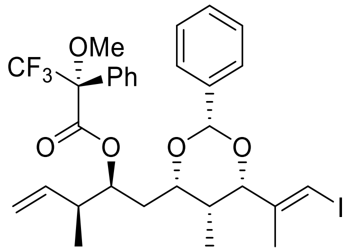

(*R*)-MTPA ester of 11. Synthesized in the same manner as the (*S*)-MTPA ester of **11** using 0.034 mmol of alcohol **11** and substituting (*R*)-(–)-α-methoxy-α-(trifluoromethyl)phenylacetyl chloride with (*S*)-(+)-α-methoxy-α-(trifluoromethyl)phenylacetyl chloride. The title compound (19.4 mg, 0.029 mmol, 87%) was obtained as a colourless oil.

$[\boldsymbol{\alpha}]_{\boldsymbol{D}}^{\mathbf{25}}$ –38.6 (c = 1.9, $CHCl_3$); **R$_f$** 0.55 (pentane/ethyl acetate = 9/1); **IR** (thin film): ν_{max}/cm^{-1} 2975, 1746, 1255, 1168, 1027, 997, 698; **^{1}H NMR** (400 MHz, $CDCl_3$): δ 7.62 – 7.60 (m, 2H, Ar*H*), 7.50 (dd, *J* = 7.9, 1.8 Hz, 2H, Ar*H*), 7.42 – 7.36 (m, 6H, Ar*H*), 6.35 (s, 1H, *H10*), 5.73 (ddd, *J* = 17.3, 10.5, 6.9 Hz, 1H, *H2*), 5.40 (ddd, *J* = 10.3, 4.7, 2.6 Hz, 1H, *H4*), 5.37 (s, 1H, C*H*Ph), 5.06 – 5.00 (m, 2H, *H1* and *H1'*), 4.15 (s, 1H, *H8*), 3.71 (td, *J* = 10.5, 2.2 Hz, 1H, H6), 3.53 (s, 3H, OCH_3), 2.61 – 2.53 (m, 1H, *H3*), 1.93 (ddd, *J* = 14.8, 10.5, 2.7 Hz, 1H, *H5*), 1.77 (s, 3H, CCH_3), 1.66 – 1.61 (m, 1H, *H5'*), 1.61 – 1.57 (m, 1H, *H7*), 1.04 (d, *J* = 6.9 Hz, 3H, CHCH_3), 0.80 (d, *J* = 6.9 Hz, 3H, CHCH_3); **^{13}C NMR** (101 MHz, $CDCl_3$): δ 166.2, 143.9, 138.9, 138.3, 132.3, 129.8, 128.9, 128.6, 128.3, 127.8, 126.2, 123.6 (q, *J* = 290 Hz), 116.1, 101.1, 84.6 (q, *J* = 28 Hz), 83.7, 78.2, 76.8, 76.4, 55.5, 41.2, 34.9, 34.3, 21.5, 14.5, 6.3; **^{19}F NMR** (376 MHz, $CDCl_3$) δ –70.5; **HRMS** (ESI) m/z calcd for $C_{30}H_{34}F_3IO_5$ $[M+Na]^+$: 681.1295, found 681.1289.

Comparison of the ^{1}H NMR data of the two Mosher ester derivatives is shown below.

**Assignment**	**δ, (*S*)-ester (ppm)**	**δ, (*R*)-ester (ppm)**	**$\Delta\delta^{SR}$**
**H1/H1'**	5.08	5.03	+0.05
**H2**	5.81	5.73	+0.08
**H3**	2.62	2.57	+0.05
**H5**	1.87	1.93	–0.06
**H5'**	1.57	1.63	–0.06
**H6**	3.58	3.71	–0.13
**H7**	1.51	1.59	–0.08
**H8**	4.08	4.15	–0.07
**H10**	6.33	6.35	–0.02

Analysis indicates that the C4 centre is of *R* configuration.

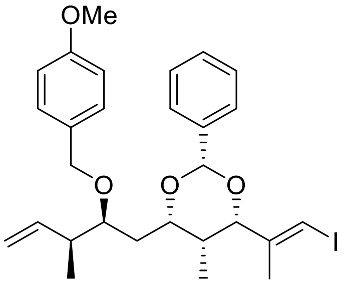

(4*S*,5*R*,6*S*)-4-((*E*)-1-iodoprop-1-en-2-yl)-6-((2*S*,3*S*)-2-((4-methoxybenzyl)oxy)-3-methylpent-4-en-1-yl)-5-methyl-2-phenyl-1,3-dioxane (11.1). To a solution of alcohol **11** (940 mg, 2.13 mmol, 1.0 eq.) in anhydrous DMF (4.3 mL) under argon at 0 °C was added NaH (60% in mineral oil, 170.4 mg, 4.26 mmol, 2.0 eq.). The mixture was stirred for 30 minutes, after which PMBCl (578 µL, 4.26 mmol, 2.0 eq.) and TBAI (158.8 mg, 0.43 mmol, 0.2 eq.) were added. The reaction mixture was stirred at room temperature for 24 hours. It was quenched slowly with saturated aqueous NH_4Cl and extracted with diethyl ether. The organic layers were washed with brine, dried over Na_2SO_4 and concentrated under reduced pressure. The residue was purified by flash chromatography on silica gel (pentane/diethyl ether = 19/1) to afford the title compound (1.08 g, 1.92 mmol, 90%) as a colourless oil.

$[\boldsymbol{\alpha}]_{\boldsymbol{D}}^{\mathbf{25}}$ –83.1 (c = 1.1, $CHCl_3$); **R**$_\mathbf{f}$ 0.32 (pentane/diethyl ether = 9/1); **IR** (thin film): ν_{max}/cm^{-1} 2930, 2885, 2361, 2341, 1612, 1513, 1457, 1348, 1248, 1059, 1035, 699; **^{1}H NMR** (400 MHz, $CDCl_3$): δ 7.44 (dd, *J* = 7.7, 2.0 Hz, 2H, Ar*H*), 7.40 – 7.35 (m, 3H, Ar*H*), 7.28 (d, *J* = 8.7 Hz, 2H, Ar*H*), 6.84 (d, *J* = 8.7 Hz, 2H, Ar*H*), 6.30 – 6.29 (m, 1H, *H10*), 5.92 (ddd, *J* = 17.7, 10.0, 6.9 Hz, 1H, *H2*), 5.36 (s, 1H, C*H*Ph), 5.10 – 5.05 (m, 2H, *H1* and *H1'*), 4.64 (d, *J* = 11.2 Hz, 1H, OCH_2Ar), 4.40 (d, *J* = 11.2 Hz, 1H, OCH'_2Ar), 4.29 (brs, 1H, *H8*), 4.06 (td, *J* = 10.3, 2.2 Hz, 1H, *H6*), 3.73 (s, 3H, OCH_3), 3.63 (ddd, *J* = 10.7, 4.7, 2.2 Hz, 1H, *H4*), 2.60 – 2.55 (m, 1H, *H3*), 1.77 – 1.71 (m, 1H, *H5*), 1.74 (s, 3H, CCH_3), 1.67 (qdd, *J* = 6.9, 2.3, 2.3 Hz, 1H, *H7*), 1.39 (ddd, *J* = 14.5, 10.7, 2.1 Hz, 1H, *H5'*), 1.05 (d, *J* = 6.9 Hz, 3H, CHCH_3), 0.79 (d, *J* = 7.0 Hz, 3H, CHCH_3); **^{13}C NMR** (101 MHz, $CDCl_3$): δ 159.4, 144.2, 140.4, 138.8, 131.0, 129.8, 128.8, 128.2, 126.2, 114.9, 114.0, 101.1, 84.0, 78.3, 77.9, 76.9, 72.1, 55.3, 40.5, 35.2, 34.4, 21.5, 15.2, 6.5; **HRMS** (ESI) m/z calcd for $C_{28}H_{35}IO_4$ $[M+Na]^+$: 585.1496, found 585.1469.

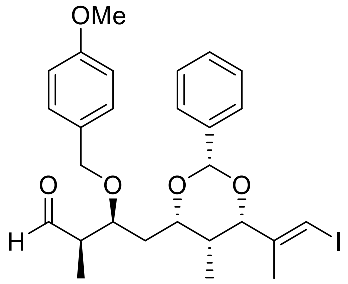

(2*R*,3*S*)-4-((4*S*,5*R*,6*S*)-6-((*E*)-1-iodoprop-1-en-2-yl)-5-methyl-2-phenyl-1,3-dioxan-4-yl)-3-((4-methoxybenzyl)oxy)-2-methylbutanal (12). To a solution of alkene **11.1** (900 mg, 1.60 mmol, 1.0 eq.) in acetone (16.0 mL) and water (5.3 mL) at 10 °C was added 4-methylmorpholine *N*-oxide (NMO) monohydrate (432.5 mg, 3.20 mmol, 2.0 eq.) and potassium osmate dihydrate (14.7 mg, 0.040 mmol, 2.5 mol%). The reaction mixture was stirred at 10 °C overnight. Saturated aqueous $Na_2S_2O_3$ (18 mL) was added and the mixture was stirred vigorously for 2 hours. It was diluted with ethyl acetate, washed with saturated aqueous $NaHCO_3$, and the aqueous layer was further extracted with ethyl acetate. The organic layers were washed with brine, dried over Na_2SO_4 and concentrated under reduced pressure. The residue was purified by flash chromatography on silica gel (pentane/ethyl acetate = 7/3 → pentane/ethyl acetate = 3/2) to afford a 3:1 diastereomeric mixture of diols (620.0 mg, 1.10 mmol). The mixture of diols was dissolved in CH_2Cl_2 (11 mL) and $NaIO_4$ (14 wt% on silica,[8] 5.04 g, 3.30 mmol, 3.0 eq.) was added. The reaction mixture was stirred at room temperature for 1 hour. It was filtered and concentrated under reduced pressure. The residue was purified by flash chromatography on silica gel (pentane/ethyl acetate = 17/3) to afford the title compound (595.6 mg, 1.06 mmol, 62% over two steps) as a colourless oil.

$[\alpha]_D^{25}$ –94.4 (c = 0.5, $CHCl_3$); **R$_f$** 0.31 (pentane/ethyl acetate = 4/1); **IR** (thin film): ν_{max}/cm^{-1} 2939, 2914, 2881, 2361, 1721, 1612, 1513, 1454, 1347, 1249, 1056, 1033, 700; **^{1}H NMR** (400 MHz, $CDCl_3$): δ 9.84 (d, *J* = 1.0 Hz, 1H, C*H*O), 7.45 (dd, *J* = 7.8, 2.0 Hz, 2H, Ar*H*), 7.41 – 7.35 (m, 3H, Ar*H*), 7.25 (d, *J* = 8.6 Hz, 2H, Ar*H*), 6.86 (d, *J* = 8.6 Hz, 2H, Ar*H*), 6.32 – 6.31 (m, 1H, *H9*), 5.39 (s, 1H, C*H*Ph), 4.57 (d, *J* = 11.2 Hz, 1H, OC*H*$_2$Ar), 4.39 (d, *J* = 11.2 Hz, 1H, OC*H'*$_2$Ar), 4.31 (brs, 1H, *H7*), 4.16 – 4.12 (m, 1H, *H3*), 4.07 (td, *J* = 10.3, 2.2 Hz, 1H, *H5*), 3.74 (s, 3H, OC*H*$_3$), 2.65 (qdd, *J* = 7.0, 3.4, 1.0 Hz, 1H, *H2*), 1.83 – 1.76 (m, 1H, *H4*), 1.76 (s, 3H, CC*H*$_3$), 1.72 – 1.67 (m, 1H, *H6*), 1.58 (ddd, *J* = 14.3, 10.3, 2.1 Hz, 1H, *H4'*), 1.13 (d, *J* = 7.1 Hz, 3H, CHC*H*$_3$), 0.81 (d, *J* = 6.8 Hz, 3H, CHC*H*$_3$); **^{13}C NMR** (101 MHz, $CDCl_3$): δ 204.6, 159.6, 144.0, 138.5, 130.2, 129.9, 129.0, 128.3, 126.2, 114.1, 101.2, 83.9, 78.1, 76.7, 74.6,

72.3, 55.4, 50.4, 36.6, 34.3, 21.5, 8.6, 6.5; **HRMS** (ESI) m/z calcd for $C_{27}H_{33}IO_5$ $[M+Na]^+$: 587.1265, found 587.1264.

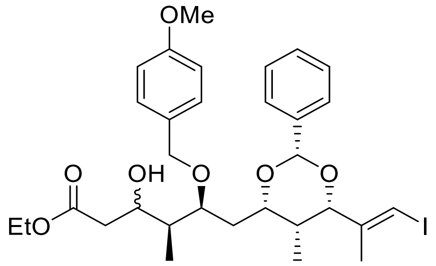

Ethyl (4*S*,5*S*)-3-hydroxy-6-((4*S*,5*R*,6*S*)-6-((*E*)-1-iodoprop-1-en-2-yl)-5-methyl-2-phenyl-1,3-dioxan-4-yl)-5-((4-methoxybenzyl)oxy)-4-methylhexanoate (12.1). To a solution of aldehyde **12** (416.5 mg, 0.74 mmol, 1.0 eq.) and (1-ethoxyvinyl)oxy-(trimethyl)silane[9] (**13**, 354.9 mg, 2.21 mmol, 3.0 eq.) in anhydrous CH_2Cl_2 (14.8 mL) at –78 °C under argon was added dropwise $BF_3{\cdot}OEt_2$ (91 µL, 0.74 mmol, 1.0 eq.) over 5 minutes. The reaction mixture was stirred at –78 °C for 1 hour, after which it was quenched with pH 7 buffer and extracted with CH_2Cl_2. The organic layers were washed with brine, dried over Na_2SO_4 and concentrated under reduced pressure. The residue was purified by flash chromatography on silica gel (pentane/ethyl acetate = 17/3) to afford the title compound (409.4 mg, 0.63 mmol, 85%)[c] as a colourless oil.

$[\boldsymbol{\alpha}]_{\boldsymbol{D}}^{\mathbf{25}}$ –49.1 (c = 1.1, $CHCl_3$); $\mathbf{R_f}$ 0.34 (pentane/ethyl acetate = 7/3); **IR** (thin film): ν_{max}/cm^{-1} 3498, 2976, 2936, 2908, 1612, 1728, 1513, 1456, 1348, 1249, 1176, 1033, 700; **^{1}H NMR** (400 MHz, $CDCl_3$): δ 7.46 (dd, *J* = 7.7, 2.0 Hz, 2H, Ar*H*), 7.40 – 7.35 (m, 3H, Ar*H*), 7.27 (d, *J* = 8.7 Hz, 2H, Ar*H*), 6.86 (d, *J* = 8.7 Hz, 2H, Ar*H*), 6.33 – 6.32 (m, 1H, *H11*), 5.42 (s, 1H, C*H*Ph), 4.55 (d, *J* = 11.2 Hz, 1H, OCH_2Ar), 4.46 (d, *J* = 11.2 Hz, 1H, OCH'_2Ar), 4.32 (brs, 1H, *H9*), 4.26 – 4.21 (m, 1H, *H3*), 4.15 (q, *J* = 7.2 Hz, 2H, $CO_2CH_2CH_3$), 4.02 (td, *J* = 9.7, 2.5 Hz, 1H, *H7*), 3.80 – 3.76 (m, 1H, *H5*), 3.75 (s, 3H, OCH_3), 3.11 (d, *J* = 3.2 Hz, 1H, O*H*), 2.56 (dd, *J* = 16.2, 8.7 Hz, 2H, *H2*), 2.49 (dd, *J* = 16.2, 4.2 Hz, 2H, *H2'*), 1.92 – 1.82 (m, 2H, *H4* and *H6*), 1.78 (s, 3H, CCH_3), 1.74 – 1.62 (m, 2H, *H6'* and *H8*), 1.26 (t, *J* = 7.2 Hz, 4H, $CO_2CH_2CH_3$), 1.00 (d, *J* = 7.1 Hz, 3H, CHCH_3), 0.84 (d, *J* = 7.0 Hz, 3H, CHCH_3); **^{13}C NMR** (101 MHz, $CDCl_3$): δ 173.0, 159.4, 144.1, 138.5, 130.4, 129.7, 128.9, 128.3, 126.2, 114.0, 101.1, 83.9,

[c]Obtained as a single diastereomer

78.7, 78.1, 77.3, 71.9, 69.4, 60.7, 55.3, 40.3, 40.2, 35.2, 34.3, 21.5, 14.3, 9.2, 6.5; **HRMS** (ESI) m/z calcd for $C_{31}H_{41}IO_7$ $[M+Na]^+$: 675.1789, found 675.1779.

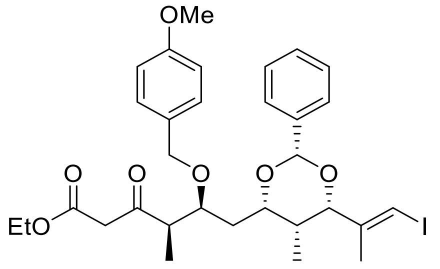

Ethyl (4*R*,5*S*)-6-((4*S*,5*R*,6*S*)-6-((*E*)-1-iodoprop-1-en-2-yl)-5-methyl-2-phenyl-1,3-dioxan-4-yl)-5-((4-methoxybenzyl)oxy)-4-methyl-3-oxohexanoate (14). To a solution of β-hydroxy ester **12.1** (521.8 mg, 0.80 mmol, 1.0 eq.) in CH_2Cl_2 (16 mL) was added $NaHCO_3$ (336.0 mg, 4.00 mmol, 5.0 eq.). The mixture was cooled to 0 °C and Dess-Martin periodinane (509.0 mg, 1.20 mmol, 1.5 eq.) was added. The reaction mixture was stirred at room temperature for 30 minutes. It was diluted with diethyl ether, quenched with saturated aqueous $Na_2S_2O_3$ (9 mL) and extracted with diethyl ether. The organic layers were washed with saturated aqueous $NaHCO_3$, water and brine. It was dried over Na_2SO_4 and concentrated under reduced pressure. The residue was purified by flash chromatography on silica gel (pentane/ethyl acetate = 17/3) to afford the title compound (452.1 mg, 0.69 mmol, 87%) as a colourless oil.

$[\alpha]_D^{25}$ –113.8 (c = 0.6, $CHCl_3$); **R_f** 0.46 (pentane/ethyl acetate = 3/1); **IR** (thin film): ν_{max}/cm^{-1} 2980, 2936, 2909, 1743, 1710, 1513, 1457, 1304, 1249, 1057, 1030, 700; **^{1}H NMR** (400 MHz, $CDCl_3$): δ 7.43 – 7.40 (m, 2H, Ar*H*), 7.39 – 7.35 (m, 3H, Ar*H*), 7.28 (d, *J* = 8.6 Hz, 2H, Ar*H*), 6.85 (d, *J* = 8.6 Hz, 2H, Ar*H*), 6.30 – 6.29 (m, 1H, *H11*), 5.31 (s, 1H, C*H*Ph), 4.66 (d, *J* = 11.3 Hz, 1H, OCH_2Ar), 4.42 (d, *J* = 11.3 Hz, 1H, OCH'_2Ar), 4.28 (brs, 1H, *H9*), 4.18 (qd, *J* = 7.2, 1.0 Hz, 2H, $CO_2CH_2CH_3$), 3.99 (dt, *J* = 10.5, 2.0 Hz, 1H, *H7*), 3.89 (ddd, *J* = 10.5, 3.8, 2.5 Hz, 1H, *H5*), 3.73 (s, 3H, OCH_3), 3.60 (d, *J* = 1.2 Hz, 2H, *H2*), 3.14 (qd, *J* = 7.1, 3.8 Hz, 1H, *H4*), 1.81 – 1.74 (m, 1H, *H6*), 1.76 (s, 3H, CCH_3), 1.70 –1.64 (m, 1H, *H8*), 1.37 (ddd, *J* = 14.3, 10.5, 2.0 Hz, 1H, *H6'*), 1.27 (t, *J* = 7.1 Hz, 3H, $CO_2CH_2CH_3$), 1.10 (d, *J* = 7.0 Hz, 3H, CHCH_3), 0.79 (d, *J* = 6.9 Hz, 3H, CHCH_3); **^{13}C NMR** (101 MHz, $CDCl_3$): δ 205.4, 167.6, 159.6, 144.1, 138.6, 130.1, 130.0, 128.9, 128.3, 126.2, 126.2, 114.1, 101.1, 83.9, 78.1, 76.5, 72.2, 61.4, 55.3, 50.2, 48.7, 35.3, 34.3, 21.5, 14.3, 12.0, 6.4; **HRMS** (ESI) m/z calcd for $C_{31}H_{39}IO_7$ $[M+Na]^+$: 673.1635, found 673.1630.

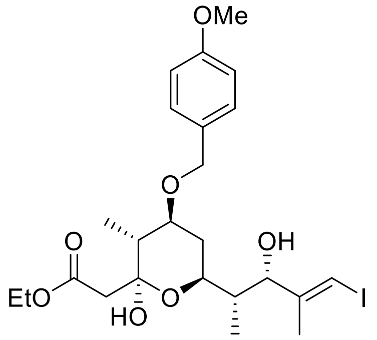

Ethyl 2-((2*S*,3*R*,4*S*,6*S*)-2-hydroxy-6-((2*S*,3*S*,*E*)-3-hydroxy-5-iodo-4-methylpent-4-en-2-yl)-4-((4-methoxybenzyl)oxy)-3-methyltetrahydro-2*H*-pyran-2-yl)acetate (15). To a solution of β-keto ester **14** (106.7 mg, 0.164 mmol, 1.0 eq.) in THF (4.1 mL) and MeOH (4.1 mL) was added 1.0 M aqueous HCl (4.1 mL). The reaction mixture was stirred at 35 °C for 22 hours. It was diluted with CH_2Cl_2, quenched with saturated aqueous $NaHCO_3$ and extracted with CH_2Cl_2. The organic layers were dried over Na_2SO_4 and concentrated under reduced pressure. The residue was purified by flash chromatography on silica gel (pentane/diethyl ether = 9/1 → pentane/diethyl ether = 4/1) to afford the title compound (43.4 mg, 0.077 mmol, 47%)[d] as a colourless oil; β-keto ester **14** (49.0 mg, 0.075 mmol, 46%) was also recovered.

$[\alpha]_D^{25}$ +29.2 (c = 0.4, $CHCl_3$); **R_f** 0.39 (pentane/diethyl ether = 1/1); **IR** (thin film): ν_{max}/cm^{-1} 3477, 2979, 2949, 2920, 2358, 1710, 1514, 1459, 1337, 1248, 1186, 1082, 1034, 821; **^{1}H NMR** (400 MHz, $CDCl_3$): δ 7.26 (d, *J* = 8.6 Hz, 2H, Ar*H*), 6.88 (d, *J* = 8.6 Hz, 2H, Ar*H*), 6.32 – 6.31 (m, 1H, *H11*), 4.82 (d, *J* = 1.9 Hz, 1H, O*H*), 4.57 (d, *J* = 11.0 Hz, 1H, OC*H*2Ar), 4.39 (d, *J* = 11.0 Hz, 1H, OC*H'*2Ar), 4.26 (d, *J* = 2.3 Hz, 1H, *H9*), 4.20 (qd, *J* = 7.1, 3.4 Hz, 2H, $CO_2CH_2CH_3$), 4.11 (dt, *J* = 12.2, 2.2 Hz, 1H, *H7*), 3.80 (s, 3H, OC*H*3), 3.56 (td, *J* = 10.6, 4.5 Hz, 1H, *H5*), 2.87 (brs, 1H, O*H*), 2.81 (d, *J* = 15.5 Hz, 1H, *H2*), 2.52 (d, *J* = 15.5 Hz, 1H, *H2'*), 1.96 (ddd, *J* = 12.2, 4.5, 2.2 Hz, 1H, *H6*), 1.75 (d, *J* = 0.7 Hz, 3H, CC*H*3), 1.73 – 1.70 (m, 1H, *H8*), 1.54 – 1.44 (m, 2H, *H4* and *H6'*), 1.29 (t, *J* = 7.1 Hz, 3H, $CO_2CH_2CH_3$), 1.09 (d, *J* = 6.6 Hz, 3H, CHC*H*3), 0.84 (d, *J* = 7.0 Hz, 3H, CHC*H*3); **^{13}C NMR** (101 MHz, $CDCl_3$): δ 173.1, 159.4, 147.0, 130.8, 129.5, 114.0, 99.3, 80.0, 78.7, 76.3, 71.9, 71.0, 61.6, 55.4, 45.2, 42.6, 39.3, 35.1, 21.7, 14.2, 12.4, 6.2; **HRMS** (ESI) m/z calcd for $C_{24}H_{35}IO_7$ $[M+Na]^+$: 585.1320, found 585.2321.

[d]Obtained with an inseparable impurity

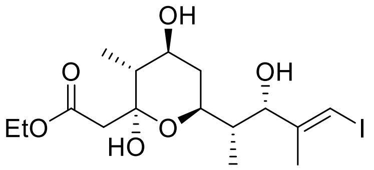

Ethyl 2-((2*S*,3*R*,4*S*,6*S*)-2,4-dihydroxy-6-((2*S*,3*S*,*E*)-3-hydroxy-5-iodo-4-methylpent-4-en-2-yl)-3-methyltetrahydro-2*H*-pyran-2-yl)acetate (2). To a solution of PMB ether **15** (36.3 mg, 0.065 mmol, 1.0 eq.) in CH_2Cl_2 (2.2 mL) and water (116 μL) at 0 °C was added DDQ (26.6 mg, 0.117 mmol, 1.8 eq.). The reaction mixture was stirred at 0 °C under argon for 1 hour. It was quenched with saturated aqueous $NaHCO_3$ and extracted with ethyl acetate. The organic layers were dried over Na_2SO_4 and concentrated under reduced vacuum. The residue was purified by flash chromatography on silica gel (pentane/ethyl acetate = 4/1) to afford the title compound (12.5 mg, 0.028 mmol, 44%) as a colourless oil.

$[\boldsymbol{\alpha}]_{\boldsymbol{D}}^{\mathbf{25}}$ +14.7 (c = 1.2, $CHCl_3$); **R_f** 0.28 (pentane/ethyl acetate = 1/1); **IR** (thin film): ν_{max}/cm^{-1} 3443, 2978, 2919, 2851, 1711, 1375, 1262, 1194, 1018, 987; **^{1}H NMR** (400 MHz, $CDCl_3$): δ 6.32 – 6.31 (m, 1H, *H11*), 4.81 (brs, 1H, O*H*), 4.26 (d, *J* = 2.8 Hz, 1H, *H9*), 4.21 (qd, *J* = 7.2, 3.1 Hz, 2H, $CO_2CH_2CH_3$), 4.15 (dt, *J* = 12.1, 2.2 Hz, 1H, *H7*), 3.81 (td, *J* = 10.8, 4.7 Hz, 1H, *H5*), 2.81 (d, *J* = 15.5 Hz, 1H, *H2*), 2.53 (d, *J* = 15.5 Hz, 1H, *H2'*), 1.81 (ddd, *J* = 12.1, 4.7, 2.2 Hz, 1H, *H6*), 1.74 (d, *J* = 0.6 Hz, 3H, CCH_3), 1.72 – 1.69 (m, 1H, *H8*), 1.53 (td, *J* = 12.1, 10.8 Hz, 2H, H6'), 1.36 – 1.33 (m, 1H, *H4*), 1.29 (t, *J* = 7.2 Hz, 3H, $CO_2CH_2CH_3$), 1.11 (d, *J* = 6.7 Hz, 3H, $CHCH_3$), 0.84 (d, *J* = 7.0 Hz, 3H, $CHCH_3$); **^{13}C NMR** (101 MHz, $CDCl_3$): δ 173.0, 147.0, 99.2, 79.9, 78.7, 71.9, 69.5, 61.6, 46.8, 42.5, 39.1, 38.5, 21.7, 14.2, 12.1, 6.2; **HRMS** (ESI) m/z calcd for $C_{16}H_{27}IO_6$ $[M+Na]^+$: 465.0749, found 465.0745.

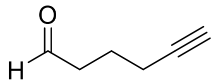

5-Hexynal (16). Synthesized according to a modified literature procedure.[10] To a solution of TEMPO (398 mg, 2.55 mmol, 0.1 eq.) and (diacetoxyiodo)benzene (9.9 g, 30.6 mmol, 1.2 eq.) in anhydrous CH_2Cl_2 (72.8 mL) under argon was added 5-hexyn-1-ol (2.8 mL, 25.5 mmol, 1.0 eq.) slowly. The reaction mixture was stirred at room temperature for 3 hours. It was quenched with saturated aqueous $Na_2S_2O_3$. The organic layer was washed with saturated aqueous $NaHCO_3$ and brine, dried over Na_2SO_4 and carefully concentrated under reduced pressure. The residue was purified by flash chromatography on silica gel (pentane/dichloromethane = 4/1 → pentane/dichloromethane = 1/4) to afford the title compound (1.82 g, 18.9 mmol, 74%) as a colourless oil. **^{1}H NMR** (400 MHz, $CDCl_3$): δ 9.81 (t, *J* = 1.3 Hz, 1H), 2.61 (td, *J* = 7.2, 1.3 Hz, 2H), 2.27 (td, *J* = 6.9, 2.7 Hz, 2H), 1.98 (t, *J* = 2.7 Hz, 1H), 1.85 (quint, *J* = 7.0 Hz, 2H); **^{13}C NMR** (101 MHz, $CDCl_3$): δ 201.8, 83.3, 69.5, 42.7, 21.0, 17.9. The physical and spectroscopic data were consistent with literature reported data.[11]

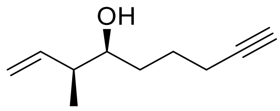

(3*S*,4*S*)-3-methylnon-1-en-8-yn-4-ol (17). Synthesized according to a modified literature procedure.[6] To a solution of (*S*,*S*)-**6'** (3.62 g, 12.47 mmol, 1.1 eq.) in anhydrous CH_2Cl_2 (45.4 mL) at 0 °C under argon was added DBU (5.6 mL, 37.42 mmol, 3.3 eq.), followed by slow addition of *cis*-crotyltrichlorosilane **7** (2.58 g, 13.61 mmol, 1.2 eq.). The mixture was stirred at room temperature for 1 hour, after which it was recooled to 0 °C. 5-Hexynal (**16**, 1.09 g, 11.34 mmol, 1.0 eq.) was added dropwise and the reaction mixture was stirred at 0 °C for 1 hour. The mixture was treated with TBAF (1.0 M in THF, 14.7 mL, 14.7 mmol, 1.3 eq.) at 0 °C, stirred at room temperature for 1 hour and carefully concentrated under reduced pressure. The residue was suspended in diethyl ether (67.5 mL) and the mixture was stirred vigorously for 20 minutes. The resulting precipitated DBU.HCl salts were removed by filtration. The filtrate was treated with 1 M HCl (62.4 mL, 62.4 mmol, 5.5 eq.) and the mixture was extracted with diethyl ether (3 × 67 mL). The organic layers were washed with water (2 × 67 mL) and saturated aqueous $NaHCO_3$ (67 mL), dried over Na_2SO_4 and carefully concentrated under reduced pressure. The residue was purified by flash chromatography on silica gel

(pentane/diethyl ether = 9/1) to afford the title compound (1.41 g, 9.26 mmol, 82%, 92% *ee*, >20:1 *dr*)[e] as a colourless oil.

$[\boldsymbol{\alpha}]_{\boldsymbol{D}}^{\mathbf{25}}$ –37.9 (c = 1.0, $CHCl_3$); **R$_f$** 0.23 (pentane/diethyl ether = 4/1); **IR** (thin film): ν_{max}/cm^{-1} 3384, 3306, 2954, 2934, 2870, 1639, 1456, 1105, 1072, 997, 954, 915, 631; **^{1}H NMR** (400 MHz, $CDCl_3$): δ 5.78 (ddd, *J* = 17.5, 9.9, 7.4 Hz, 1H, *H2*), 5.11 – 5.06 (m, 2H, *H1*), 3.51 (ddd, *J* = 9.2, 5.0, 3.1 Hz, 1H, *H4*), 2.32 – 2.25 (m, 1H, *H3*), 2.23 (td, *J* = 6.6, 2.6 Hz, 2H, *H7*), 1.95 (t, *J* = 2.6 Hz, 1H, *H9*), 1.79 – 1.41 (m, 4H, *H5* and *H6*), 1.03 (d, *J* = 6.9 Hz, 3H, CHC*H*$_3$); **^{13}C NMR** (101 MHz, $CDCl_3$): δ 140.9, 115.6, 84.5, 74.4, 68.6, 43.8, 33.1, 25.2, 18.5, 14.3; **HRMS** not found.

Recovery of (*S*,*S*)-6' ligand

The combined aqueous acid and water layers were treated with 1 M NaOH (124.7 mL, 124.7 mmol, 10 eq. with respect to the ligand) and extracted with CH_2Cl_2 (5 × 60 mL). The organic layers were washed with water (2 × 60 mL), dried over Na_2SO_4 and concentrated under reduced pressure. The resulting solid was recrystallized from minimal boiling hexanes to afford the recovered (*S*,*S*)-**6'** ligand (2.35 g, 8.09 mmol, 65%) as white crystals.

[e]The product was obtained as a single diastereomer; its *ee* was determined by ^{1}H NMR analysis of the Mosher esters (^{19}F NMR peaks were overlapping).

Mosher ester analysis of alcohol 17. Alcohol **17** was derivatized to the (*R*)-MTPA and (*S*)-MTPA esters using (*S*)-(+)-MTPA-Cl and (*R*)-(–)-MTPA-Cl respectively according to a modified literature procedure.[7]

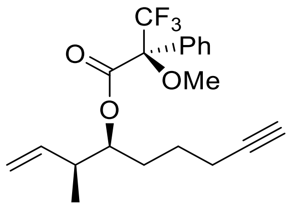

(*S*)-MTPA ester of 17. To a solution of alcohol **17** (10.0 mg, 0.066 mmol, 1.0 eq.) in CH_2Cl_2 (660 µL) was added pyridine (32.4 mg, 0.409 mmol, 6.2 eq.) and (*R*)-(–)-α-methoxy-α-(trifluoromethyl)phenylacetyl chloride (63.3 mg, 0.251 mmol, 3.8 eq.). The reaction mixture was stirred at room temperature overnight. Water was added and the mixture was extracted with CH_2Cl_2. The organic layers were dried over Na_2SO_4 and concentrated under reduced pressure. The residue was purified by flash chromatography on silica gel (pentane/diethyl ether = 49/1) to afford the title compound (22.9 mg, 0.062 mmol, 94%) as a colourless oil.

$[\boldsymbol{\alpha}]_{\boldsymbol{D}}^{\mathbf{25}}$ –51.3 (c = 2.3, $CHCl_3$); **R$_f$** 0.49 (pentane/diethyl ether = 9/1); **IR** (thin film): ν_{max}/cm^{-1} 3307, 2953, 1743, 1259, 1169, 1123, 1018, 994; **^{1}H NMR** (500 MHz, $CDCl_3$): δ 7.55 (dd, *J* = 6.9, 2.8 Hz, 2H, Ar*H*), 7.41 – 7.38 (m, 3H, Ar*H*), 5.76 (ddd, *J* = 16.9, 10.8, 7.1 Hz, 1H, *H2*), 5.09 – 5.05 (m, 3H, *H1* and *H4*), 3.54 (s, 3H, OC*H*3), 2.57 – 2.50 (m, 1H, *H3*), 2.12 (td, *J* = 6.8, 2.7 Hz, 2H, *H7*), 1.93 (t, *J* = 2.7 Hz, 1H, *H9*), 1.77 – 1.63 (m, 2H, *H5*), 1.49 – 1.33 (m, 2H, *H6*), 1.04 (d, *J* = 6.9 Hz, 3H, CHC*H*3); **^{13}C NMR** (126 MHz, $CDCl_3$): δ 166.5, 139.1, 132.3, 129.7, 128.5, 127.6, 123.5 (q, *J* = 289 Hz), 116.1, 84.8 (q, *J* = 28 Hz), 83.7, 79.7, 68.9, 55.6, 40.8, 30.0, 24.0, 18.2, 15.0; **^{19}F NMR** (470 MHz, $CDCl_3$): δ –71.1; **HRMS** (ESI) m/z calcd for $C_{20}H_{23}F_3O_3$ $[M+Na]^+$: 391.1492, found 391.1493.

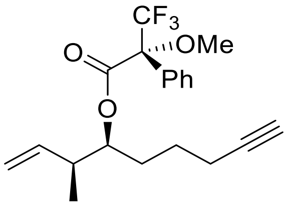

(*R*)-MTPA ester of 17. Synthesized in the same manner as the (*S*)-MTPA ester of **17** using 0.066 mmol of alcohol **17** and substituting (*R*)-(–)-α-methoxy-α-(trifluoromethyl)phenylacetyl chloride with (*S*)-(+)-α-methoxy-α-(trifluoromethyl)phenylacetyl chloride. The title compound (24.0 mg, 0.065 mmol, 99%) was obtained as a colourless oil.

$[\boldsymbol{\alpha}]_{\boldsymbol{D}}^{\mathbf{25}}$ +7.3 (c = 2.4, $CHCl_3$); **R_f** 0.49 (pentane/diethyl ether = 9/1); **IR** (thin film): ν_{max}/cm^{-1} 3307, 2952, 1744, 1259, 1169, 1123, 1018, 994; **^{1}H NMR** (500 MHz, $CDCl_3$): δ 7.56 (dd, *J* = 7.0, 3.0 Hz, 2H, Ar*H*), 7.41 – 7.39 (m, 3H, Ar*H*), 5.65 (ddd, *J* = 17.4, 10.5, 7.4 Hz, 1H, *H2*), 5.07 (ddd, *J* = 8.0, 5.7, 4.0 Hz, 1H, *H4*), 5.02 – 4.96 (m, 2H, *H1*), 3.55 (s, 3H, OCH_3), 2.51 – 2.44 (m, 1H, *H3*), 2.19 (td, *J* = 6.8, 2.7 Hz, 2H, *H7*), 1.95 (t, *J* = 2.7 Hz, 1H, *H9*), 1.82 – 1.69 (m, 2H, *H5*), 1.60 – 1.47 (m, 2H, *H6*), 0.96 (d, *J* = 6.9 Hz, 3H, CHCH_3); **^{13}C NMR** (126 MHz, $CDCl_3$): δ 166.5, 139.0, 132.4, 129.7, 128.5, 127.6, 123.5 (q, *J* = 289 Hz), 116.0, 84.6 (q, *J* = 28 Hz), 83.7, 79.7, 69.0, 55.6, 40.9, 30.3, 24.3, 18.3, 14.9; **^{19}F NMR** (470 MHz, $CDCl_3$): δ –71.1; **HRMS** (ESI) m/z calcd for $C_{20}H_{23}F_3O_3$ $[M+Na]^+$: 391.1492, found 391.1496.

Comparison of the ^{1}H NMR data of the two Mosher ester derivatives is shown below.

Assignment	δ, (*S*)-ester (ppm)	δ, (*R*)-ester (ppm)	$\Delta\delta^{SR}$
**H1**	5.07	4.99	+0.08
**H2**	5.76	5.65	+0.11
**H3**	2.54	2.48	+0.06
**CH_3**	1.04	0.96	+0.08
**H5**	1.70	1.75	–0.05
**H6**	1.41	1.53	–0.12
**H7**	2.12	2.19	–0.07
**H9**	1.93	1.95	–0.02

Analysis indicates that the C4 centre is of *S* configuration.

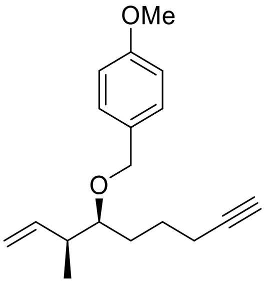

1-Methoxy-4-((((3*S*,4*S*)-3-methylnon-1-en-8-yn-4-yl)oxy)methyl)benzene (17.1). To a solution of alcohol **17** (1.38 g, 9.06 mmol, 1.0 eq.) in anhydrous DMF (18.1 mL) under argon at 0 °C was added NaH (60% in mineral oil, 724.8 mg, 18.12 mmol, 2.0 eq.). The mixture was stirred for 30 minutes, after which PMBCl (2.5 mL, 18.12 mmol, 2.0 eq.) and TBAI (699.3 mg, 1.81 mmol, 0.2 eq.) were added. The reaction mixture was stirred at room temperature overnight. It was quenched slowly with saturated aqueous NH_4Cl and extracted with diethyl ether. The organic layers were washed with brine, dried over Na_2SO_4 and concentrated under reduced pressure. The residue was purified by flash chromatography on silica gel (pentane/diethyl ether = 49/1) to afford the title compound (2.06 g, 7.56 mmol, 83%) as a colourless oil.

$[\boldsymbol{\alpha}]_D^{25}$ –38.9 (c = 1.1, $CHCl_3$); **R**$_f$ 0.43 (pentane/diethyl ether = 9/1); **IR** (thin film): ν_{max}/cm^{-1} 3300, 2954, 2936, 2867, 1612, 1513, 1247, 1173, 1078, 1036, 821; ^{1}**H NMR** (400 MHz, $CDCl_3$): δ 7.27 (d, *J* = 8.7 Hz, 2H, Ar*H*), 6.87 (d, *J* = 8.7 Hz, 2H, Ar*H*), 5.84 (ddd, *J* = 17.2, 10.4, 7.4 Hz, 1H, *H2*), 5.08 – 5.01 (m, 2H, *H1*), 4.50 (d, *J* = 11.0 Hz, 1H, OCH_2Ar), 4.44 (d, *J* = 11.0 Hz, 1H, OCH'_2Ar), 3.80 (s, 3H, OCH_3), 3.26 (ddd, *J* = 7.4, 5.7, 3.5 Hz, 1H, *H4*), 2.54 – 2.45 (m, 1H, *H3*), 2.19 – 2.14 (m, 2H, *H7*), 1.94 (t, *J* = 2.7 Hz, 1H, *H9*), 1.73 – 1.50 (m, 4H, *H5* and *H6*), 1.05 (d, *J* = 6.9 Hz, 3H, $CHCH_3$); ^{13}**C NMR** (101 MHz, $CDCl_3$): δ 159.3, 140.9, 131.1, 129.5, 114.6, 113.9, 84.7, 82.2, 71.5, 68.5, 55.4, 40.8, 30.2, 24.6, 18.6, 15.9; **HRMS** (ESI) m/z calcd for $C_{18}H_{24}O_2$ $[M+Na]^+$: 295.1669, found 295.1670.

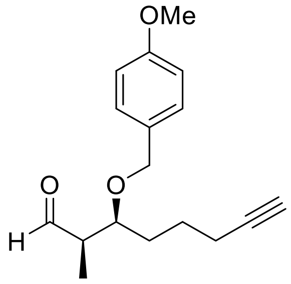

(2*R*,3*S*)-3-((4-methoxybenzyl)oxy)-2-methyloct-7-ynal (18). To a solution of alkene **17.1** (1.49 g, 5.47 mmol, 1.0 eq.) in acetone (54.7 mL) and water (18.2 mL) at 0 °C was added 4-methylmorpholine *N*-oxide (NMO) monohydrate (1.48 g, 10.94 mmol, 2.0 eq.), followed by potassium osmate dihydrate (60.4 mg, 0.164 mmol, 3 mol%). The reaction mixture was stirred at 0 °C overnight, allowing the cooling bath (acetone/dry ice) to warm to ~15 °C. Saturated aqueous $Na_2S_2O_3$ (45 mL) was added and the mixture was stirred vigorously for 2 hours. It was diluted with ethyl acetate and washed with saturated aqueous $NaHCO_3$. The aqueous layer was extracted with ethyl acetate and the combined organic layers were washed with brine, dried over Na_2SO_4, and concentrated under reduced pressure to afford a 3:1 diastereomeric mixture of diols (1.46 g, 4.77 mmol). The diol mixture was dissolved in CH_2Cl_2 (47.7 mL) and $NaIO_4$ (14 wt% on silica,[8] 18.2 g, 11.93 mmol, 2.5 eq.) was added. The reaction mixture was stirred at room temperature for 1 hour. It was filtered and concentrated under reduced pressure. The residue was purified by flash chromatography on silica gel (pentane/ethyl acetate = 9/1) to afford the title compound (940 mg, 3.43 mmol, 63% over two steps) as a colourless oil.

$[\boldsymbol{\alpha}]_{\boldsymbol{D}}^{\mathbf{25}}$ –36.8 (c = 0.6, $CHCl_3$); **R$_f$** 0.42 (pentane/ethyl acetate = 4/1); **IR** (thin film): ν_{max}/cm^{-1} 3291, 2939, 2870, 1720, 1613, 1514, 1249, 1174, 1065, 1034, 821; **^{1}H NMR** (400 MHz, $CDCl_3$): δ 9.76 (d, *J* = 1.1 Hz, 1H, C*H*O), 7.23 (d, *J* = 8.7 Hz, 2H, Ar*H*), 6.87 (d, *J* = 8.7 Hz, 2H, Ar*H*), 4.45 (s, 2H, OC*H*$_2$Ar), 3.84 – 3.81 (m, 1H, *H3*), 3.80 (s, 3H, OC*H*$_3$), 2.58 (qdd, *J* = 7.1, 3.9, 1.0 Hz, 1H, *H2*), 2.23 – 2.18 (m, 2H, *H6*), 1.96 (t, *J* = 2.6 Hz, 1H, *H8*), 1.75 – 1.51 (m, 4H, *H4* and *H5*), 1.13 (d, *J* = 7.0 Hz, 3H, C*H*$_3$); **^{13}C NMR** (101 MHz, $CDCl_3$): δ 204.7, 159.4, 130.3, 129.5, 114.0, 84.1, 77.8, 71.6, 68.9, 55.4, 49.8, 31.0, 24.9, 18.5, 8.5; **HRMS** (ESI) m/z calcd for $C_{17}H_{22}O_3$ $[M+Na]^+$: 297.1461, found 297.1463.

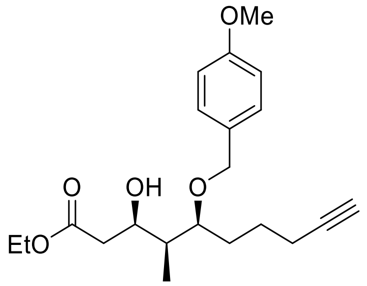

Ethyl (3*R*,4*S*,5*S*)-3-hydroxy-5-((4-methoxybenzyl)oxy)-4-methyldec-9-ynoate (19). To a solution of aldehyde **18** (1.51 g, 5.50 mmol, 1.0 eq.) and (1-ethoxyvinyl)oxy-(trimethyl)silane[9] (2.64 g, 16.50 mmol, 3.0 eq.) in anhydrous CH_2Cl_2 (110 mL) at –78 °C under argon was added dropwise BF_3•OEt_2 (679 µL, 5.50 mmol, 1.0 eq.) over 5 minutes. The reaction mixture was stirred at –78 °C for 1 hour, after which it was quenched with pH 7 buffer and extracted with CH_2Cl_2. The organic layers were washed with brine, dried over Na_2SO_4 and concentrated under reduced pressure. The residue was purified by flash chromatography on silica gel (pentane/ethyl acetate = 17/3) to afford the title compound (1.71 g, 4.72 mmol, 86%, 5:1 *dr*) as a colourless oil.

R_f 0.32 (pentane/ethyl acetate = 7/3); **IR** (thin film): ν_{max}/cm^{-1} 3498, 3293, 2938, 2909, 2361, 1730, 1612, 1514, 1248, 1176, 1034; **^{1}H NMR** (400 MHz, $CDCl_3$): δ 7.24 (d, *J* = 8.7, 2H, Ar*H*), 6.86 (d, *J* = 8.7 Hz, 2H, Ar*H*), 4.55 (d, *J* = 11.0 Hz, 1H, OC*H*$_2$Ar), 4.39 (d, *J* = 11.0 Hz, 1H, OC*H'*$_2$Ar), 4.22 – 4.15 (m, 1H, *H3*), 4.15 (q, *J* = 7.1 Hz, 2H, CO_2C*H*$_2$$CH_3$), 3.79 (s, 3H, OC*H*$_3$), 3.57 (ddd, *J* = 7.3, 5.7, 3.6 Hz, 1H, *H5*), 3.34 (d, *J* = 2.3 Hz, 1H, O*H*), 2.52 (dd, *J* = 15.9, 8.6 Hz, 1H, *H2*), 2.40 (dd, *J* = 15.9, 4.5 Hz, 1H, *H2'*), 2.21 (tdd, *J* = 6.9, 2.6, 1.1 Hz, 2H, *H8*), 1.97 (t, *J* = 2.6 Hz, 1H, *H10*), 1.87 – 1.60 (m, 4H, *H4* and *H6*), 1.57 – 1.50 (m, 2H, *H7*), 1.26 (t, *J* = 7.1 Hz, 3H, CO_2CH_2C*H*$_3$), 0.97 (d, *J* = 7.0 Hz, 3H, C*H*$_3$); **^{13}C NMR** (101 MHz, $CDCl_3$): δ 172.8, 159.4, 130.4, 129.5, 114.0, 84.2, 82.1, 71.0, 71.0, 68.9, 60.7, 55.4, 40.0, 39.5, 29.6, 24.8, 18.7, 14.3, 7.6; **HRMS** (ESI) m/z calcd for $C_{21}H_{30}O_5$ $[M+Na]^+$: 385.1986, found 385.1988.

Mosher ester analysis of alcohol 19. Alcohol **19** was derivatized to the (*R*)-MTPA and (*S*)-MTPA esters using (*S*)-(+)-MTPA-Cl and (*R*)-(–)-MTPA-Cl respectively according to a modified literature procedure.[7]

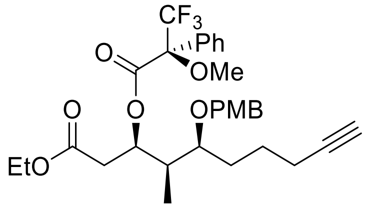

(*S*)-MTPA ester of 19. To a solution of alcohol **19** (10.0 mg, 0.028 mmol, 1.0 eq.) in CH_2Cl_2 (400 µL) was added pyridine (13.8 mg, 0.174 mmol, 6.2 eq.) and (*R*)-(–)-α-methoxy-α-(trifluoromethyl)phenylacetyl chloride (26.8 mg, 0.106 mmol, 3.8 eq.). The reaction mixture was stirred at room temperature overnight. Water was added and the mixture was extracted with CH_2Cl_2. The organic layers were dried over Na_2SO_4 and concentrated under reduced pressure. The residue was purified by flash chromatography on silica gel (pentane/ethyl acetate = 9/1 → pentane/ethyl acetate = 4/1) to afford the title compound (14.2 mg, 0.025 mmol, 89%)[f] as a colourless oil.

R_f 0.34 (pentane/ethyl acetate = 4/1); **IR** (thin film): ν_{max}/cm^{-1} 3288, 2952, 2361, 2341, 1746, 1514, 1250, 1172, 1022; **^{1}H NMR** (500 MHz, $CDCl_3$): δ 7.57 – 7.54 (m, 2H, Ar*H*), 7.40 – 7.39 (m, 3H, Ar*H*), 7.21 (d, *J* = 8.6 Hz, 2H, Ar*H*), 6.86 (d, *J* = 8.6 Hz, 2H, Ar*H*), 5.52 (dt, *J* = 7.7, 4.9 Hz, 1H, *H3*), 4.38 (d, *J* = 11.1 Hz, 1H, OCH_2Ar), 4.29 (d, *J* = 11.1 Hz, 1H, OCH'_2Ar), 4.09 (q, *J* = 7.2 Hz, 2H, $CO_2CH_2CH_3$), 3.80 (s, 3H, OCH_3), 3.55 (s, 3H, OCH_3), 3.22 – 3.18 (m, 1H, *H5*), 2.75 – 2.63 (m, 2H, *H2*), 2.14 (tdd, *J* = 6.8, 2.7, 1.0 Hz, 2H, *H8*), 2.07 – 2.02 (m, 1H, *H4*), 1.96 (t, *J* = 2.6 Hz, 1H, *H10*), 1.61 – 1.57 (m, 2H, *H6*), 1.50 – 1.41 (m, 2H, *H7*), 1.22 (t, *J* = 7.2 Hz, 3H, $CO_2CH_2CH_3$), 0.91 (d, *J* = 7.0 Hz, 3H, CH_3); **^{13}C NMR** (126 MHz, $CDCl_3$): δ 170.5, 165.9, 159.3, 132.4, 130.6, 129.7, 129.5, 128.5, 127.5, 123. 46 (q, *J* = 289 Hz), 113.9, 84.5 (q, *J* = 28 Hz), 84.2, 78.3, 74.9, 71.1, 68.8, 61.0, 55.6, 55.4, 38.8, 36.9, 29.8, 24.4, 18.6, 14.2, 10.4; **^{19}F NMR** (470 MHz, $CDCl_3$) δ –71.1; **HRMS** (ESI) m/z calcd for $C_{31}H_{37}F_3O_7$ $[M+Na]^+$: 601.2384, found 601.2377.

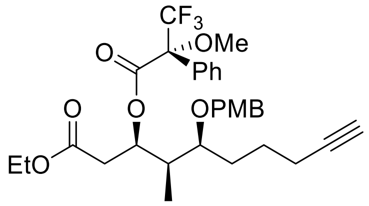

(*R*)-MTPA ester of 19. Synthesized in the same manner as the (*S*)-MTPA ester of **19** using 0.028 mmol of alcohol **19** and substituting (*R*)-(–)-α-methoxy-α-(trifluoromethyl)phenylacetyl chloride with (*S*)-(+)-α-methoxy-α-(trifluoromethyl)phenylacetyl chloride. The title compound (13.6 mg, 0.024 mmol, 85%)[f] was obtained as a colourless oil.

R_f 0.34 (pentane/ethyl acetate = 4/1); **IR** (thin film): ν_{max}/cm^{-1} 3302, 2950, 2360, 2341, 1745, 1514, 1250, 1173, 1030; **^{1}H NMR** (400 MHz, $CDCl_3$): δ 7.56 – 7.51 (m, 2H, Ar*H*), 7.43 – 7.37 (m, 3H, Ar*H*), 7.23 (d, *J* = 8.6 Hz, 2H, Ar*H*), 6.86 (d, *J* = 8.6 Hz, 2H, Ar*H*), 5.53 (dt, *J* = 7.4, 5.1 Hz, 1H, *H3*), 4.43 (d, *J* = 11.0 Hz, 1H, OCH_2Ar), 4.35 (d, *J* = 11.0 Hz, 1H, OCH'_2Ar), 4.05 (q, *J* = 7.1 Hz, 2H, $CO_2CH_2CH_3$), 3.80 (s, 3H, OCH_3), 3.46 (s, 3H, OCH_3), 3.35 – 3.30 (m, 1H, *H5*), 2.73 – 2.57 (m, 2H, *H2*), 2.18 (tdd, *J* = 6.8, 2.7, 1.4 Hz, 2H, *H8*), 2.13 – 2.07 (m, 1H, *H4*), 1.97 (t, *J* = 2.7 Hz, 1H, *H10*), 1.71 – 1.65 (m, 2H, *H6*), 1.61 – 1.47 (m, 2H, *H7*), 1.21 (t, *J* = 7.1 Hz, 3H, $CO_2CH_2CH_3$), 0.98 (d, *J* = 7.1 Hz, 3H, CH_3); **^{13}C NMR** (101 MHz, $CDCl_3$): δ 170.3, 165.9, 159.3, 132.0, 130.6, 129.7, 129.5, 128.6, 127.8, 123.5 (q, *J* = 289 Hz), 113.9, 84.8 (q, *J* = 28 Hz), 84.2, 78.6, 75.1, 71.1, 68.9, 60.9, 55.4, 55.4, 38.7, 36.9, 29.7, 24.5, 18.6, 14.2, 10.5; **^{19}F NMR** (470 MHz, $CDCl_3$) δ –71.2; **HRMS** (ESI) m/z calcd for $C_{31}H_{37}F_3O_7$ $[M+Na]^+$: 601.2384, found 601.2380.

[f]Obtained as a mixture of diastereomers (carried over from the starting material)

Comparison of the ^{1}H NMR data of the two Mosher ester derivatives is shown below.

Assignment	δ, (*S*)-ester (ppm)	δ, (*R*)-ester (ppm)	$\Delta\delta^{SR}$
**ester CH_3**	1.22	1.21	+0.01
**ester CH_2**	4.09	4.05	+0.04
**H2**	2.69	2.66	+0.03
**H4**	2.04	2.09	–0.05
**CH_3**	0.91	0.98	–0.07
**H5**	3.20	3.33	–0.13
**H6**	1.59	1.68	–0.09
**H7**	1.46	1.52	–0.06
**H8**	2.14	2.18	–0.04
**H10**	1.96	1.97	–0.01

Analysis indicates that the C3 centre is of *S* configuration.

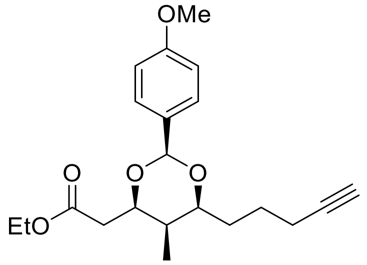

Ethyl 2-((4*R*,5*R*,6*S*)-2-(4-methoxyphenyl)-5-methyl-6-(pent-4-yn-1-yl)-1,3-dioxan-4-yl) acetate (3). To a mixture of alcohol **19** (1.15 g, 3.17 mmol, 1.0 eq.) and molecular sieves (3Å, 3.42 g) in anhydrous CH_2Cl_2 (63.4 mL) at 0 °C under argon was added DDQ (1.08 g, 4.76 mmol, 1.5 eq.) in three portions. The reaction mixture was stirred at 0 °C for 2 hours. It was filtered through celite, and the filtrate was washed with saturated aqueous $NaHCO_3$. The aqueous layer was further extracted with CH_2Cl_2. The organic layers were dried over Na_2SO_4 and concentrated under reduced pressure. The residue was purified by flash chromatography on silica gel (pentane/ethyl acetate = 9/1) to afford the title compound as a colourless oil (pure major diastereomer: 623.0 mg, 1.73 mmol, 55%; mixture of major and minor diastereomers: 142.8 mg, 0.40 mmol, 13%).

$[\alpha]_D^{25}$ –5.8 (c = 1.0, $CHCl_3$); **R$_f$** 0.42 (pentane/ethyl acetate = 4/1); **IR** (thin film): ν_{max}/cm^{-1} 3290, 2941, 2360, 1734, 1518, 1249, 1182, 1063, 1033; **^{1}H NMR** (400 MHz, $CDCl_3$): δ 7.40 (d, *J* = 8.8 Hz, 2H, Ar*H*), 6.87 (d, *J* = 8.8 Hz, 2H, Ar*H*), 5.52 (s, 1H, C*H*PMP), 4.38 (ddd, *J* = 7.9, 5.9, 2.3 Hz, 1H, *H3*), 4.15 (qd, *J* = 7.1, 0.9 Hz, 2H, $CO_2CH_2CH_3$), 3.89 (ddd, *J* = 8.4, 4.6, 2.3 Hz, 1H, *H5*), 3.79 (s, 3H, OCH_3), 2.69 (dd, *J* = 15.7, 7.9 Hz, 1H, *H2*), 2.47 (dd, *J* = 15.7, 5.9 Hz, 1H, *H2'*), 2.29 – 2.18 (m, 2H, *H8*), 1.96 (t, *J* = 2.6 Hz, 1H, *H10*), 1.84 – 1.66 (m, 2H, *H6*), 1.65 – 1.51 (m, 3H, *H4* and *H7*), 1.26 (t, *J* = 7.1 Hz, 3H, $CO_2CH_2CH_3$), 0.99 (d, *J* = 6.9 Hz, 3H, CH_3); **^{13}C NMR** (101 MHz, $CDCl_3$): δ 171.2, 160.0, 131.4, 127.5, 113.7, 101.6, 84.4, 80.5, 77.3, 68.7, 60.7, 55.4, 38.2, 34.6, 31.9, 24.8, 18.5, 14.4, 6.1; **HRMS** (ESI) m/z calcd for $C_{21}H_{28}O_5$ $[M+Na]^+$: 383.1829, found 383.1826.

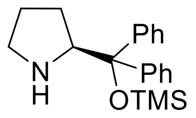

(*S*)-2-(diphenyl((trimethylsilyl)oxy)methyl)pyrrolidine ((*S*)-20). To a solution of imidazole (1.6 g, 23.7 mmol, 3.0 eq.) in anhydrous CH_2Cl_2 (21.6 mL) at 0 °C under argon was added chlorotrimethylsilane (2.5 mL, 19.8 mmol, 2.5 eq.) dropwise. The solution was stirred for 10 minutes, after which a solution of (*S*)-(–)-diphenylprolinol (2.0 g, 7.9 mmol, 1.0 eq.) in anhydrous CH_2Cl_2 (10 mL) was added dropwise. The reaction mixture was stirred at room temperature overnight. Water was added and the mixture was extracted with CH_2Cl_2. The organic layers were dried over Na_2SO_4 and concentrated under reduced pressure. The residue was purified by flash chromatography on silica gel (dichloromethane/methanol = 49/1) to afford the title compound (2.2 g, 6.8 mmol, 86%) as a light yellow oil. $[\alpha]_D^{25}$ –49.4 (*c* = 1.0, $CHCl_3$); **^{1}H NMR** (400 MHz, $CDCl_3$): δ 7.45 (dd, *J* = 8.4, 1.4 Hz, 2H), 7.35 (dd, *J* = 8.4, 1.4 Hz, 2H), 7.30 – 7.21 (m, 6H), 4.04 (t, J = 7.4 Hz, 1H), 2.89 – 2.75 (m, 2H), 1.78 (brs, 1H), 1.63 – 1.53 (m, 3H), 1.42 – 1.33 (m, 1H), –0.10 (s, 9H); **^{13}C NMR** (101 MHz, $CDCl_3$): δ 146.9, 145.9, 128.6, 127.8, 127.7, 127.7, 127.1, 126.9, 83.3, 65.6, 47.3, 27.6, 25.2, 2.3. The physical and spectroscopic data were consistent with literature reported data.[12]

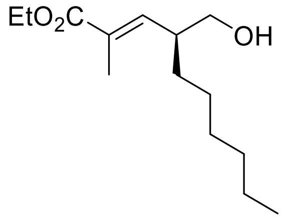

Ethyl (*S*,*E*)-4-(hydroxymethyl)-2-methyldec-2-enoate (21). Synthesized according to a modified literature procedure.[13] To a solution of catalyst (*S*)-**20** (488 mg, 1.5 mmol, 0.3 eq.) in toluene (10 mL) was added solid potassium phosphate buffer (pH 7, 2.5 g) and formaldehyde (37% in water, 1.1 mL, 15.0 mmol, 3.0 eq.). The mixture was stirred vigorously for 15 minutes, after which octanal (782 µL, 5.0 mmol, 1.0 eq.) was added in one portion. The reaction mixture was stirred at room temperature for 24 hours. It was diluted with water (2.5 mL), the organic layer was separated, and the aqueous layer was further extracted with toluene (2.5 mL). The organic layers were concentrated under reduced pressure (keeping the water bath at room temperature). The residue was dissolved in CH_2Cl_2 (5 mL) and added dropwise to a solution

of ethyl 2-(triphenylphosphoranylidene) propionate (5.44 g, 15.0 mmol, 3.0 eq.) in CH_2Cl_2 (15 mL). The reaction mixture was stirred at room temperature for 24 hours. It was concentrated under reduced pressure and the residue was taken up in a pentane/diethyl ether (4:1) solution. The mixture was filtered through celite and concentrated under reduced pressure. The residue was purified by flash chromatography on silica gel (pentane/ethyl acetate = 9/1 → pentane/ethyl acetate = 4/1) to afford the title compound (814 mg, 3.36 mmol, 67%, 88% *ee*) as a colourless oil.

$[\boldsymbol{\alpha}]_D^{25}$ +4.0 (c = 1.0, $CHCl_3$); **R_f** 0.27 (pentane/ethyl acetate = 4/1); **IR** (thin film): ν_{max}/cm^{-1} 3426, 2956, 2928, 2857, 1710, 1650, 1465, 1368, 1273, 1100, 1035, 751; **^{1}H NMR** (400 MHz, $CDCl_3$): δ 6.53 (dq, *J* = 10.4, 1.5 Hz, 1H, *H3*), 4.20 (q, *J* = 7.1 Hz, 2H, $CO_2CH_2CH_3$), 3.63 (ddd, *J* = 10.6, 7.3, 5.4 Hz, 1H, *H5*), 3.51 (ddd, *J* = 10.6, 7.6, 4.7 Hz, 1H, *H5'*), 2.70 – 2.61 (m, 1H, *H4*), 1.89 (d, *J* = 1.5 Hz, 3H, CCH_3), 1.53 – 1.21 (m, 13H, $CO_2CH_2CH_3$ and *n*-Hex-$(CH_2)_5$), 0.87 (t, *J* = 7.0 Hz, 3H, *n*-Hex-CH_3); **^{13}C NMR** (101 MHz, $CDCl_3$): δ 168.2, 143.4, 130.3, 66.2, 60.8, 42.1, 31.9, 31.2, 29.6, 27.3, 22.8, 14.4, 14.2, 13.2; **HRMS** (ESI) m/z calcd for $C_{14}H_{26}O_3$ $[M+Na]^+$: 265.1774, found 265.1774.

HPLC (CHIRALPAK-IC, *n*-hexane/isopropanol = 93/7, flow rate = 1.3 mL/min, λ = 254 nm): t_R = 7.9 min (major), 10.4 min (minor); 88% *ee*

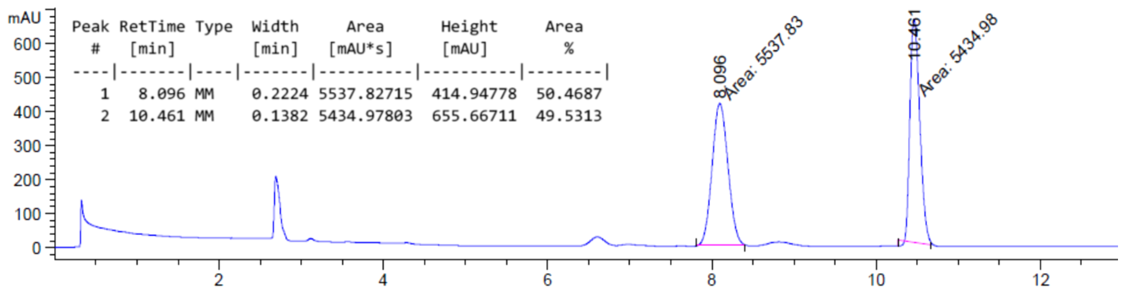

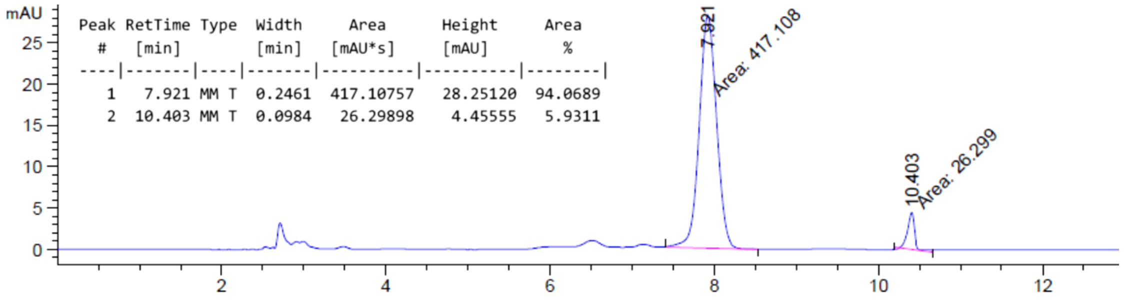

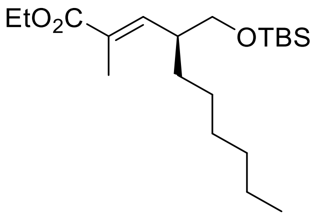

Ethyl (*S*,*E*)-4-(((tert-butyldimethylsilyl)oxy)methyl)-2-methyldec-2-enoate (21.1). To a solution of alcohol **21** (1.57 g, 6.5 mmol, 1.0 eq.) and imidazole (885 mg, 13.0 mmol, 2.0 eq.) in anhydrous CH_2Cl_2 (21.6 mL) at 0 °C under argon was added TBSCl (1.96 g, 13.0 mmol, 2.0 eq.). The reaction mixture was stirred at room temperature overnight. It was quenched with water and extracted with CH_2Cl_2. The organic layers were dried over Na_2SO_4 and concentrated under reduced pressure. The residue was purified by flash chromatography on silica gel (pentane/diethyl ether = 19/1) to afford the title compound (1.79 g, 5.0 mmol, 77%) as a colourless oil.

$[\boldsymbol{\alpha}]_D^{25}$ +11.3 (c = 1.0, $CHCl_3$); **R_f** 0.54 (pentane/diethyl ether = 9/1); **IR** (thin film): ν_{max}/cm^{-1} 2956, 2929, 2857, 1713, 1470, 1253, 1233, 1100, 837, 776; **^{1}H NMR** (400 MHz, $CDCl_3$): δ 6.53 (dq, *J* = 10.3, 1.4 Hz, 1H, *H3*), 4.19 (qd, *J* = 7.1, 6.2 Hz, 2H, $CO_2CH_2CH_3$), 3.52 (qd, *J* = 9.8, 6.2 Hz, 2H, *H5*), 2.62 – 2.53 (m, 1H, *H4*), 1.85 (d, *J* = 1.4 Hz, 3H, CCH_3), 1.55 – 1.21 (m, 13H, $CO_2CH_2CH_3$ and *n*-Hex-$(CH_2)_5$), 0.89 – 0.85 (m, 12H, *n*-Hex-CH_3 and $SiC(CH_3)_3$), 0.03 (s, 3H, $SiCH_3$), 0.02 (s, 3H, $SiCH_3$); **^{13}C NMR** (101 MHz, $CDCl_3$): δ 168.4, 144.4, 128.8, 66.1, 60.5, 42.0, 31.9, 31.3, 29.6, 27.3, 26.0, 22.8, 18.4, 14.4, 14.2, 13.1, –5.2, –5.3; **HRMS** (ESI) m/z calcd for $C_{20}H_{40}O_3Si$ $[M+Na]^+$: 379.2639, found 379.2639.

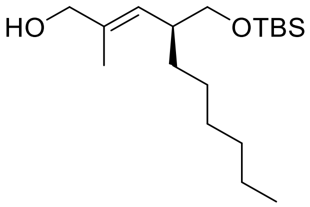

(*S*,*E*)-4-(((tert-butyldimethylsilyl)oxy)methyl)-2-methyldec-2-en-1-ol (22). To a solution of ester **21.1** (773.3 mg, 2.17 mmol, 1.0 eq.) in anhydrous CH_2Cl_2 (2.7 mL) at –78 °C under argon was added DIBALH (1.0 M in hexanes, 5.4 mL, 5.43 mmol, 2.5 eq.) dropwise. The reaction mixture was stirred at –78 °C for 1.5 hours. It was quenched slowly with water (290 μL), 15% aqueous NaOH (290 μL), then water (580 μL). The mixture was stirred at room

temperature for 15 minutes, dried over $MgSO_4$, filtered, and concentrated under reduced pressure. The residue was purified by flash chromatography on silica gel (pentane/ethyl acetate = 9/1) to afford the title compound (558.5 mg, 1.78 mmol, 82%) as a colourless oil.

$[\alpha]_D^{25}$ +14.2 (c = 1.0, $CHCl_3$); **R_f** 0.32 (pentane/diethyl ether = 9/1); **IR** (thin film): ν_{max}/cm^{-1} 3319, 2955, 2928, 2856, 1470, 1254, 1096, 836, 775; **^{1}H NMR** (400 MHz, $CDCl_3$): δ 5.14 (dq, *J* = 9.8, 1.4 Hz, 1H, *H3*), 4.01 (s, 2H, *H1*), 3.44 (d, *J* = 6.8 Hz, 2H, *H5*), 2.49 – 2.40 (m, 1H, *H4*), 1.68 (d, *J* = 1.4 Hz, 3H, CCH_3), 1.57 – 1.10 (m, 10H, *n*-Hex-(CH_2)$_5$), 0.89 – 0.86 (m, 12H, *n*-Hex-CH_3 and SiC(CH_3)$_3$), 0.03 (s, 3H, SiCH_3), 0.02 (s, 3H, SiCH_3); **^{13}C NMR** (101 MHz, $CDCl_3$): δ 136.1, 128.4, 69.2, 66.8, 40.8, 32.0, 31.8, 29.7, 27.3, 26.1, 22.8, 18.5, 14.4, 14.3, –5.1, –5.2; **HRMS** (ESI) m/z calcd for $C_{18}H_{38}O_2Si$ $[M+Na]^+$: 337.2533, found 337.2534.

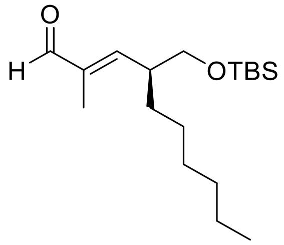

(*S*,*E*)-4-(((tert-butyldimethylsilyl)oxy)methyl)-2-methyldec-2-enal (4). To a solution of alcohol **22** (1.18 g, 3.75 mmol, 1.0 eq.) in CH_2Cl_2 (41.7 mL) was added $NaHCO_3$ (945.1 mg, 11.25 mmol, 3.0 eq.). The mixture was cooled to 0 °C and Dess-Martin periodinane (2.39 g, 5.63 mmol, 1.5 eq.) was added. The reaction mixture was stirred at room temperature for 1 hour. It was quenched with saturated aqueous $Na_2S_2O_3$ (42 mL) and extracted with diethyl ether. The organic layers were washed with saturated aqueous $NaHCO_3$, water and brine. It was dried over Na_2SO_4 and concentrated under reduced pressure. The residue was purified by flash chromatography on silica gel (pentane/diethyl ether = 19/1) to afford the title compound (1.09 g, 3.49 mmol, 93%) as a colourless oil.

$[\alpha]_D^{25}$ +17.0 (c = 1.2, $CHCl_3$); **R_f** 0.69 (pentane/diethyl ether = 9/1); **IR** (thin film): ν_{max}/cm^{-1} 2955, 2928, 2856, 1691, 1470, 1253, 1104, 836, 775; **^{1}H NMR** (400 MHz, $CDCl_3$): δ 9.42 (s, 1H, C*H*O), 6.30 (dq, *J* = 10.2, 1.4 Hz, 1H, *H3*), 3.63 (dd, *J* = 9.8, 5.4 Hz, 1H, *H5*), 3.56 (dd, *J* = 9.8, 6.5 Hz, 1H, *H5'*), 2.78 (dddt, *J* = 10.2, 6.5, 5.4, 8.7 Hz, 1H, *H4*), 1.77 (d, *J* = 1.4 Hz, 3H, CCH_3), 1.58 – 1.20 (m, 10H, *n*-Hex-(CH_2)$_5$), 0.87 – 0.85 (m, 12H, *n*-Hex-CH_3 and SiC(CH_3)$_3$),

0.02 (s, 3H, SiC*H*$_3$), 0.02 (s, 3H, SiC*H*$_3$); **^{13}C NMR** (101 MHz, $CDCl_3$): δ 195.6, 157.2, 140.3, 65.8, 42.3, 31.9, 31.1, 29.5, 27.3, 26.0, 22.7, 18.4, 14.2, 9.91, –5.3, –5.3; **HRMS** (ESI) m/z calcd for $C_{18}H_{36}O_2Si$ $[M+H]^+$: 313.2557, found 313.2558.

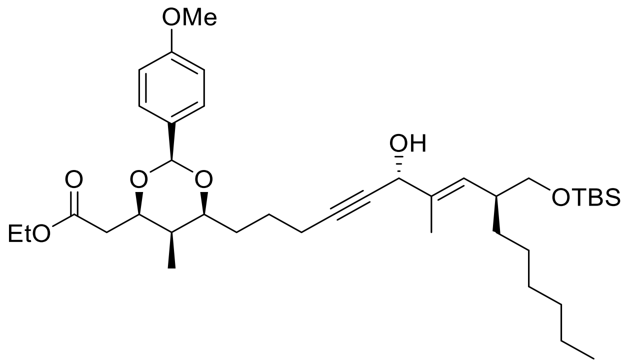

Ethyl 2-((4*R*,5*R*,6*S*)-6-((6*R*,9*S*,*E*)-9-(((tert-butyldimethylsilyl)oxy)methyl)-6-hydroxy-7-methylpentadec-7-en-4-yn-1-yl)-2-(4-methoxyphenyl)-5-methyl-1,3-dioxan-4-yl)acetate (23). Synthesized according to a modified literature procedure.[14] To a solution of alkyne **3** (865.1 mg, 2.40 mmol, 3.0 eq.) and (*S*)-BINOL (91.6 mg, 0.32 mmol, 0.4 eq.) in anhydrous diethyl ether (8 mL) under argon was added dicyclohexylamine (31.8 µL, 0.16 mmol, 0.2 eq.), followed by dropwise addition of diethyl zinc (1.0 M in hexanes, 2.4 mL, 2.40 mmol, 3.0 eq.). The reaction mixture was stirred at room temperature for 2–3 days (Note: the mixture should become cloudy with precipitate). $Ti(OiPr)_4$ (237 µL, 0.80 mmol, 1.0 eq.) was added and the resulting yellow/orange mixture was stirred at room temperature for 2 hours. It was cooled to 0 °C, and a solution of aldehyde **4** (250.0 mg, 0.80 mmol, 1.0 eq.) in anhydrous diethyl ether (2 mL) was added. The reaction mixture was stirred at 0 °C for 1 hour and at room temperature for 2 hours. It was cooled to 0 °C, quenched slowly with saturated aqueous NH_4Cl, filtered through celite and extracted with ethyl acetate. The organic layers were dried over Na_2SO_4 and concentrated under reduced pressure. The residue was purified by flash chromatography on silica gel (pentane/ethyl acetate = 9/1 → pentane/ethyl acetate = 4/1) to afford the title compound (331.7 mg, 0.49 mmol, 62%, 11:1 *dr*) as a colourless oil. Alkyne **3** (525.0 mg, 1.46 mmol) was also recovered.

$[\boldsymbol{\alpha}]_{\boldsymbol{D}}^{\mathbf{25}}$ –11.7 (c = 1.0, $CHCl_3$); **R_f** 0.28 (pentane/ethyl acetate = 4/1); **IR** (thin film): ν_{max}/cm^{-1} 3439, 2953, 2928, 2856, 1735, 1518, 1248, 1182, 1097, 1033, 834; **^{1}H NMR** (400 MHz, $CDCl_3$): δ 7.39 (d, *J* = 8.8 Hz, 2H, Ar*H*), 6.86 (d, *J* = 8.8 Hz, 2H, Ar*H*), 5.51 (s, 1H, C*H*PMP),

5.32 (d, *J* = 9.9 Hz, 1H, *H13*), 4.75 – 4.68 (m, 1H, *H11*), 4.37 (ddd, *J* = 7.9, 5.8, 2.2 Hz, 1H, *H3*), 4.14 (qd, *J* = 7.1, 1.0 Hz, 2H, $CO_2CH_2CH_3$), 3.88 (ddd, *J* = 8.4, 4.5, 2.1 Hz, 1H, *H5*), 3.77 (s, 3H, OCH_3), 3.46 (dd, *J* = 6.3, 1.8 Hz, 2H, *H15*), 2.68 (dd, *J* = 15.7, 7.9 Hz, 1H, *H2*), 2.46 (dd, *J* = 15.7, 5.8 Hz, 1H, *H2'*), 2.45 – 2.38 (m, 1H, *H14*), 2.31 – 2.23 (m, 2H, *H8*), 2.09 (d, *J* = 5.1 Hz, 1H, O*H*), 1.75 (d, *J* = 1.4 Hz, 3H, CC*H*3), 1.74 – 1.50 (m, 5H, *H4*, *H6* and *H7*), 1.34 – 1.08 (m, 13H, $CO_2CH_2CH_3$ and *n*-Hex-$(CH_2)_5$), 0.98 (d, *J* = 6.9 Hz, 3H, $CHCH_3$), 0.89 – 0.85 (m, 12H, *n*-Hex-CH_3 and $OSiC(CH_3)_3$), 0.03 (s, 3H, $OSiCH_3$), 0.03 (s, 3H, $OSiCH_3$); **^{13}C NMR** (101 MHz, $CDCl_3$): δ 171.1, 159.9, 136.0, 131.3, 129.5, 127.4, 113.6, 101.5, 85.9, 80.4, 77.2, 68.2, 66.5, 60.6, 55.3, 40.7, 38.1, 34.4, 31.9, 31.9, 31.6, 29.5, 27.0, 26.0, 24.8, 22.7, 18.8, 18.4, 14.2, 14.2, 12.9, 6.0, –5.3, –5.3; **HRMS** (ESI) m/z calcd for $C_{39}H_{64}O_7Si$ $[M+Na]^+$: 695.4314, found 695.4307.

Mosher ester analysis of alcohol 23. Alcohol **23** was derivatized to the (*R*)-MTPA and (*S*)-MTPA esters using (*S*)-(+)-MTPA-Cl and (*R*)-(–)-MTPA-Cl respectively according to a modified literature procedure.[7]

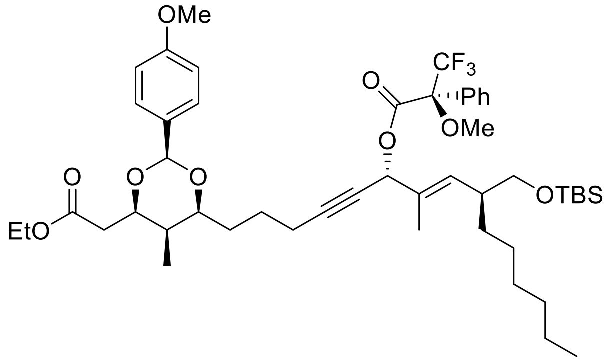

(*S*)-MTPA ester of 23. To a solution of alcohol **23** (23.0 mg, 0.034 mmol, 1.0 eq.) in CH_2Cl_2 (680 µL) was added pyridine (16.7 mg, 0.211 mmol, 6.2 eq.) and (*R*)-(–)-α-methoxy-α-(trifluoromethyl)phenylacetyl chloride (32.6 mg, 0.129 mmol, 3.8 eq.). The reaction mixture was stirred at room temperature overnight. Water was added and the mixture was extracted with CH_2Cl_2. The organic layers were dried over Na_2SO_4 and concentrated under reduced pressure. The residue was purified by flash chromatography on silica gel (pentane/ethyl acetate = 9/1) to afford the title compound (24.3 mg, 0.027 mmol, 80%) as a colourless oil.

$[\alpha]_D^{25}$ –20.9 (c = 2.4, $CHCl_3$); **R$_f$** 0.38 (pentane/ethyl acetate = 17/3); **IR** (thin film): ν_{max}/cm^{-1} 2953, 2929, 2856, 1745, 1518, 1249, 1183, 1171, 1102, 1034, 1014, 835; **^{1}H NMR** (500 MHz, $CDCl_3$): δ 7.52 (dd, *J* = 6.7, 3.0 Hz, 2H, Ar*H*), 7.39 (d, *J* = 8.8 Hz, 2H, Ar*H*), 7.38 – 7.36 (m, 3H, Ar*H*), 6.87 (d, *J* = 8.8 Hz, 2H, Ar*H*), 5.95 (s, 1H, *H11*), 5.51 (s, 1H, C*H*PMP), 5.50 (d, *J* = 10.0 Hz, 1H, *H13*), 4.37 (ddd, *J* = 8.0, 5.7, 2.3 Hz, 1H, *H3*), 4.16 (qd, *J* = 7.2, 1.8 Hz, 2H, $CO_2CH_2CH_3$), 3.85 (ddd, *J* = 8.6, 4.3, 2.2 Hz, 1H, *H5*), 3.79 (s, 3H, OCH_3), 3.52 (s, 3H, OCH_3), 3.45 (d, *J* = 6.2 Hz, 2H, *H15*), 2.69 (dd, *J* = 15.7, 8.0 Hz, 1H, *H2*), 2.46 (dd, *J* = 15.7, 5.7 Hz, 1H, *H2'*), 2.46 – 2.40 (m, 1H, *H14*), 2.31 – 2.21 (m, 2H, *H8*), 1.76 – 1.66 (m, 2H, *H6* and *H7*), 1.73 (d, *J* = 0.9 Hz, 3H, CCH_3), 1.56 – 1.50 (m, 3H, *H4*, *H6'* and *H7'*), 1.31 – 1.10 (m, 13H, $CO_2CH_2CH_3$ and *n*-Hex-$(CH_2)_5$), 0.96 (d, *J* = 6.9 Hz, 3H, $CHCH_3$), 0.88 – 0.85 (m, 12H, *n*-Hex-CH_3 and $OSiC(CH_3)_3$), 0.01 (s, 3H, $OSiCH_3$), 0.01 (s, 3H, $OSiCH_3$); **^{13}C NMR** (126 MHz, $CDCl_3$): δ 171.2, 165.6, 160.0, 134.1, 132.3, 131.4, 131.4, 129.7, 128.4, 127.7, 127.6, 123.4 (q, *J* = 289 Hz), 113.7, 101.6, 88.1, 84.8 (q, *J* = 28 Hz), 80.5, 77.3, 75.7, 72.2, 66.3, 60.7, 55.5, 55.4, 41.1, 38.2, 34.6, 32.0, 31.9, 31.5, 29.6, 27.1, 26.0, 24.8, 22.7, 18.9, 18.4, 14.3, 14.2, 13.4, 6.1, –5.2, –5.3; **^{19}F NMR** (470 MHz, $CDCl_3$): δ –71.8; **HRMS** (ESI) m/z calcd for $C_{49}H_{71}F_3O_9Si$ $[M+Na]^+$: 911.4712, found 911.4712.

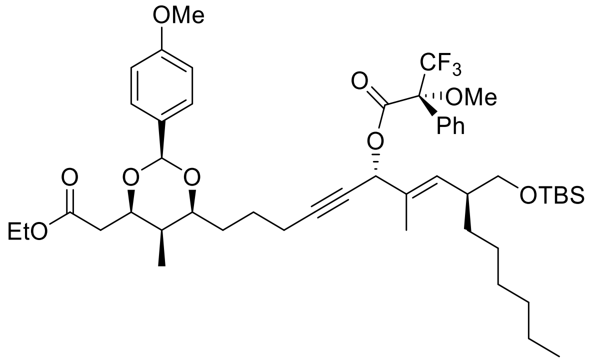

(*R*)-MTPA ester of 23. Synthesized in the same manner as the (S)-MTPA ester of **23** using 0.034 mmol of alcohol **23** and substituting (*R*)-(–)-α-methoxy-α-(trifluoromethyl)phenylacetyl chloride with (*S*)-(+)-α-methoxy-α-(trifluoromethyl)phenylacetyl chloride. The title compound (23.1 mg, 0.026 mmol, 76%) was obtained as a colourless oil.

$[\alpha]_D^{25}$ +9.7 (c = 2.3, $CHCl_3$); **R$_f$** 0.38 (pentane/ethyl acetate = 17/3); **IR** (thin film): ν_{max}/cm^{-1} 2952, 2929, 2856, 1745, 1518, 1249, 1183, 1171, 1106, 1034, 1014, 835; **^{1}H NMR** (500 MHz, $CDCl_3$): δ 7.55 (dd, *J* = 6.7, 2.9 Hz, 2H, Ar*H*), 7.39 (d, *J* = 8.8 Hz, 2H, Ar*H*), 7.37 – 7.35 (m,

3H, Ar*H*), 6.87 (d, *J* = 8.8 Hz, 2H, Ar*H*), 5.97 (s, 1H, *H11*), 5.51 (s, 1H, C*H*PMP), 5.45 (d, *J* = 10.0 Hz, 1H, *H13*), 4.37 (ddd, *J* = 8.0, 5.7, 2.3 Hz, 1H, *H3*), 4.15 (qd, *J* = 7.1, 1.6 Hz, 2H, $CO_2CH_2CH_3$), 3.86 (ddd, *J* = 8.3, 4.6, 2.1 Hz, 1H, *H5*), 3.79 (s, 3H, OCH_3), 3.57 (s, 3H, OCH_3), 3.43 (dd, *J* = 6.2, 2.0 Hz, 2H, *H15*), 2.69 (dd, *J* = 15.7, 8.0 Hz, 1H, *H2*), 2.46 (dd, *J* = 15.7, 5.7 Hz, 1H, *H2'*), 2.44 – 2.38 (m, 1H, *H14*), 2.34 – 2.25 (m, 2H, *H8*), 1.77 – 1.68 (m, 2H, *H6* and *H7*), 1.64 (d, *J* = 0.9 Hz, 3H, CCH_3), 1.58 – 1.51 (m, 3H, *H4*, *H6'* and *H7'*), 1.31 – 1.10 (m, 13H, $CO_2CH_2CH_3$ and *n*-Hex-$(CH_2)_5$), 0.96 (d, *J* = 6.8 Hz, 3H, $CHCH_3$), 0.88 – 0.85 (m, 12H, *n*-Hex-CH_3 and $OSiC(CH_3)_3$), 0.00 (s, 3H, $OSiCH_3$), –0.01 (s, 3H, $OSiCH_3$); **^{13}C NMR** (126 MHz, $CDCl_3$): δ 171.2, 165.6, 160.0, 133.7, 132.6, 131.3, 131.2, 129.7, 128.4, 127.5, 127.5, 123.4 (q, *J* = 289 Hz), 113.7, 101.6, 88.3, 84.5 (q, *J* = 28 Hz), 80.5, 77.3, 75.8, 71.8, 66.3, 60.7, 55.6, 55.4, 41.0, 38.2, 34.6, 32.0, 31.9, 31.5, 29.5, 27.1, 26.0, 24.8, 22.7, 18.9, 18.4, 14.3, 14.2, 13.2, 6.1, –5.2, –5.3; **^{19}F NMR** (470 MHz, $CDCl_3$): δ –71.7; **HRMS** (ESI) m/z calcd for $C_{49}H_{71}F_3O_9Si$ $[M+Na]^+$: 911.4712, found 911.4713.

Comparison of the ^{1}H NMR data of the two Mosher ester derivatives is shown below.

Assignment	δ, (*S*)-ester (ppm)	δ, (*R*)-ester (ppm)	$\Delta\delta^{SR}$
**H5**	3.85	3.86	–0.01
**H8**	2.26	2.29	–0.03
**CCH3**	1.73	1.64	+0.09
**H13**	5.50	5.45	+0.05
**H14**	2.43	2.41	+0.02
**H15**	3.45	3.43	+0.02

Analysis indicates that the C11 centre is of *R* configuration.

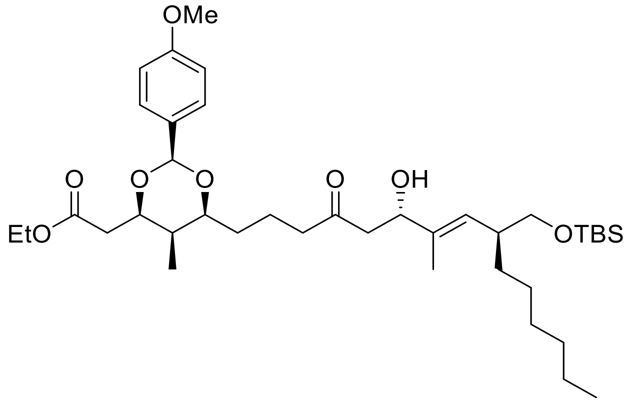

Ethyl 2-((4R,5R,6S)-6-((6S,9S,E)-9-(((tert-butyldimethylsilyl)oxy)methyl)-6-hydroxy-7-methyl-4-oxopentadec-7-en-1-yl)-2-(4-methoxyphenyl)-5-methyl-1,3-dioxan-4-yl)acetate (24). Synthesized according to a modified literature procedure.[15] To a solution of propagylic alcohol **23** (580.7 mg, 0.86 mmol, 1.0 eq.) in anhydrous diethyl ether (4.3 mL) under argon was added bis(pinacolato)diboron (523.1 mg, 2.06 mmol, 2.4 eq.), followed by sodium *tert*-butoxide (50.0 mg, 0.52 mmol, 0.6 eq.) in one portion. The mixture was cooled to 0 °C and IMesCuCl[16] (17.3 mg, 0.043 mmol, 5 mol%) was added, followed by anhydrous methanol (139 μL, 3.44 mmol, 4.0 eq.). The reaction mixture was stirred at 0 °C for 30 minutes. The dark-coloured mixture was quenched with 2-3 drops of triethanolamine and stirred at 0 °C for 10 minutes and at room temperature for 10 minutes. It was filtered through celite and washed with brine. The organic layer was dried over Na_2SO_4 and concentrated under reduced pressure. The residue was dissolved in THF (10.7 mL) and water (10.7 mL), and sodium perborate monohydrate (429.2 mg, 4.30 mmol, 5.0 eq.) was added. The reaction mixture was stirred at room temperature overnight. Water was added and the mixture was extracted with diethyl ether. The organic layers were dried over Na_2SO_4 and concentrated under reduced pressure. The residue was purified by flash chromatography on silica gel (pentane/ethyl acetate = 17/3) to afford the title compound (476.4 mg, 0.69 mmol, 80%) as a colourless oil.

$[\boldsymbol{\alpha}]_{\boldsymbol{D}}^{\mathbf{25}}$ +0.8 (c = 1.2, $CHCl_3$); **R**$_f$ 0.41 (pentane/ethyl acetate = 7/3); **IR** (thin film): ν_{max} / cm^{-1} 3497, 2954, 2928, 2855, 1736, 1714, 1518, 1248, 1181, 1097, 1032, 833; **^{1}H NMR** (400 MHz, $CDCl_3$): δ 7.36 (d, *J* = 8.7 Hz, 2H, Ar*H*), 6.83 (d, *J* = 8.7 Hz, 2H, Ar*H*), 5.48 (s, 1H, C*H*PMP), 5.15 (d, *J* = 9.9 Hz, 1H, *H13*), 4.42 (dd, *J* = 9.6, 3.5 Hz, 1H, *H11*), 4.34 (ddd, *J* = 8.0, 5.8, 2.2 Hz, 1H, *H3*), 4.12 (qd, *J* = 7.1, 1.3 Hz, 2H, $CO_2CH_2CH_3$), 3.83 (ddd, *J* = 7.8 , 5.0, 2.0 Hz, 1H, *H5*), 3.74 (s, 3H, OC*H*$_3$), 3.45 – 3.37 (m, 2H, *H15*), 2.87 (s, 1H, O*H*), 2.68 – 2.59 (m, 2H, *H2* and *H10*), 2.51 – 2.35 (m, 5H, *H2'*, *H8*, *H10'* and *H14*), 1.72 – 1.38 (m, 5H, *H4, H6* and *H7*),

1.61 (d, J = 1.0 Hz, 3H, CCH_3), 1.53 – 1.06 (m, 13H, $CO_2CH_2CH_3$ and n-Hex-(CH_2)$_5$), 0.95 (d, J = 6.9 Hz, 3H, $CHCH_3$), 0.87 – 0.83 (m, 12H, n-Hex-CH_3 and $OSiC(CH_3)_3$), 0.01 (s, 3H, $OSiCH_3$), 0.01 (s, 3H, $OSiCH_3$); **^{13}C NMR** (101 MHz, $CDCl_3$): δ 210.9, 170.9, 159.9, 137.0, 131.2, 128.4, 127.4, 113.5, 101.5, 80.6, 77.2, 73.2, 66.5, 60.5, 55.2, 48.1, 43.4, 40.5, 38.0, 34.3, 32.0, 31.8, 31.6, 29.5, 27.1, 25.9, 22.6, 19.6, 18.3, 14.2, 14.1, 12.4, 5.9, –5.3, –5.3; **HRMS** (ESI) m/z calcd for $C_{39}H_{66}O_8Si$ $[M+Na]^+$: 713.4430, found 713.4412.

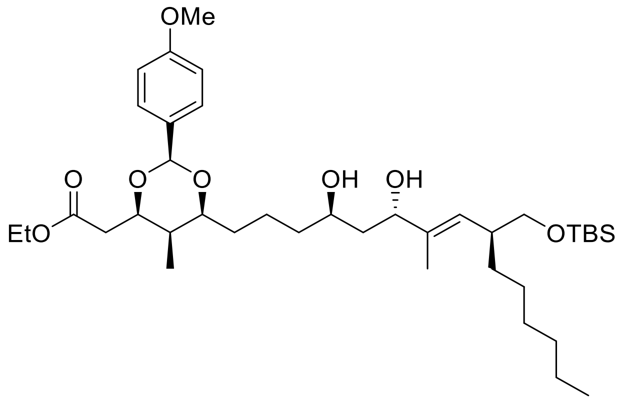

Ethyl 2-((4*R*,5*R*,6*S*)-6-((4*R*,6*S*,9*S*,*E*)-9-(((tert-butyldimethylsilyl)oxy)methyl)-4,6-dihydroxy-7-methylpentadec-7-en-1-yl)-2-(4-methoxyphenyl)-5-methyl-1,3-dioxan-4-yl)acetate (24.1). Synthesized according to a modified literature procedure.[17] To a stirred suspension of tetramethylammonium triacetoxyborohydride (1.05 g, 4.00 mmol, 5.8 eq.) in anhydrous acetonitrile (18 mL) under argon was added acetic acid (3.8 mL). The solution was cooled to –20 °C and a solution of β-hydroxy ketone **24** (476.4 mg, 0.69 mmol, 1.0 eq.) in anhydrous acetonitrile (5 mL) was added. The reaction mixture was stirred at –20 °C overnight. The mixture was quenched with saturated aqueous potassium sodium tartrate (16.3 mL), warmed to room temperature and stirred for 30 minutes, upon which a white precipitate formed. It was poured over ice, neutralized with saturated aqueous $NaHCO_3$ and extracted with ethyl acetate. The organic layers were dried over Na_2SO_4 and concentrated under reduced pressure. The residue was purified by flash chromatography on silica gel (pentane/ethyl acetate = 7/3) to afford the title compound (432.1 mg, 0.62 mmol, 90%, >20:1 *dr*) as a colourless oil.

$[\alpha]_D^{25}$ +2.6 (c = 1.1, $CHCl_3$); **R_f** 0.27 (pentane/ethyl acetate = 3/2); **IR** (thin film): ν_{max} / cm^{-1} 3413, 2952, 2928, 2856, 1736, 1518, 1248, 1181, 1098, 1060, 1032, 834; **^{1}H NMR** (400 MHz, $CDCl_3$): δ 7.37 (d, J = 8.8 Hz, 2H, ArH), 6.84 (d, J = 8.8 Hz, 2H, ArH), 5.49 (s, 1H, CHPMP),

5.17 (d, J = 9.9 Hz, 1H, *H13*), 4.34 (ddd, J = 8.0, 5.7, 2.2 Hz, 1H, *H3*), 4.26 (dd, J = 8.3, 3.5 Hz, 1H, *H11*), 4.12 (qd, J = 7.1, 1.0 Hz, 2H, $CO_2CH_2CH_3$), 3.86 – 3.78 (m, 2H, *H5* and *H9*), 3.74 (s, 3H, OCH_3), 3.43 (d, J = 6.5 Hz, 2H, *H15*), 2.80 (s, 1H, O*H*), 2.66 (dd, J = 15.7, 8.0 Hz, 1H, *H2*), 2.44 (dd, J = 15.7, 5.7 Hz, 1H, *H2'*), 2.44 – 2.37 (m, 1H, *H14*), 1.75 – 1.32 (m, 9H, *H4*, *H6*, *H7*, *H8* and *H10*), 1.60 (d, J = 0.9 Hz, 3H, CCH_3), 1.54 – 1.06 (m, 13H, $CO_2CH_2CH_3$ and *n*-Hex-$(CH_2)_5$), 0.95 (d, J = 6.8 Hz, 3H, $CHCH_3$), 0.88 – 0.84 (m, 12H, *n*-Hex-CH_3 and $OSiC(CH_3)_3$), 0.02 (s, 3H, $OSiCH_3$), 0.01 (s, 3H, $OSiCH_3$); **^{13}C NMR** (101 MHz, $CDCl_3$): δ 171.1, 159.8, 138.4, 131.3, 127.4, 113.5, 101.5, 80.8, 77.2, 74.4, 68.9, 66.8, 60.5, 55.2, 41.0, 40.5, 38.1, 37.2, 34.3, 32.6, 31.8, 31.7, 29.5, 27.1, 26.0, 22.7, 21.7, 18.3, 14.2, 14.1, 12.9, 6.0, –5.3, –5.3; **HRMS** (ESI) m/z calcd for $C_{39}H_{68}O_8Si$ $[M+Na]^+$: 715.4587, found 715.4569.

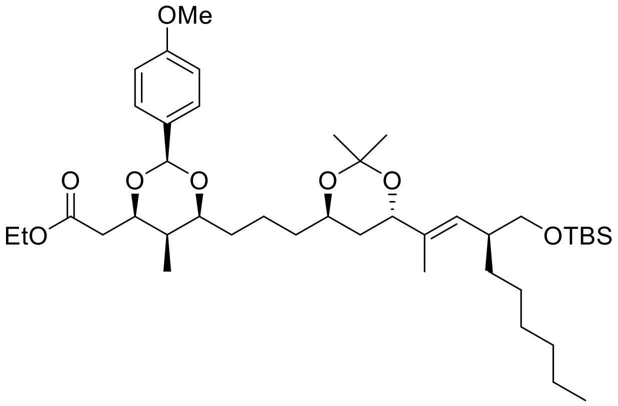

Ethyl 2-((4*R*,5*R*,6*S*)-6-(3-((4*R*,6*S*)-6-((*S*,*E*)-4-(((tert-butyldimethylsilyl)oxy)methyl)dec-2-en-2-yl)-2,2-dimethyl-1,3-dioxan-4-yl)propyl)-2-(4-methoxyphenyl)-5-methyl-1,3-dioxan-4-yl)acetate (25). To a solution of diol **24.1** (365.3 mg, 0.53 mmol, 1.0 eq.) in 2,2-dimethoxypropane/CH_2Cl_2 (3.8 mL, 1:1) was added pyridinium *p*-toluenesulfonate (11 mg, 0.04 mmol, 8 mol%). The reaction mixture was stirred at room temperature for 24 hours. It was quenched with saturated aqueous $NaHCO_3$ and extracted with CH_2Cl_2. The organic layers were dried over Na_2SO_4 and concentrated under reduced pressure. The residue was purified by flash chromatography on silica gel (pentane/diethyl ether = 17/3) to afford the title compound (311.7 mg, 0.43 mmol, 80%) as a colourless oil.

$[\alpha]_D^{25}$ –10.0 (c = 1.1, $CHCl_3$); **R_f** 0.39 (pentane/diethyl ether = 3/1); **IR** (thin film): ν_{max}/cm^{-1} 2951, 2928, 2856, 1738, 1518, 1377, 1249, 1171, 1101, 1034, 835; **^{1}H NMR** (400 MHz,

CDCl$_3$): δ 7.40 (d, *J* = 8.7 Hz, 2H, Ar*H*), 6.87 (d, *J* = 8.7 Hz, 2H, Ar*H*), 5.52 (s, 1H, C*H*PMP), 5.14 (d, *J* = 9.7 Hz, 1H, *H13*), 4.37 (ddd, *J* = 7.8, 5.8, 2.2 Hz, 1H, *H3*), 4.21 – 4.12 (m, 3H, *H11* and CO$_2$C*H*$_2$CH$_3$), 3.87 (ddd, *J* = 7.6, 5.4, 2.1 Hz, 1H, *H5*), 3.83 – 3.78 (m, 1H, *H9*), 3.77 (s, 3H, OC*H*$_3$), 3.49 (dd, *J* = 9.8, 5.7 Hz, 1H, *H15*), 3.43 (dd, *J* = 9.8, 6.9 Hz, 1H, *H15'*), 2.69 (dd, *J* = 15.7, 7.8 Hz, 1H, *H2*), 2.47 (dd, *J* = 15.7, 5.8 Hz, 1H, *H2'*), 2.39 – 2.46 (m, 1H, *H14*), 1.86 – 1.40 (m, 9H, *H4*, *H6*, *H7*, *H8* and *H10*), 1.65 (d, *J* = 0.9 Hz, 3H, CC*H*$_3$), 1.36 (s, 3H, acetonide-C*H*$_3$), 1.36 (s, 3H, acetonide-C*H*$_3$), 1.56 – 1.09 (m, 13H, CO$_2$CH$_2$C*H*$_3$ and *n*-Hex-(C*H*$_2$)$_5$), 0.98 (d, *J* = 6.8 Hz, 3H, CHC*H*$_3$), 0.89 – 0.86 (m, 12H, *n*-Hex-C*H*$_3$ and OSiC(C*H*$_3$)$_3$), 0.03 (s, 3H, OSiC*H*$_3$), 0.03 (s, 3H, OSiC*H*$_3$); **^{13}C NMR** (101 MHz, CDCl$_3$): δ 171.1, 159.9, 136.0, 131.4, 127.9, 127.5, 113.6, 101.6, 100.3, 80.8, 77.3, 71.7, 66.6, 66.6, 60.6, 55.3, 40.4, 38.2, 37.0, 35.9, 34.2, 32.4, 31.9, 31.7, 29.6, 27.1, 26.0, 25.1, 24.9, 22.7, 21.2, 18.4, 14.3, 14.2, 12.7, 6.0, –5.2, –5.3; **HRMS** (ESI) m/z calcd for $C_{42}H_{72}O_8Si$ $[M+Na]^+$: 755.4889, found 755.4886.

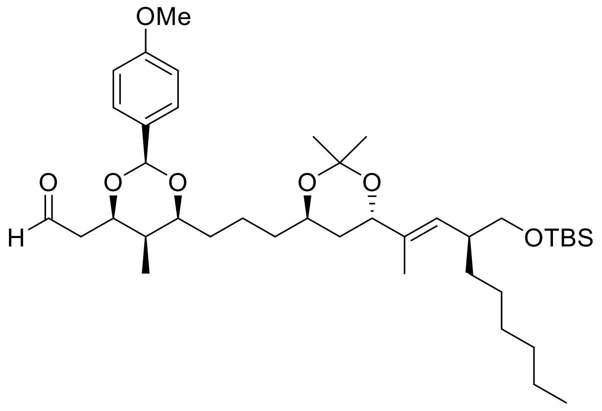

2-((4*R*,5*R*,6*S*)-6-(3-((4*R*,6*S*)-6-((*S*,*E*)-4-(((tert-butyldimethylsilyl)oxy)methyl)dec-2-en-2-yl)-2,2-dimethyl-1,3-dioxan-4-yl)propyl)-2-(4-methoxyphenyl)-5-methyl-1,3-dioxan-4-yl)acetaldehyde (25.1). To a solution of ester **25** (327.3 mg, 0.45 mmol, 1.0 eq.) in anhydrous CH$_2$Cl$_2$ (4.5 mL) at –78 °C under argon was added DIBALH (1.0 M in hexanes, 540 µL, 0.54 mmol, 1.2 eq.) dropwise. The reaction mixture was stirred at –78 °C for 15 minutes. It was quenched with ethyl acetate, followed by slow addition of water (65 µL), 15% aqueous NaOH (65 µL), and water (130 µL). The mixture was stirred at room temperature for 15 minutes, dried over MgSO$_4$, filtered and concentrated under reduced pressure. The residue was purified by flash chromatography on silica gel (pentane/ethyl acetate = 9/1) to afford the title compound (227.4 mg, 0.33 mmol, 73%) as a colourless oil.

$[\alpha]_D^{25}$ –3.7 (c = 1.1, $CHCl_3$); **R$_f$** 0.41 (pentane/ethyl acetate = 4/1); **IR** (thin film): ν_{max}/cm^{-1} 2950, 2928, 2856, 1729, 1518, 1378, 1249, 1224, 1170, 1111, 1063, 1036, 835; **^{1}H NMR** (400 MHz, $CDCl_3$): δ 9.83 (t, *J* = 1.8 Hz, 1H, C*H*O), 7.40 (d, *J* = 8.8 Hz, 2H, Ar*H*), 6.88 (d, *J* = 8.8 Hz, 2H, Ar*H*), 5.54 (s, 1H, C*H*PMP), 5.13 (d, *J* = 9.7 Hz, 1H, *H13*), 4.46 (ddd, *J* = 8.7, 4.5, 2.2 Hz, 1H, *H3*), 4.19 (dd, *J* = 9.3, 6.1 Hz, 1H, *H11*), 3.90 (ddd, *J* = 7.5, 5.3, 2.2 Hz, 1H, *H5*), 3.82 – 3.76 (m, 1H, *H9*), 3.79 (s, 3H, OCH_3), 3.48 (dd, *J* = 9.8, 5.7 Hz, 1H, *H15*), 3.42 (dd, *J* = 9.8, 6.9 Hz, 1H, *H15'*), 2.84 (ddd, *J* = 17.0, 8.7, 1.8 Hz, 1H, *H2*), 2.48 (ddd, *J* = 16.9, 4.5, 1.8 Hz, 1H, *H2'*), 2.45 – 2.38 (m, 1H, *H14*), 1.82 (ddd, *J* = 12.8, 9.3, 5.8 Hz, 1H, *H10*), 1.73 – 1.67 (m, 1H, *H6*), 1.65 (d, *J* = 1.3 Hz, 3H, CCH_3), 1.60 – 1.35 (m, 7H, *H4*, *H6'*, *H7*, *H8* and *H10'*), 1.36 (s, 3H, acetonide-CH_3), 1.35 (s, 3H, acetonide-CH_3), 1.56 – 1.09 (m, 10H, *n*-Hex-$(CH_2)_5$), 0.99 (d, *J* = 6.9 Hz, 3H, CHCH_3), 0.89 – 0.86 (m, 12H, *n*-Hex-CH_3 and OSiC$(CH_3)_3$), 0.02 (s, 3H, OSiCH_3), 0.02 (s, 3H, OSiCH_3); **^{13}C NMR** (101 MHz, $CDCl_3$): δ 200.8, 160.1, 136.0, 131.2, 128.0, 127.6, 113.8, 101.7, 100.4, 80.9, 75.9, 71.8, 66.7, 66.6, 55.4, 46.9, 40.5, 37.0, 36.0, 34.5, 32.5, 32.0, 31.8, 29.6, 27.2, 26.1, 25.1, 24.9, 22.8, 21.2, 18.5, 14.2, 12.8, 6.2, –5.1, –5.2; **HRMS** (ESI) m/z calcd for $C_{40}H_{68}O_7Si$ $[M+Na]^+$: 711.4627, found 711.4623.

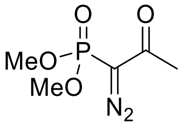

Dimethyl (1-diazo-2-oxopropyl)phosphonate (**S6**). Synthesized according to a modified literature producedure.[18] To a suspension of NaH (60% in mineral oil, 475.2 mg, 11.88 mmol, 1.1 eq.) in anhydrous THF (34 mL) at 0 °C under argon was added a solution of dimethyl 2-oxopropylphosphonate (1.5 mL, 10.80 mmol, 1.0 eq.) in anhydrous THF (20 mL) dropwise, upon which a white solid formed. The mixture was stirred at 0 °C for 1 hour, after which *p*-toluenesulfonyl azide solution (30% in toluene, 8.8 mL, 11.56 mmol, 1.07 eq.) was added. The resulting yellow reaction mixture was stirred at 0 °C for 3 hours. It was filtered through celite, washing with ethyl acetate, and concentrated under reduced pressure. The residue was purified by flash chromatography on silica gel (pentane/ethyl acetate = 3/2 → pentane/ethyl acetate = 1/4) to afford the title compound (1.82 g, 9.47 mmol, 88%) as a light yellow oil. **^{1}H NMR** (400 MHz, $CDCl_3$): δ 3.84 (s, 3H), 3.81 (s, 3H), 2.25 (s, 3H); **^{13}C NMR** (101 MHz, $CDCl_3$): δ 190.0, 189.9, 53.7, 53.7, 27.3. The physical and spectroscopic data were consistent with literature reported data.[18]

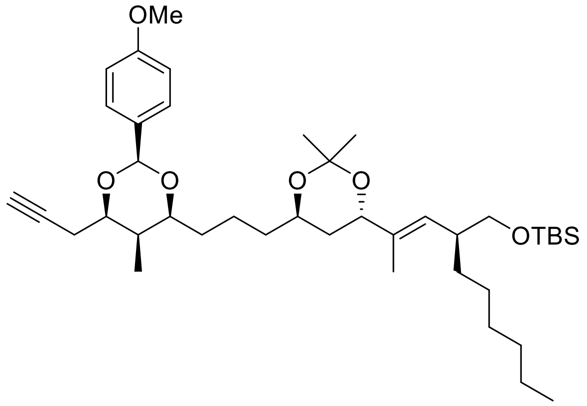

***tert*-Butyl(((2*S*)-2-((*E*)-2-((4*S*,6*R*)-6-(3-((4*S*,5*R*,6*R*)-2-(4-methoxyphenyl)-5-methyl-6-(prop-2-yn-1-yl)-1,3-dioxan-4-yl)propyl)-2,2-dimethyl-1,3-dioxan-4-yl)prop-1-en-1-yl)octyl)oxy) dimethylsilane (26).** To a solution of dimethyl (1-diazo-2-oxopropyl) phosphonate (**S6**, 441.1 mg, 2.30 mmol, 8.0 eq.[g]) in anhydrous MeOH (4.2 mL) under argon was added K_2CO_3 (325.2 mg, 2.35 mmol, 8.2 eq.), followed by a solution of aldehyde **25.1** (197.9 mg, 0.29 mmol, 1.0 eq.) in anhydrous MeOH (3 mL). The reaction mixture was stirred at room temperature for 2 hours. It was quenched with saturated aqueous NH_4Cl, diluted with water and extracted with diethyl ether. The organic layers were dried over Na_2SO_4 and concentrated under reduced pressure. The residue was purified by flash chromatography on silica gel (pentane/ethyl acetate = 19/1) to afford the title compound (158.6 mg, 0.23 mmol, 81%) as a colourless oil.

$[\boldsymbol{\alpha}]_{\boldsymbol{D}}^{\mathbf{25}}$ –16.3 (c = 1.0, $CHCl_3$); **R_f** 0.47 (pentane/ethyl acetate = 9/1); **IR** (thin film): ν_{max}/cm^{-1} 3313, 2951, 2928, 2856, 1517, 1378, 1249, 1171, 1105, 1062, 1037, 832; **^{1}H NMR** (400 MHz, $CDCl_3$): δ 7.42 (d, *J* = 8.7 Hz, 2H, Ar*H*), 6.88 (d, *J* = 8.7 Hz, 2H, Ar*H*), 5.49 (s, 1H, C*H*PMP), 5.14 (d, *J* = 9.7 Hz, 1H, *H14*), 4.20 (dd, *J* = 9.2, 6.2 Hz, 1H, *H12*), 4.04 (ddd, *J* = 9.3, 6.0, 2.3 Hz, 1H, *H4*), 3.86 – 3.77 (m, 2H, *H6* and *H10*), 3.79 (s, 3H, OCH_3), 3.49 (dd, *J* = 9.8, 5.7 Hz, 1H, *H16*), 3.43 (dd, *J* = 9.8, 6.9 Hz, 1H, *H16'*), 2.59 (ddd, *J* = 16.7, 6.0, 2.7 Hz, 1H, *H3*), 2.44 (ddd, *J* = 16.7, 9.3, 2.7 Hz, 1H, *H3'*), 2.46 – 2.40 (m, 1H, *H15*), 2.00 (t, *J* = 2.7 Hz, 1H, *H1*), 1.87 – 1.75 (m, 2H, *H5* and *H11*), 1.72 – 1.36 (m, 7H, *H7, H8, H9* and *H11'*), 1.66 (d, *J* = 1.3 Hz, 3H, CCH_3), 1.37 (s, 3H, acetonide-CH_3), 1.36 (s, 3H, acetonide-CH_3), 1.59 – 1.09 (m, 10H, *n*-Hex-(CH_2)$_5$), 0.99 (d, *J* = 6.9 Hz, 3H, CHCH_3), 0.90 – 0.86 (m, 12H, *n*-Hex-CH_3 and OSiC(CH_3)$_3$), 0.03 (s, 3H, OSiCH_3), 0.03 (s, 3H, OSiCH_3); **^{13}C NMR** (101 MHz, $CDCl_3$): δ

[g]An excess of the Ohira-Bestmann reagent is needed to prevent the formation of a side product (PMP acetal ring-opened compound), which lowers the yield of the alkyne and results in an inseparable impurity.

160.1, 136.1, 131.4, 127.9, 127.6, 113.8, 101.7, 100.3, 81.0, 80.1, 79.0, 71.8, 70.2, 66.7, 66.6, 55.4, 40.5, 37.0, 36.0, 33.2, 32.6, 32.0, 31.8, 29.6, 27.2, 26.1, 25.2, 25.0, 22.8, 22.5, 21.3, 18.5, 14.2, 12.8, 5.4, –5.1, –5.2; **HRMS** (ESI) m/z calcd for $C_{41}H_{68}O_6Si$ $[M+Na]^+$: 707.4688, found 707.4672.

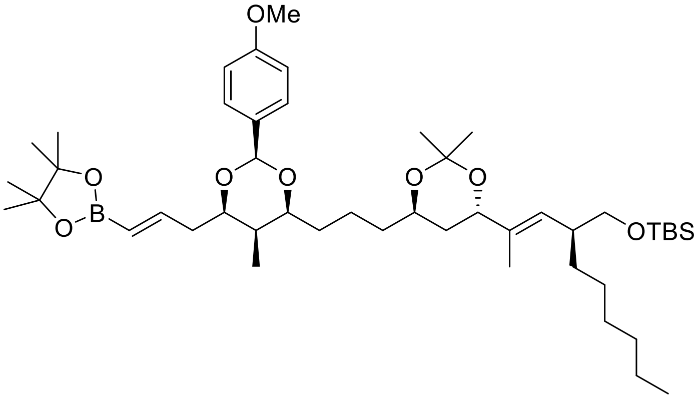

***tert*-Butyl(((2*S*)-2-((*E*)-2-((4*S*,6*R*)-6-(3-((4*S*,5*R*,6*R*)-2-(4-methoxyphenyl)-5-methyl-6-((*E*)-3-(4,4,5,5-tetramethyl-1,3,2-dioxaborolan-2-yl)allyl)-1,3-dioxan-4-yl)propyl)-2,2-dimethyl-1,3-dioxan-4-yl)prop-1-en-1-yl)octyl)oxy)dimethylsilane (27).** Synthesized according to a modified literature procedure.[19] To a mixture of alkyne **26** (71.3 mg, 0.104 mmol, 1.0 eq.) and pinacolborane (189 µL, 1.300 mmol, 12.5 eq.) under argon was added bis(cyclopentadienyl)zirconium chloride hydride (2.7 mg, 0.0104 mmol, 0.1 eq.) and anhydrous triethylamine (14.5 µL, 0.104 mmol, 1.0 eq.). The reaction mixture was heated at 60 °C for 2 hours. Upon cooling to room temperature, the mixture was diluted with diethyl ether, quenched with brine and extracted with diethyl ether. The organic layers were dried over Na_2SO_4 and concentrated under reduced pressure. The residue was purified by flash chromatography on silica gel (pentane/ethyl acetate = 19/1) to afford the title compound (62.7 mg, 0.077 mmol, 74%) as a colourless oil.

$[\alpha]_D^{25}$ +4.0 (c = 1.2, $CHCl_3$); **R_f** 0.43 (pentane/ethyl acetate = 9/1); **IR** (thin film): ν_{max}/cm^{-1} 2951, 2928, 2855, 1640, 1518, 1358, 1248, 1144, 1100, 1063, 1003, 834; **^{1}H NMR** (400 MHz, $CDCl_3$): δ 7.42 (d, *J* = 8.7 Hz, 2H, Ar*H*), 6.87 (d, *J* = 8.7 Hz, 2H, Ar*H*), 6.59 (ddd, *J* = 18.0, 7.6, 5.8 Hz, 1H, *H2*), 5.56 (d, *J* = 18.0 Hz, 1H, *H1*), 5.47 (s, 1H, C*H*PMP), 5.12 (d, *J* = 9.8 Hz, 1H, *H14*), 4.18 (dd, *J* = 9.4, 6.1 Hz, 1H, *H12*), 3.93 (td, *J* = 7.0, 2.0 Hz, 1H, *H4*), 3.81 – 3.76 (m, 2H, *H6* and *H10*), 3.79 (s, 3H, OC*H*3), 3.48 (dd, *J* = 9.9, 5.7 Hz, 1H, *H16*), 3.42 (dd, J =

9.8, 7.0 Hz, 1H, *H16'*), 2.59 – 2.52 (m, 1H, *H3*), 2.46 – 2.32 (m, 2H, H3' and *H15*), 1.82 (ddd, *J* = 12.8, 9.4, 5.9 Hz, 1H, *H11*), 1.69 – 1.34 (m, 8H, *H5*, *H7, H8, H9* and *H11'*), 1.64 (d, *J* = 1.0 Hz, 3H, CCH_3), 1.36 (s, 3H, acetonide-CH_3), 1.35 (s, 3H, acetonide-CH_3), 1.58 – 1.09 (m, 22H, Bpin-CH_3 × 4 and *n*-Hex-$(CH_2)_5$), 0.97 (d, *J* = 6.8 Hz, 3H, $CHCH_3$), 0.89 – 0.85 (m, 12H, *n*-Hex-CH_3 and $OSiC(CH_3)_3$), 0.02 (s, 3H, $OSiCH_3$), 0.02 (s, 3H, $OSiCH_3$); **^{13}C NMR** (101 MHz, $CDCl_3$): δ 160.0, 149.4, 136.1, 131.7, 127.9, 127.6, 113.7, 101.7, 100.4, 83.3, 81.1, 80.1, 71.8, 66.7, 66.7, 55.4, 40.5, 39.4, 37.1, 36.1, 34.1, 32.6, 32.0, 31.8, 29.7, 27.2, 26.1, 25.2, 24.9, 24.9, 22.8, 21.3, 18.5, 14.3, 12.8, 5.8, –5.1, –5.2; **HRMS** (ESI) m/z calcd for $C_{47}H_{81}BO_8Si$ $[M+Na]^+$: 834.5722, found 834.5717.

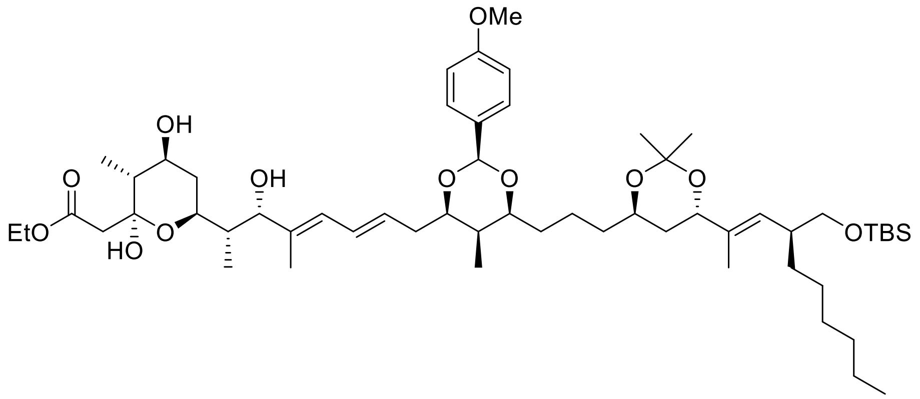

Ethyl 2-((2*S*,3*R*,4*S*,6*S*)-6-((2*S*,3*S*,4*E*,6*E*)-8-((4*R*,5*R*,6*S*)-6-(3-((4*R*,6*S*)-6-((*S*,*E*)-4-(((*tert*-butyl-dimethylsilyl)oxy)methyl)dec-2-en-2-yl)-2,2-dimethyl-1,3-dioxan-4-yl)propyl)-2-(4-methoxyphenyl)-5-methyl-1,3-dioxan-4-yl)-3-hydroxy-4-methylocta-4,6-dien-2-yl)-2,4-dihydroxy-3-methyltetrahydro-2*H*-pyran-2-yl)acetate (28). To a solution of iodide **2** (8.4 mg, 0.019 mmol, 1.1 eq.), boronate **27** (14.0 mg, 0.017 mmol, 1.0 eq.) and $Pd(PPh_3)_4$ (5.9 mg, 0.0051 mmol, 0.3 eq.) in degassed anhydrous THF (2.1 mL) under argon was added dropwise a solution of Tl_2CO_3 (14.5 mg, 0.031 mmol, 1.8 eq.) in degassed water (680 μL) over 5 minutes. (Note: Tl_2CO_3 was dissolved in degassed water under argon and stirred for 30 minutes prior to addition to the reaction mixture.) The reaction mixture was stirred at room temperature overnight. It was diluted with ethyl acetate, water was added, and the mixture was extracted with ethyl acetate. The organic layers were dried over Na_2SO_4 and concentrated under reduced pressure. The residue was purified by flash chromatography on silica gel (pentane/ethyl acetate = 3/1) to afford the title compound (9.1 mg, 0.009 mmol, 53%) as a colourless oil.

$[\alpha]_D^{25}$ +18.8 (c = 0.8, $CHCl_3$); **R$_f$** 0.35 (pentane/ethyl acetate = 1/1); **IR** (thin film): ν_{max}/cm^{-1} 3459, 2928, 2855, 1713, 1518, 1462, 1376, 1249, 1193, 1172, 1099, 1033, 986, 835, 776; **^{1}H NMR** (400 MHz, $CDCl_3$): δ 7.43 (d, *J* = 8.7 Hz, 2H, Ar*H*), 6.87 (d, *J* = 8.7 Hz, 2H, Ar*H*), 6.37 (dd, *J* = 15.0, 11.0 Hz, 1H, *H12*), 6.14 (d, *J* = 11.0 Hz, 1H, *H11*), 5.72 (dt, *J* = 15.0, 7.2 Hz, 1H, *H13*), 5.47 (s, 1H, C*H*PMP), 5.12 (d, *J* = 9.7 Hz, 1H, *H25*), 4.74 (d, *J* = 1.9 Hz, 1H, O*H*), 4.25 – 4.17 (m, 3H, $CO_2CH_2CH_3$ and *H23*), 4.16 – 4.07 (m, 2H, *H7* and *H9*), 3.86 (td, *J* = 7.2, 2.1 Hz, 1H, *H15*), 3.82 – 3.74 (m, 3H, *H5*, *H17*, *H21*), 3.79 (s, 3H, OCH_3), 3.48 (dd, *J* = 9.8, 5.7 Hz, 1H, *H27*), 3.42 (dd, *J* = 9.8, 7.0 Hz, 1H, *H27'*), 2.79 (d, *J* = 15.3 Hz, 1H, *H2*), 2.54 – 2.46 (m, 2H, *H2'* and *H14*), 2.45 – 2.38 (m, 1H, *H26*), 2.31 (dt, *J* = 14.4, 7.2 Hz, 1H, *H14'*), 1.87 – 1.78 (m, 2H, *H6* and *H22*), 1.70 – 1.35 (m, 10H, *H6'*, *H8*, *H16*, *H18*, *H19*, *H20, H22'*), 1.67 (s, 3H, CCH_3), 1.64 (d, *J* = 1.0 Hz, 3H, CCH_3), 1.36 (s, 3H, acetonide-CH_3), 1.35 (s, 3H, acetonide-CH_3), 1.34 – 1.31 (m, 1H, *H4*), 1.30 (t, *J* = 7.1 Hz, 3H, $CO_2CH_2CH_3$), 1.58 – 1.09 (m, 10H, *n*-Hex-$(CH_2)_5$), 1.10 (d, *J* = 6.6 Hz, 3H, $CHCH_3$), 0.97 (d, *J* = 6.8 Hz, 3H, $CHCH_3$), 0.89 – 0.85 (m, 15H, $CHCH_3$, *n*-Hex-CH_3 and $OSiC(CH_3)_3$), 0.02 (s, 3H, $OSiCH_3$), 0.02 (s, 3H, $OSiCH_3$); **^{13}C NMR** (101 MHz, $CDCl_3$): δ 172.8, 160.0, 136.1, 136.0, 131.8, 129.2, 128.7, 128.0, 127.6, 125.0, 113.7, 101.6, 100.4, 99.0, 81.2, 81.1, 79.5, 71.9, 71.7, 69.7, 66.7, 66.7, 61.4, 55.5, 46.8, 42.6, 40.5, 39.5, 38.7, 37.1, 36.4, 36.1, 34.1, 32.6, 32.0, 31.8, 29.7, 27.2, 26.1, 25.2, 25.0, 22.8, 21.3, 18.5, 14.3, 14.3, 13.8, 12.8, 12.1, 6.8, 5.8, –5.1, –5.2; **HRMS** (ESI) m/z calcd for $C_{57}H_{96}O_{12}Si$ $[M+Na]^+$: 1023.6587, found 1023.6552.

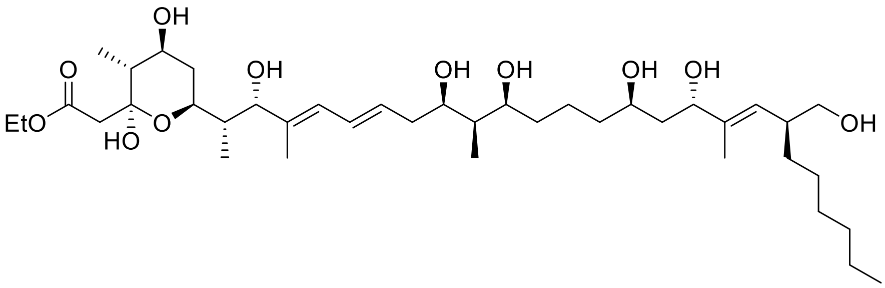

Ethyl 2-((2*S*,3*R*,4*S*,6*S*)-2,4-dihydroxy-3-methyl-6-((2*S*,3*S*,4*E*,6*E*,9*R*,10*R*,11*S*,15*R*,17*S*,18*E*,20*S*)-3,9,11,15,17-pentahydroxy-20-(hydroxymethyl)-4,10,18-trimethylhexacosa-4,6,18-trien-2-yl)tetrahydro-2*H*-pyran-2-yl)acetate (1). To a solution of **28** (18.0 mg, 0.018 mmol, 1.0 eq.) in THF (1 mL) and MeOH (2 mL) was added 0.1 M aqueous HCl (2 mL). The reaction mixture was stirred at room temperature for 3 hours. It was diluted with chloroform, quenched with saturated aqueous $NaHCO_3$ and extracted with chloroform. The organic layers

were washed with brine, dried over Na_2SO_4 and concentrated under reduced pressure. The residue was purified by flash chromatography on silica gel (CH_2Cl_2/MeOH = 24/1 → CH_2Cl_2/MeOH = 47/3) to afford the title compound (5.4 mg, 0.007 mmol, 41%) as a colourless oil.

$[\alpha]_D^{25}$ +7.4 (c = 0.4, $CHCl_3$); **R_f** 0.14 (CH_2Cl_2/MeOH = 9/1); **IR** (thin film): ν_{max}/cm^{-1} 3352, 2924, 2855, 1713, 1459, 1375, 1331, 1193, 1019, 986, 756; **^{1}H NMR** (600 MHz, MeOD): δ 6.37 (dd, *J* = 15.0, 10.9 Hz, 1H, *H12*), 6.08 (d, *J* = 10.9 Hz, 1H, *H11*), 5.77 (dt, *J* = 15.0, 7.3 Hz, 1H, *H13*), 5.15 (d, *J* = 9.8 Hz, 1H, *H25*), 4.24 – 4.15 (m, 3H, *H23* and $CO_2CH_2CH_3$), 3.93 (d, *J* = 7.0 Hz, 1H, *H9*), 3.84 (dt, *J* = 12.2, 2.7 Hz, 1H, *H7*), 3.78 (td, *J* = 6.5, 3.7 Hz, 1H, *H15*), 3.75 – 3.71 (m, 2H, *H17* and *H21*), 3.60 (td, *J* = 10.7, 4.6 Hz, 1H, *H5*), 3.43 (dd, *J* = 10.7, 6.3 Hz, 1H, *H27*), 3.38 (dd, *J* = 10.7, 6.8 Hz, 1H, *H27'*), 2.74 (d, *J* = 14.3 Hz, 1H, *H2*), 2.55 (d, *J* = 14.3 Hz, 1H, *H2'*), 2.51 – 2.44 (m, 1H, *H26*), 2.34 (app t, *J* = 6.9 Hz, 2H, *H14*), 1.76 – 1.73 (m, 1H, *H6*), 1.71 – 1.66 (m, 1H, H*22*), 1.67 (s, 3H, CCH_3), 1.66 (s, 3H, CCH_3), 1.63 – 1.33 (m, 11H, *H4, H6', H8, H16, H18, H19, H20* and *H22'*), 1.53 – 1.09 (m, 13H, $CO_2CH_2CH_3$ and *n*-Hex-$(CH_2)_5$), 1.06 (d, *J* = 6.5 Hz, 3H, $CHCH_3$), 0.94 (d, *J* = 7.0 Hz, 3H, $CHCH_3$), 0.92 (d, *J* = 6.8 Hz, 3H, $CHCH_3$), 0.90 (t, *J* = 7.1 Hz, 3H, *n*-Hex-CH_3); **^{13}C NMR** (151 MHz, MeOD): δ 173.6, 140.8, 137.3, 131.3, 129.6, 129.1, 127.6, 100.0, 80.1, 75.9, 75.7, 75.4, 70.4, 70.4, 69.2, 67.0, 61.9, 47.3, 44.4, 43.8, 42.5, 41.7, 41.2, 39.7, 39.5, 38.8, 36.1, 33.1, 32.9, 30.6, 28.3, 23.7, 23.3, 14.6, 14.5, 12.5, 12.4, 12.2, 9.2, 7.0; **HRMS** (ESI) m/z calcd for $C_{40}H_{72}O_{11}$ $[M+Na]^+$: 751.4991, found 751.4967.

NMR data comparison of stambomycin D and C1–C27 fragment 1

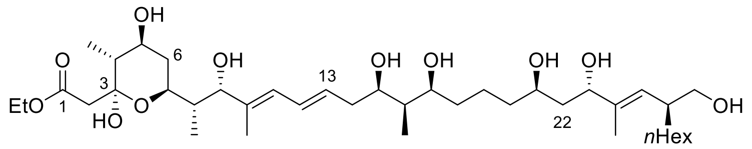

1

	stambomycin D (MeOD, 700 MHz)[20]		C1–C27 fragment 1 (MeOD, 600 MHz)	
Position	δ_C (ppm)	δ_H (ppm)	δ_C (ppm)	δ_H (ppm)
**1**	173.3		173.6	
**2**	44.3	2.52, 2.83	44.4	2.55, 2.74
**3**	100.0		100.0	
**4**	46.2	1.56	47.3	1.40
**4-Me**	12.4	1.04	12.2	1.06
**5**	81.0	3.66	70.4	3.60
**6**	39.1	1.61, 1.90	39.5	1.45, 1.74
**7**	70.0	3.83	70.4	3.84
**8**	40.8	1.56	41.2	1.60
**8-Me**	9.3	0.93	9.2	0.92
**9**	79.9	3.90	80.1	3.93
**10**	137.1		137.3	
**10-Me**	12.4	1.65	12.5	1.67
**11**	128.0	6.06	127.6	6.08
**12**	129.9	6.35	129.6	6.37
**13**	131.7	5.81	131.3	5.77
**14**	39.8	2.34	39.7	2.34
**15**	75.8	3.77	75.9	3.78
**16**	42.6	1.52	42.5	1.53
**16-Me**	7.4	0.95	7.0	0.94
**17**	75.5	3.72	75.7	3.73
**18**	36.1	1.48	36.1	1.48
**19**	23.5	1.32, 1.34	23.3	1.35, 1.60
**20**	38.5	1.47	38.8	1.47
**21**	71.1	3.55	69.2	3.72
**22**	43.1	1.61, 1.67	43.8	1.53, 1.67
**23**	77.5	4.23	75.4	4.21
**24**	139.4		140.8	
**24-Me**	12.2	1.64	12.4	1.66
**25**	129.3	5.23	129.1	5.15
**26**	41.9	2.45	41.7	2.48
**27**	79.7	3.36	67.0	3.38, 3.43

3. ^{1}H and ^{13}C NMR spectra

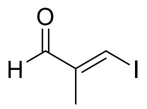

5

^{1}H NMR (400 MHz, CDCl$_3$)

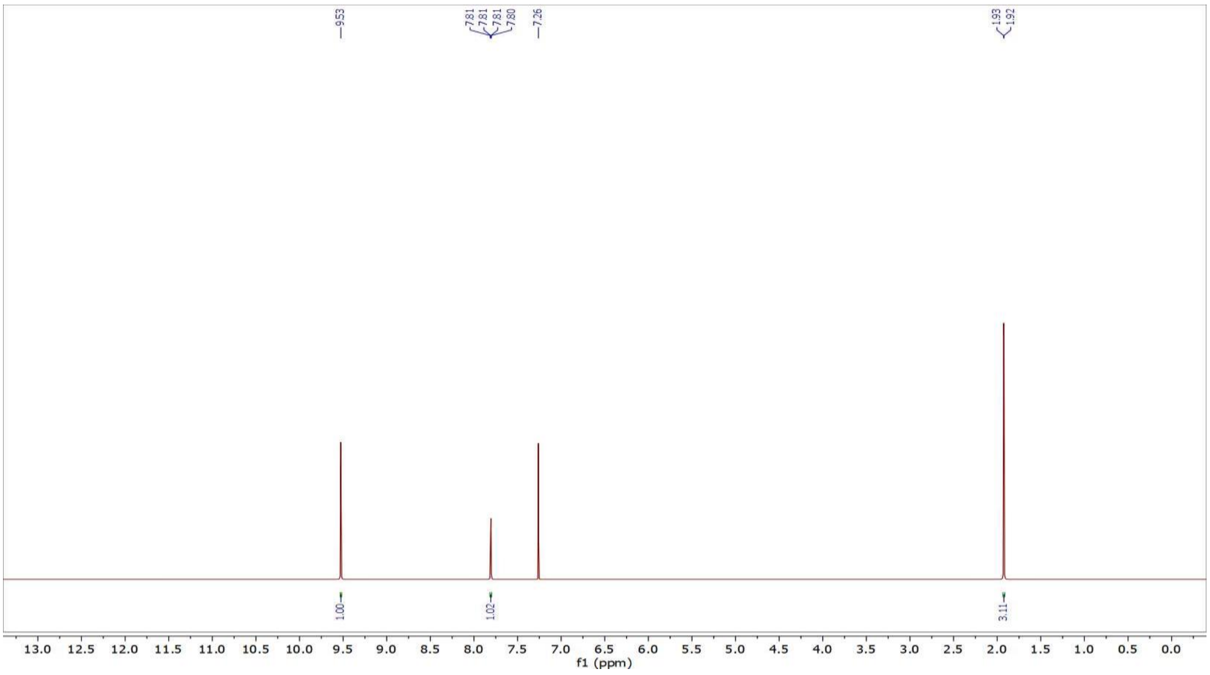

^{13}C NMR (101 MHz, CDCl$_3$)

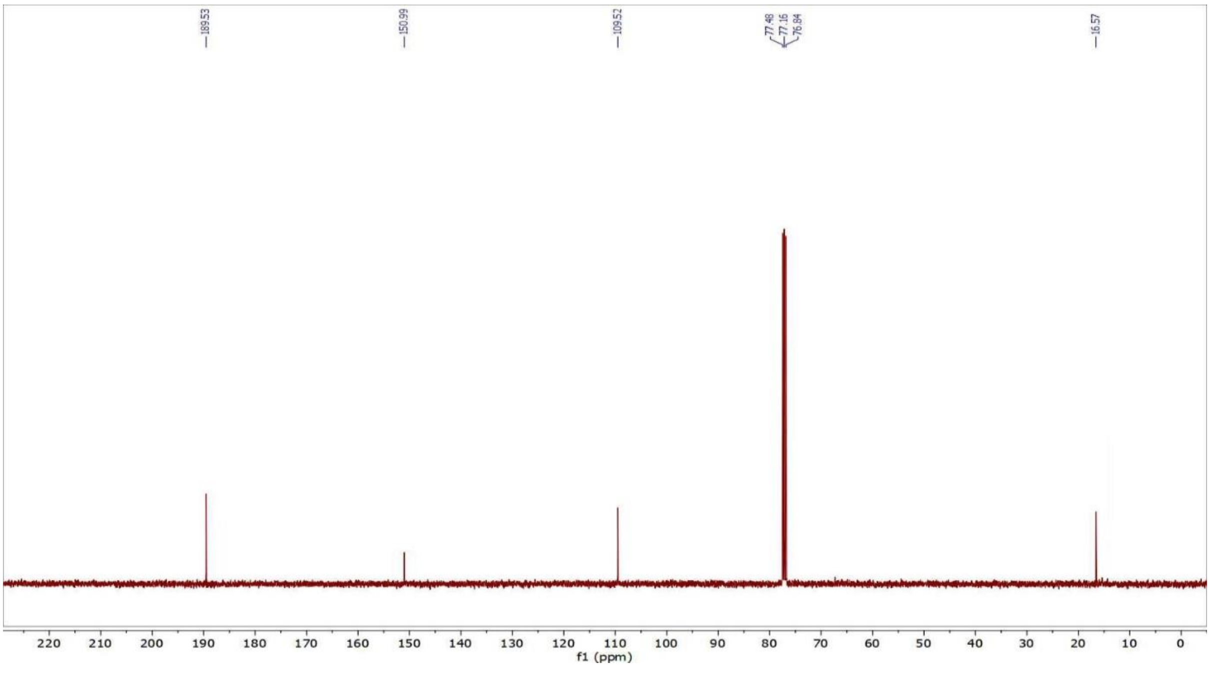

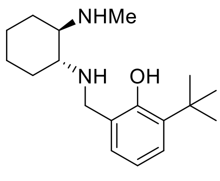

(*R*,*R*)-**6**

^{1}H NMR (400 MHz, CDCl$_3$)

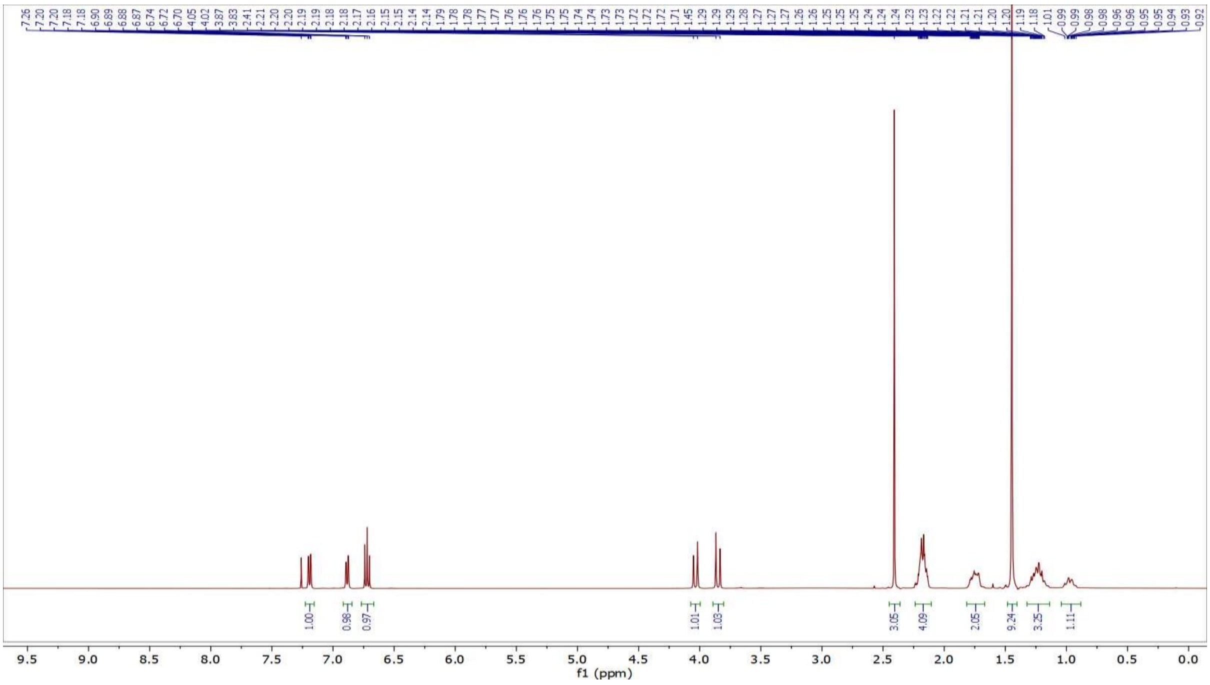

^{13}C NMR (101 MHz, CDCl$_3$)

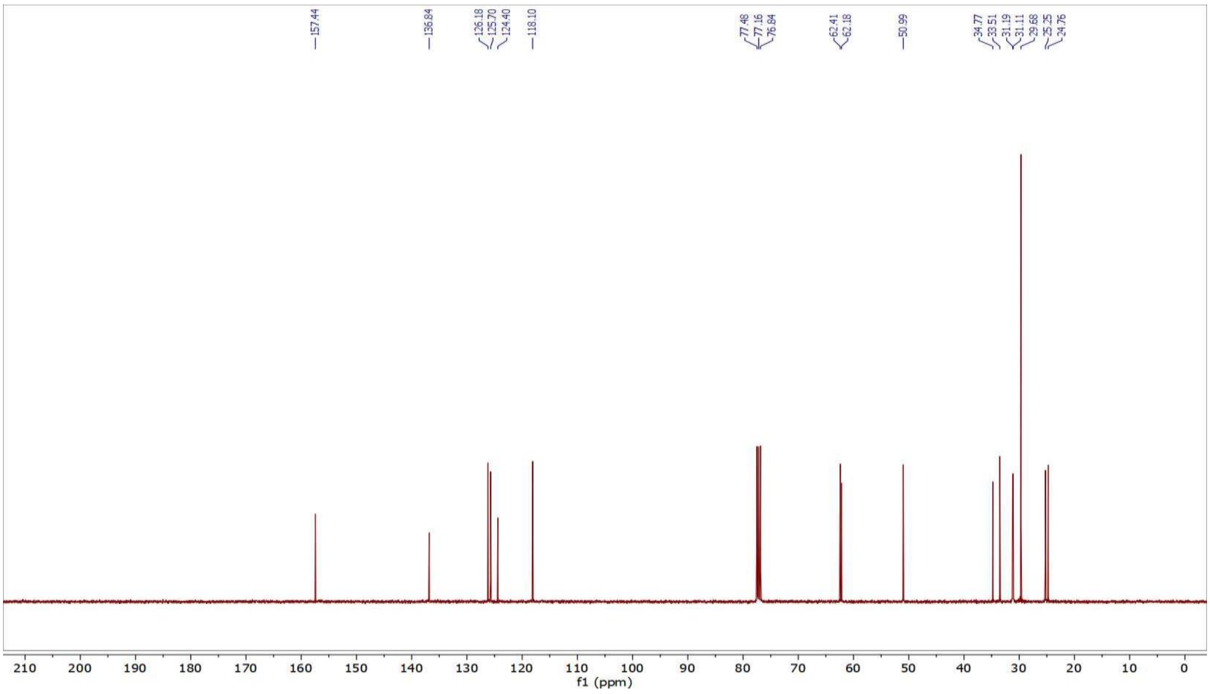

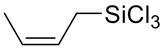

7

^{1}H NMR (400 MHz, $CDCl_3$)

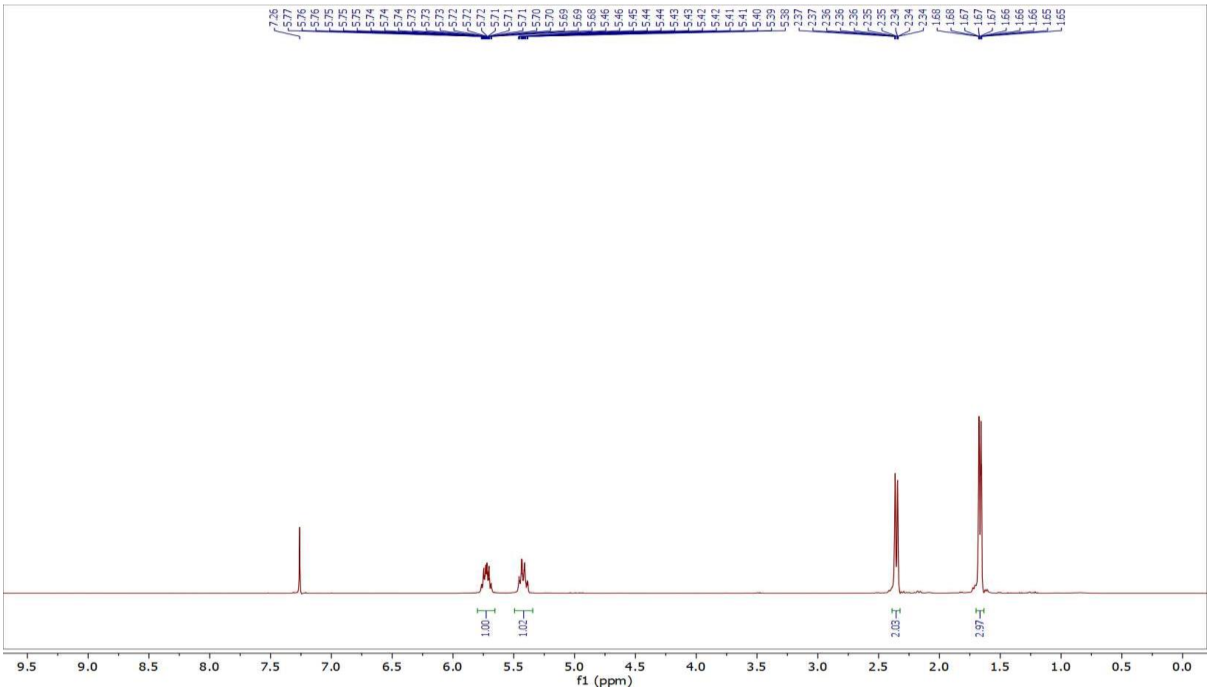

^{13}C NMR (101 MHz, $CDCl_3$)

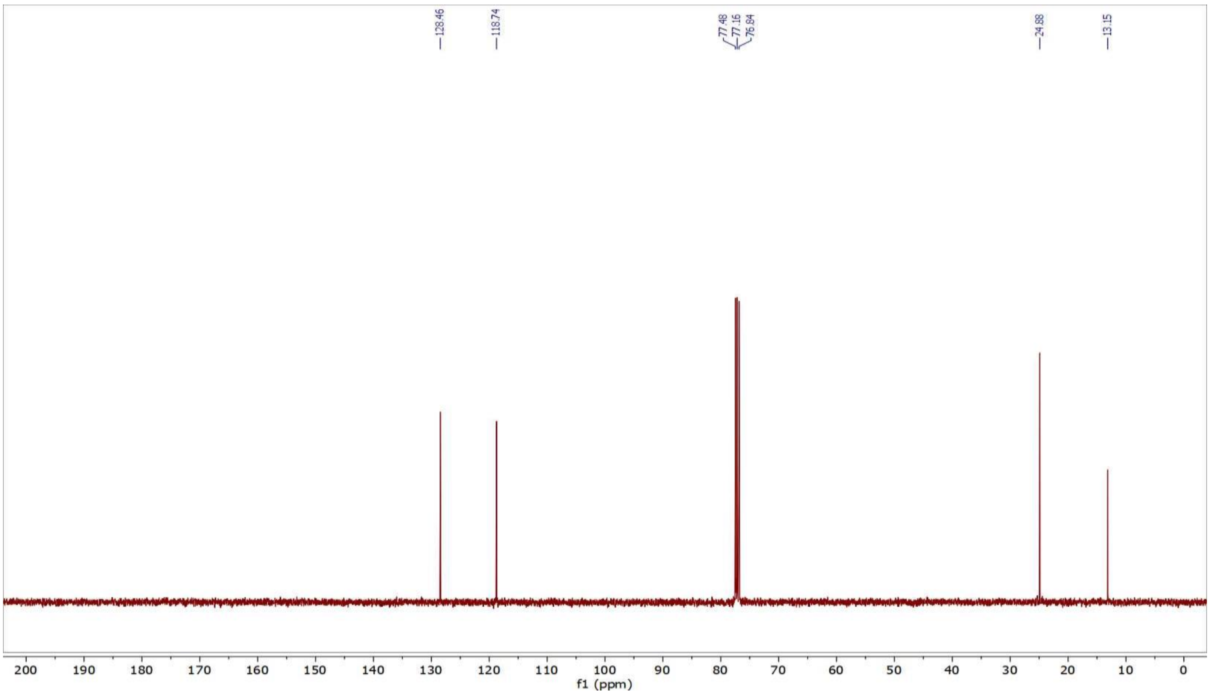

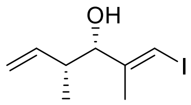

8

^{1}H NMR (400 MHz, CDCl3)

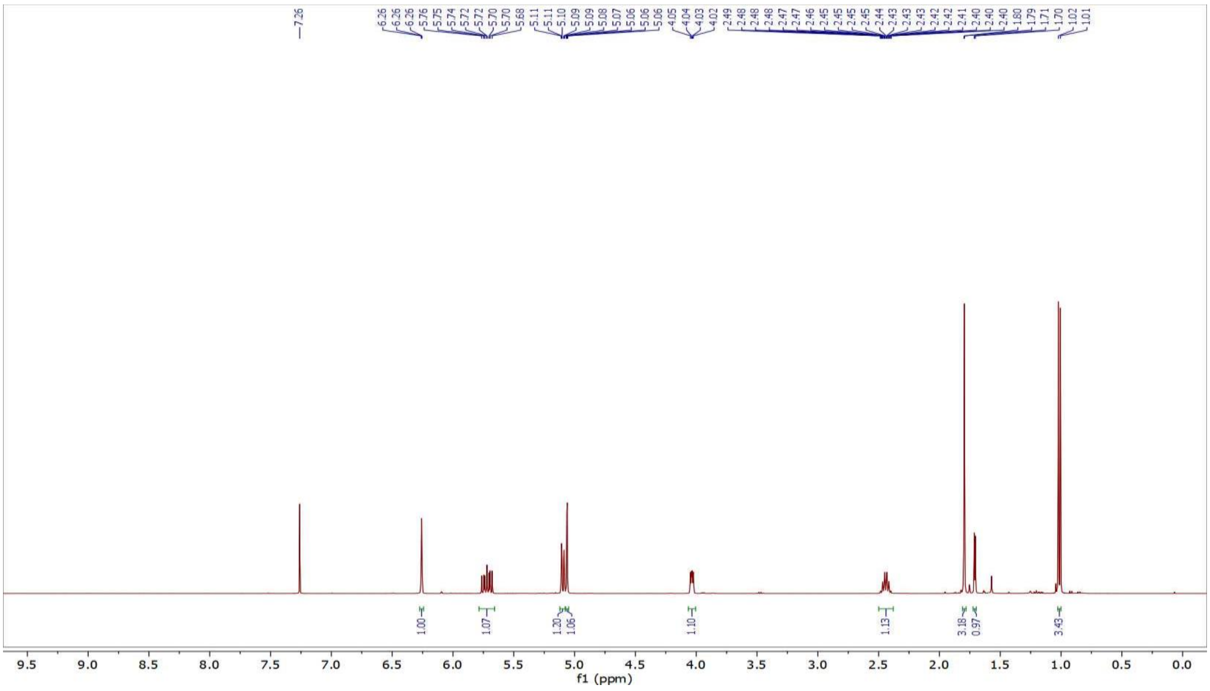

^{13}C NMR (101 MHz, CDCl3)

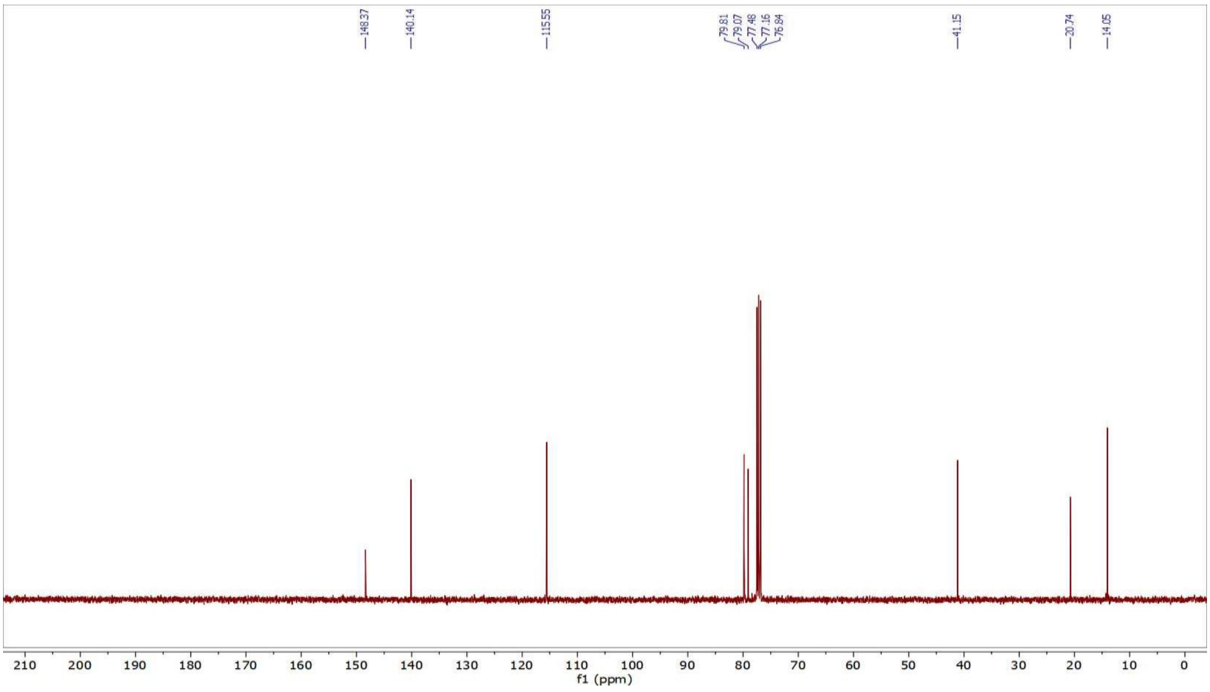

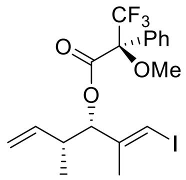

(*S*)-MTPA ester of 8

^{1}H NMR (400 MHz, CDCl$_3$)

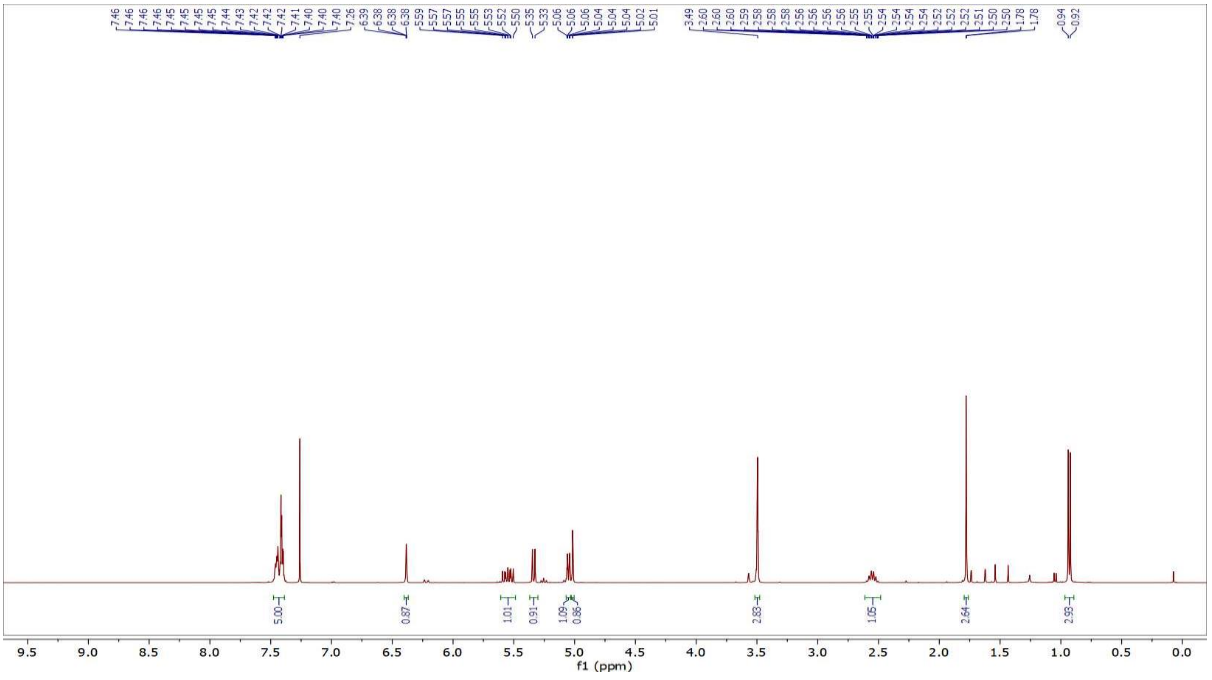

^{13}C NMR (101 MHz, CDCl$_3$)

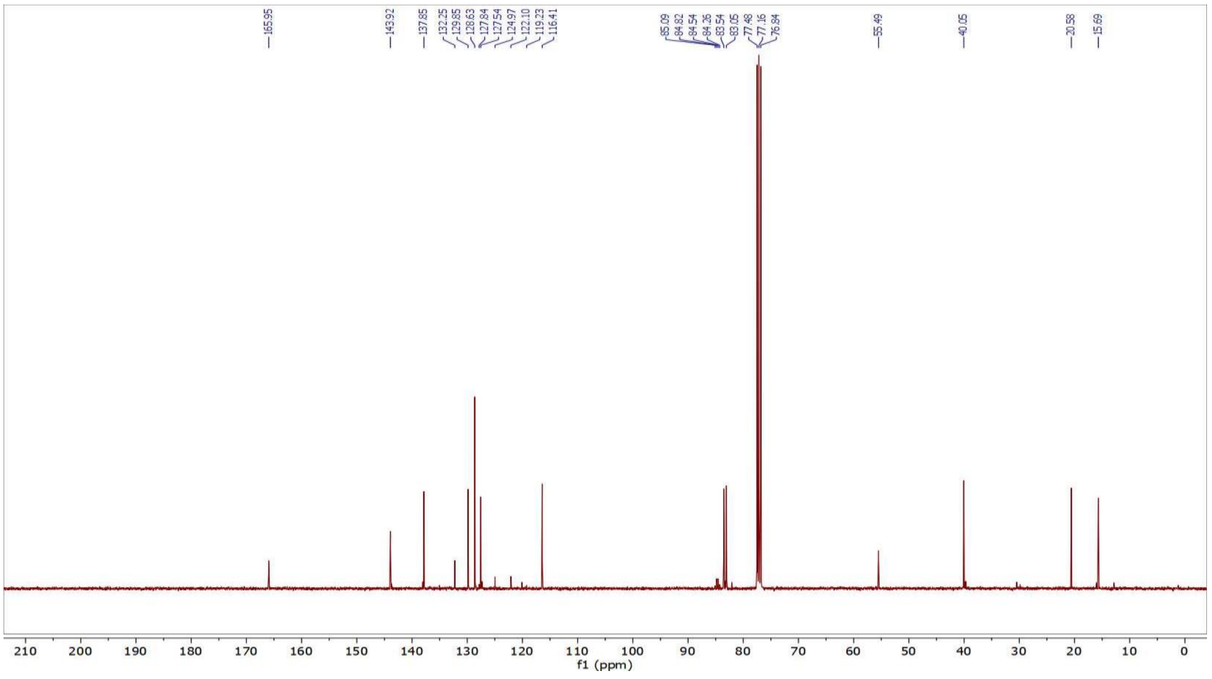

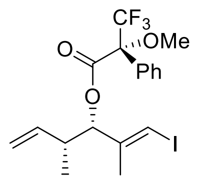

(*R*)-MTPA ester of 8

^{1}H NMR (400 MHz, CDCl$_3$)

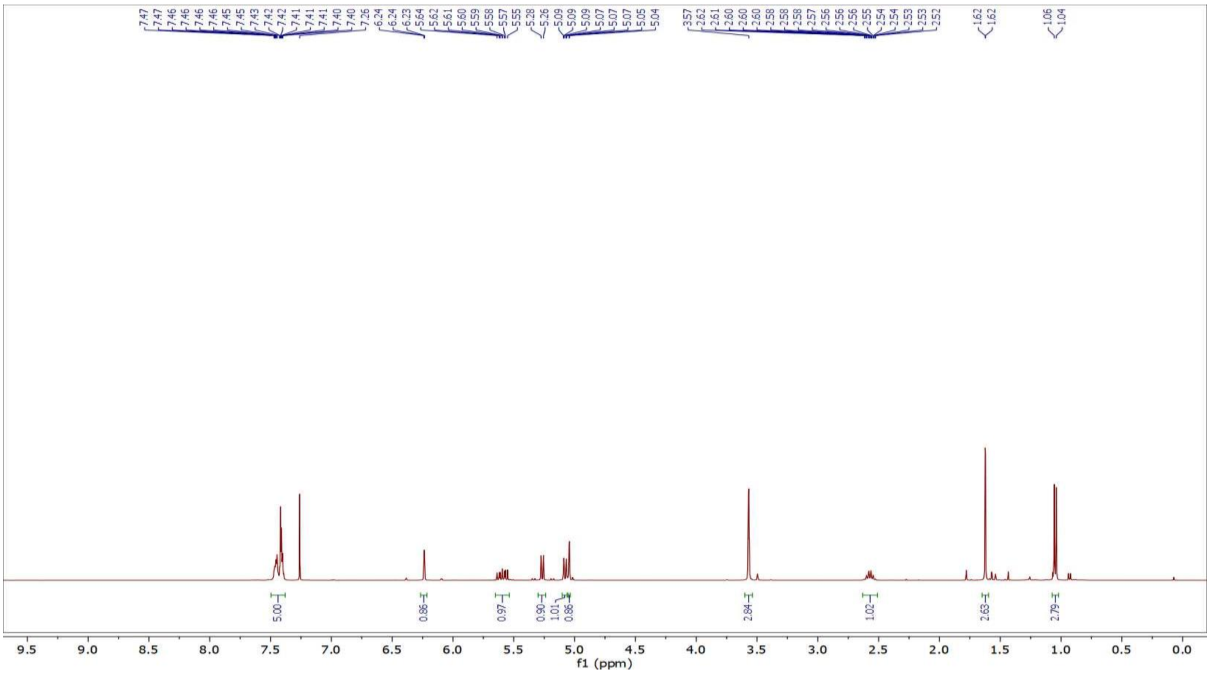

^{13}C NMR (101 MHz, CDCl$_3$)

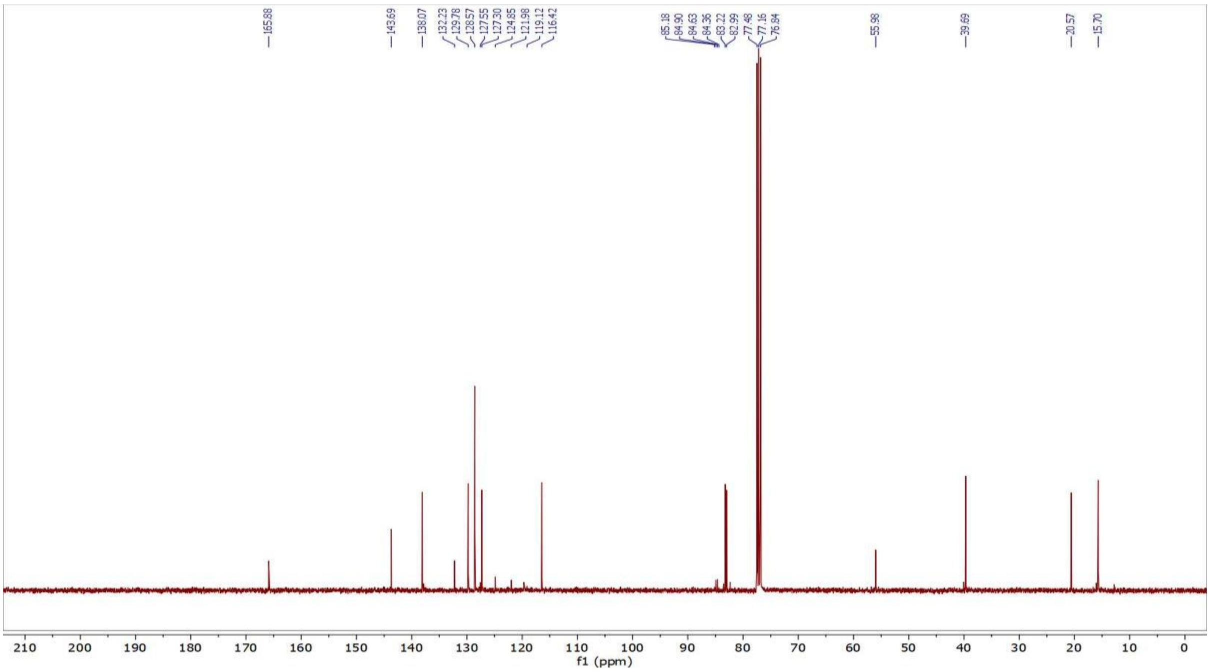

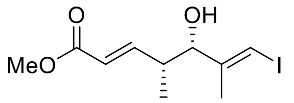

9

^{1}H NMR (400 MHz, $CDCl_3$)

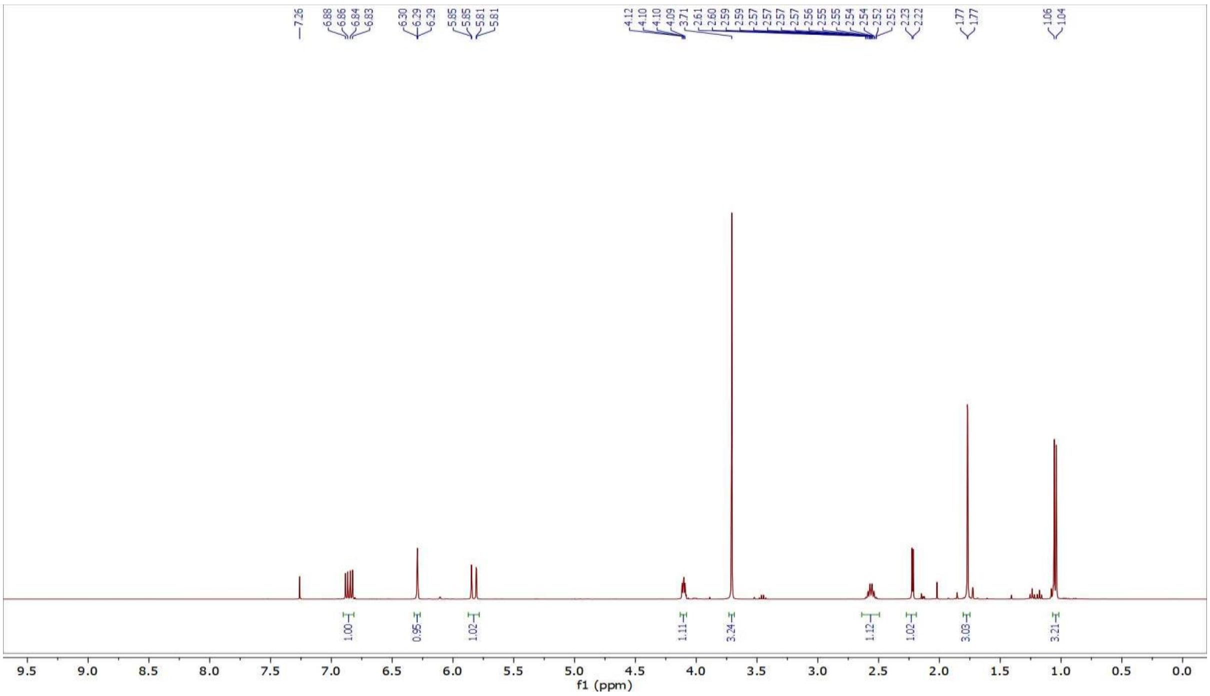

^{13}C NMR (101 MHz, $CDCl_3$)

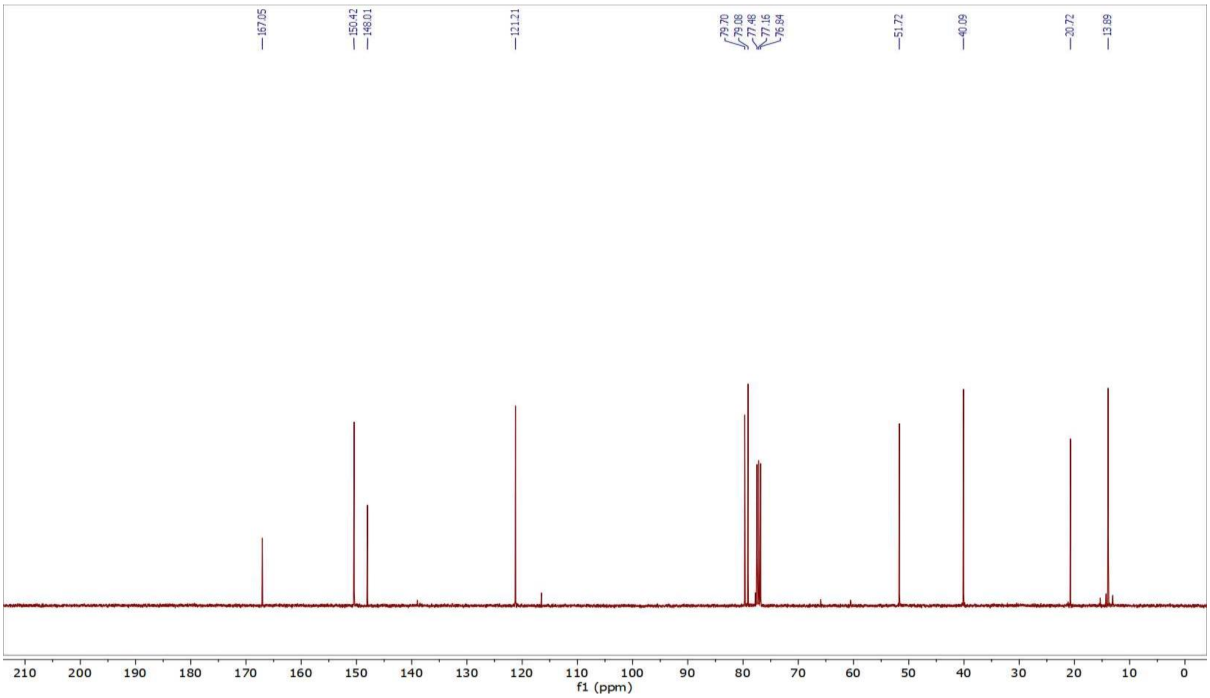

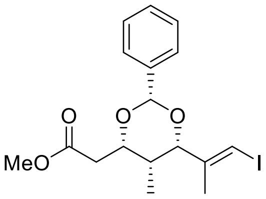

10

^{1}H NMR (400 MHz, CDCl$_3$)

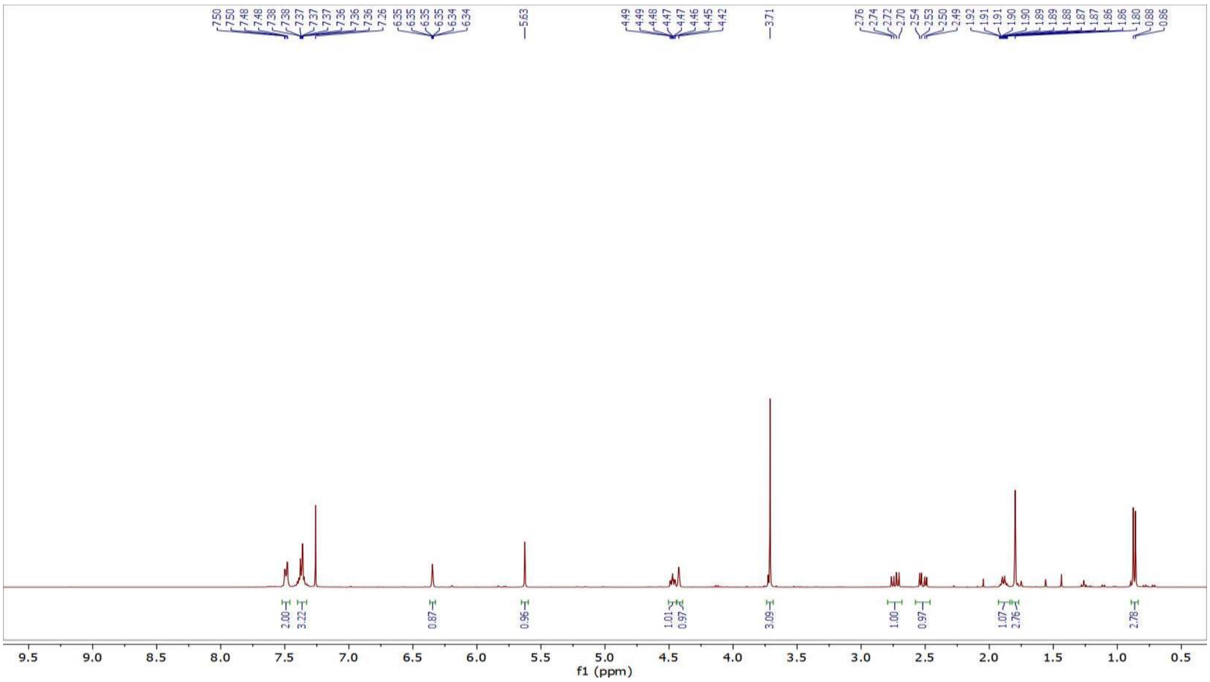

^{13}C NMR (101 MHz, CDCl$_3$)

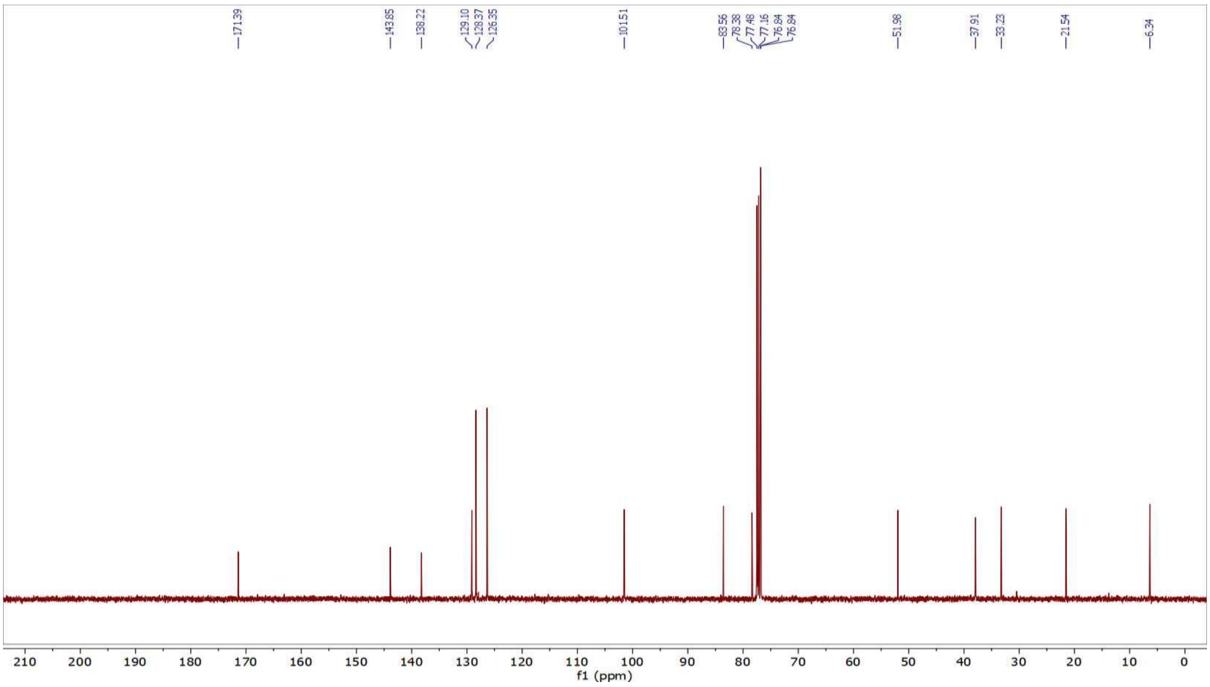

NOESY spectrum of **10**

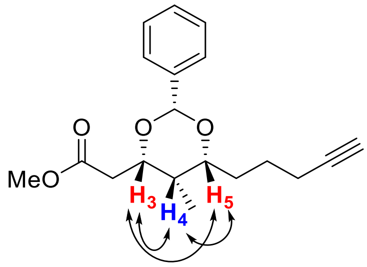

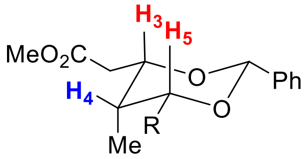

$J_{H3\text{-}H4}$ = 2.3 Hz
$J_{H4\text{-}H5}$ = 2.3 Hz

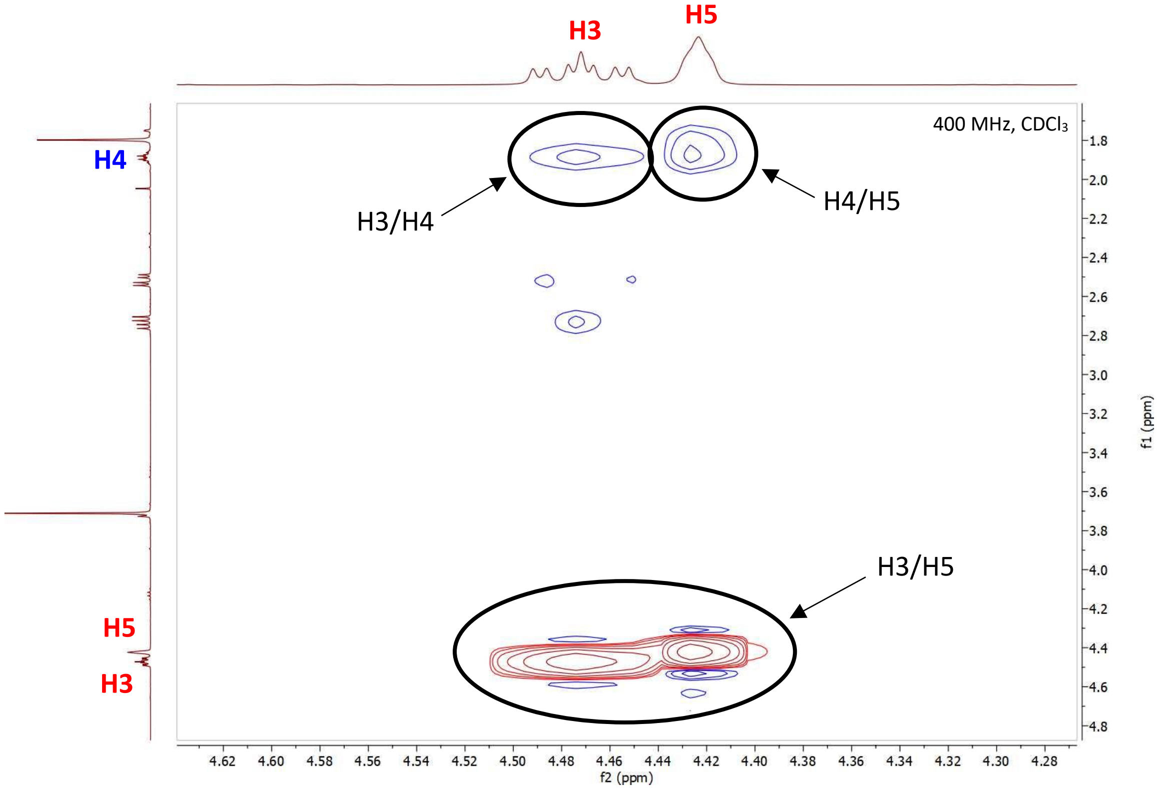

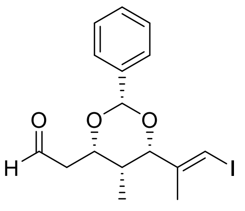

10.1

^{1}H NMR (400 MHz, $CDCl_3$)

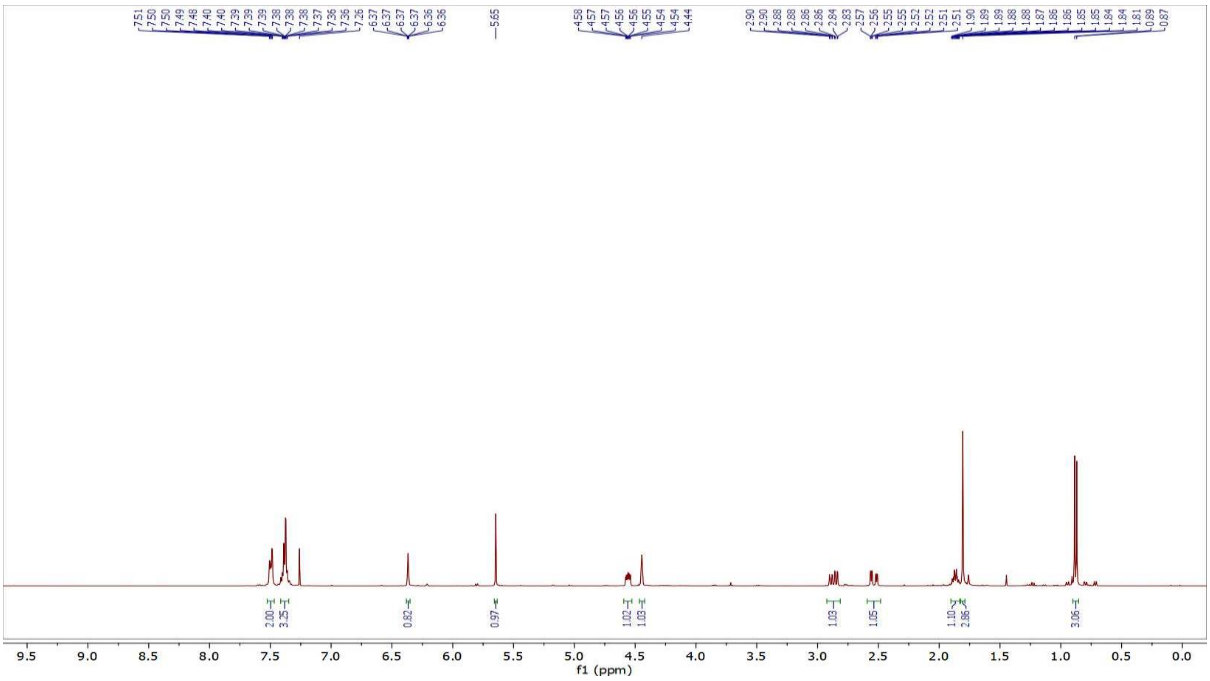

^{13}C NMR (101 MHz, $CDCl_3$)

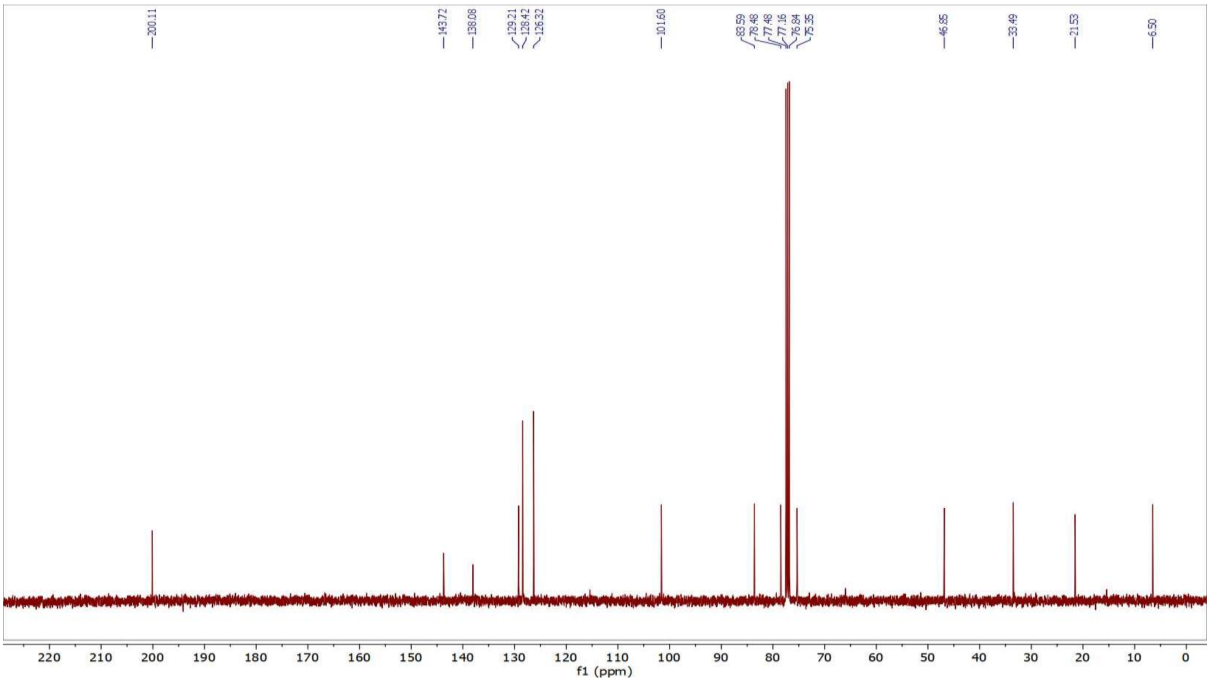

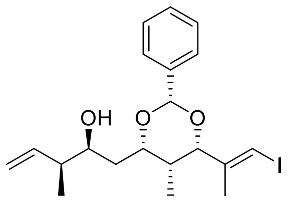

11

^{1}H NMR (400 MHz, $CDCl_3$)

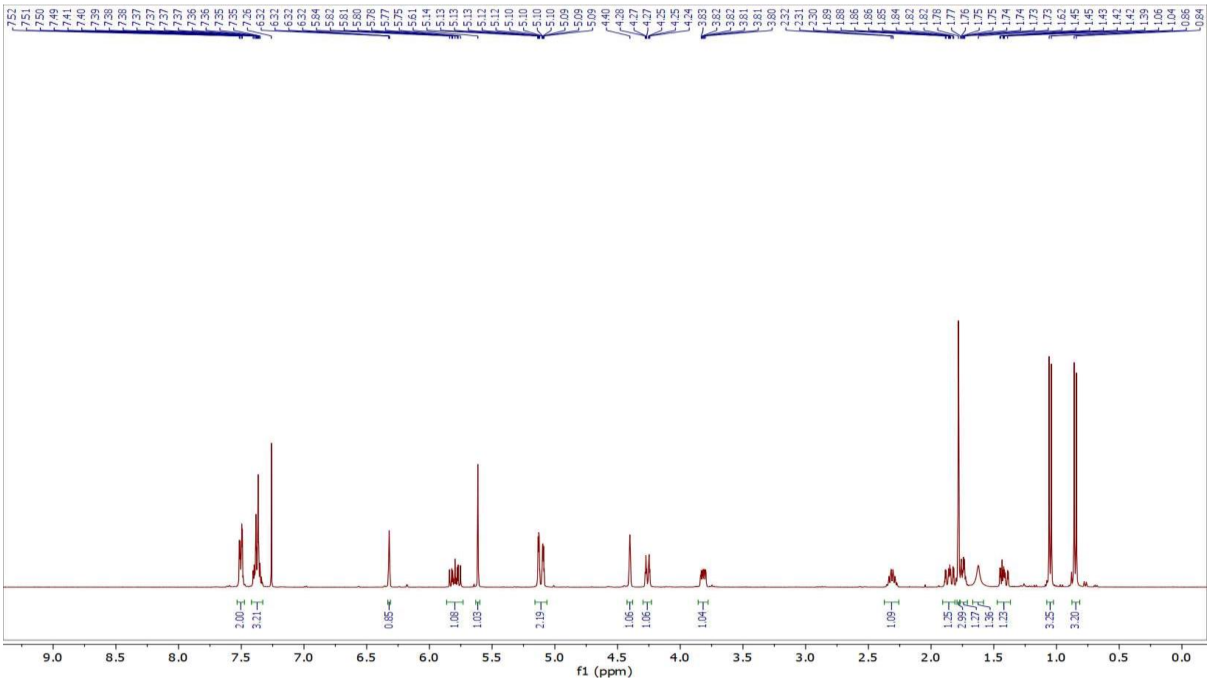

^{13}C NMR (101 MHz, $CDCl_3$)

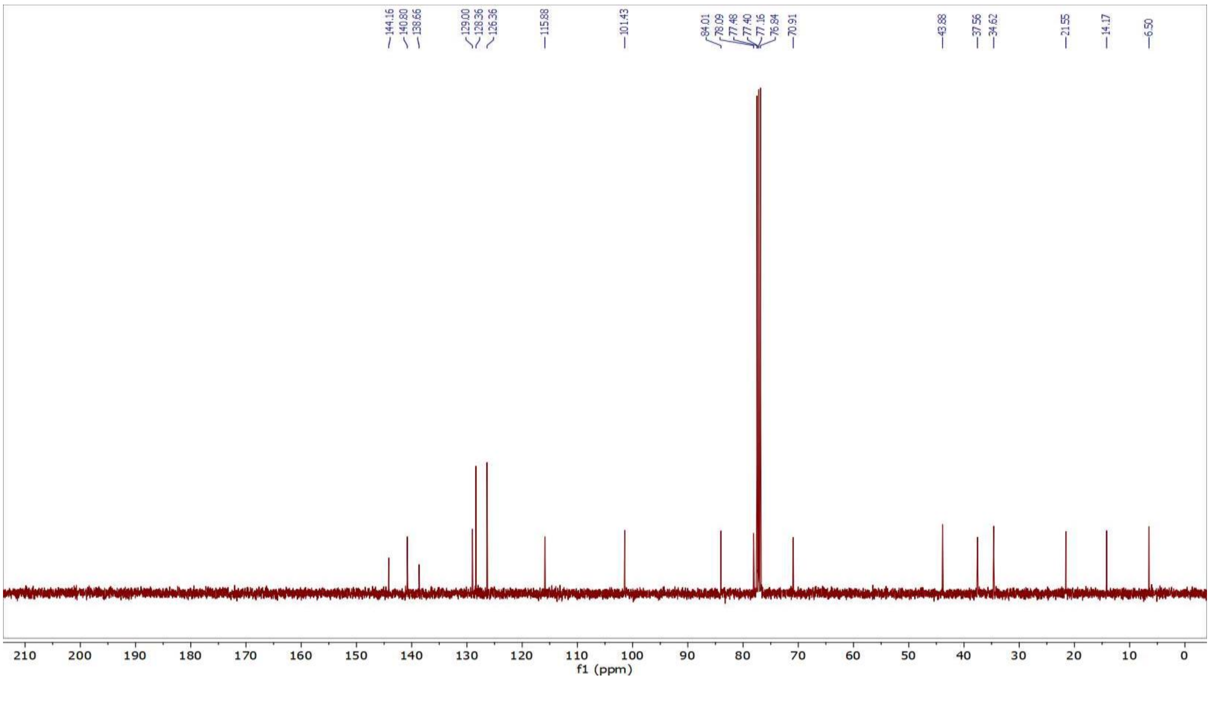

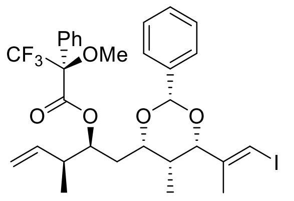

(S)-MTPA ester of 11

^{1}H NMR (400 MHz, CDCl₃)

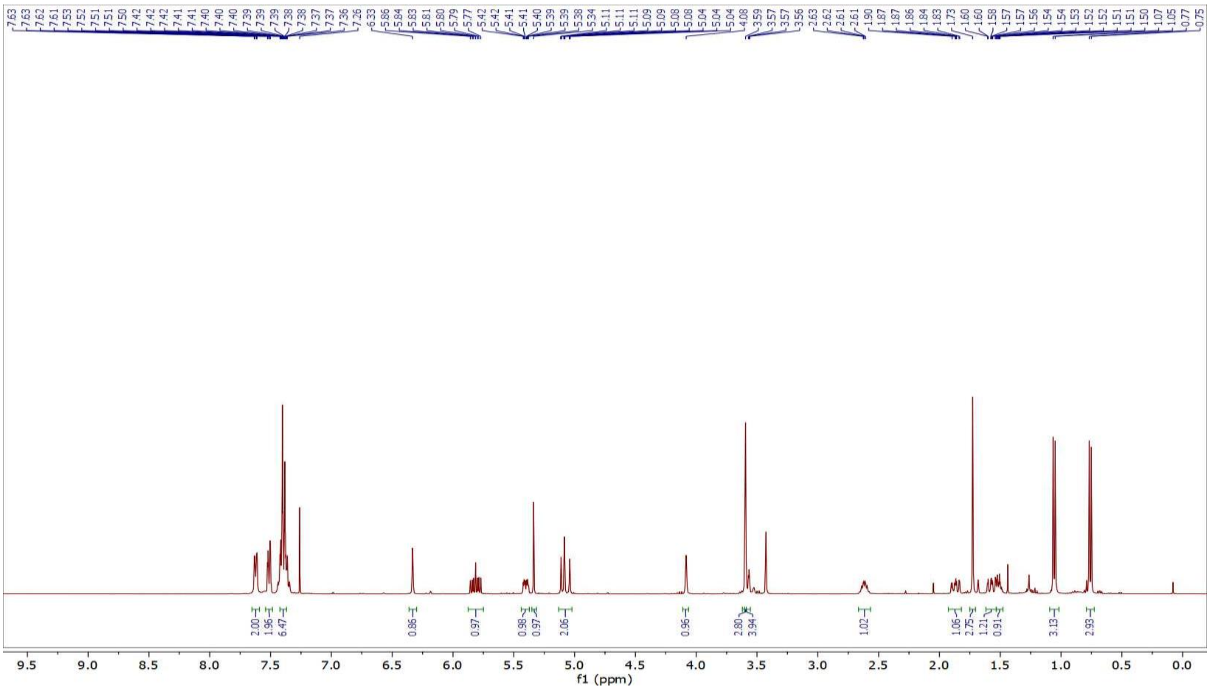

^{13}C NMR (126 MHz, CDCl₃)

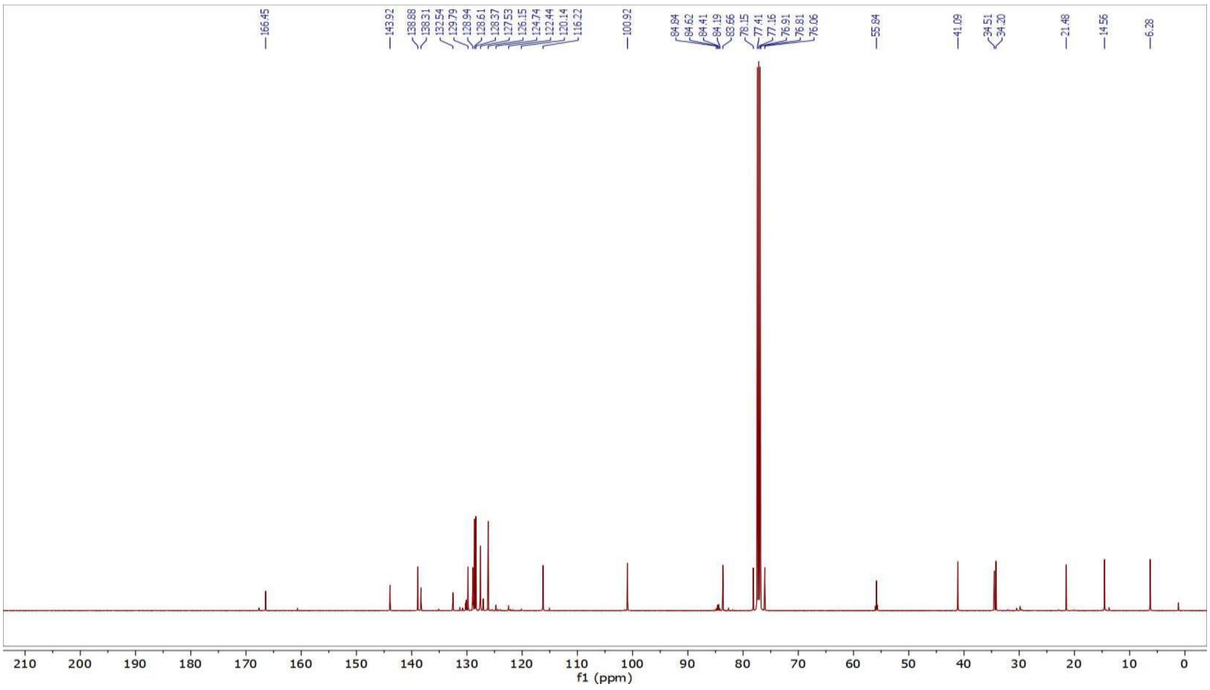

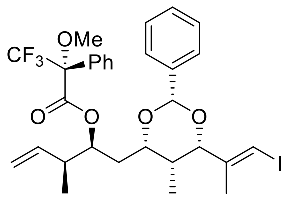

(R)-MTPA ester of 11

^{1}H NMR (400 MHz, $CDCl_3$)

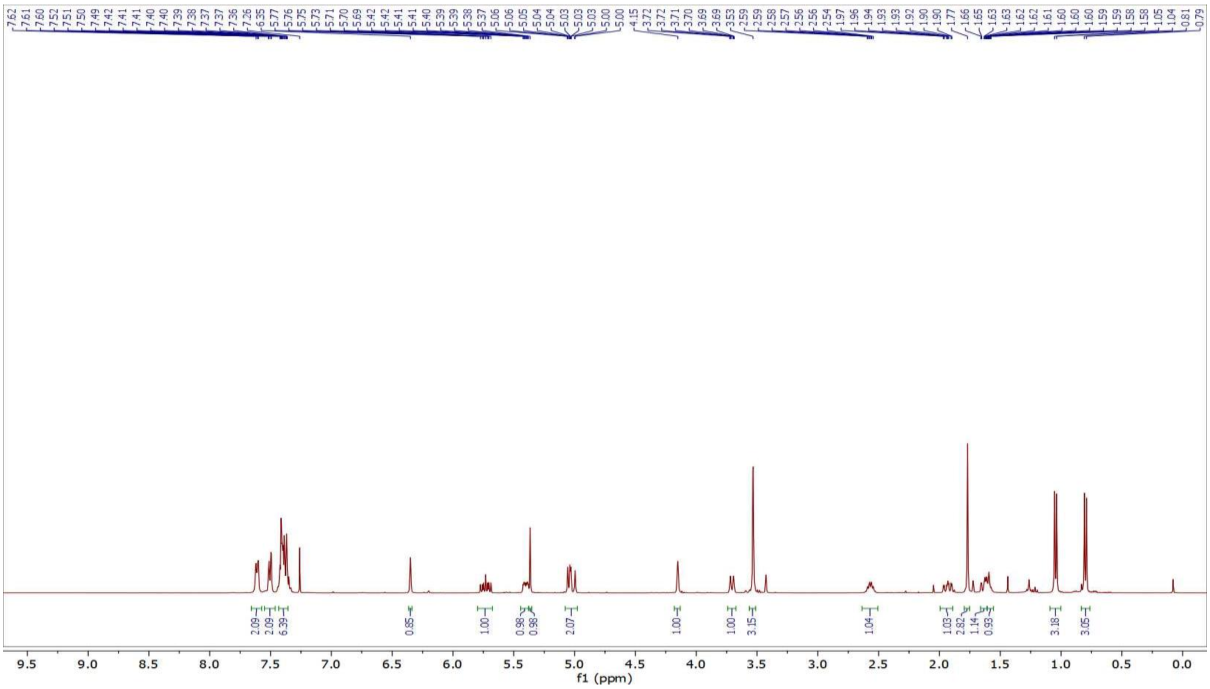

^{13}C NMR (101 MHz, $CDCl_3$)

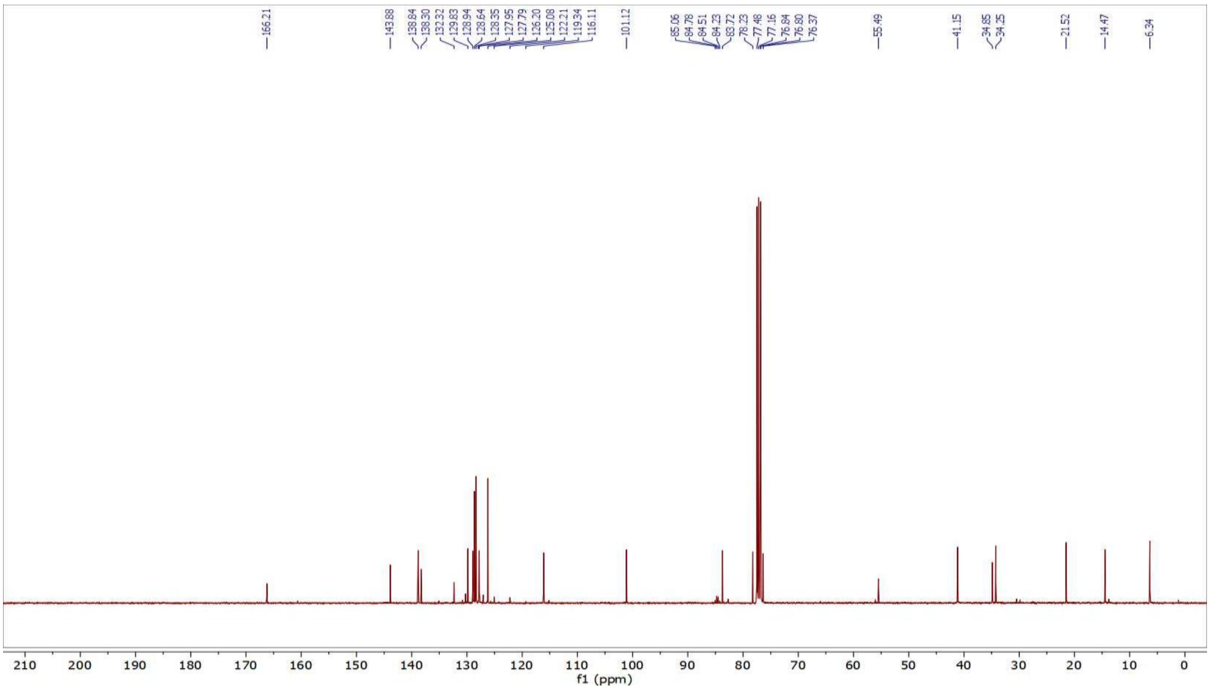

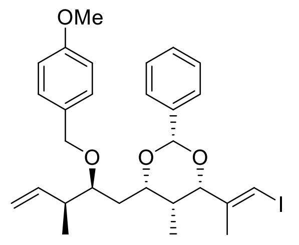

11.1

^{1}H NMR (400 MHz, CDCl$_3$)

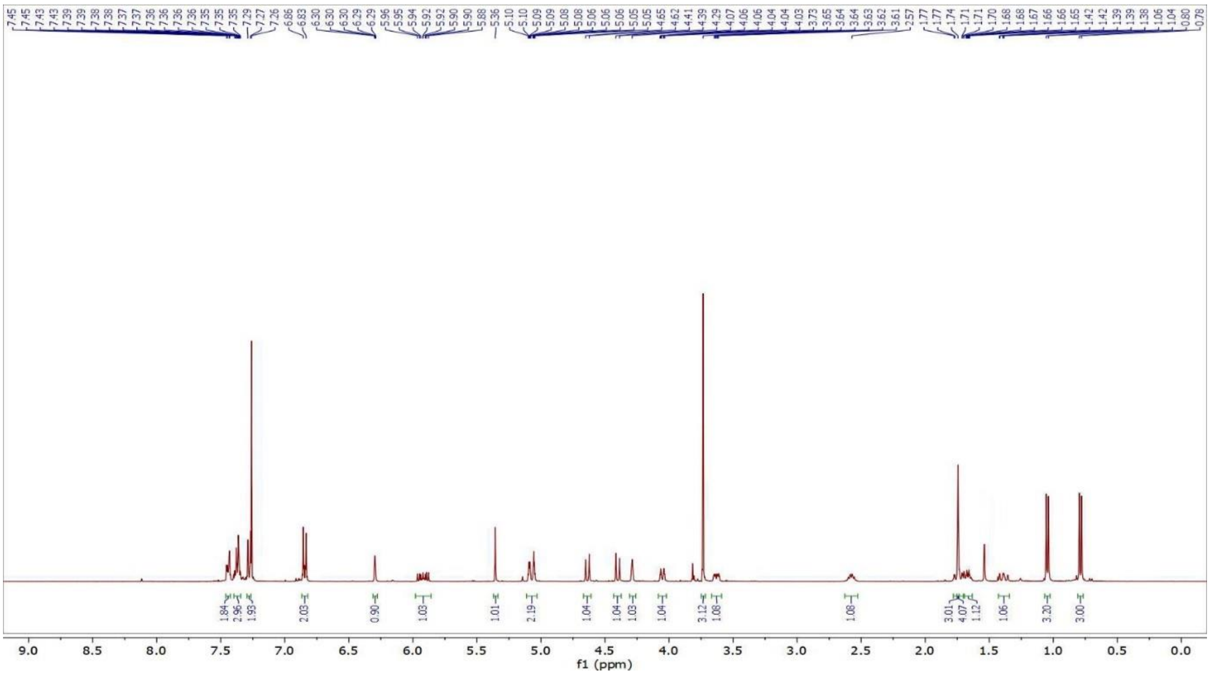

^{13}C NMR (101 MHz, CDCl$_3$)

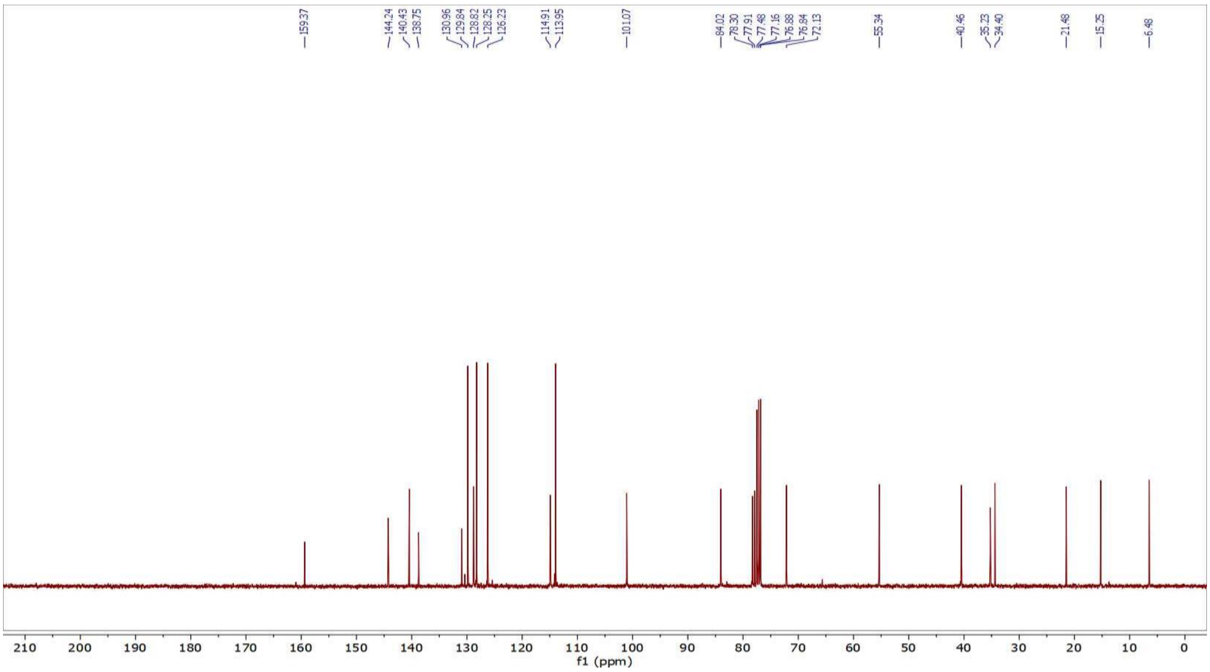

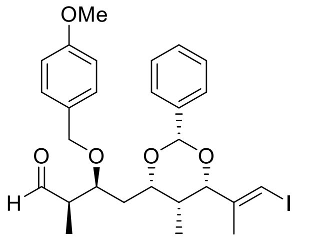

12

^{1}H NMR (400 MHz, CDCl$_3$)

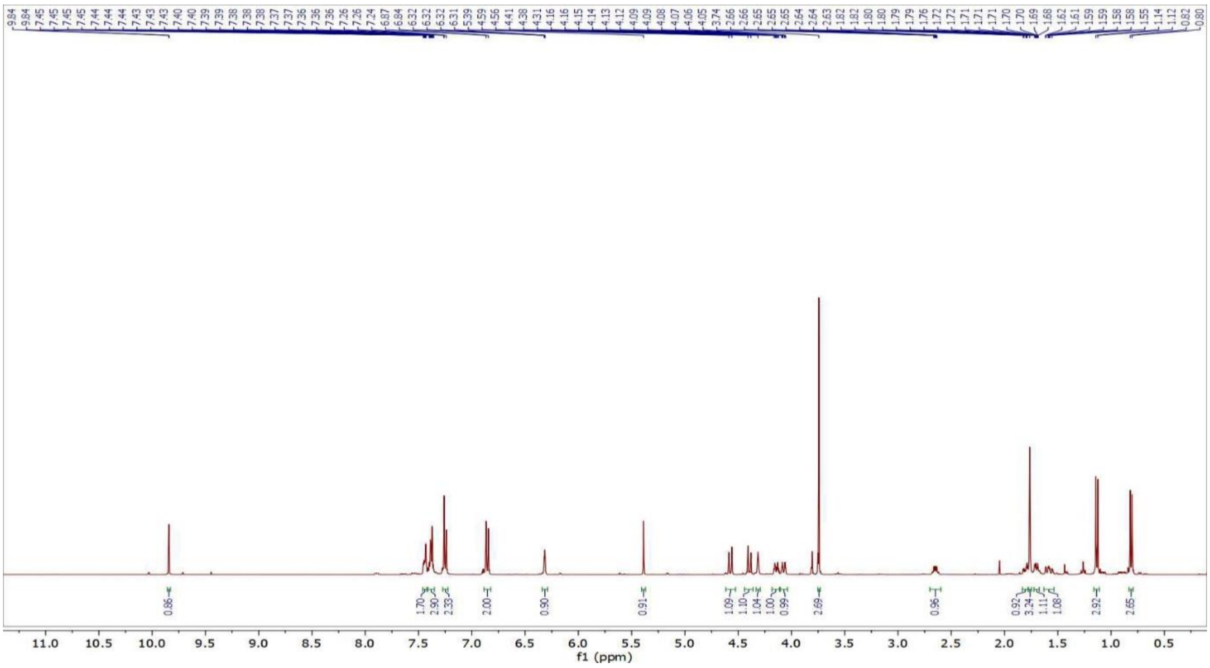

^{13}C NMR (101 MHz, CDCl$_3$)

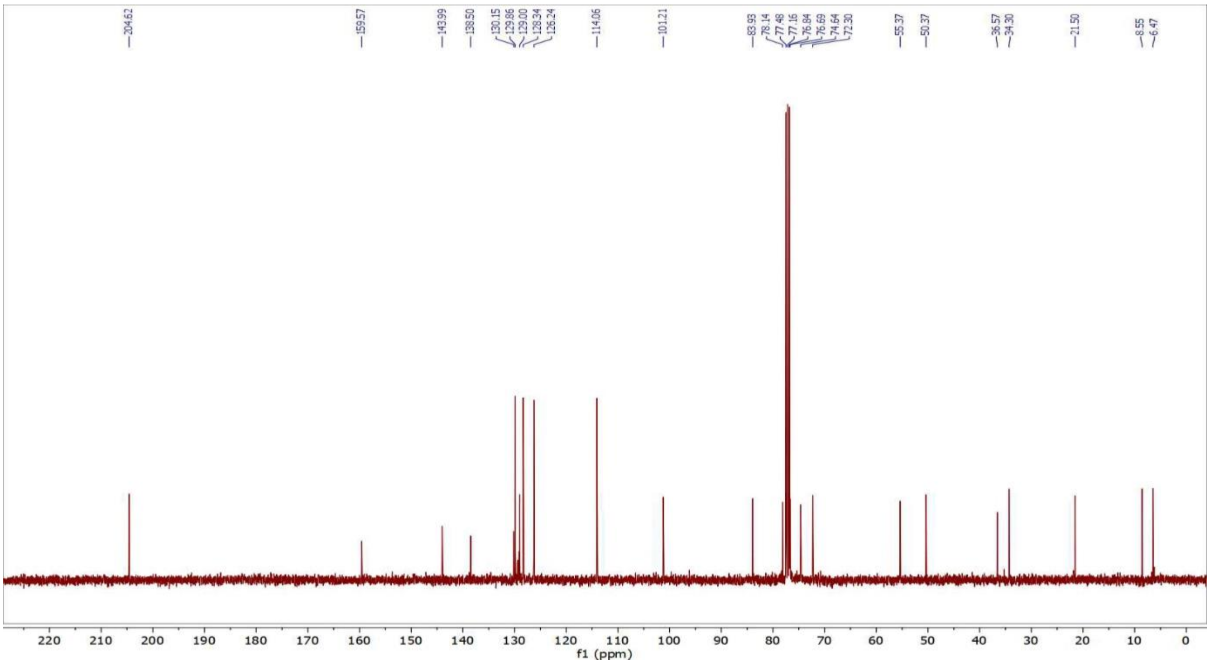

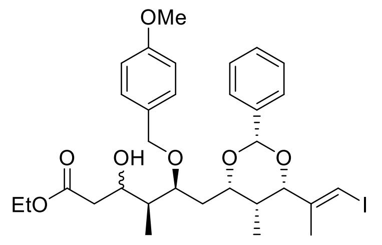

12.1

^{1}H NMR (400 MHz, CDCl$_3$)

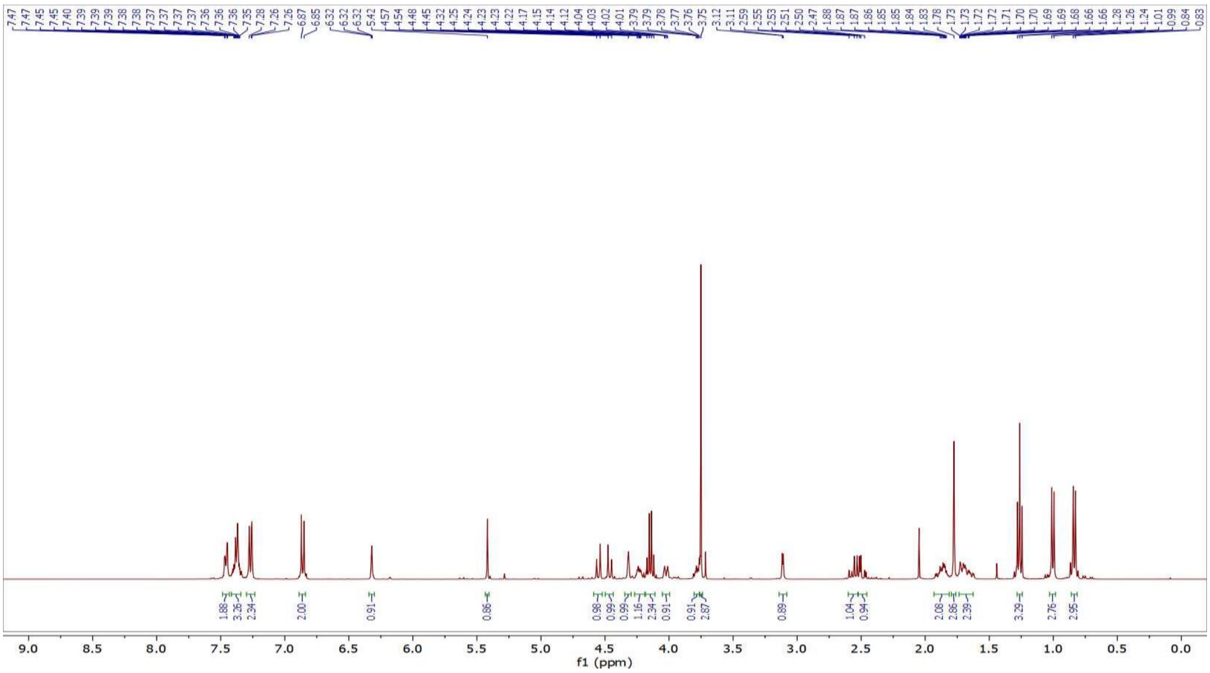

^{13}C NMR (101 MHz, CDCl$_3$)

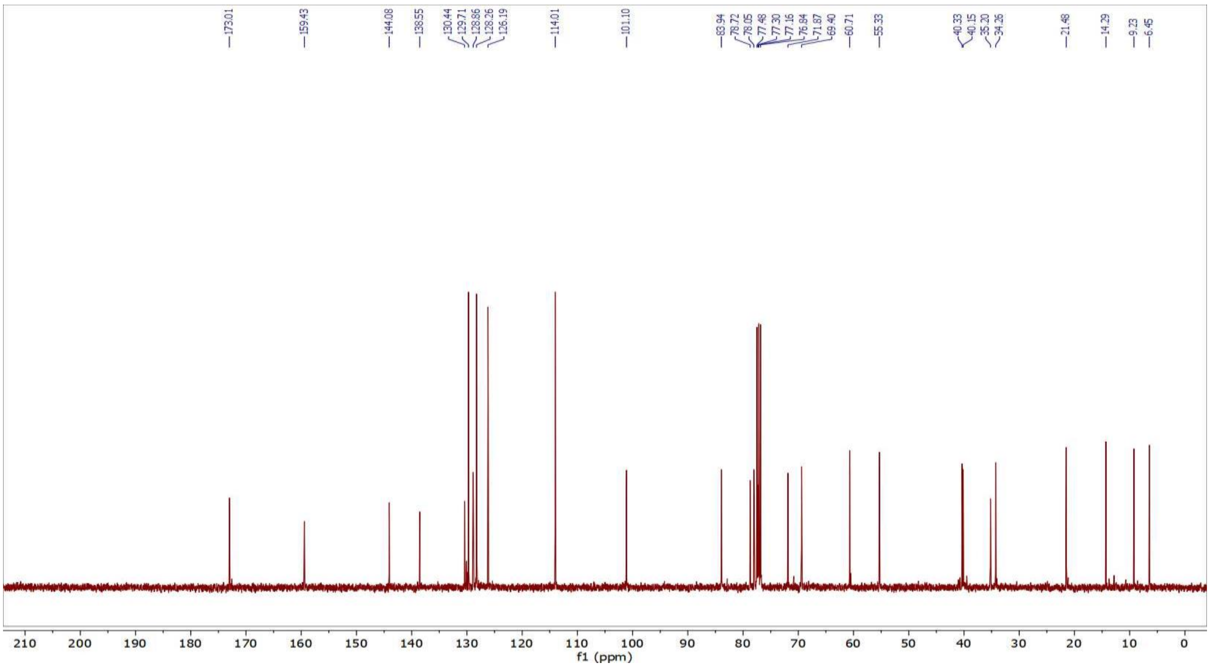

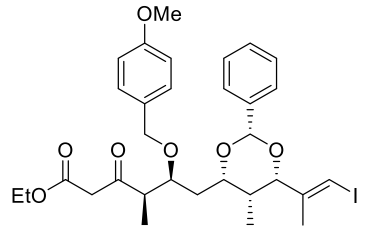

14

^{1}H NMR (400 MHz, CDCl$_3$)

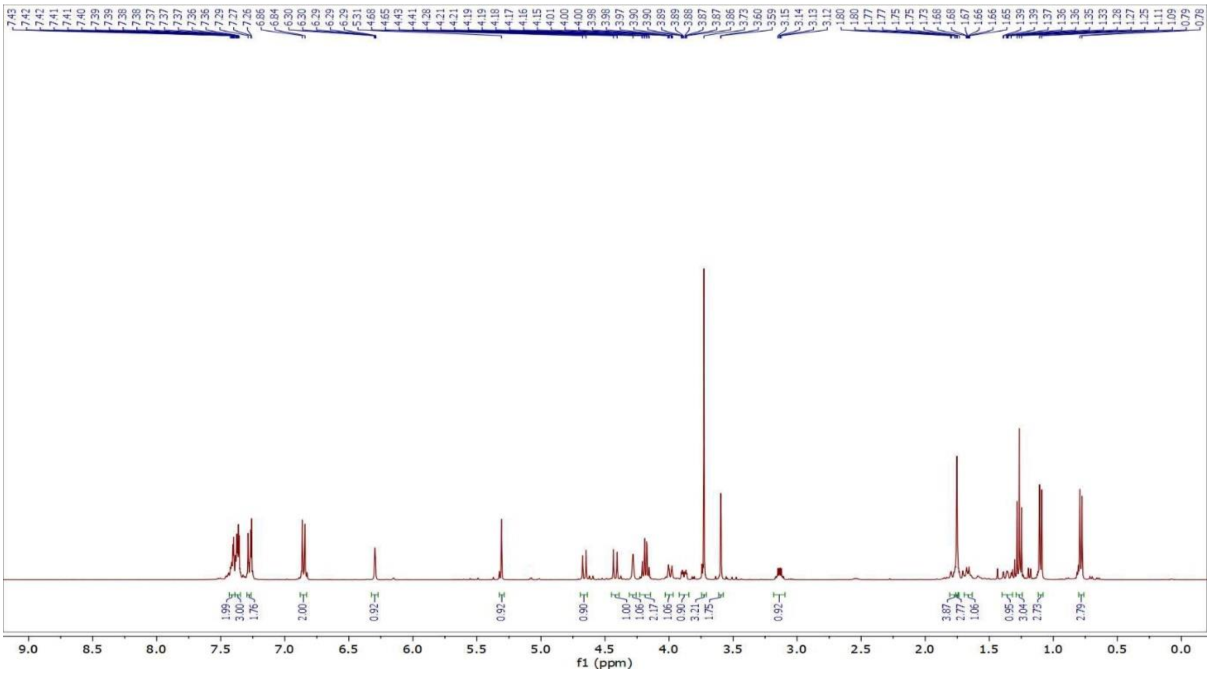

^{13}C NMR (101 MHz, CDCl$_3$)

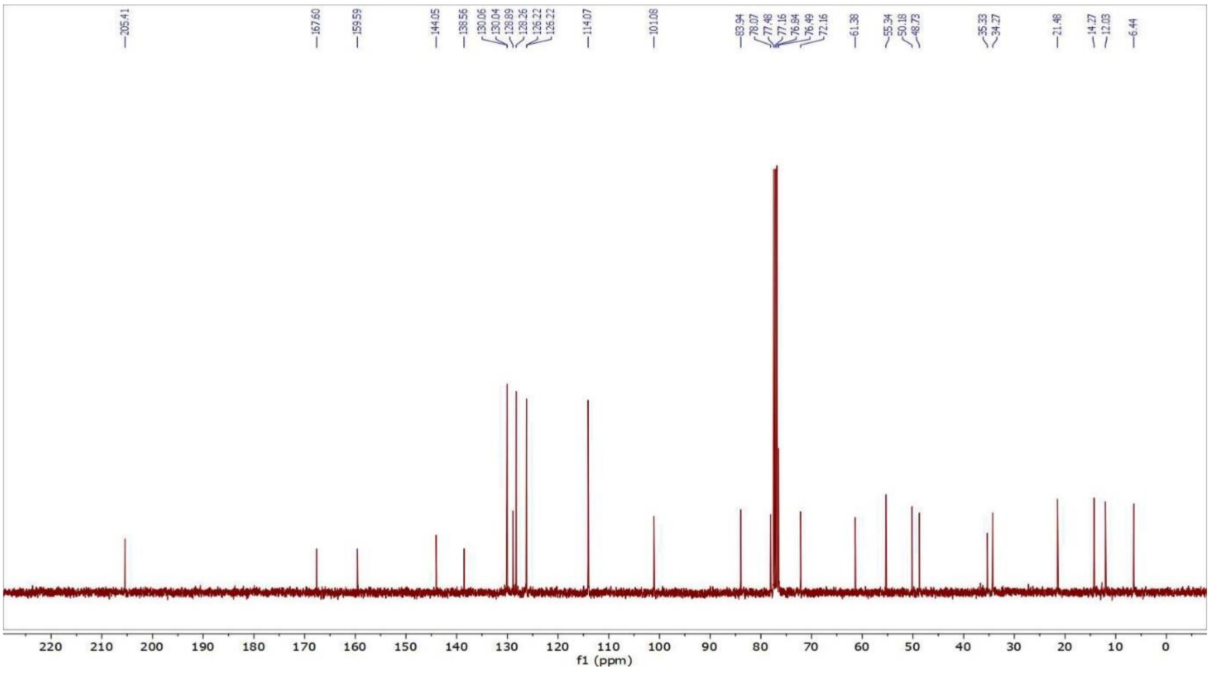

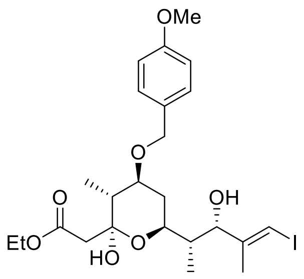

15

^{1}H NMR (400 MHz, $CDCl_3$)

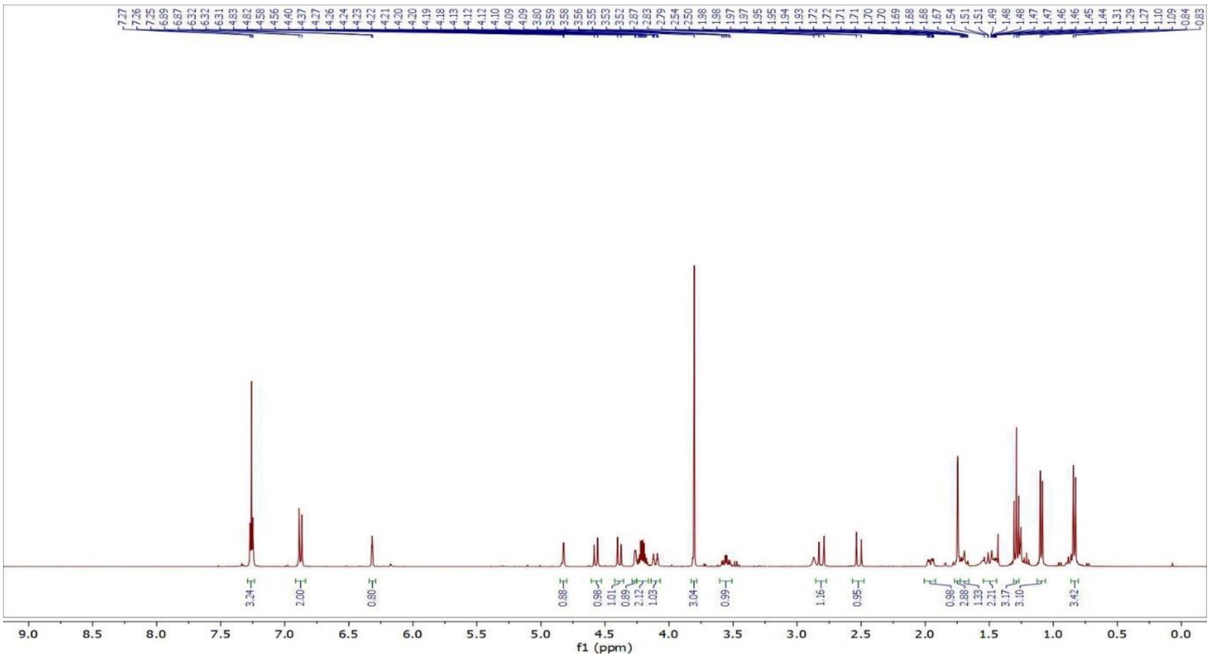

^{13}C NMR (101 MHz, $CDCl_3$)

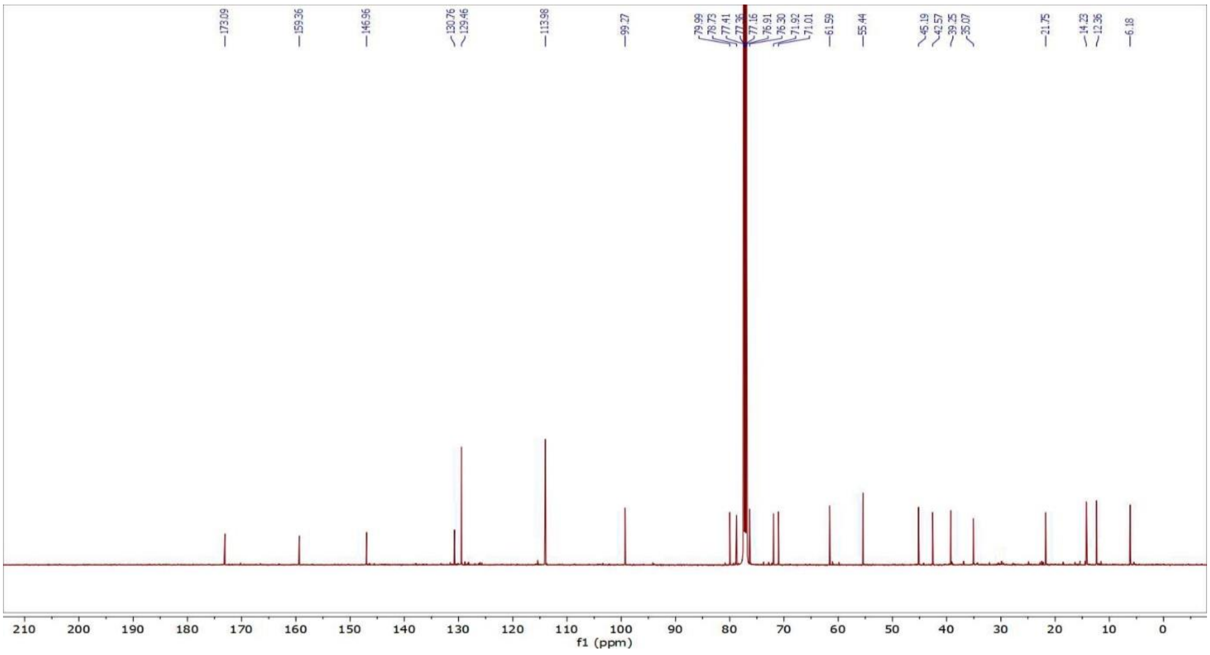

<u>NOESY spectrum of **15**</u>

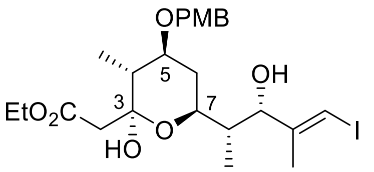

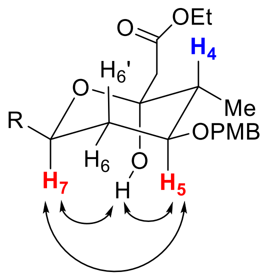

$J_{H5\text{-}H6}$ = 4.5 Hz
$J_{H5\text{-}H6'}$ (= $J_{H5\text{-}H4}$) = 10.6 Hz
$J_{H7\text{-}H6}$ = 2.2 Hz
$J_{H7\text{-}H6'}$ (= $J_{H7\text{-}H8}$) = 12.2 Hz

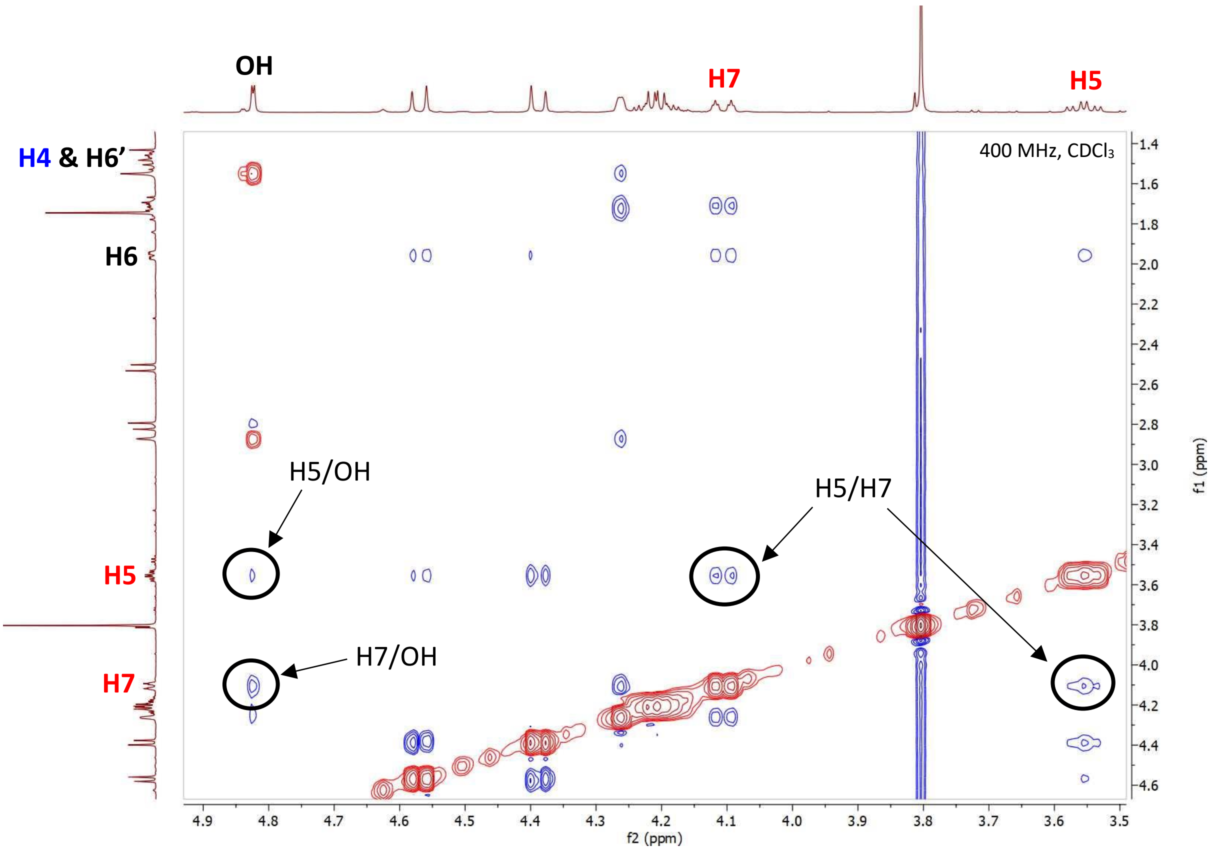

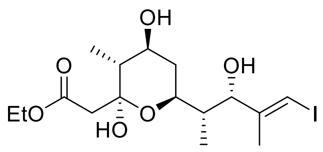

2

^{1}H NMR (400 MHz, $CDCl_3$)

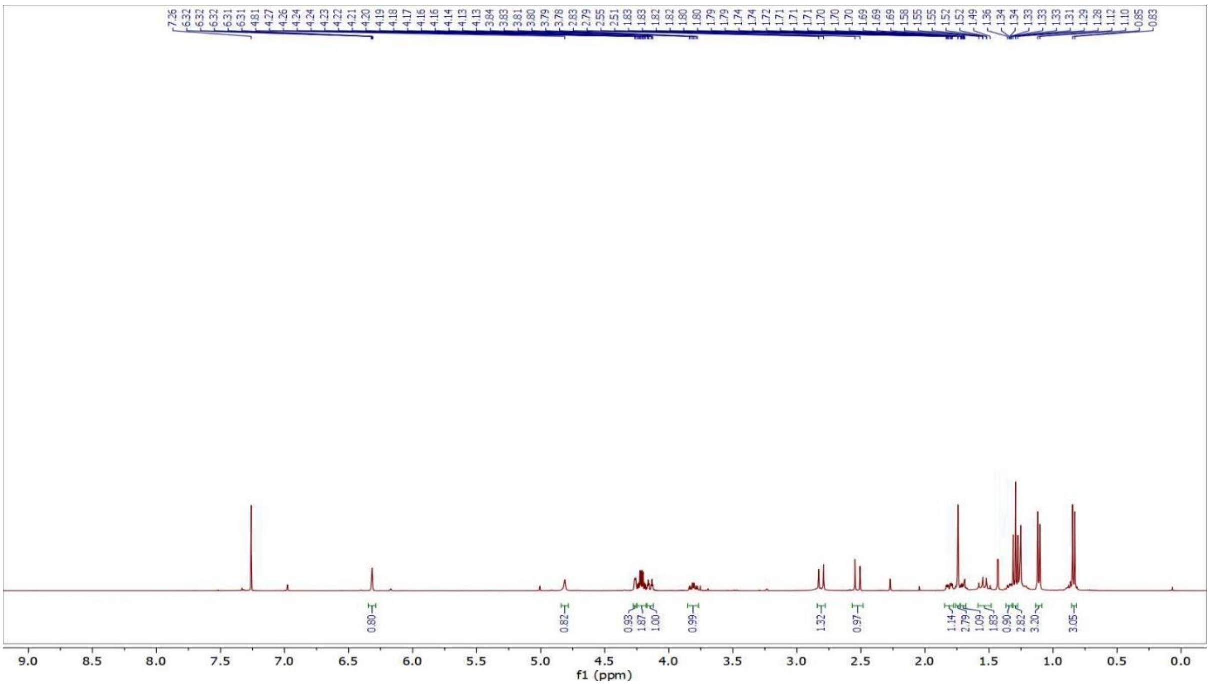

^{13}C NMR (101 MHz, $CDCl_3$)

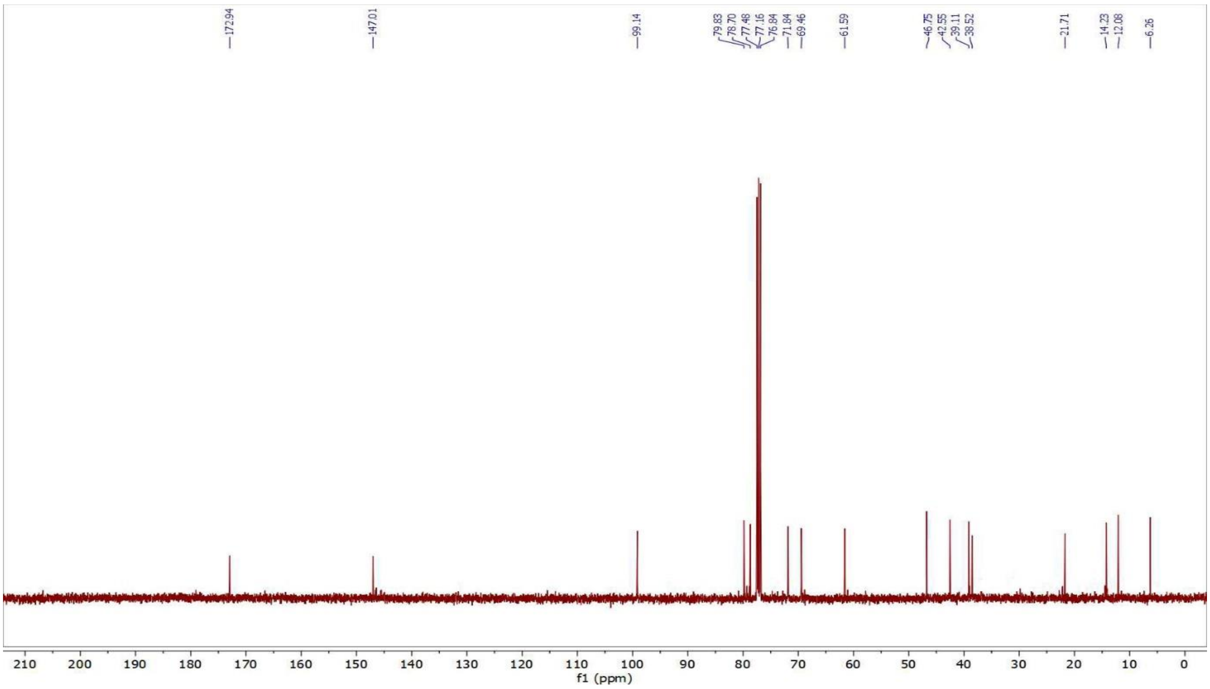

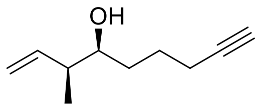

17

^{1}H NMR (400 MHz, $CDCl_3$)

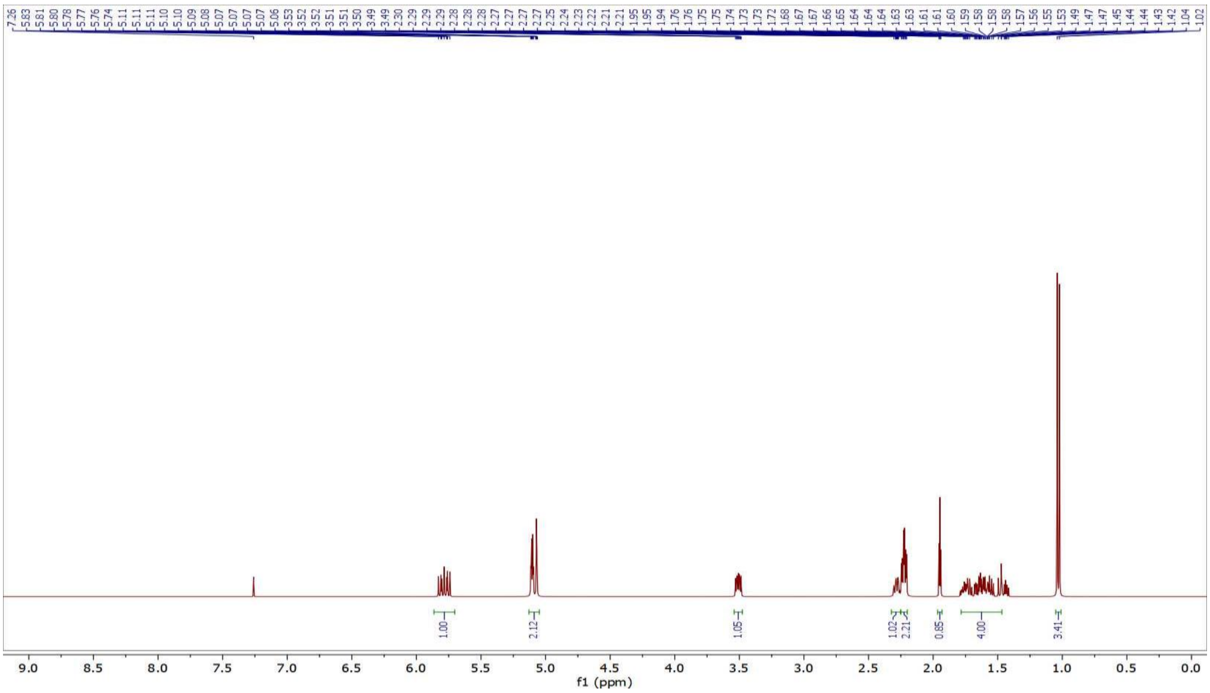

^{13}C NMR (101 MHz, $CDCl_3$)

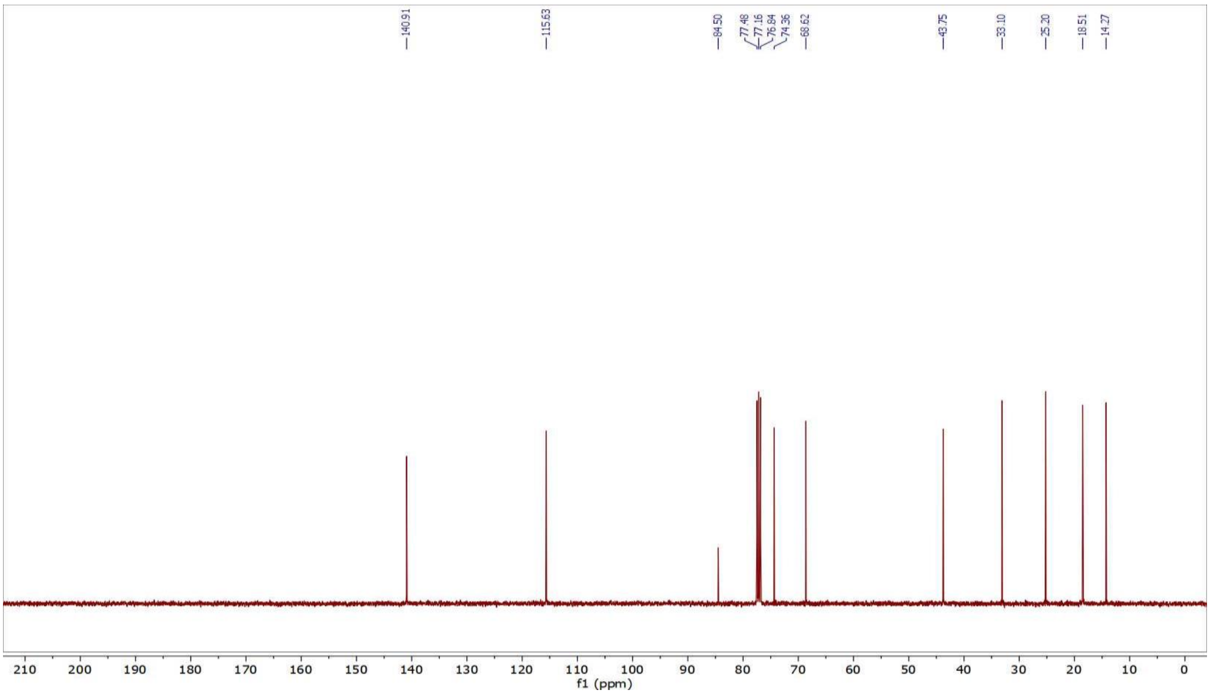

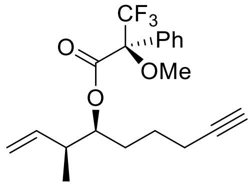

(*S*)-MTPA ester of 17

^{1}H NMR (500 MHz, $CDCl_3$)

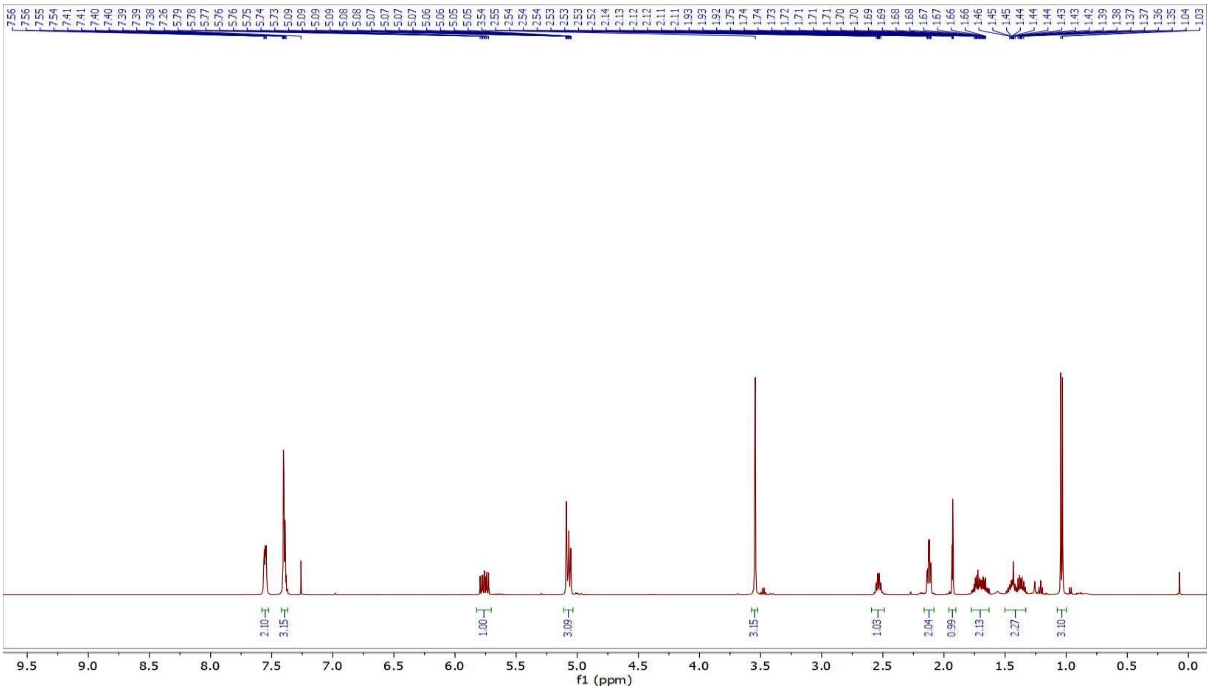

^{13}C NMR (126 MHz, $CDCl_3$)

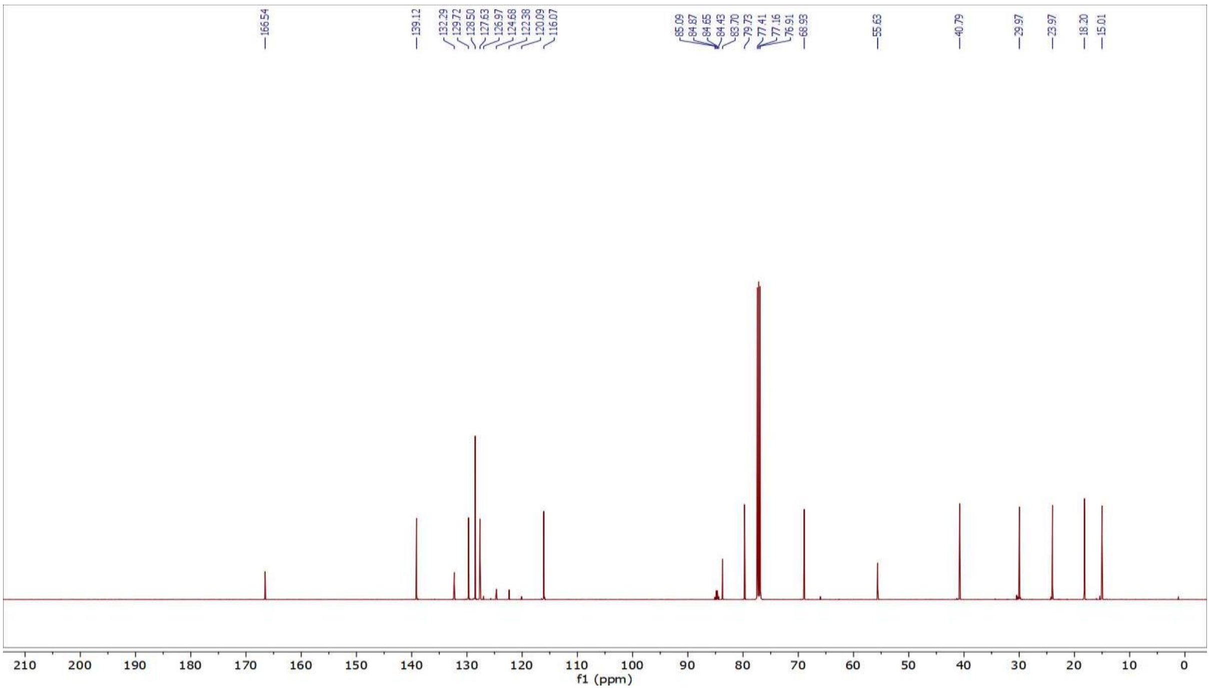

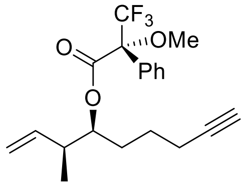

(*R*)-MTPA ester of 17

^{1}H NMR (500 MHz, CDCl$_3$)

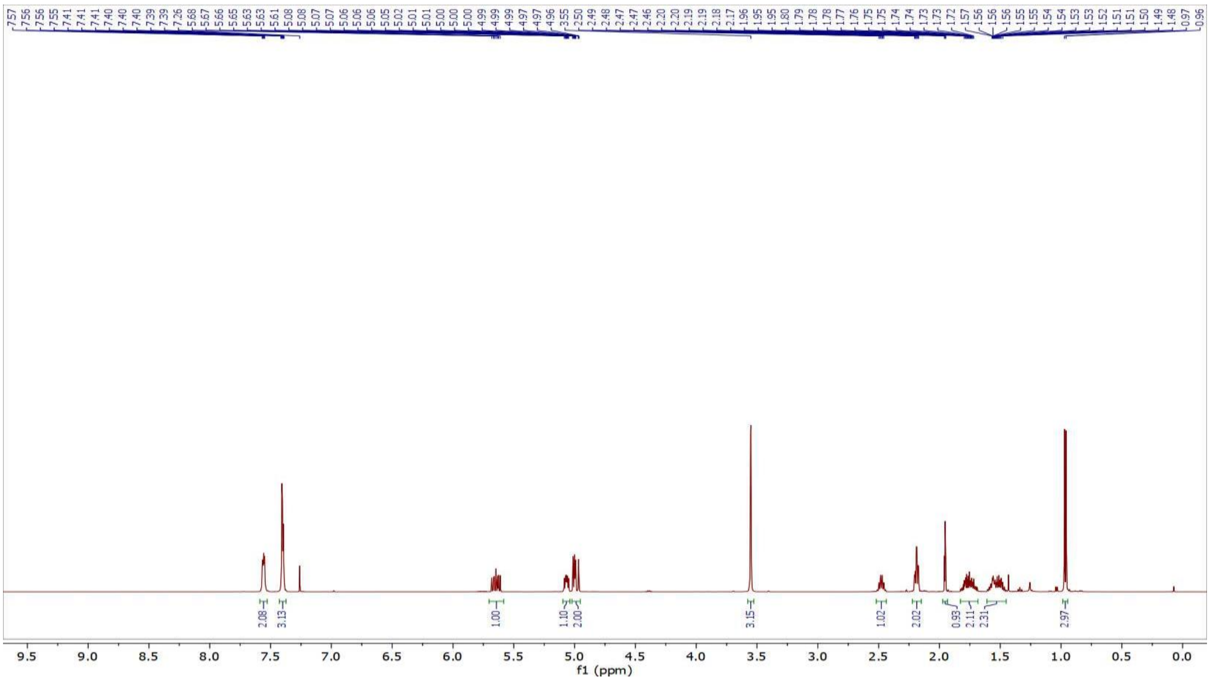

^{13}C NMR (126 MHz, CDCl$_3$)

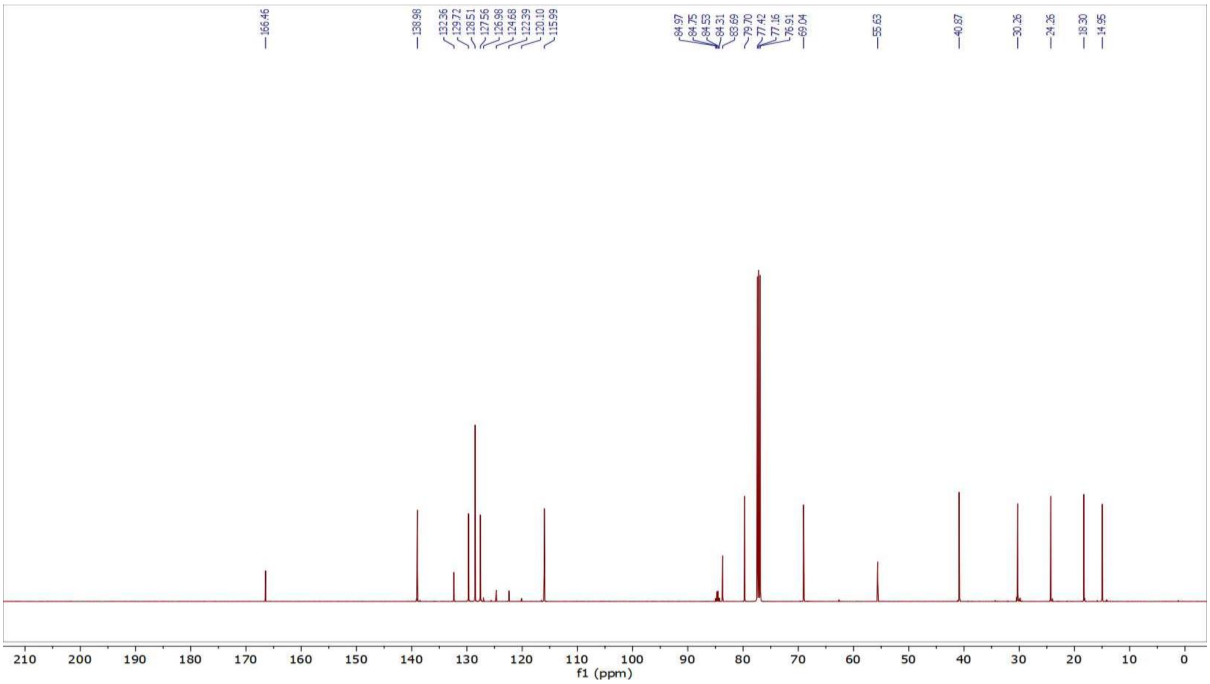

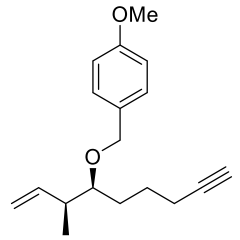

17.1

^{1}H NMR (400 MHz, CDCl$_3$)

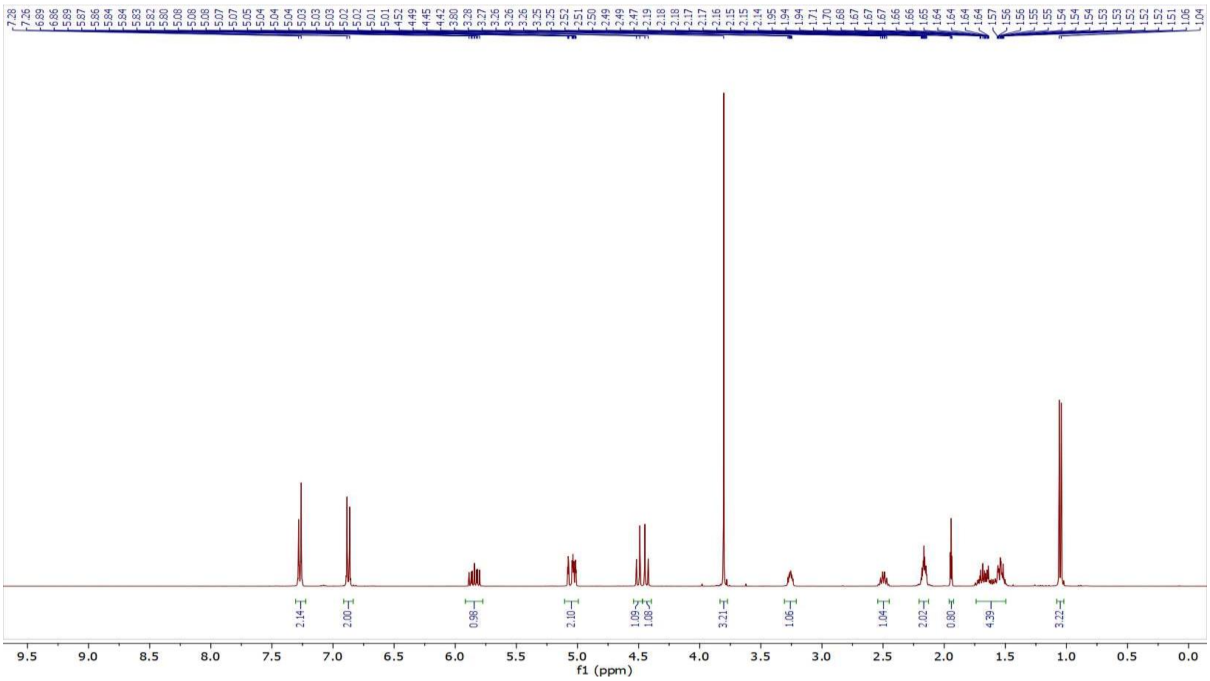

^{13}C NMR (101 MHz, CDCl$_3$)

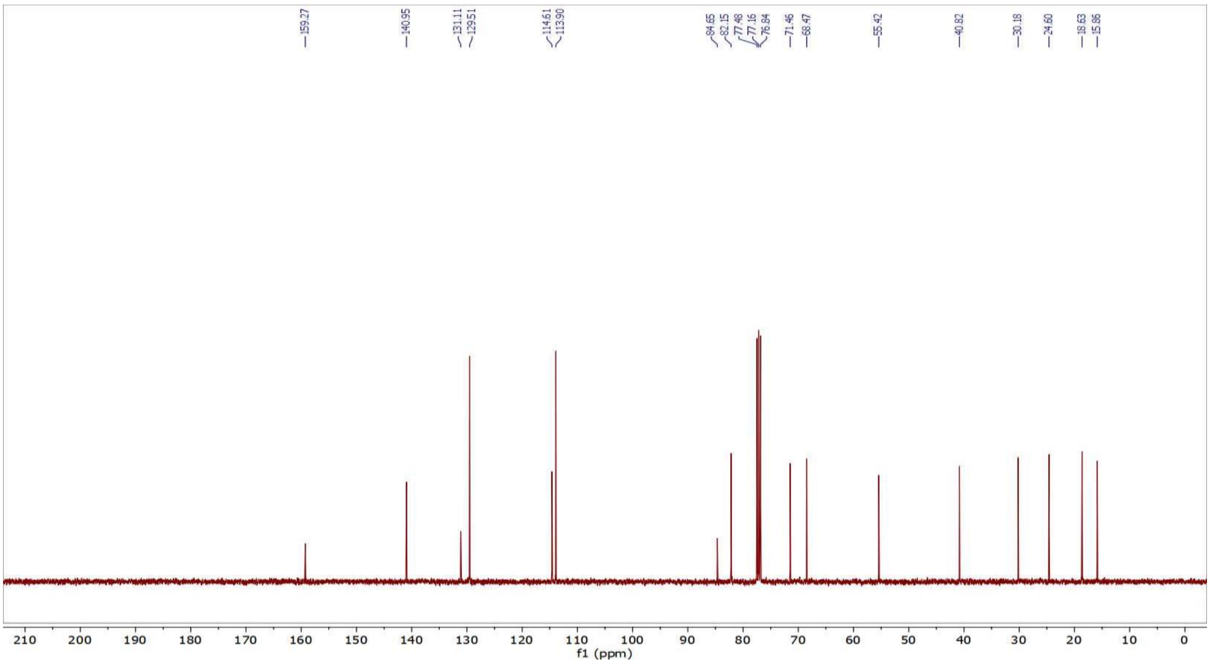

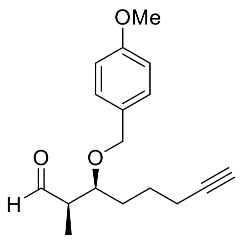

18

^{1}H NMR (400 MHz, $CDCl_3$)

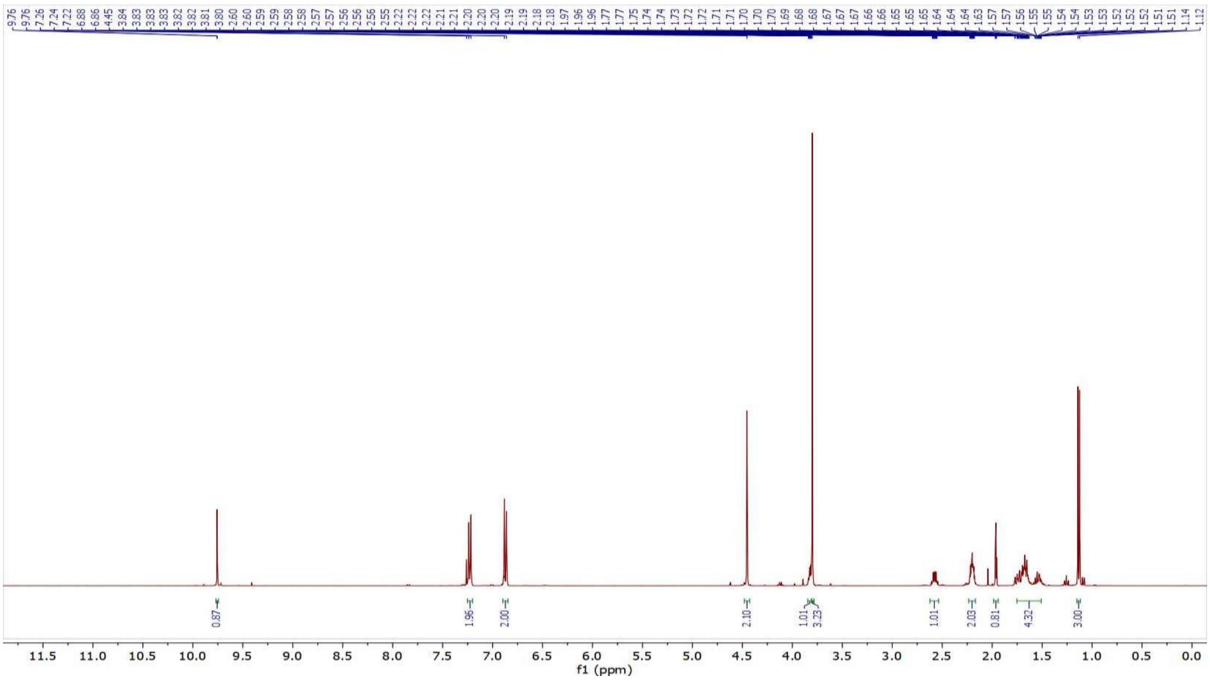

^{13}C NMR (101 MHz, $CDCl_3$)

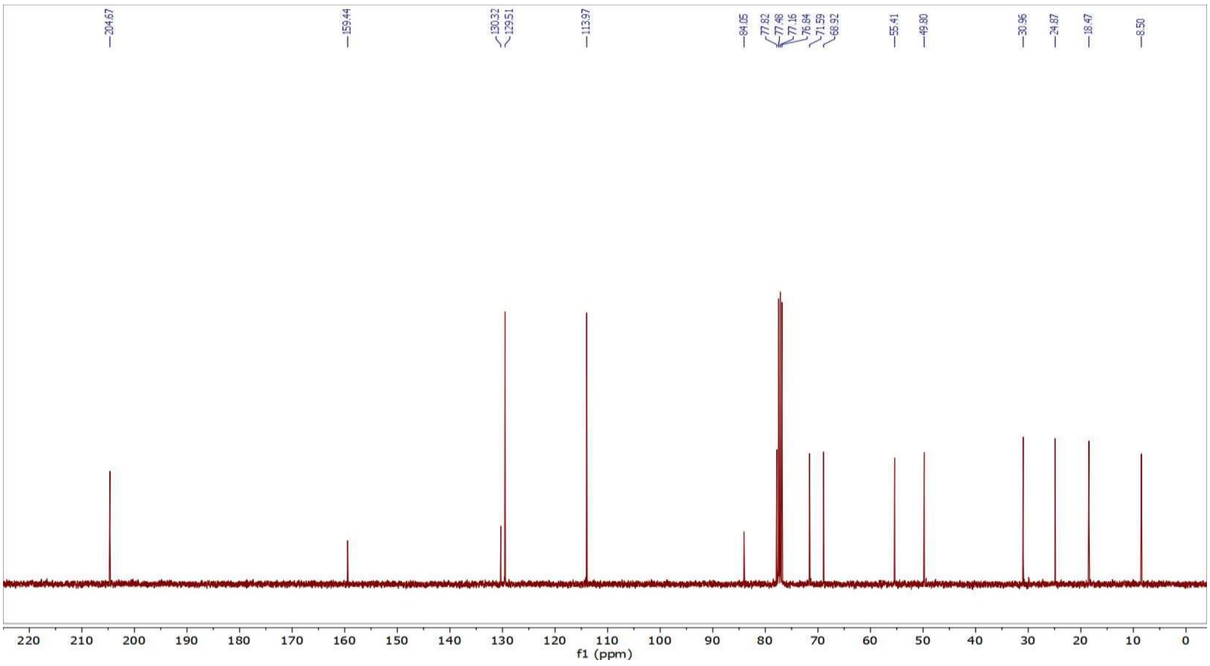

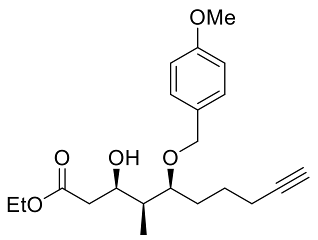

19

^{1}H NMR (400 MHz, CDCl$_3$)

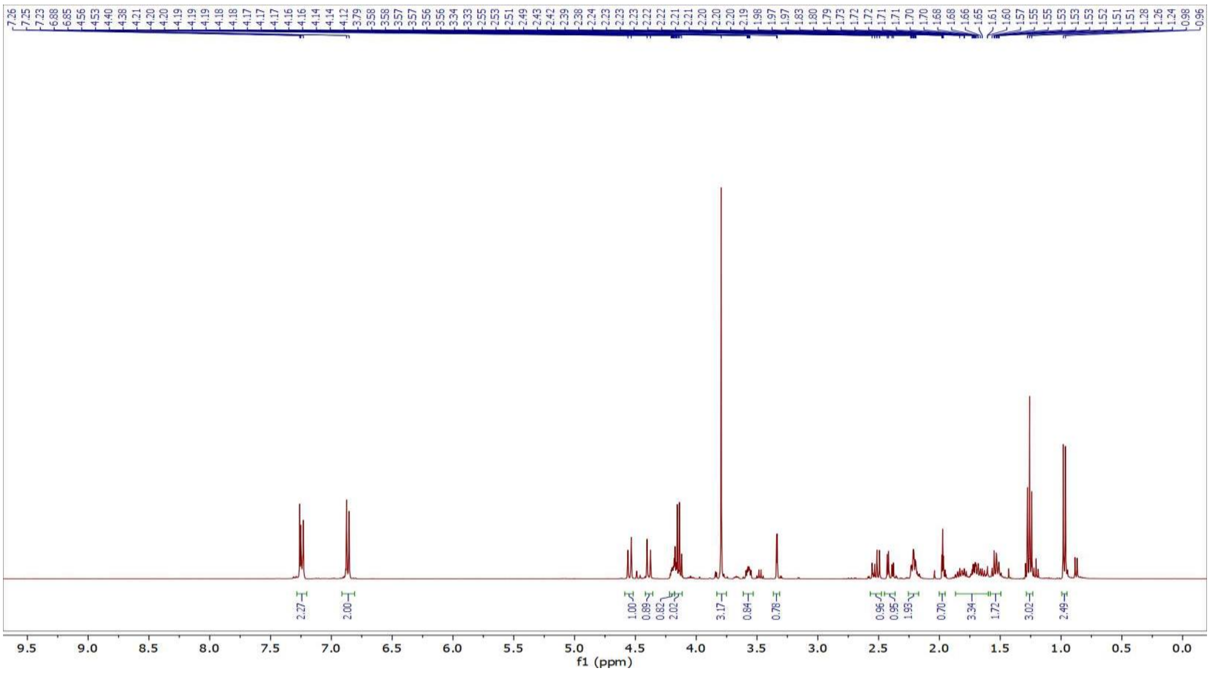

^{13}C NMR (101 MHz, CDCl$_3$)

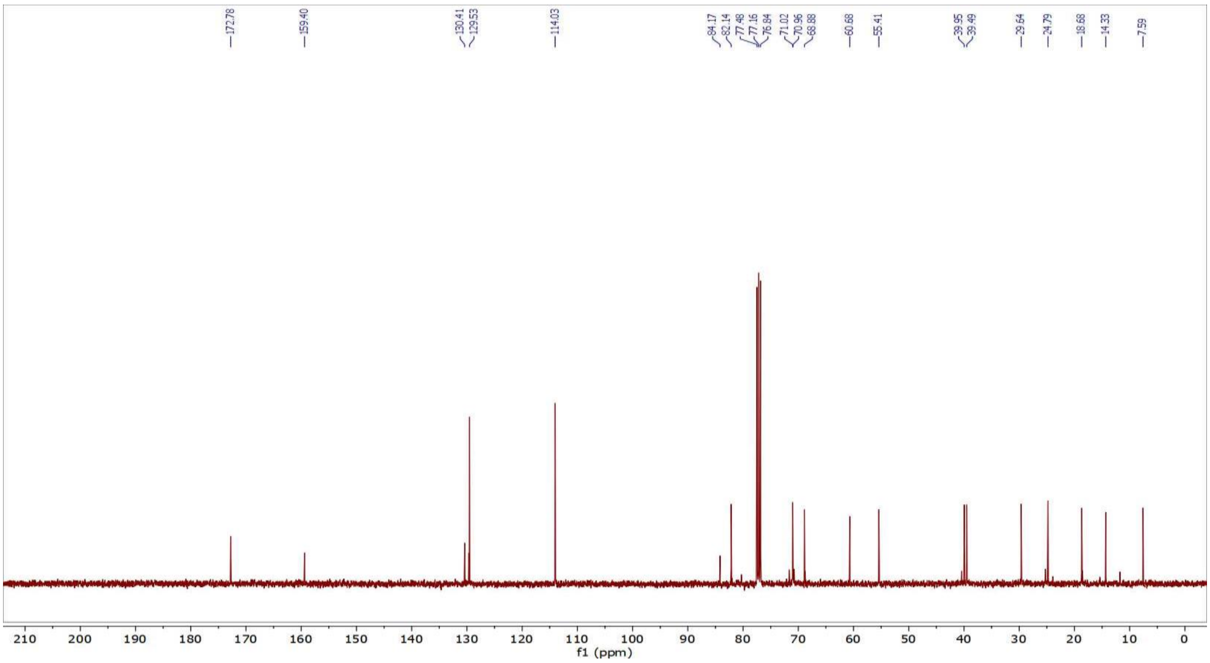

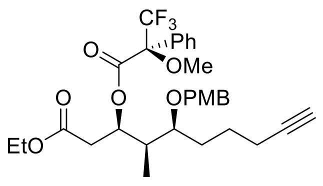

(*S*)-MTPA ester of 19

^{1}H NMR (500 MHz, CDCl₃)

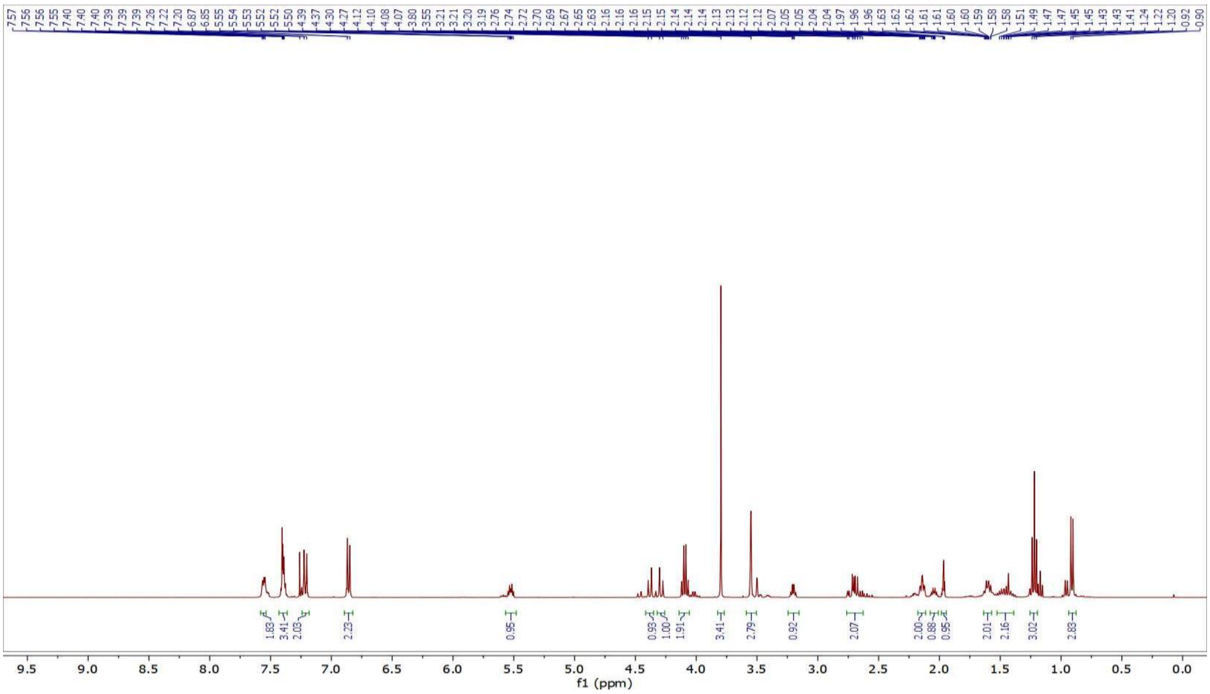

^{13}C NMR (126 MHz, CDCl₃)

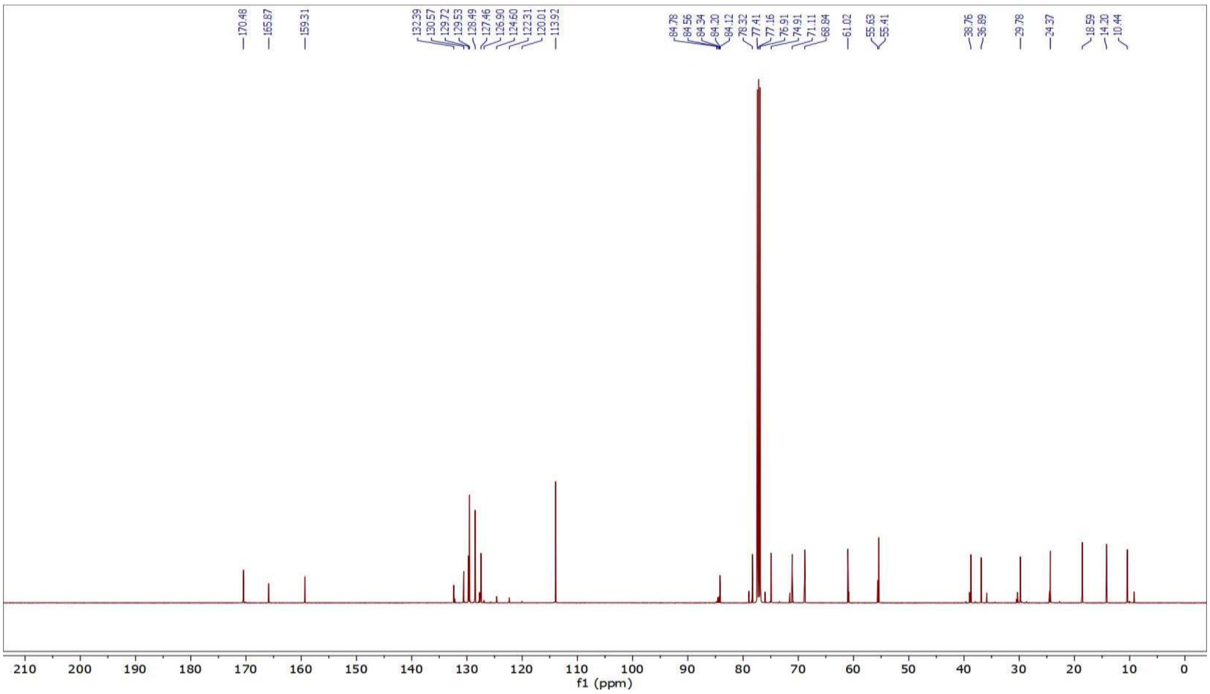

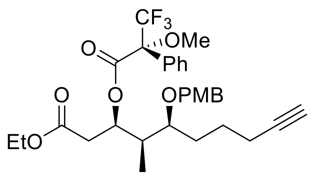

(*R*)-MTPA ester of 19

^{1}H NMR (500 MHz, $CDCl_3$)

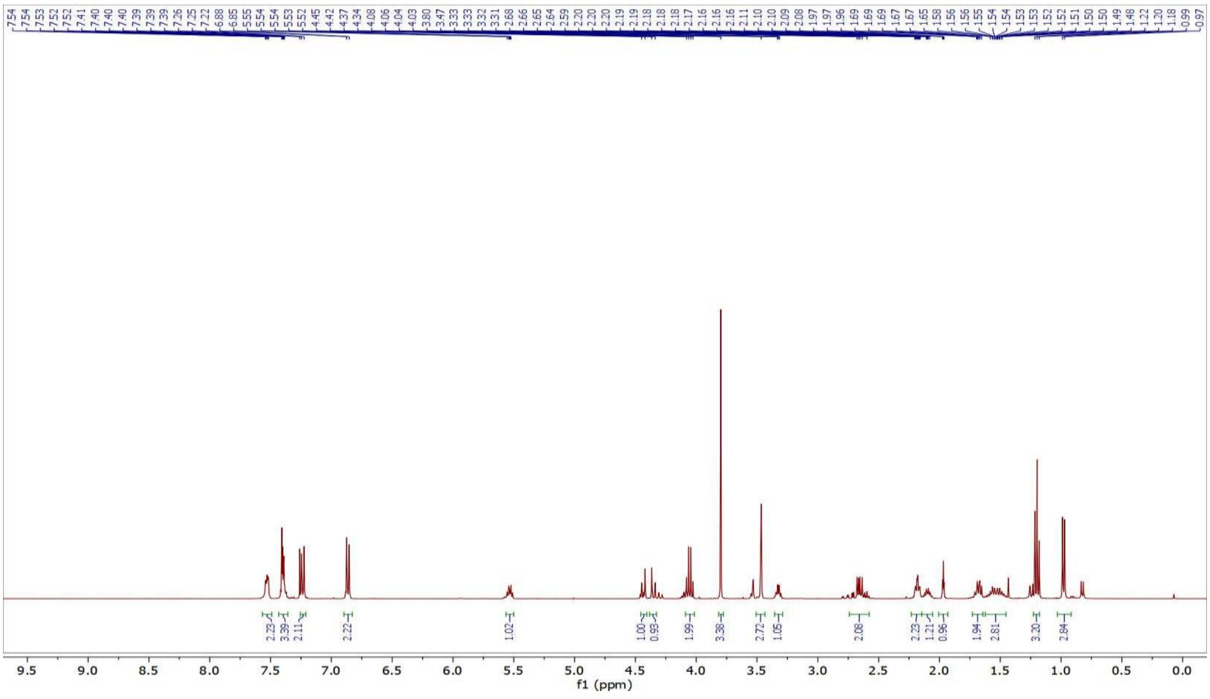

^{13}C NMR (126 MHz, $CDCl_3$)

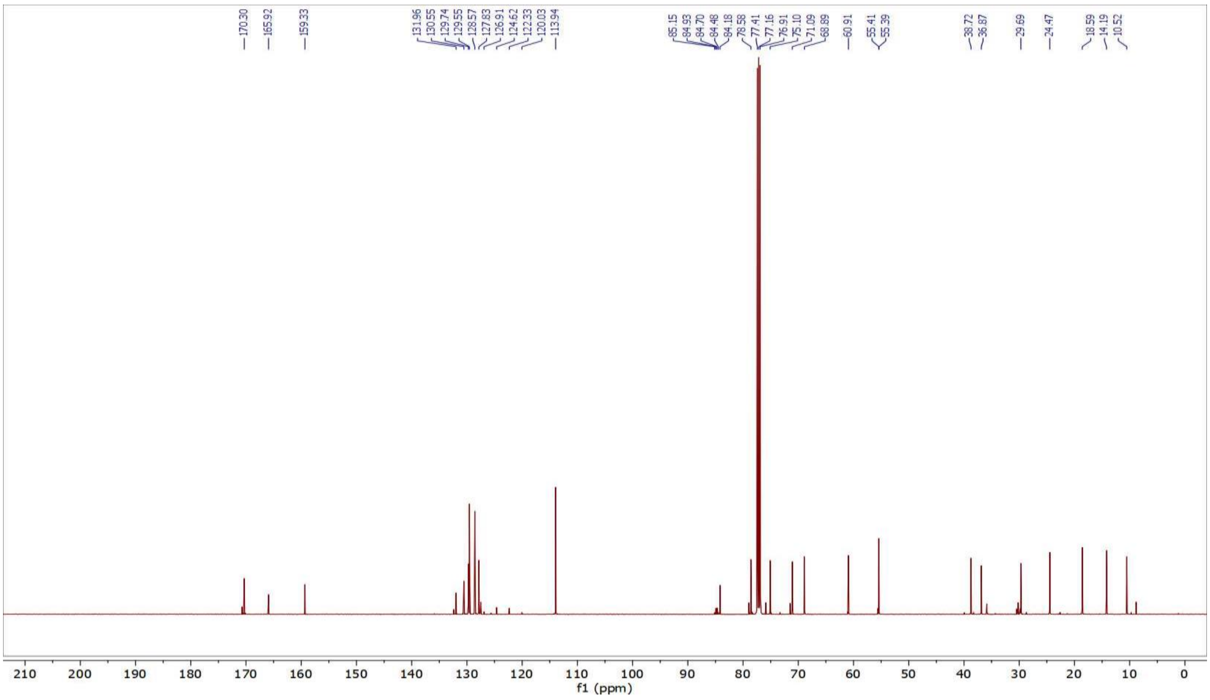

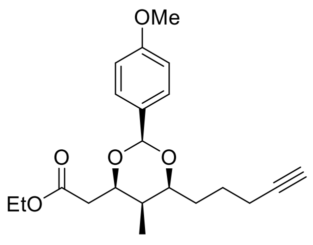

3

^{1}H NMR (400 MHz, CDCl$_3$)

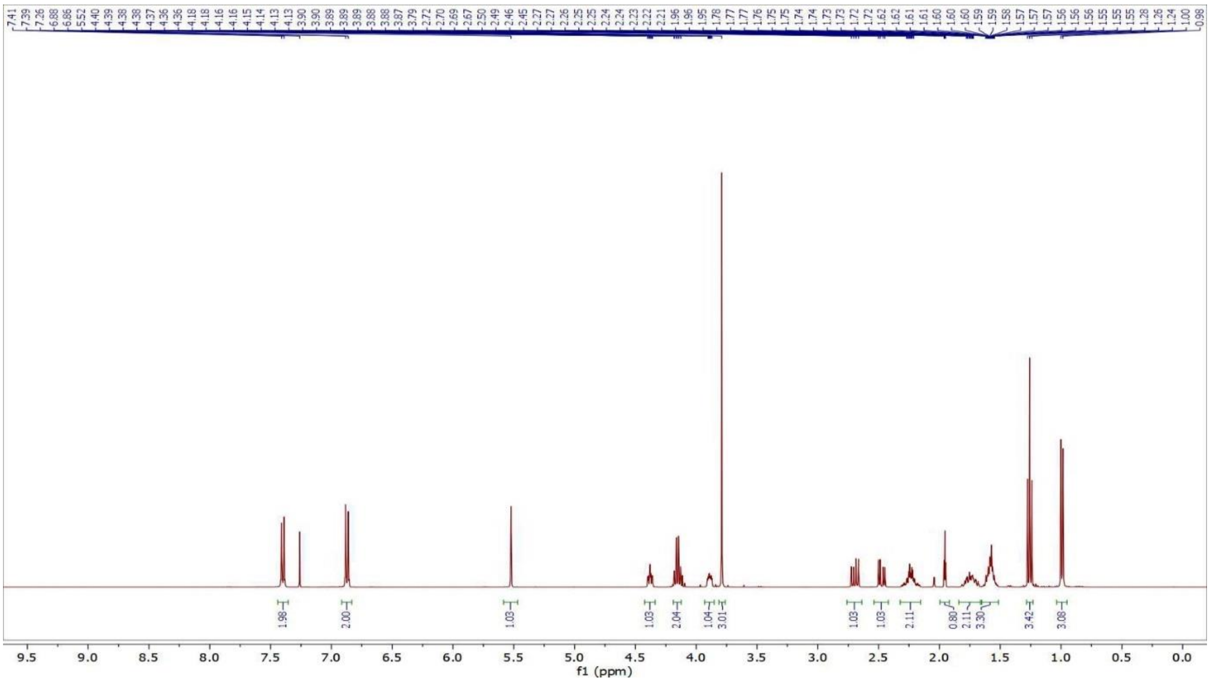

^{13}C NMR (101 MHz, CDCl$_3$)

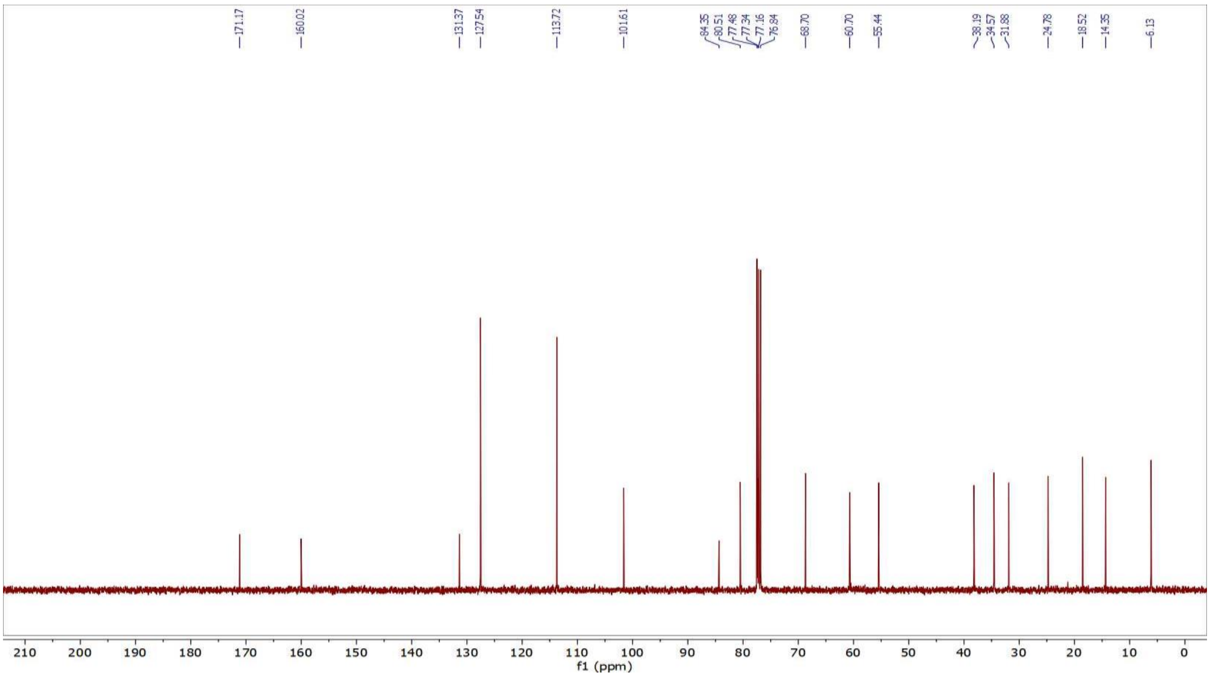

NOESY spectrum of **3**

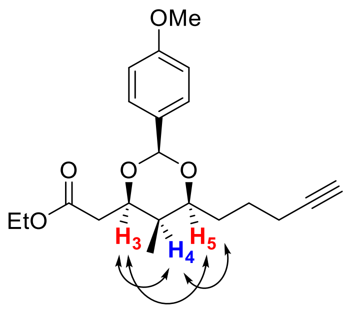

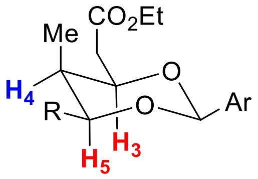

$J_{H3\text{-}H4}$ = 2.3 Hz
$J_{H4\text{-}H5}$ = 2.3 Hz

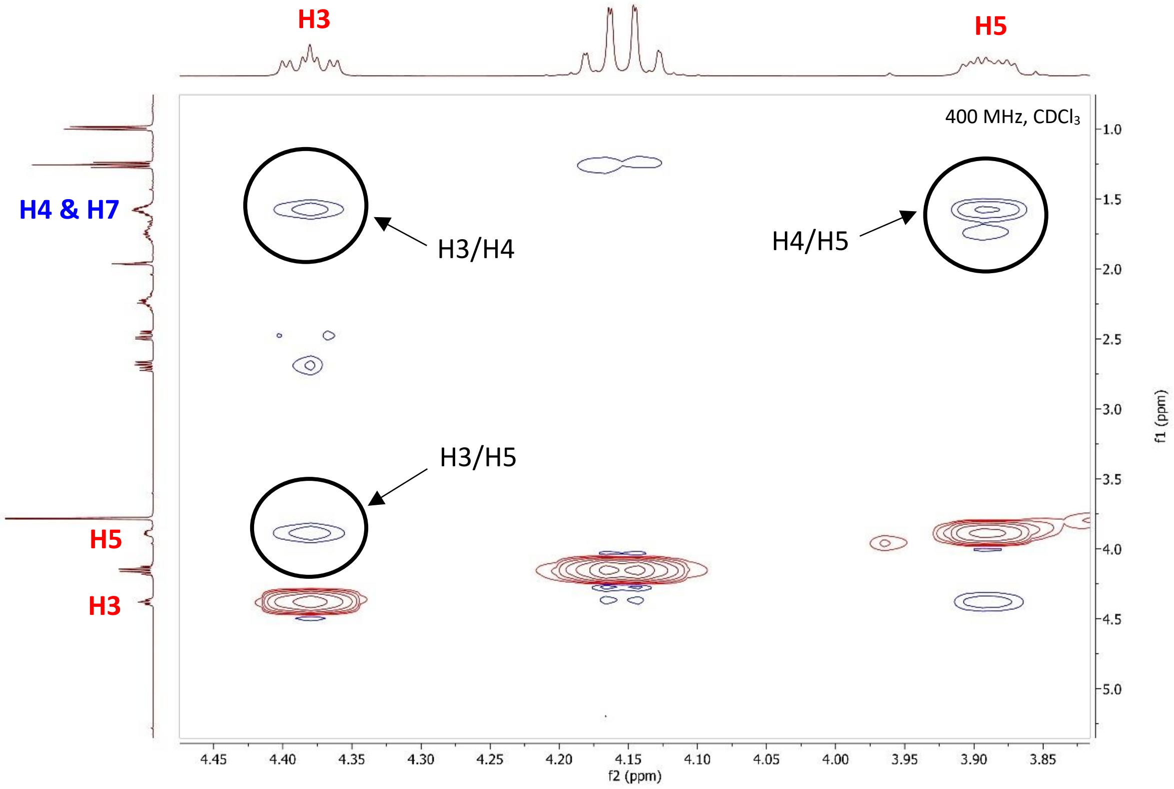

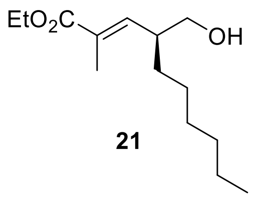

21

^{1}H NMR (400 MHz, CDCl$_3$)

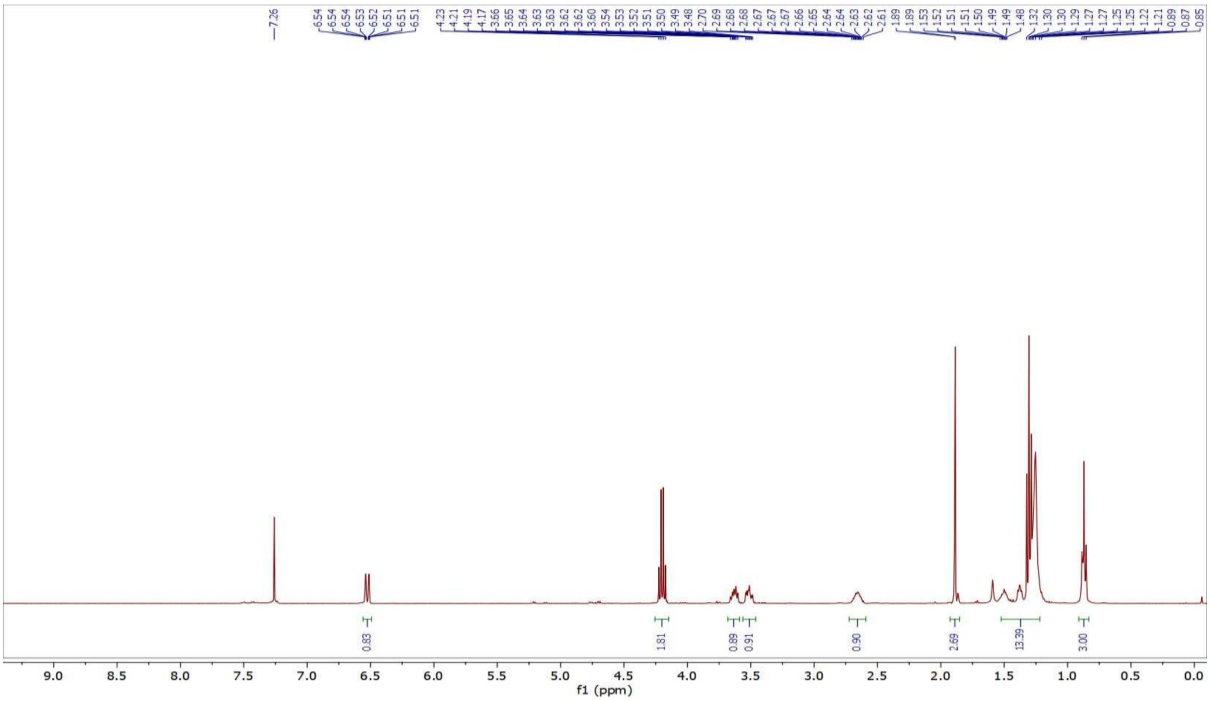

^{13}C NMR (101 MHz, CDCl$_3$)

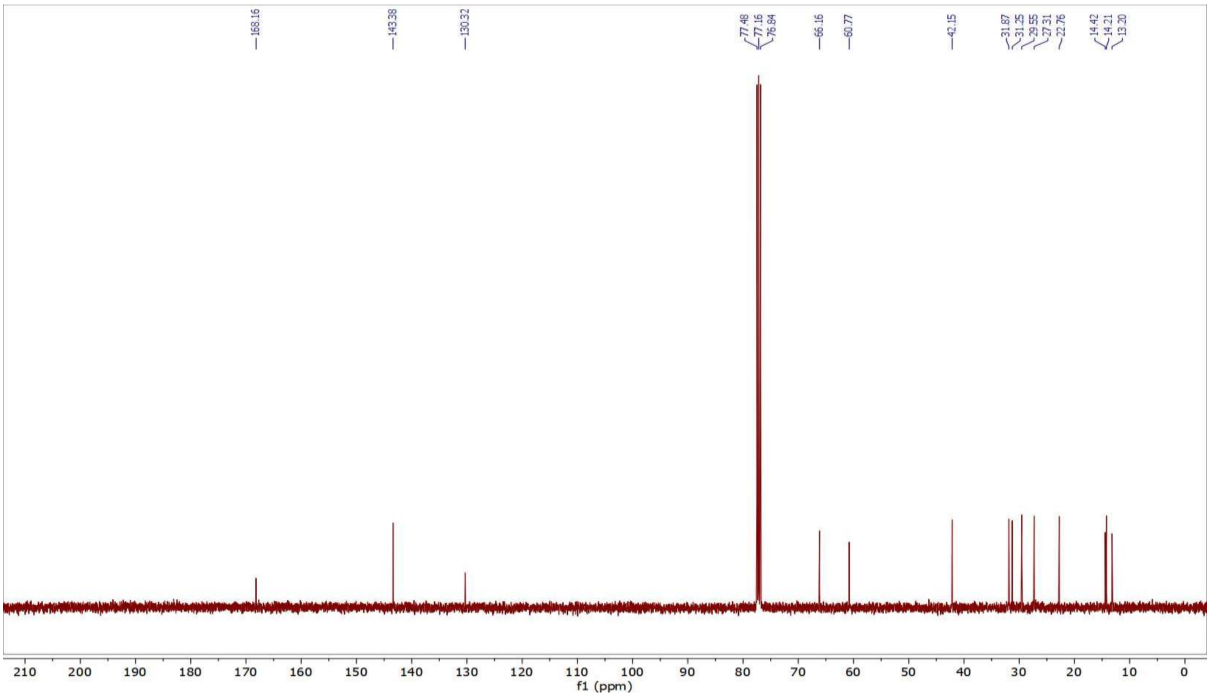

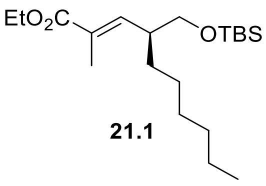

21.1

^{1}H NMR (400 MHz, CDCl$_3$)

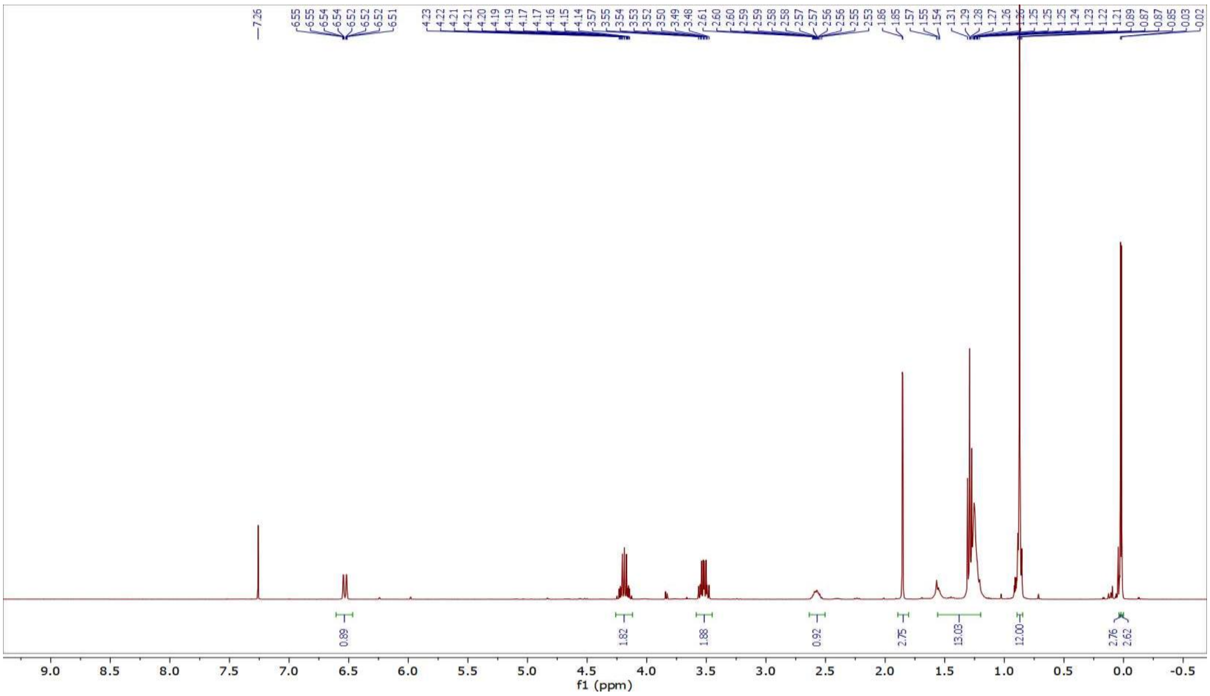

^{13}C NMR (101 MHz, CDCl$_3$)

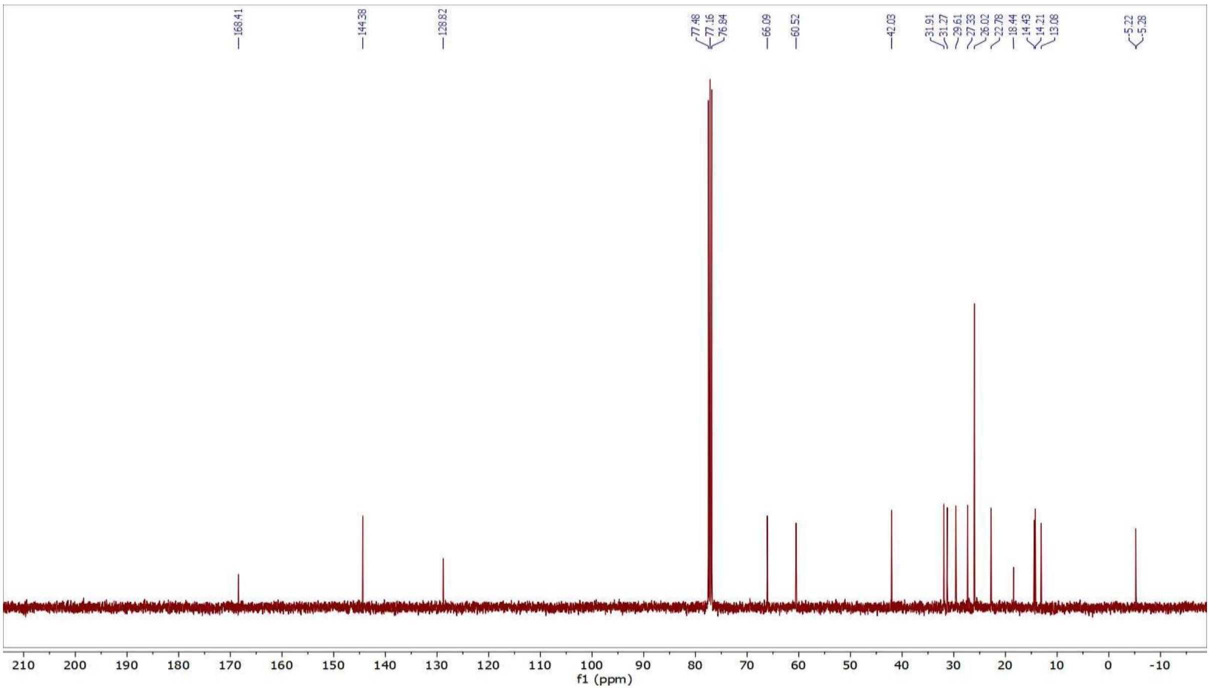

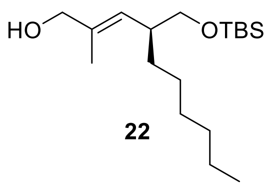

22

^{1}H NMR (400 MHz, CDCl3)

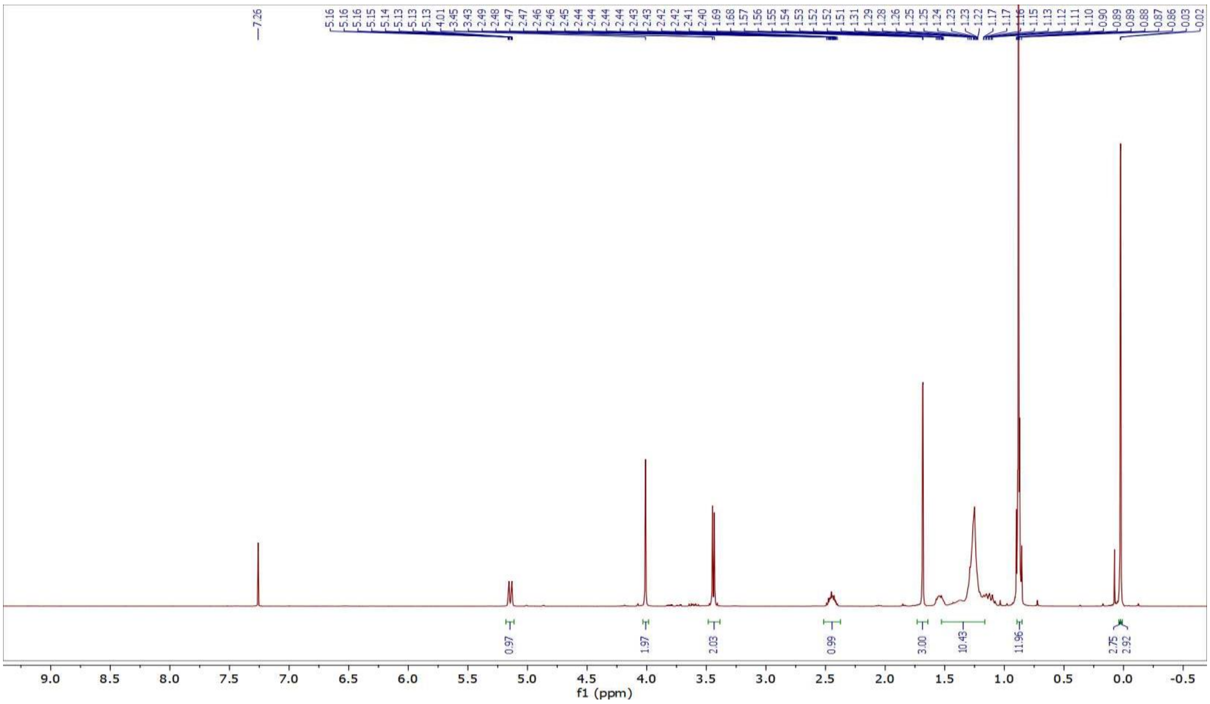

^{13}C NMR (101 MHz, CDCl3)

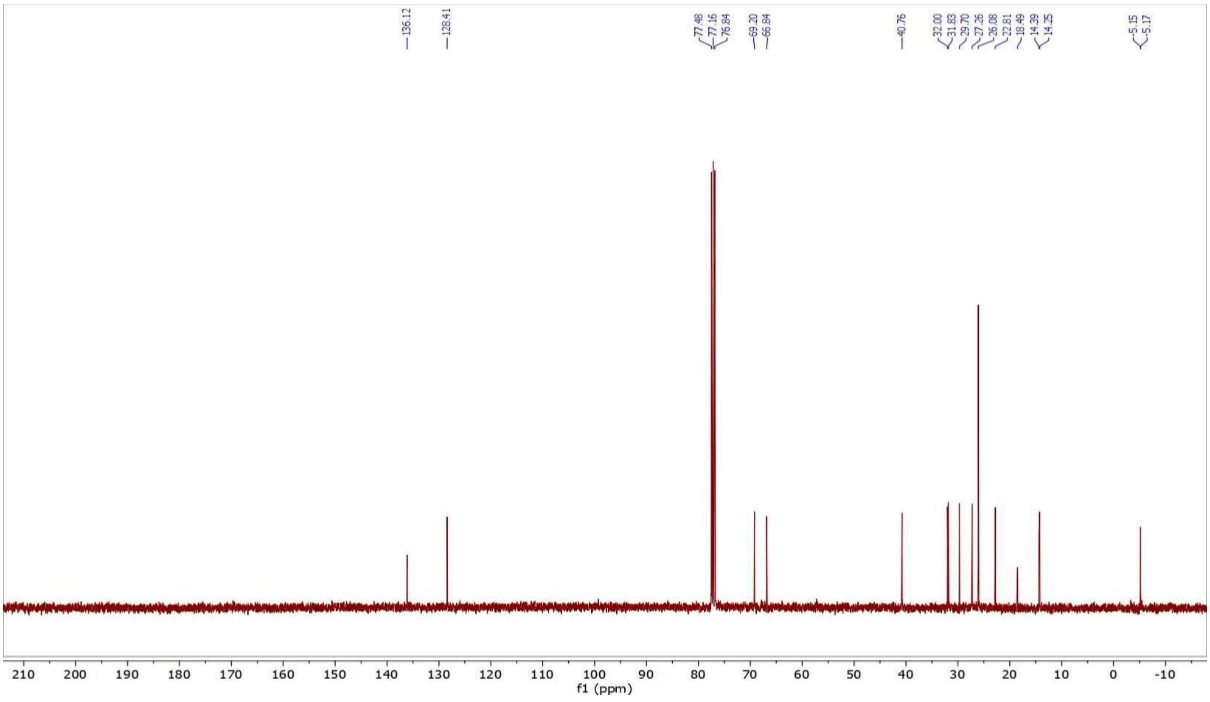

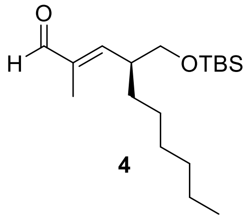

4

^{1}H NMR (400 MHz, CDCl$_3$)

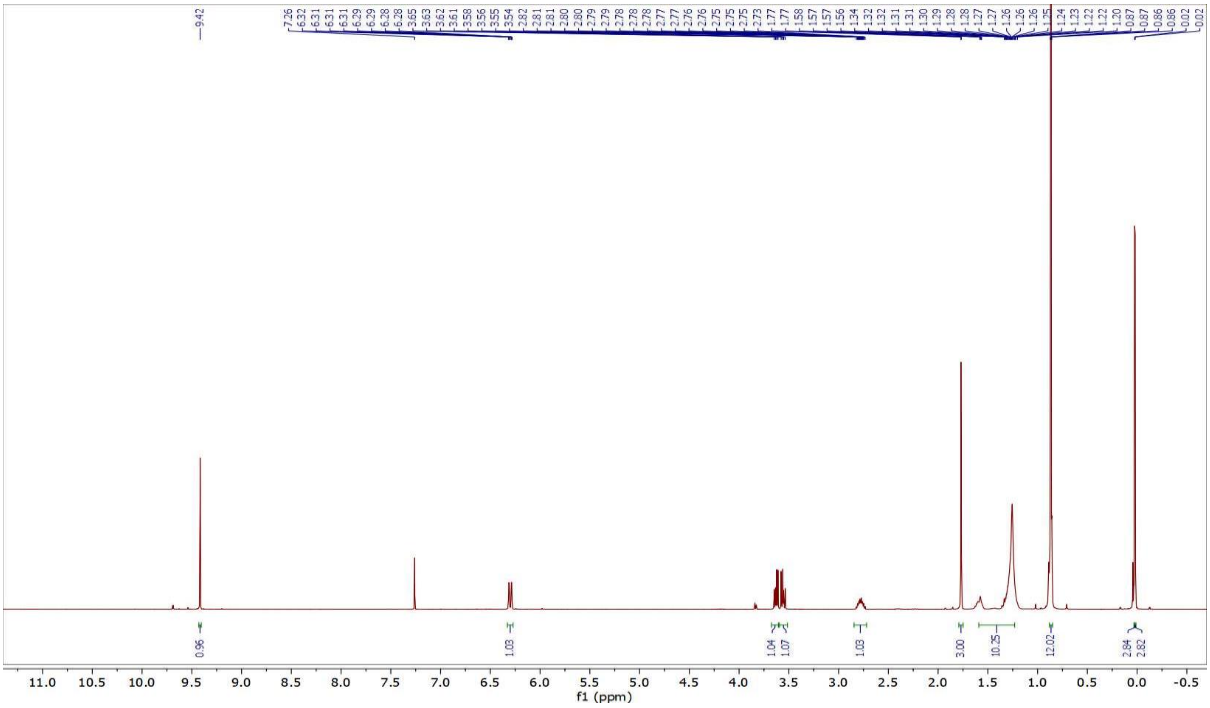

^{13}C NMR (101 MHz, CDCl$_3$)

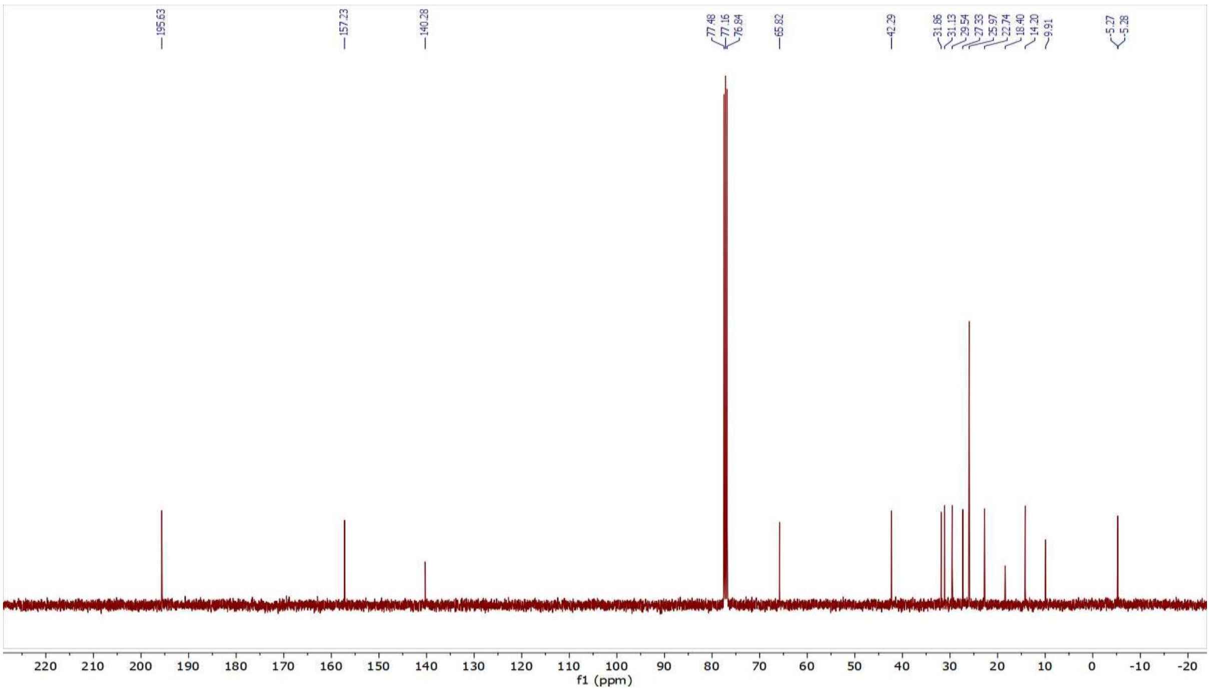

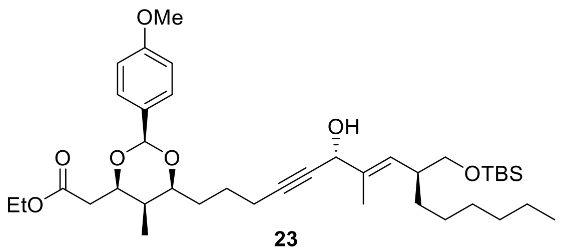

23

^{1}H NMR (400 MHz, $CDCl_3$)

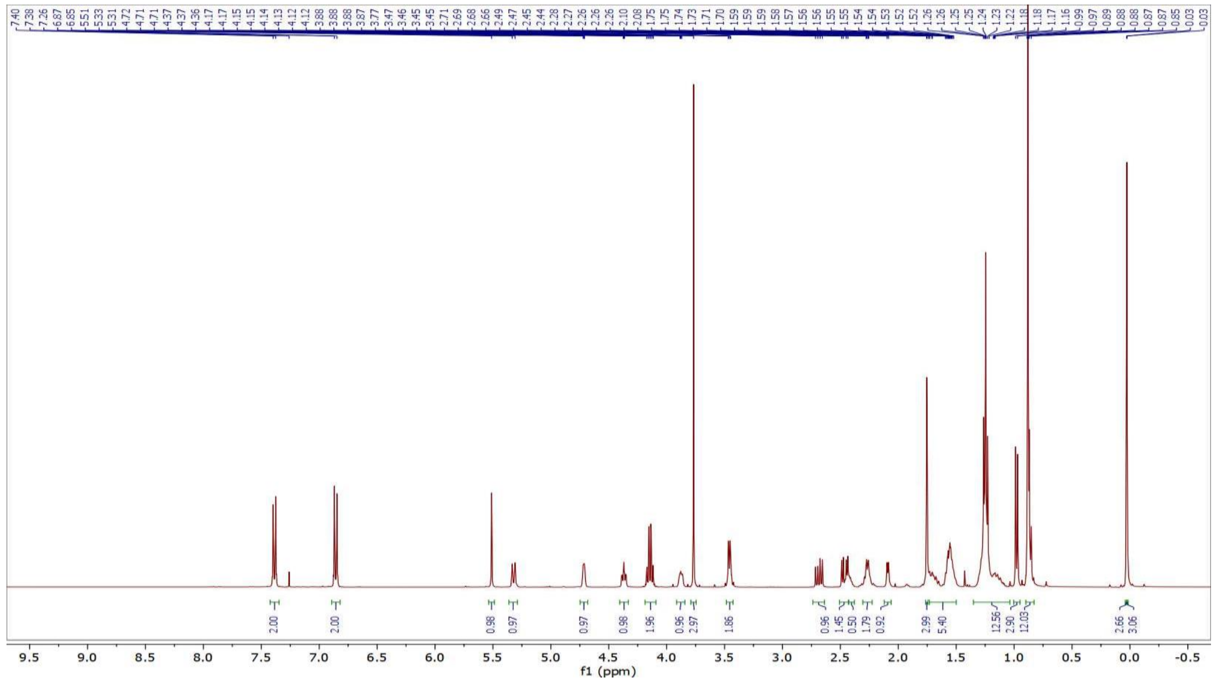

^{13}C NMR (101 MHz, $CDCl_3$)

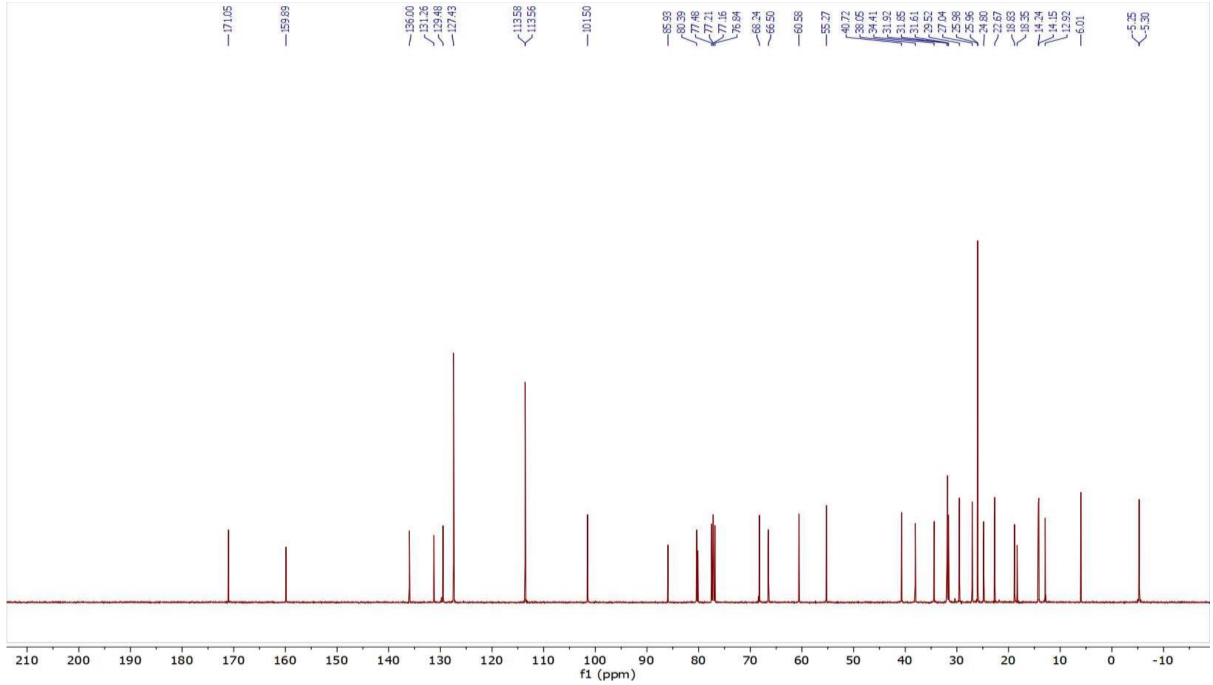

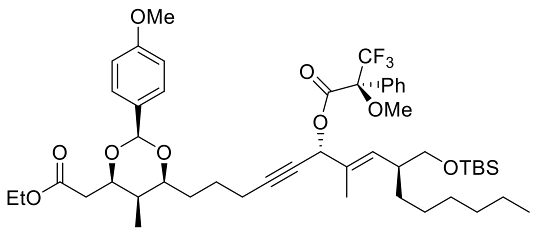

(S)-MTPA ester of 23

^{1}H NMR (500 MHz, $CDCl_3$)

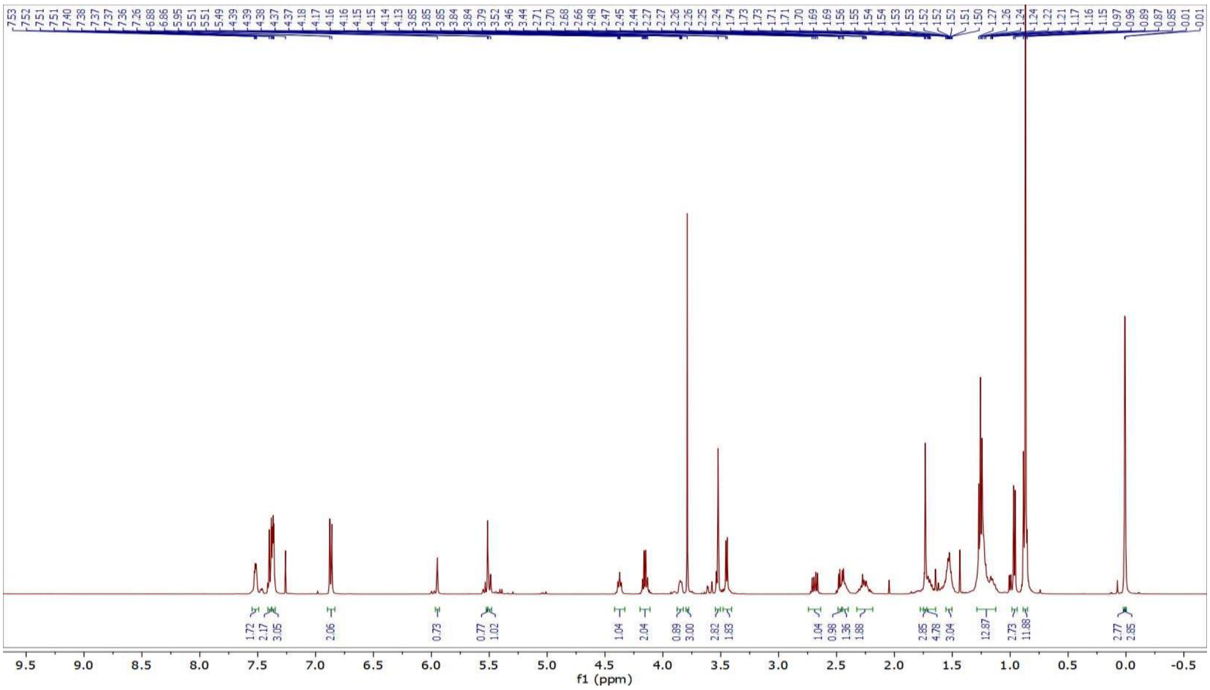

^{13}C NMR (126 MHz, $CDCl_3$)

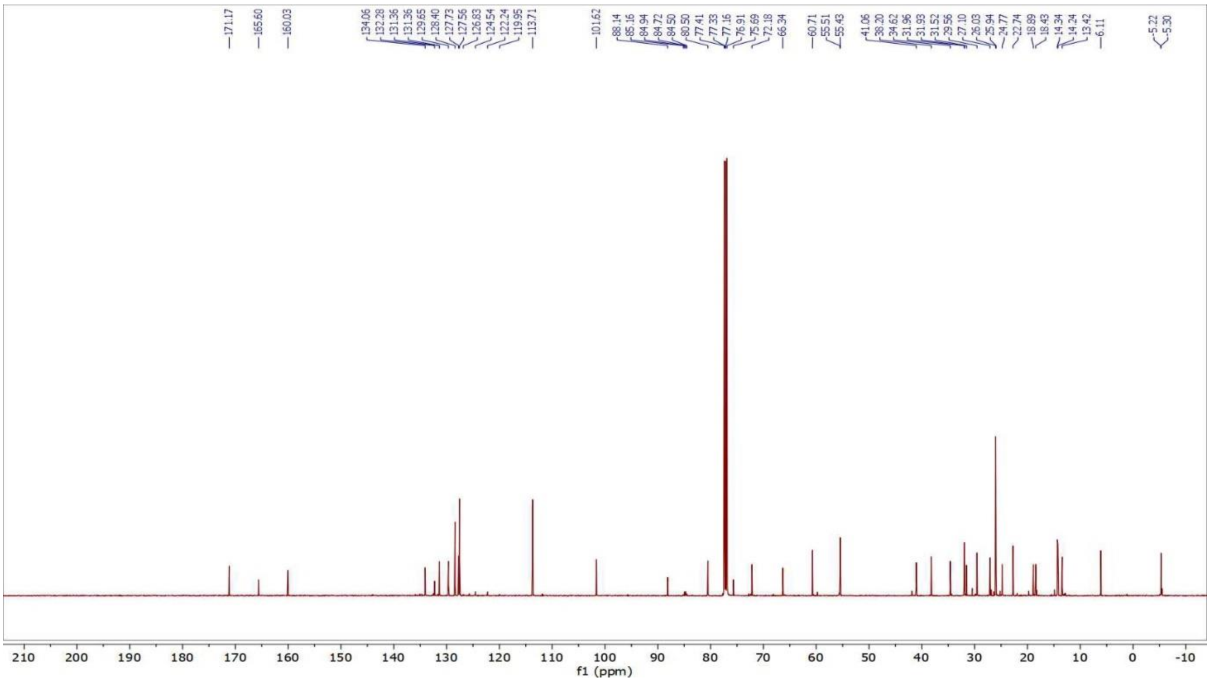

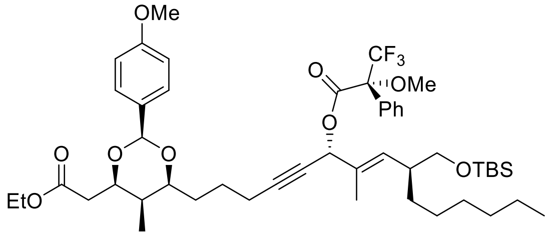

(*R*)-MTPA ester of 23

^{1}H NMR (500 MHz, CDCl$_3$)

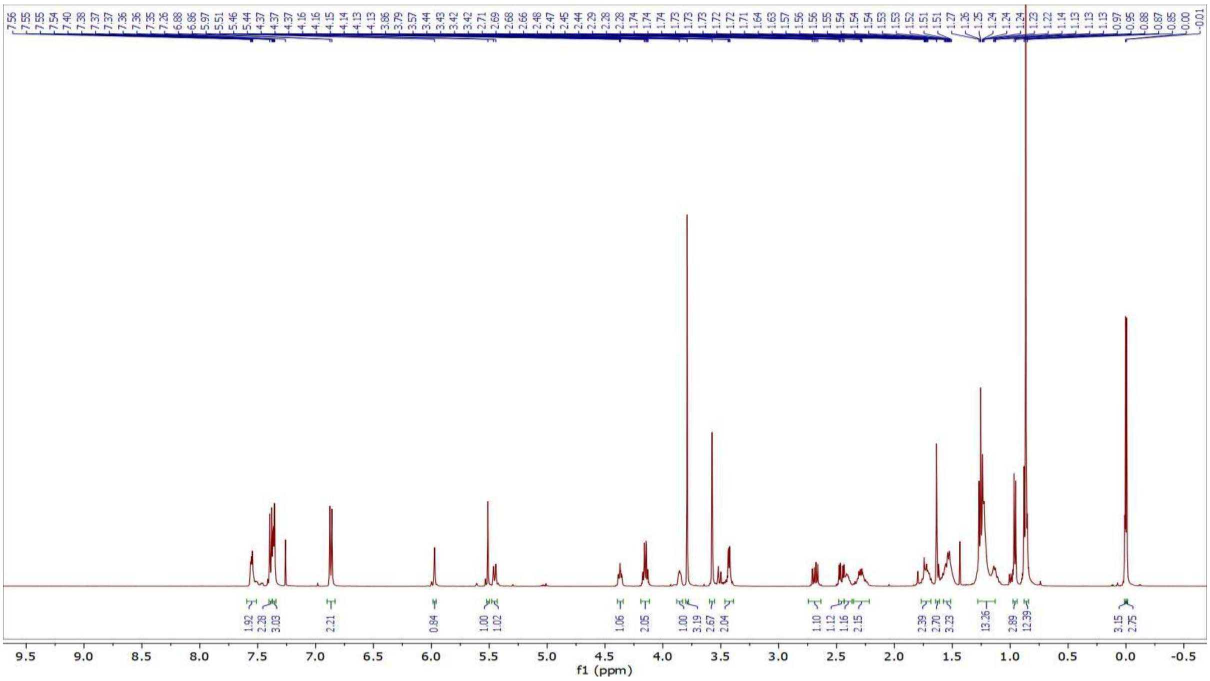

^{13}C NMR (126 MHz, CDCl$_3$)

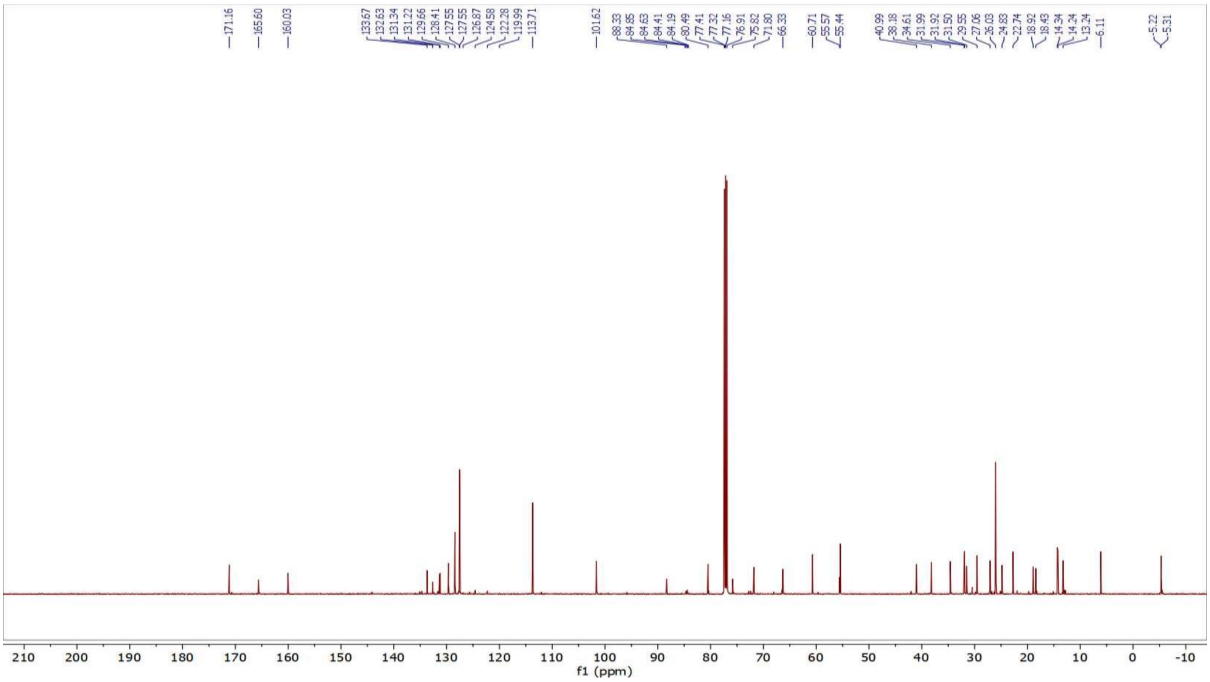

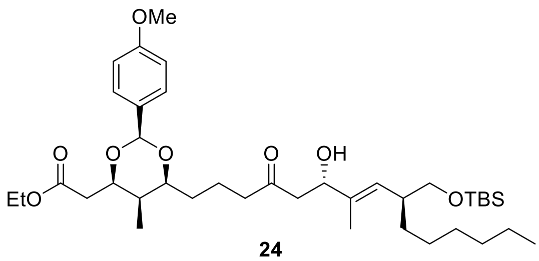

24

^{1}H NMR (400 MHz, $CDCl_3$)

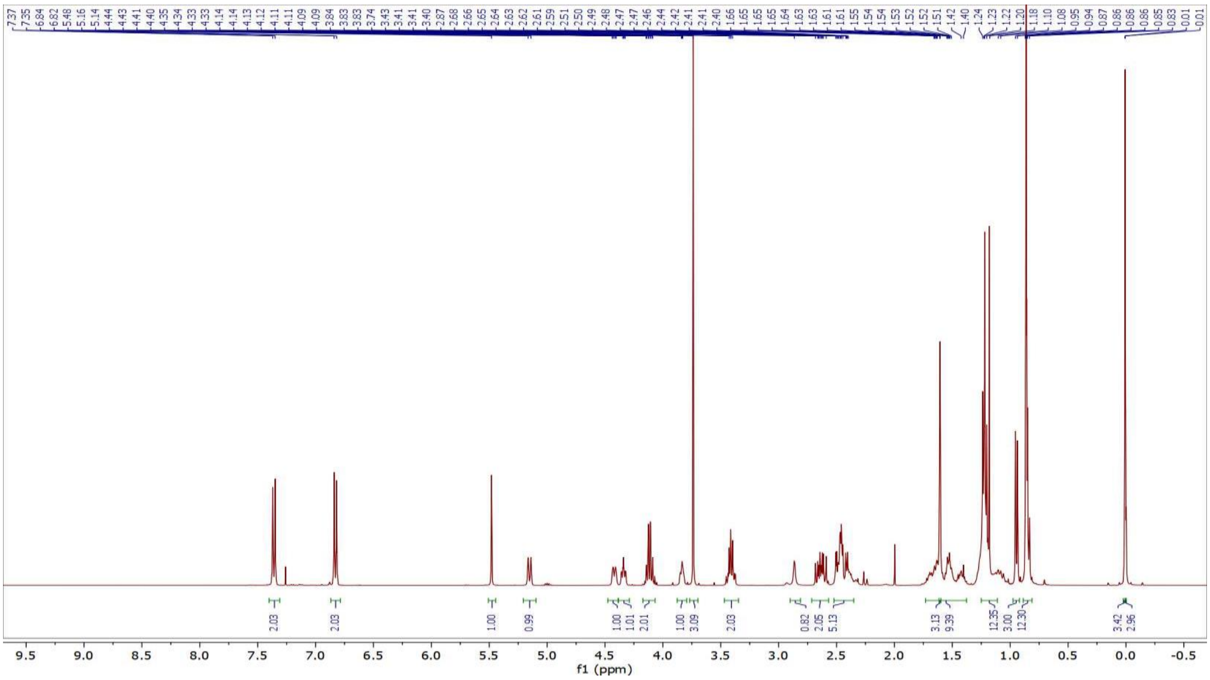

^{13}C NMR (101 MHz, $CDCl_3$)

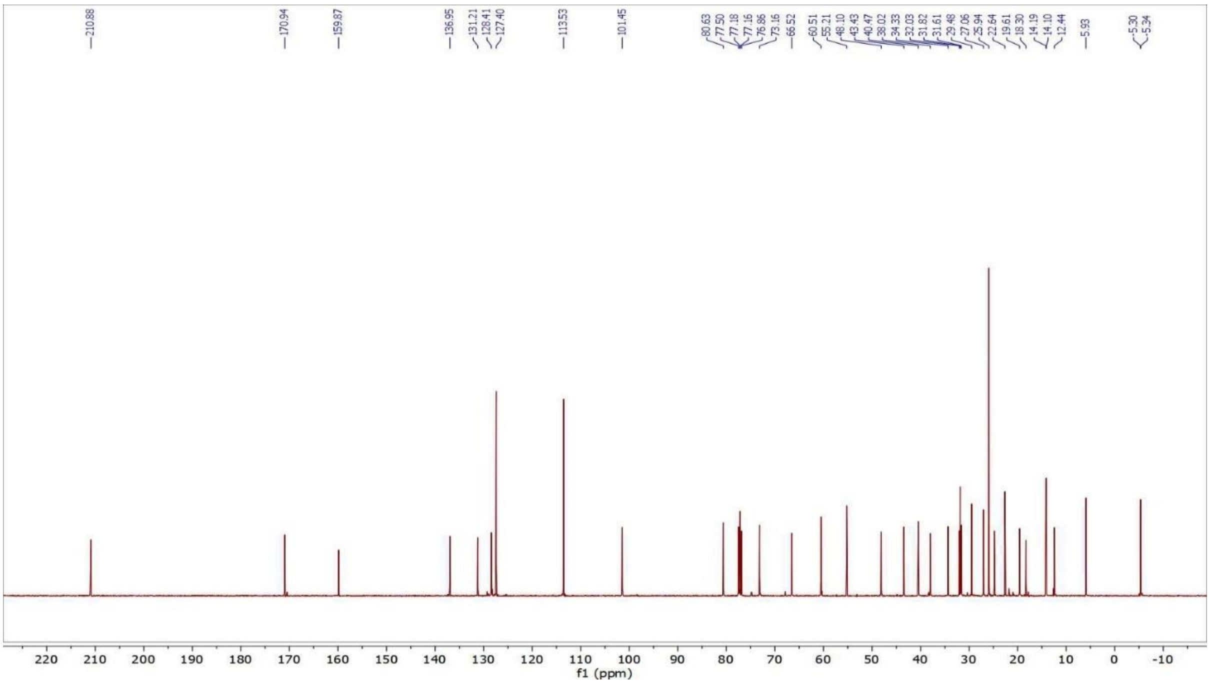

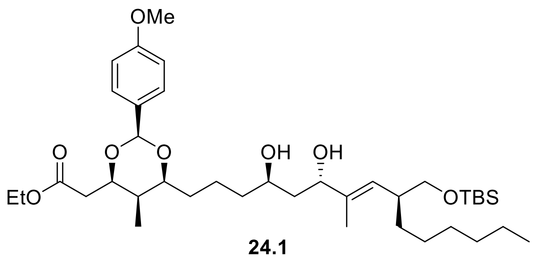

24.1

^{1}H NMR (400 MHz, $CDCl_3$)

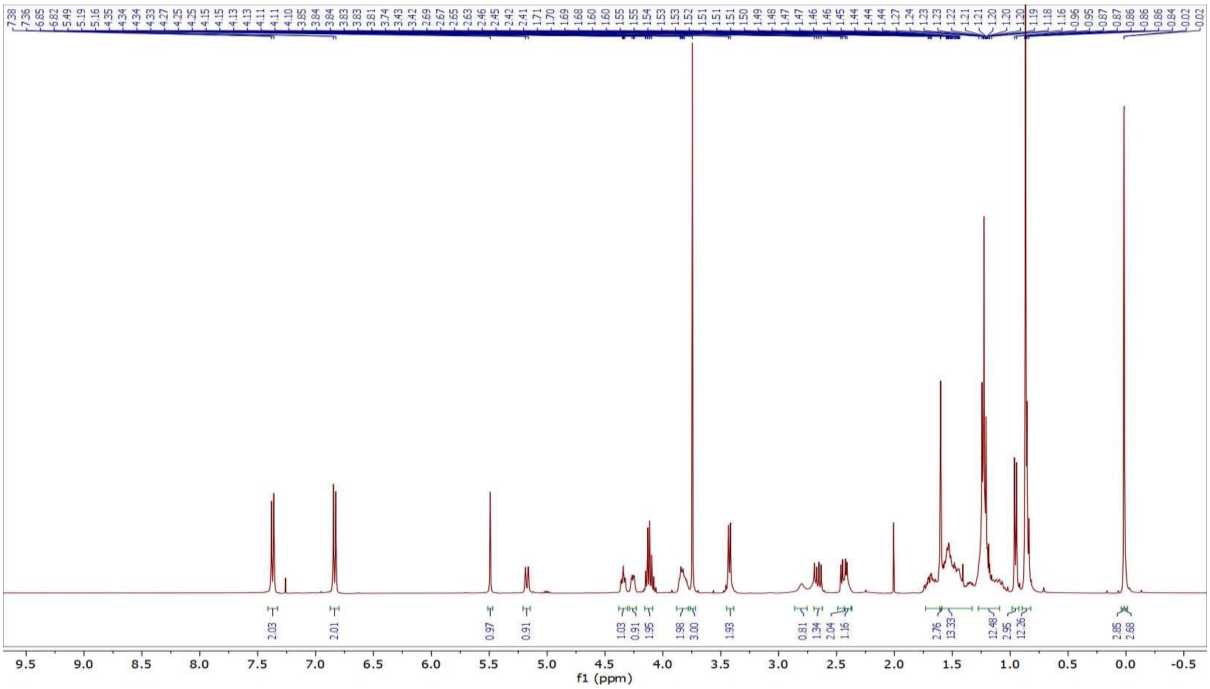

^{13}C NMR (101 MHz, $CDCl_3$)

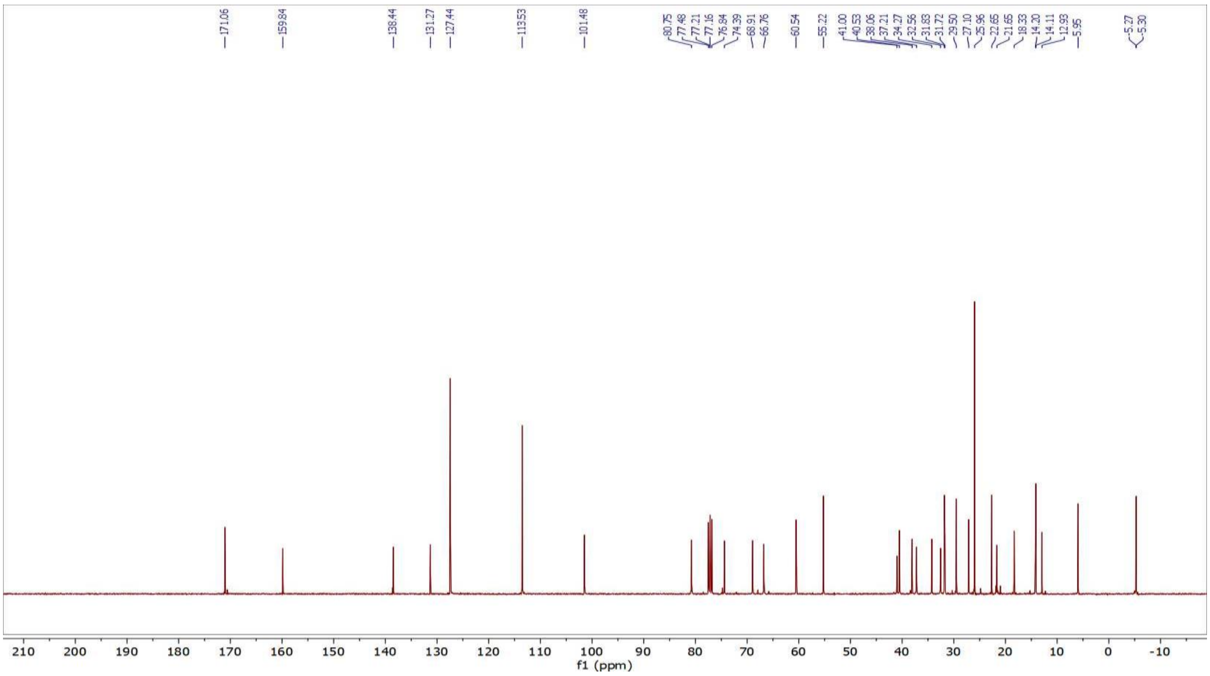

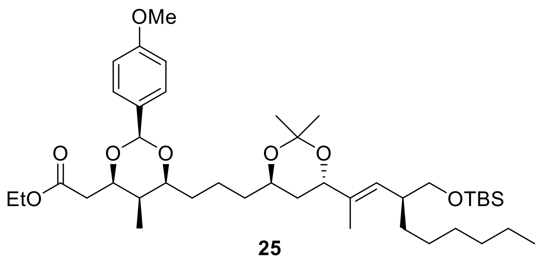

25

^{1}H NMR (400 MHz, CDCl$_3$)

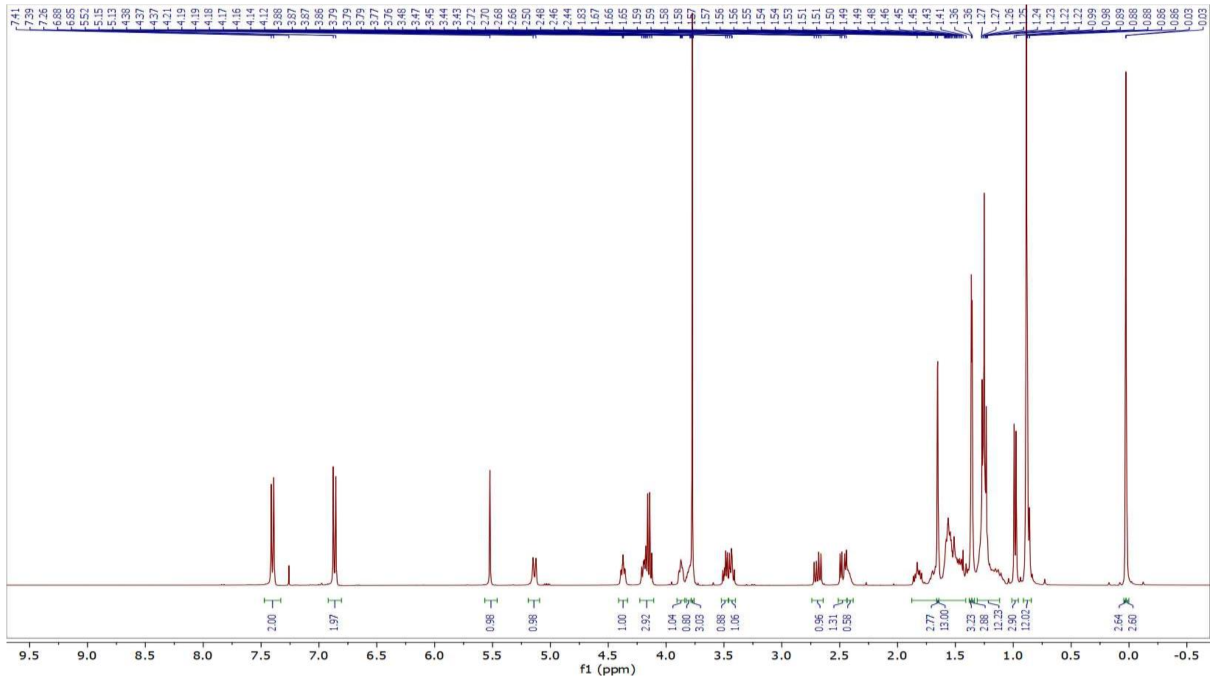

^{13}C NMR (101 MHz, CDCl$_3$)

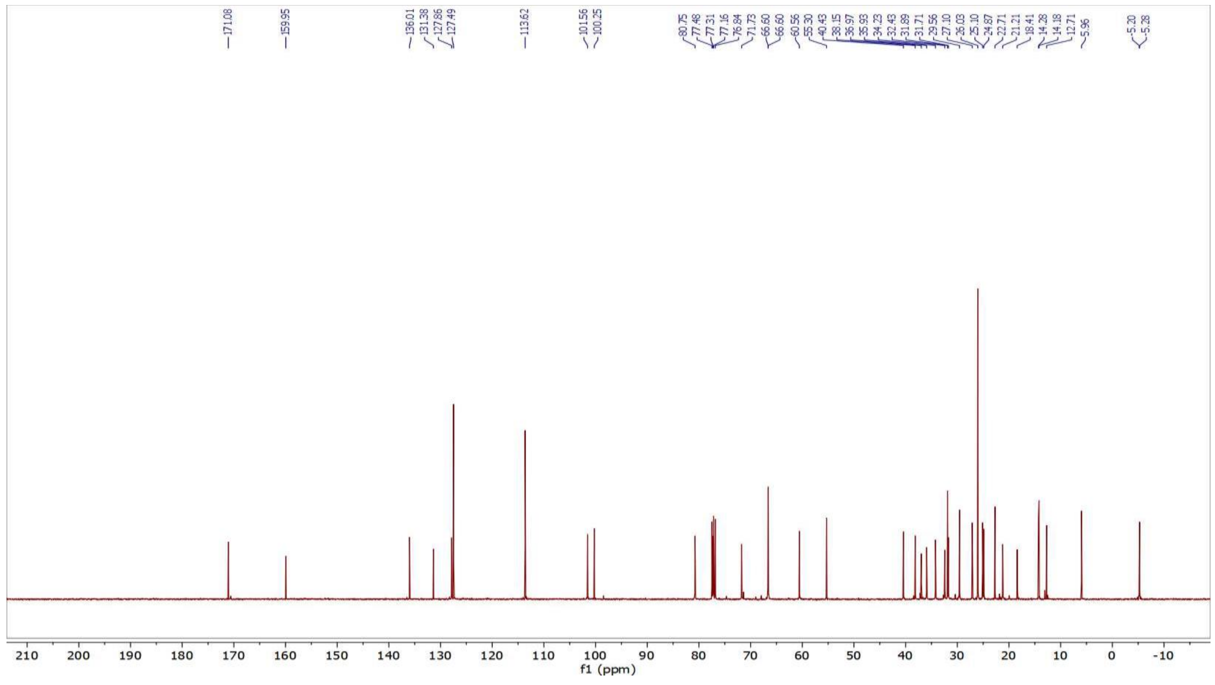

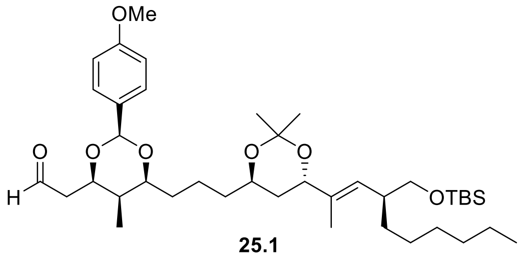

25.1

^{1}H NMR (400 MHz, $CDCl_3$)

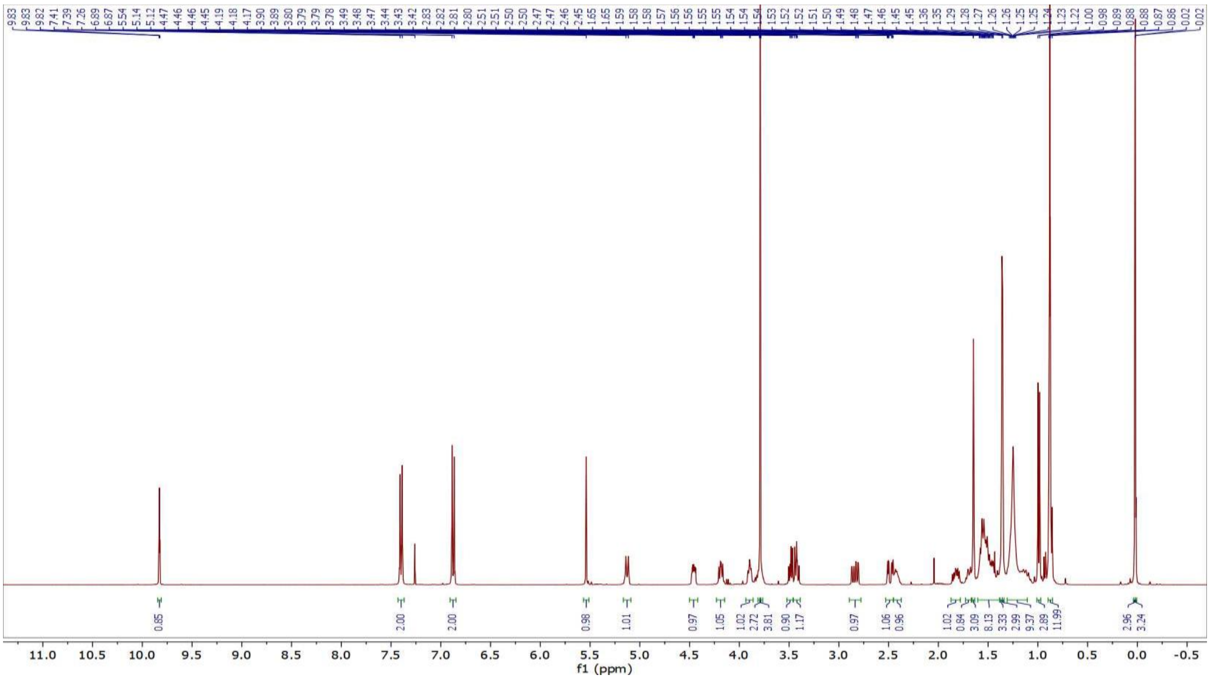

^{13}C NMR (101 MHz, $CDCl_3$)

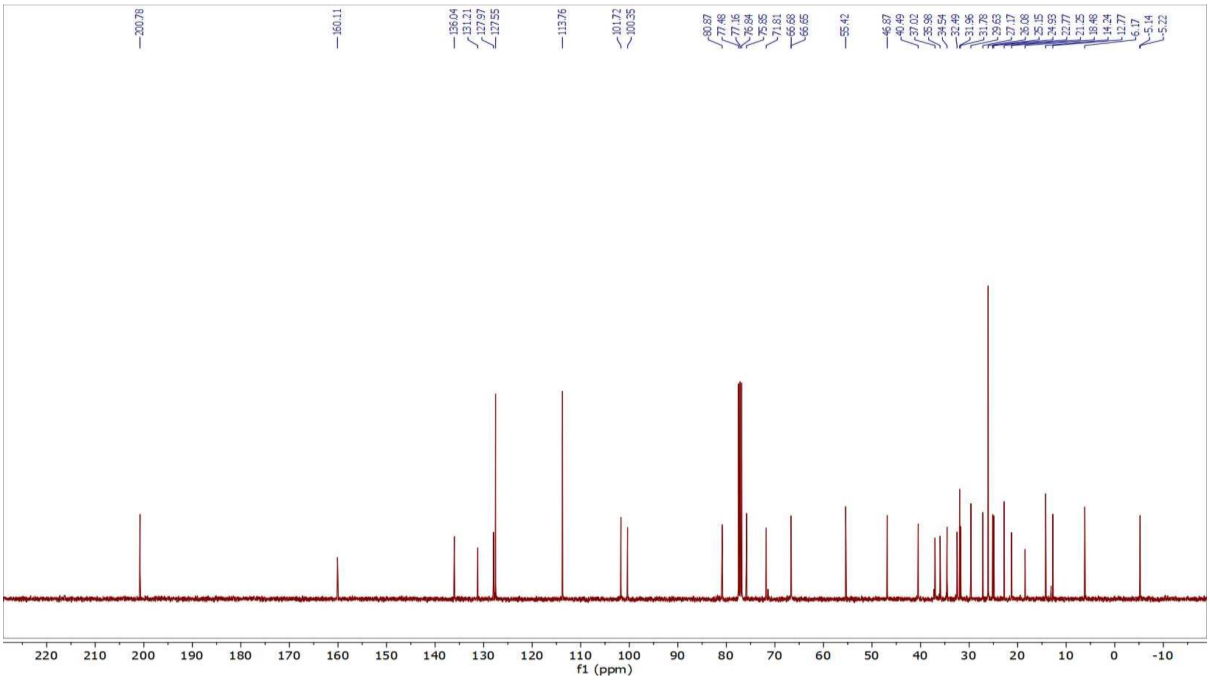

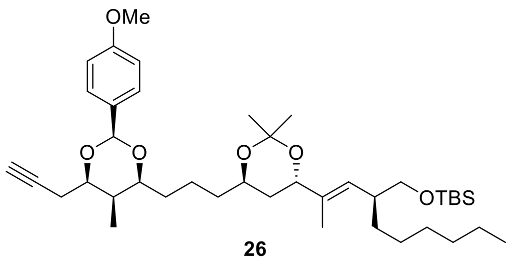

26

^{1}H NMR (400 MHz, CDCl3)

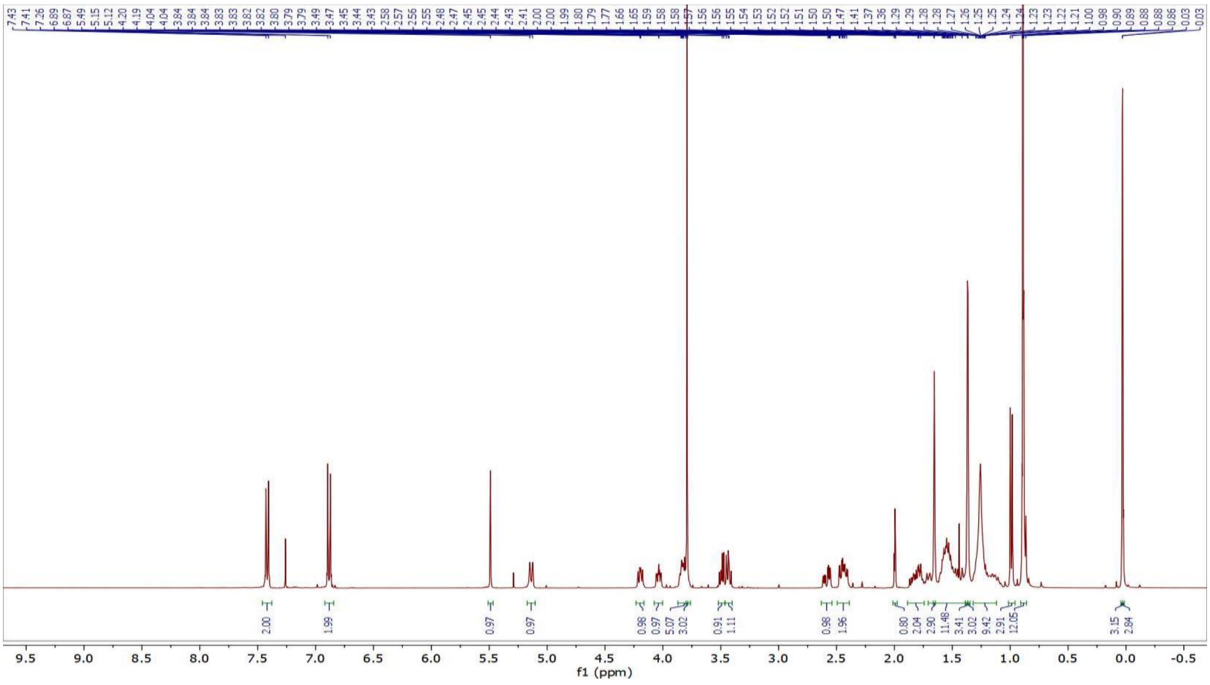

^{13}C NMR (101 MHz, CDCl3)

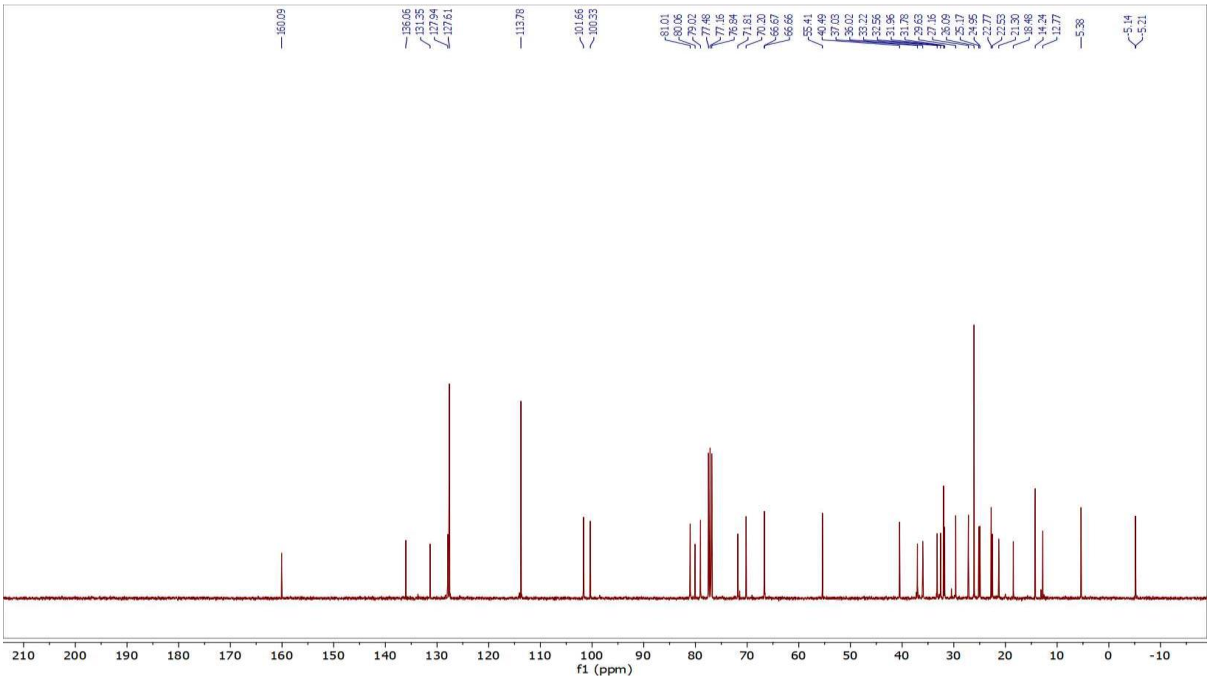

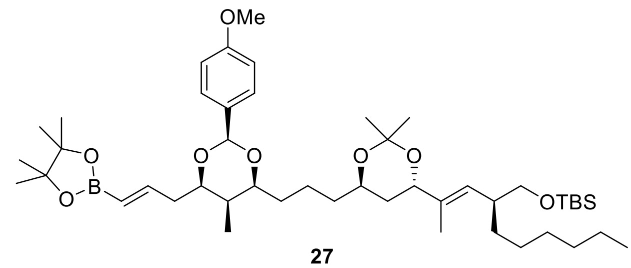

27

^{1}H NMR (400 MHz, $CDCl_3$)

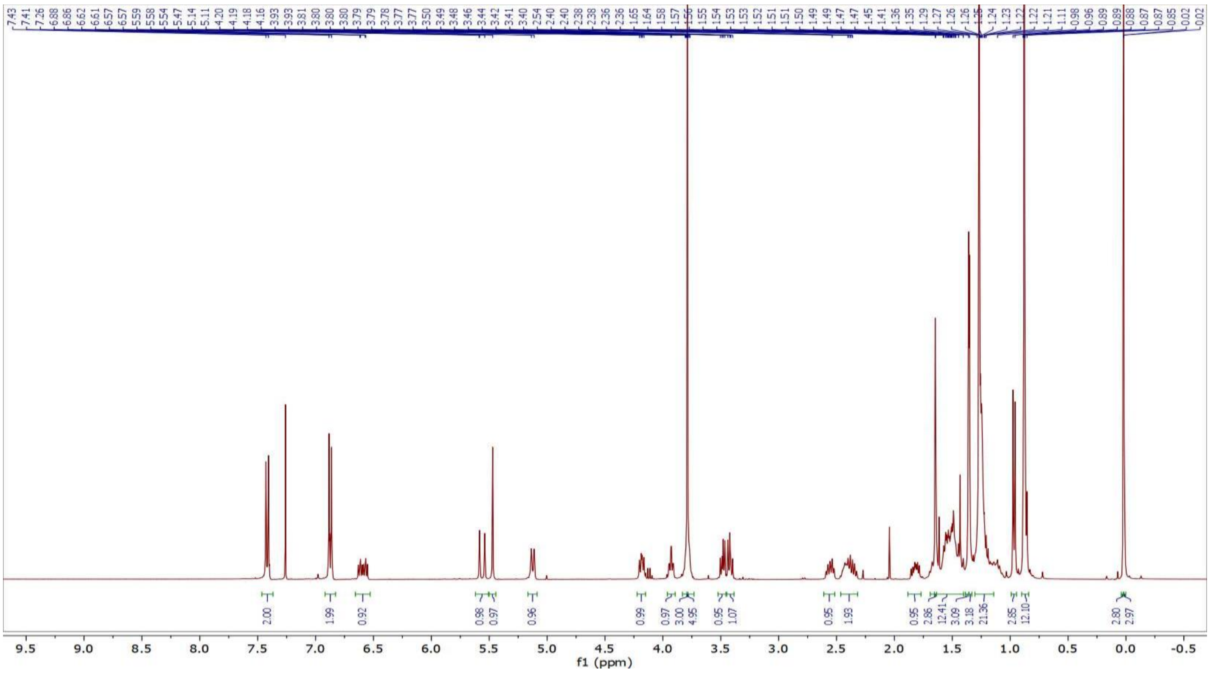

^{13}C NMR (101 MHz, $CDCl_3$)

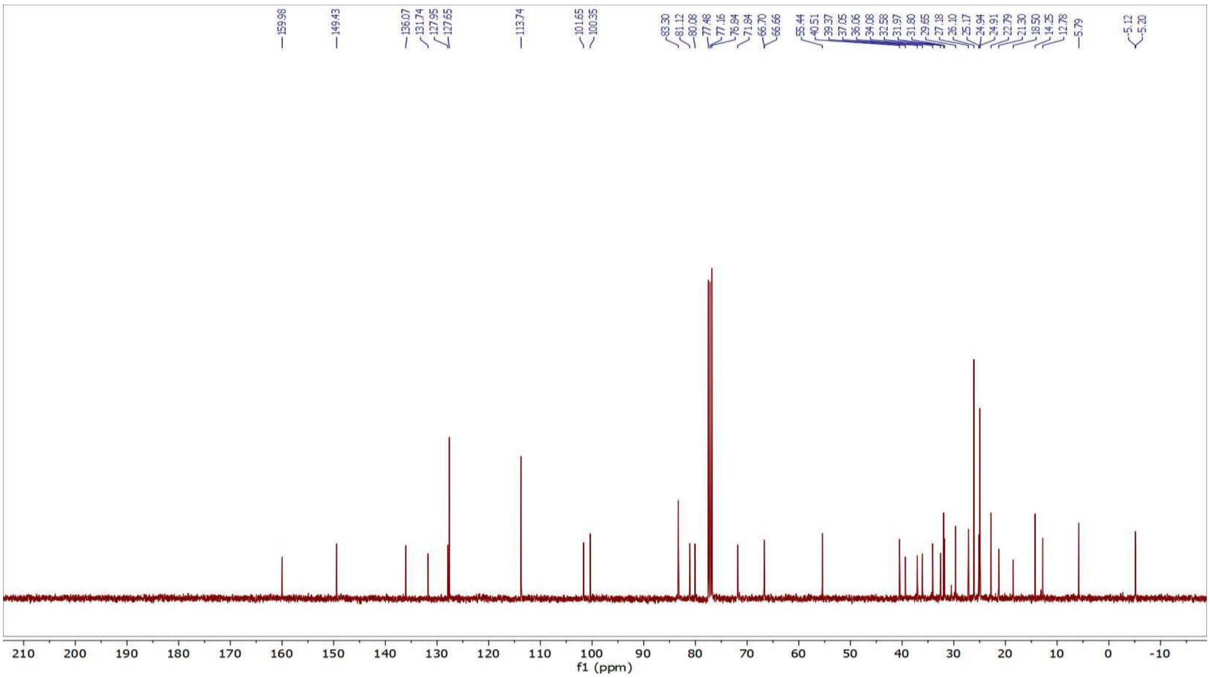

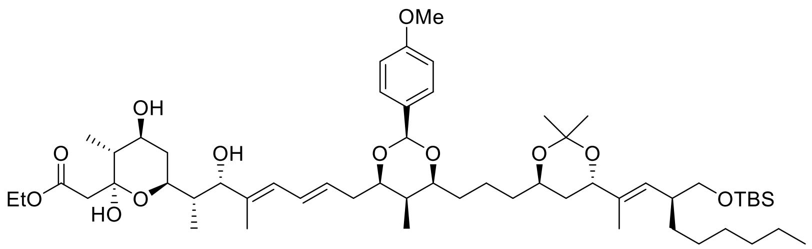

28

^{1}H NMR (400 MHz, $CDCl_3$)

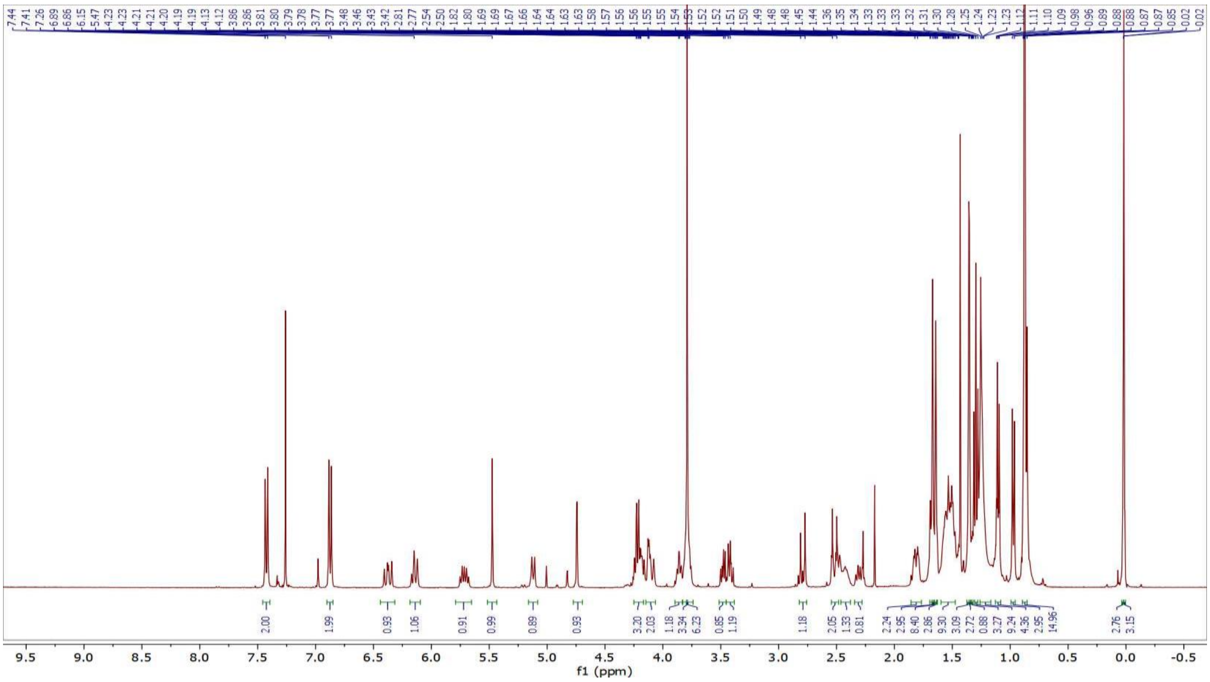

^{13}C NMR (101 MHz, $CDCl_3$)

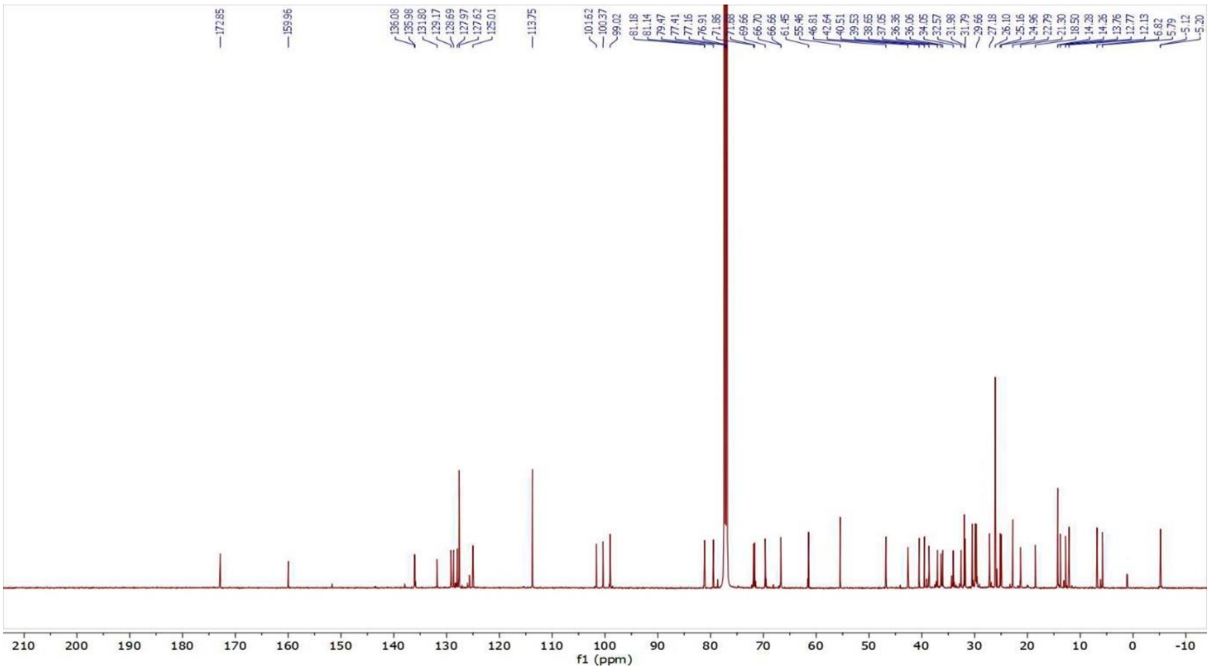

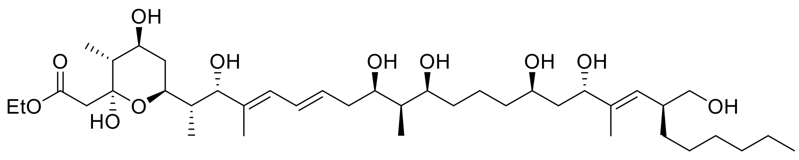

1

^{1}H NMR (600 MHz, MeOD)

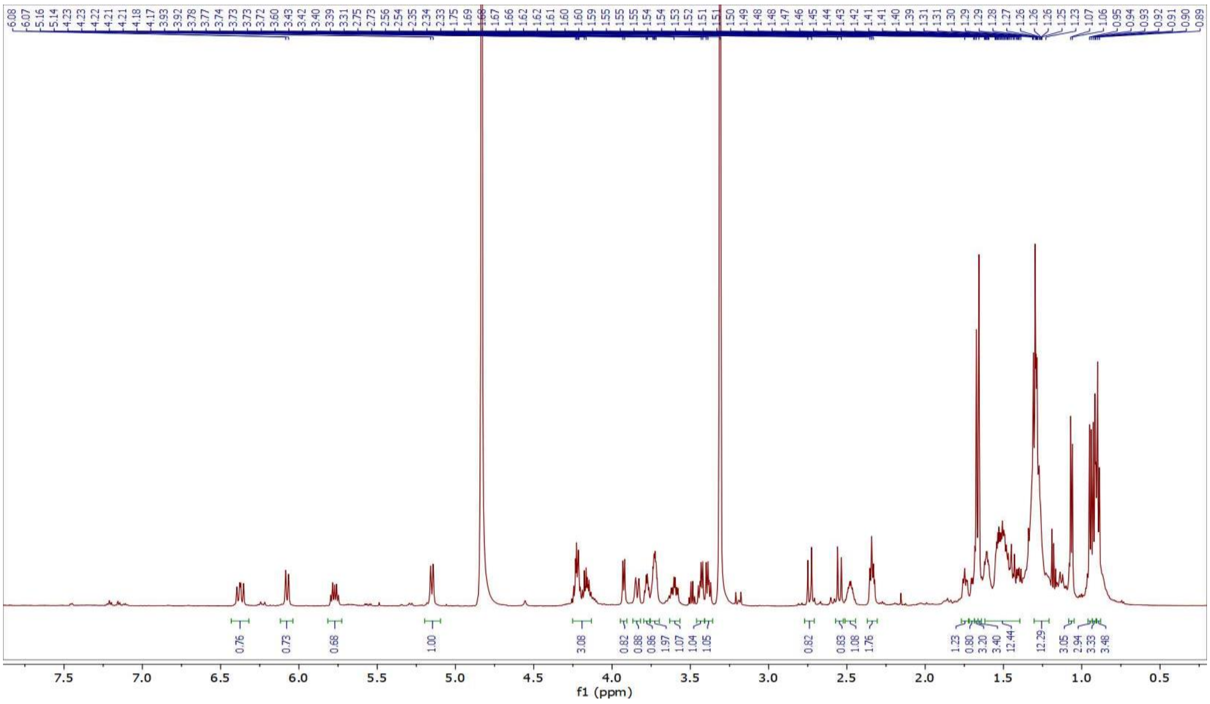

^{13}C NMR (151 MHz, MeOD)

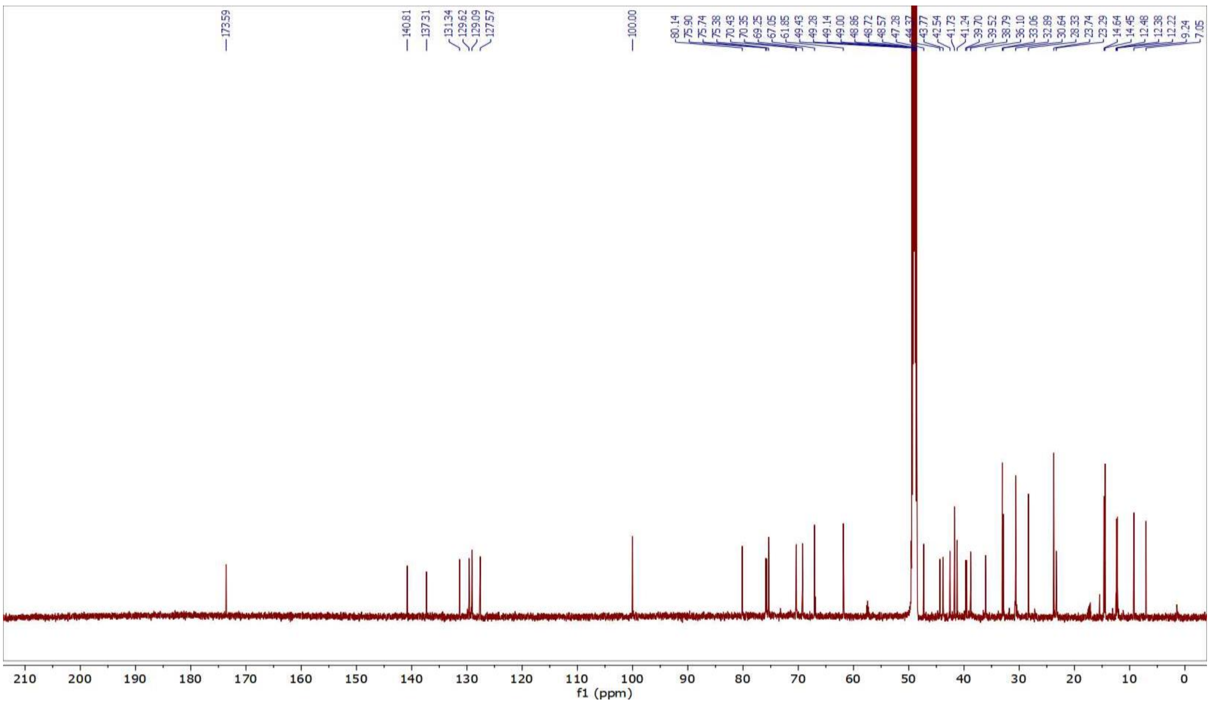

HSQC spectrum of **1**

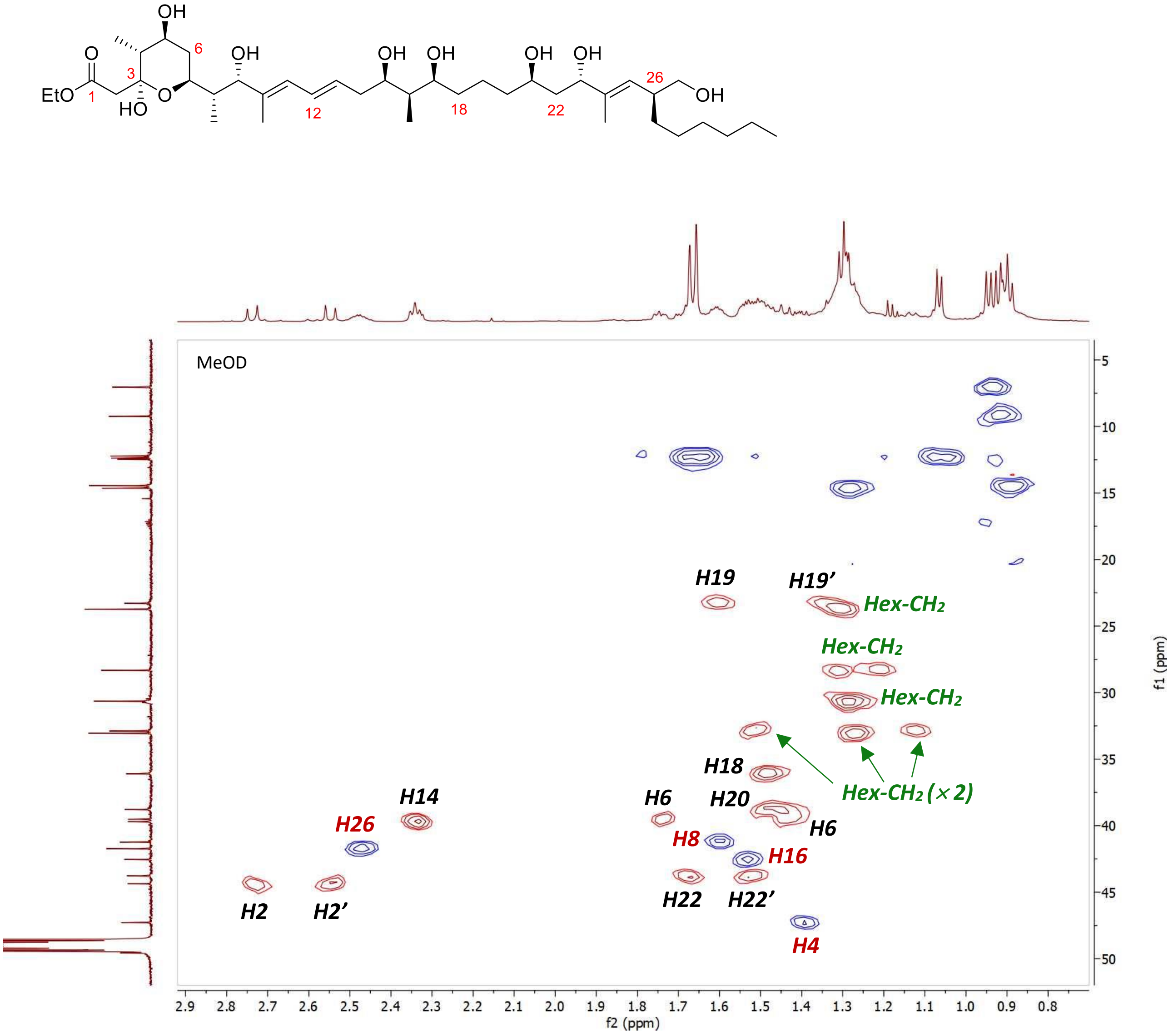

4. References

1. Menche, D.; Hassfeld, J.; Li, J.; Mayer, K.; Rudolph, S. *J. Org. Chem.* **2009**, *74*, 7220–7229.
2. Peňaška, T.; Koukal, P. Kotora, M. *Eur. J. Org. Chem.* **2018**, 147-149.
3. White, J. D.; Blakemore, P. R.; Green, N. J.; Hauser, E. B.; Holoboski, M. A.; Keown, L. E.; Kolz, C. S. N.; Phillips, B. W. *J. Org. Chem.* **2002**, *67*, 7750–7760.
4. Tekle-Smith, M. A.; Williamson, K. S.; Hughes, I. F.; Leighton, J. L. *Org Lett.* **2017**, *19*, 6024–6027.
5. Fuentes de Arriba, Á. L.; Seisdedos, D. G.; Simón, L.; Alcázar, V.; Raposo, C.; Morán, J. R. *J. Org. Chem.* **2010**, *75*, 8303–8306.
6. Suen, L. M.; Steigerwald, M. L.; Leighton, J. L. *Chem. Sci.,* **2013**, *4*, 2413–2417.
7. Hoye, T. R.; Jeffrey, C. S.; Shao, F. *Nat. Protoc.* **2007**, *2*, 2451–2458.
8. Prepared according to a literature procedure: Zhong, Y.-L.; Shing, T. K. M. *J. Org. Chem.* **1997**, *62*, 2622–2624.
9. Prepared according to a literature procedure: Bouaouli, S.; Spielmann, K.; Vrancken, E.; Campagne, J.-M; Gérard, H. *Chem. Eur. J.* **2018**, *24*, 6617–6624.
10. Majmudar, J. D.; Konopko, A. M.; Labby, K. J.; Tom, C. T. M. B.; Crellin, J. E.; Prakash, A.; Martin, B. R. *J. Am. Chem. Soc.* **2016**, *138*, 1852–1859.
11. Amoroso, J. W.; Borketey, L. S.; Prasad, G.; Schnarr, N. A. *Org. Lett.* **2010**, *12*, 2330–2333.
12. Hayashi, Y.; Gotoh, H.; Hayashi, T.; Shoji, M. *Angew. Chem. Int. Ed.* **2005**, *44*, 4212–4215.
13. Boeckman, Jr., R. K.; Biegasiewicz, K. F.; Tusch, D. J.; Miller, J. R. *J. Org. Chem.* **2015**, *80*, 4030–4045.
14. Chen, W.; Tay, J.-H.; Ying, J.; Yu, X.-Q.; Pu, L. *J. Org. Chem.* **2013**, *78,* 2256–2265.
15. Park, J. K.; Ondrusek, B. A.; McQuade, D. T. Org. Lett. 14, 2012, 4790–4793.
16. Prepared according to a literature procedure: Santoro, O.; Collado, A.; Slawin, A. M. Z.; Nolan, S. P.; Cazin, C. S. J. *Chem. Commun.* **2013**, *49*, 10483–10485.
17. Anderson, E. A.; Davidson, J. E. P.; Harrison, J. R.; O'Sullivan, P. T.; Burton, J. W.; Collins, I.; Holmes, A. B. *Tetrahedron.* **2002**, *58*, 1943–1971.
18. Kalhor-Monfared, S.; Beauvineau, C.; Scherman, D.; Girard, C. *Eur. J. Med. Chem.* **2016**, *122*, 436–441.

19. Wang, Y. D.; Kimball, G.; Prashad, A. S.; Wang, Y. *Tetrahedron Lett.* **2005**, *46*, 8777–8780.
20. Laureti, L.; Song, L.; Huang, S.; Corre, C.; Leblond, P.; Challis, G. L.; Aigle, B. *Proc. Natl. Acad. Sci. U. S. A.* **2011**, *108,* 6258–6263.
